# Paclitaxel Inhibits Synoviocyte Migration and Inflammatory Mediator Production in Rheumatoid Arthritis

**DOI:** 10.3389/fphar.2021.714566

**Published:** 2021-09-09

**Authors:** Xiaochen Chen, Haofeng Lin, Jinyang Chen, Lisheng Wu, Junqing Zhu, Yongnong Ye, Shixian Chen, Hongyan Du, Juan Li

**Affiliations:** ^1^ Department of Rheumatic and TCM Medical Center, Nanfang Hospital, Southern Medical University, Guangzhou, China; ^2^ Department of Traditional Chinese Internal Medicine, School of Traditional Chinese Medicine, Southern Medical University, Guangzhou, China; ^3^ School of Laboratory Medicine and Biotechnology, Southern Medical University, Guangzhou, China; ^4^ Department of Drug and Device Center, Huaxin Orthopaedic Hospital, Shantou University, Guangzhou, China

**Keywords:** paclitaxel, RA-FLSs, CIA, MAPK, AKT/mTOR

## Abstract

Activated fibroblast-like synoviocytes (FLSs) play a crucial role in the pathogenesis and progression of rheumatoid arthritis (RA). It is urgent to develop new drugs that can effectively inhibit the abnormal activation of RA-FLS. In our study, the RA-FLS cell line, MH7A, and mice with collagen-induced arthritis (CIA) were used to evaluate the effect of paclitaxel (PTX). Based on the results, PTX inhibited the migration of RA-FLS in a dose-dependent manner and significantly reduced the spontaneous expression of *IL-6*, *IL-8*, and *RANKL* mRNA and TNF-*α*-induced transcription of the *IL-1* *β*, *IL-8*, *MMP-8*, and *MMP-9* genes. However, PTX had no significant effect on apoptosis in RA-FLS. Mechanistic studies revealed that PTX significantly inhibited the TNF-α-induced phosphorylation of ERK1/2 and JNK in the mitogen-activated protein kinase (MAPK) pathway and suppressed the TNF-α-induced activation of AKT, p70S6K, 4EBP1, and HIF-1α in the AKT/mTOR pathway. Moreover, PTX alleviated synovitis and bone destruction in CIA mice. In conclusion, PTX inhibits the migration and inflammatory mediator production of RA-FLS by targeting the MAPK and AKT/mTOR signaling pathways, which provides an experimental basis for the potential application in the treatment of RA.

## Introduction

Rheumatoid arthritis (RA) is a chronic autoimmune disease characterized by progressive synovitis and bone destruction. Although important advances have been made in treatment of RA in recent years, existing therapies are unable to prevent the progression of RA. The treatment of refractory synovitis in RA remains tricky (Buch et al., 2021). Fibroblast-like synoviocytes (FLSs) are a prominent component of hyperplastic synovial pannus, which mediate cartilage and bone damage in RA (Bartok and Firestein, 2010). FLSs are overactivated in RA and show abnormal biological behavior, including excessive proliferation, migration, invasion, and secretion of inflammatory cytokines, such as interleukin-1β (IL-1β), interleukin-6 (IL-6), and interleukin-8 (IL-8). Increasing evidence suggests that the inhibition of the aggressive behavior of FLSs may be promising for ameliorating joint damage in RA (Masoumi et al., 2020).

Paclitaxel (PTX), a compound extracted from Taxus, is often used in cancer owing to its remarkable antitumor efficacy (Shetti et al., 2019; Xiaomeng et al., 2020; Zhang et al., 2020). Although PTX has been extensively studied in the field of oncology, researchers have found that PTX also exerts therapeutic effects on non-neoplastic diseases, such as inflammatory diseases, fibroproliferative diseases, and vascular diseases (Zhang et al., 2010; Wang et al., 2013). Our previous study showed that PTX suppressing angiogenesis in CIA mice (Xu et al., 2019). However, the effect of PTX on RA-FLS has not been illustrated. Here, we discussed the effects of PTX on RA-FLS and the effect of PTX was further validated *in vivo* using CIA mice.

## Materials and Methods

### Cell Culture

MH7A cells were obtained from the Riken Cell Bank (Tsukuba, Japan). Cells were cultured in a mixture of Dulbecco’s Modified Essential Medium (DMEM) and Ham’s F-12 medium (DMEM/F12) supplemented with 10% fetal bovine serum (FBS) in a CO_2_ incubator at 37°C. All cell culture reagents were obtained from Gibco (Thermo Fisher Scientific, MA, United States).

### Cell Viability Assay

Cell viability was determined using a Cell Counting kit-8 (CCK-8 kit, KeyGEN, Jiangsu, China). MH7A cells were seeded in a 96-well culture plate at a density of 5,000 cells/well. Cells were treated with different concentrations of PTX (Selleck, Shanghai, China) for 24 and 48 h, 10 µL of CCK-8 and 90 µL of DMEM with 10% FBS were added to each well and the plate was incubated at 37°C for 1.5 h. The optical density was measured at 450 nm using a microplate reader (Bio-Rad, United States).

### Scratch Assay

A scratch assay was used to assess cell migration. MH7A cells were seeded in 6-well plates. When the cells reached more than 90% confluent, they were scratched using pipette tips. The shedding cells were gently washed with PBS. After treatment with PTX (2.5, 5, and 10 nM) for 12 and 24 h, the scratched areas were photographed using a microscope (IX51; Olympus, Japan) and the migration distance was analyzed.

### Transwell Assay

The Boyden chamber method was used to perform the migration assay in 24-well plates with 6.5 mm diameter inserts containing 8 μm pores (Corning, NY, United States). MH7A cells were pretreated with PTX (2.5, 5, and 10 nM) for 24 h, and resuspended in serum-free medium at a final concentration of 1 × 10^6^ cells/mL. A volume of 200 µL of cell suspension was seeded in the upper chambers, and 600 µL of DMEM with 10% FBS was added to the lower wells as a chemoattractant. After incubation for 12 h, the migrated cells were fixed for 15 min in methanol and stained with 0.1% crystal violet for 15 min. Cells in the upper surface were removed from the filters. Five fields were randomly selected for cell counting and the mean number of cells was calculated.

### Quantitative Real-Time PCR Analysis

Tumor necrosis factor-α (TNF-α) (PeproTech, Rocky Hill, NJ, United States) was used to induce an inflammatory microenvironment. MH7A cells were treated with TNF-α (20 ng/ml) and PTX (2.5, 5, and 10 nM) for 24 h, total RNA was extracted using Trizol (Invitrogen, San Diego, CA, United States), and cDNA was reverse-transcribed according to the protocol of the TAKARA reverse transcription kit (Takara Biotechnology, Dalian, China). The ABI-7500 Thermal Cycler (Applied Biosystems, Foster City, CA, United States) was used for quantitative real-time polymerase chain reaction (PCR) to evaluate cytokine expression. The relative gene expression was calculated using the ΔΔCt method (Livak and Schmittgen, 2001).

### Flow Cytometry

The apoptosis of MH7A cells was assessed using a flow cytometer (FACSCalibur BD LSRFortessa ^TM^, United States). Cells were treated with PTX (100 and 200 nM) for 24 and 48 h. According to the manufacturer’s instructions, cells were collected and stained with Annexin V-fluorescein isothiocyanate (FITC) and propidium iodide (PI) for 15 min at 4°C in the dark using a FITC-Annexin V/PI Apoptosis Detection Kit (Multisciences Bio, China). The apoptotic rate was calculated using Flow-Jo V10. Three replicates were employed for the flow cytometry assay.

### Western Blotting

MH7A cells were treated with TNF-α (20 ng/ml) and PTX (5 and 10 nM) for 12 h. The total protein concentrations were detected using BCA Protein Assay Kit (Thermo Fisher Scientific, MA, United States). Based on the protein concentration, 40 µg of protein were separated using 10% SDS-PAGE and transferred onto a polyvinylidene fluoride (PVDF) membrane. The membranes were blocked with 5% bovine serum albumin (BSA) for 60 min and incubated overnight at 4°C with primary antibodies on a rotary shaker. The membranes were then incubated for 1 h with secondary antibodies. Antibody binding was detected using an enhanced chemiluminescence detection kit (Beyotime Biotechnology, Shanghai, China). The western blotting results were quantified using ImageJ. The primary antibodies included p38 MAPK, JNK, Erk1/2, AKT, mTOR, p70S6K, 4E-BP1, and HIF-α and their corresponding phosphorylation antibodies, phospho-p38 MAPK (Thr180/Tyr182), phospho-ERK1/2 (Thr183/Tyr185), phospho-ERK1/2 (Thr202/Tyr204), phospho-AKT (Ser473), phospho-mTOR (Ser2448), phospho-p70S6K (Thr389), and phospho-4E-BP1 (Ser65). The primary and secondary antibodies were purchased from Cell Signaling Technology (United States).

### Induction of Collagen-Induced Arthritis (CIA)

Six-to eight-week-old male DBA/1 mice were obtained from Beijing Vital River Experimental Animal Technique Co. Ltd (Beijing, China) and raised under standard laboratory conditions. The mice were divided into three groups: group 1 (control, nonimmunized and untreated); group 2, CIA (vehicle, positive control); group 3, CIA + PTX (PTX-treated group). Arthritis was induced in mice of group 2 and group 3 according to a previously described protocol (Grotsch et al., 2019). Bovine type II collagen (CII) was dissolved in 0.1 M acetic acid to a concentration of 4 mg/ml, and then emulsified in equal volumes of complete Freund’s adjuvant (CFA) on ice. Mice were administered 0.1 ml of this collagen emulsion. After 21 days, mice received a second immunization with 0.1 ml CII emulsified in incomplete Freund’s adjuvant (IFA). Mice in group 3 were intraperitoneally (*i.p.*) administered 1.5 mg/kg PTX every 2 days, and mice in group 2 received an equal volume of solvent. CII, CFA, and IFA were purchased from Sigma-Aldrich (St. Louis, MO, United States).

### Arthritis and Body Weight Assessment

After the second immunization, body weight of mice was recorded once a week, and the arthritis index was measured every 3 days after the second immunization by two independent investigators who was blind to the mice. The severity of arthritis was assessed by visual evaluation using a score on a scale of 0–4: 0, no evidence of erythema and swelling; 1, erythema and mild swelling confined to the tarsals or ankle joint; 2, erythema and mild swelling extending from the ankle to the tarsals; 3, erythema and moderate swelling extending from the ankle to metatarsal joints; 4, erythema and severe swelling encompassing the ankle, foot, and digits, or ankylosis of the limb. The total scores of hind paws represented arthritis score for each mouse.

### Histopathological Examination

Mice were sacrificed on day 80 of the experiment, and their spleen, liver, and paws were collected, weighed, and fixed in 4% paraformaldehyde for 48 h. The paws were decalcified in 10% EDTA, dehydrated, and embedded in paraffin. About 5 µM-thick sections were made and stained with hematoxylin and eosin (H&E), tartrate-resistant acid phosphatase (TRAP), safranine-O and fast green. The spleen and liver sections were fixed in the same manner as paws, without decalcification, and were stained with H&E. The histological scores were assessed by two independent investigators, who were blind to the identity of the specimens, and the average of two scores was used. The histological score for paws was determined according to the previous research and was mainly based on synovitis, pannus, and bone destruction (Xu et al., 2019). The spleen pathology was evaluated according to the degree of lymphoid nodule hyperplasia, marginal area hyperplasia and red medullary hyperemia (Du et al., 2019; Du et al., 2020).The pathological scores of liver were graded on a scale of 0–4 according to the degree of liver lesions (Lande and Hill, 1988): 0, normal or mild degeneration of the liver, and no necrosis of liver cells; 1, punctate necrosis, accounted for less than 1/4 of the hepatic lobule; 2, focal necrosis, accounted for 1/4–1/3 of the hepatic lobule; 3, focal necrosis, accounted for 1/3–1/2 of the hepatic lobule; 4, patchy necrosis, necrosis over 1/2 of hepatic lobule.

### Micro-computed Tomography (Micro-CT) Analysis

The paws were collected after mice were sacrificed. The attachments around paws were removed carefully, and paws were fixed in 4% paraformaldehyde. Then, they were scanned using micro-CT (SCANO Medical AG, Switzerland), and the images of paws in 3D were reconstructed. Bone parameters such as bone mineral density (BMD) and bone volume/tissue volume (BV/TV) of paws were obtained and analyzed with µCT RAY V4.0.

### Statistical Analysis

Data are presented as mean ± standard deviation (SD). All data were analyzed using Prism 8 (GraphPad Software, San Diego, CA, United States). Student’s *t*-test was used to analyze statistical differences between the two groups. One-way or two-way ANOVA was applied to determine the statistical significance between the results of control and treatment groups. Statistical significance was set at *p* < 0.05.

## Results

### PTX Inhibits the Migration of RA-FLS

**FIGURE 1:**
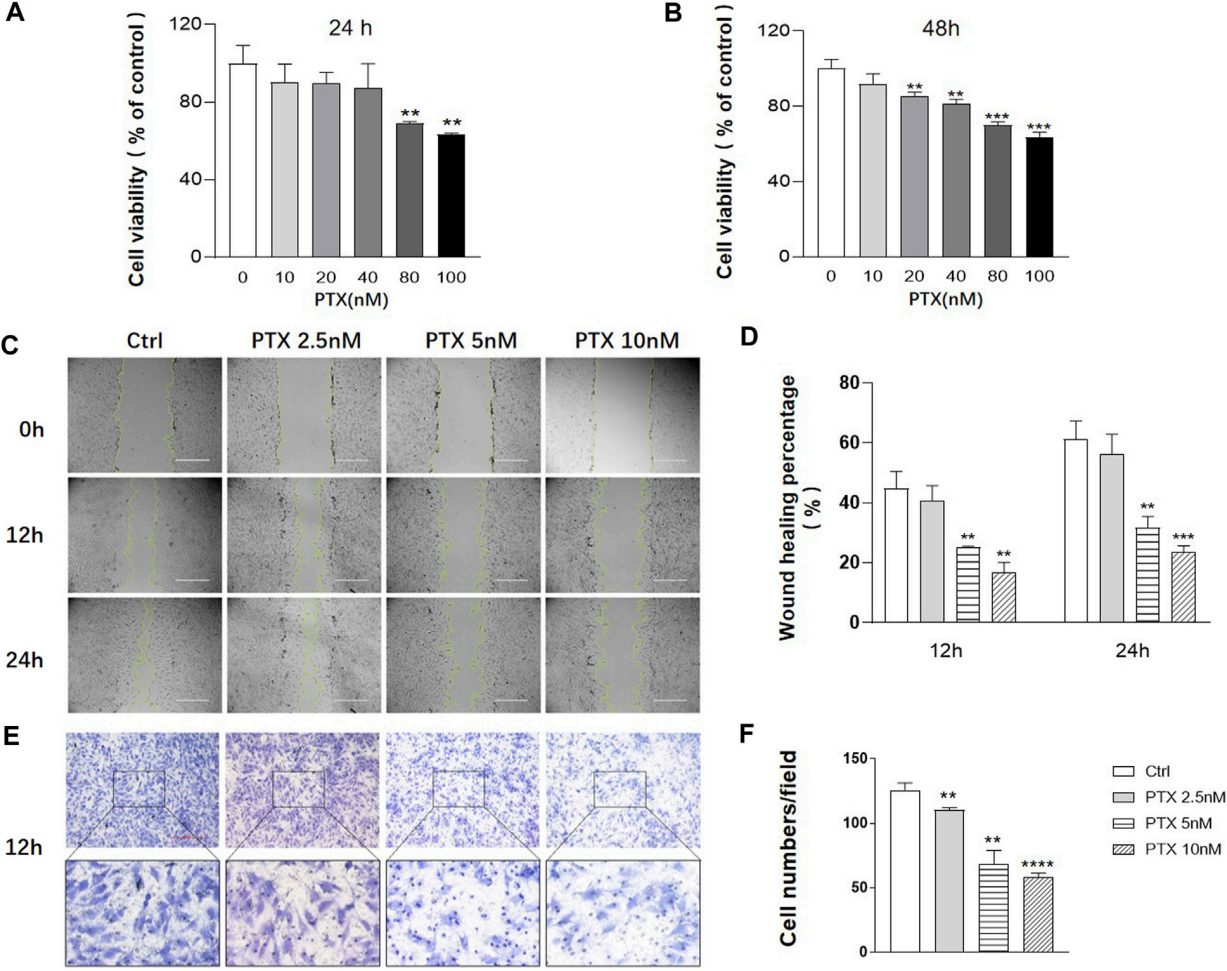
PTX inhibits the migration of RA-FLS. **(A**–**B)** MH7A cells were treated with the indicated concentrations of PTX for 24 and 48 h. Cell viability was analyzed using the CCK-8 assay. **(C**–**D)** Effect of PTX on cell migration was detected using a scratch assay. The scratching area was photographed at 12 and 24 h. The scratch assay was presented as the percentage by which the original scratch width decreased at each measured time point. **(E**–**F)** Transwell assay was conducted with a Boyden chamber, and the migrated cells were photographed. Five fields were randomly selected for cell counting, the mean number of cells was analyzed. Data are expressed as mean ± SD (n = 3) ^*^ *p* < 0.05, ^**^ *p* < 0.01, ^***^ *p* < 0.001 *vs* Ctrl.

Cell Counting Kit-8 (CCK-8) assay was used to assess MH7A cell viability after PTX intervention for 24 and 48 h. When the cells were exposed to 0–40 nM PTX for 24 h or 0–10 nM PTX for 48 h, there was no significant change in cell viability (Figures 1A,B). To minimize the toxicity of PTX, the concentration range of 0–10 nM was employed for subsequent experiments.

To evaluate the effects of PTX on cell migration, the scratch and transwell assays were conducted. The result of scratch assay showed that 2.5, 5 and 10 nM PTX can inhibit the migration of RA-FLS in a dose-dependent manner (Figures 1C–D). And the result was further confirmed by transwell assay (Figures 1E,F).

### PTX has No Significant Effect on the Apoptosis of RA-FLS

**FIGURE 2:**
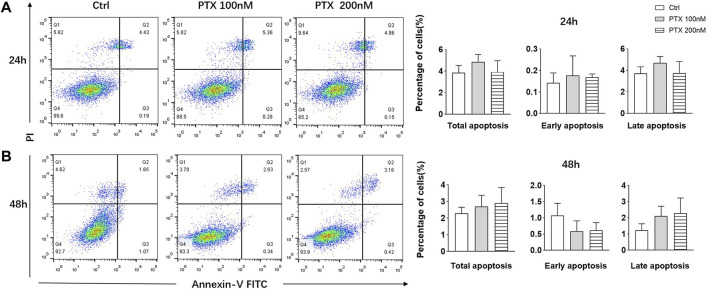
PTX had no significant effect on the apoptosis of RA-FLS. **(A)** RA-FLS apoptosis was measured using the annexin V-FITC/PI staining assay after PTX treatment for 24 and 48 h. **(B)** Total apoptosis, early apoptosis and late apoptosis rate of RA-FLS were analyzed. Data are expressed as mean ± SD (n = 3).

Studies have shown that PTX are able to induce apoptosis of tumor cells (Zhao et al., 2019; Xiaomeng et al., 2020); therefore, we explored whether PTX could induce RA-FLS apoptosis using Annexin-FITC/PI staining. After treating RA-FLSs with 100 and 200 nM PTX for 24 and 48 h, apoptosis was examined by flow cytometry analysis. However, the results showed that no obvious change in cell apoptosis rates after treatment, despite the use of a high PTX concentration (Figure 2).

### PTX Suppresses the Inflammatory Mediator Production of RA-FLS

TNF-α plays a key role in synovial inflammation; it induces the activation of RA-FLS and promote the release of inflammatory factors, such as IL-1, IL-6 and IL-8, etc. These proinflammatory mediators are widely involved in synovitis and bone destruction. RA-FLS also secrete a variety of matrix metalloproteinases (MMPs) to drive joint destruction, among these, MMP-2, MMP-3 and MMP-9 are critical factors, which can directly destroy type II collagen and thus promote cartilage destruction. The interplay of receptor activator of nuclear factor-κB (RANK), its ligand RANKL and osteoprotegerin (OPG) are established regulators of bone metabolism. The balance of OPG and RANKL expression regulates osteoclast formation (Gravallese, 2002).

**FIGURE 3:**
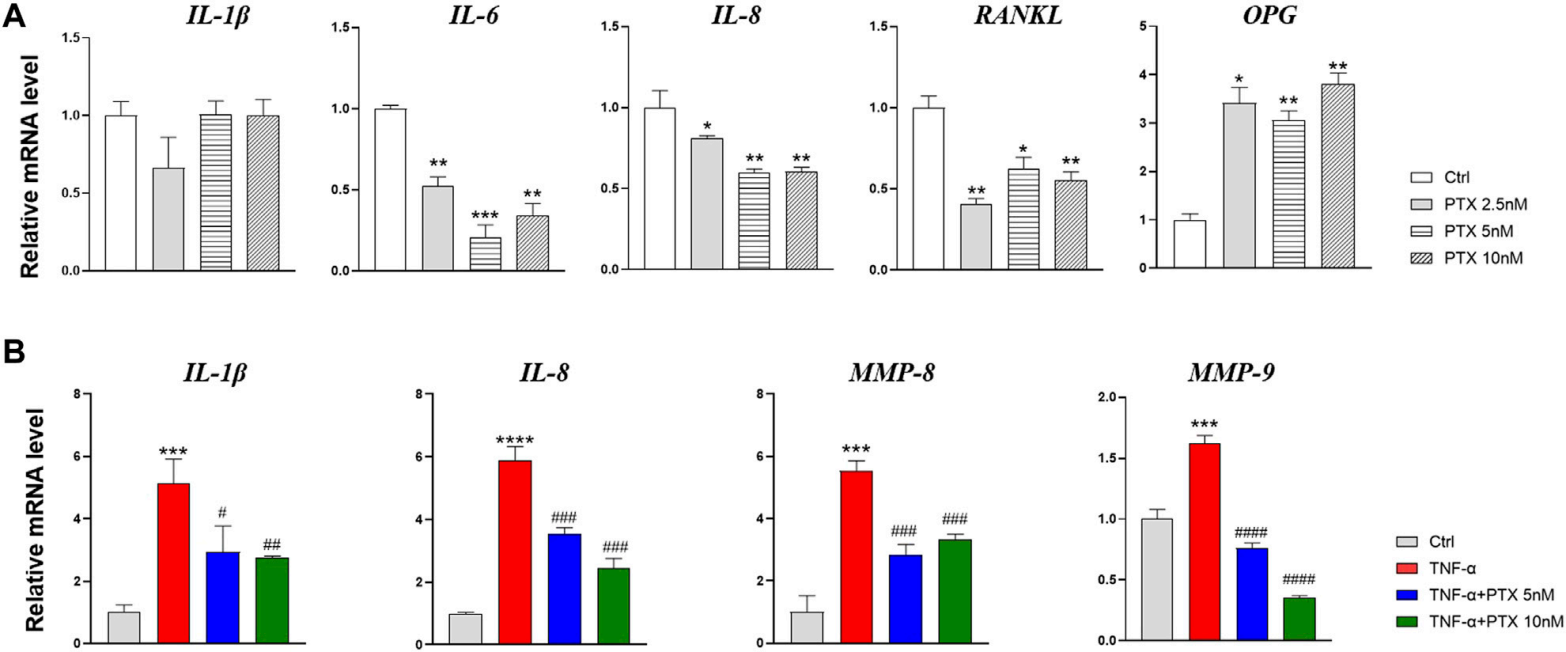
PTX suppresses the inflammatory mediator production of RA-FLS. **(A)** The relative mRNA expression of *IL-1β, IL-6, IL-8, RANKL* and *OPG* were detected by qRT-PCR after treatment with PTX (2.5, 5 and 10 nM). **(B)** The relative mRNA expression of *IL-1β, IL-6, IL-8, MMP-8* and *MMP-9* were detected by qRT-PCR after treatment with PTX (5 and 10 nM) and TNF-α (20 ng/ml). Data are expressed as mean ± SD (n = 3). ^*^ *p* < 0.05, ^**^ *p* < 0.01, ^***^ *p* < 0.001, ^****^ *p* < 0.0001 *vs* Ctrl group. ^#^ *p* < 0.05, ^##^ *p* < 0.01, ^###^ *p* < 0.001, ^####^ *p* < 0.0001 *vs* TNF-α group.

To explore the effect of PTX on inflammatory mediators in RA-FLS, we checked the mRNA expression of inflammatory factors using qRT-PCR. As shown in Figure 3A, the expression of *IL-6, IL-8,* and *RANKL* was suppressed by PTX, while the *OPG* transcription level increased. However, the expression of *IL-1β* mRNA did not significantly change. As shown in Figure 3B, TNF-α induced the transcription of *IL-1β, IL-8*, *MMP-8*, and *MMP-9* genes; however, after PTX treatment, the expression of all these genes were all downregulated.

### PTX Suppresses the Activation of the MAPK and AKT/mTOR Pathways

**FIGURE 4:**
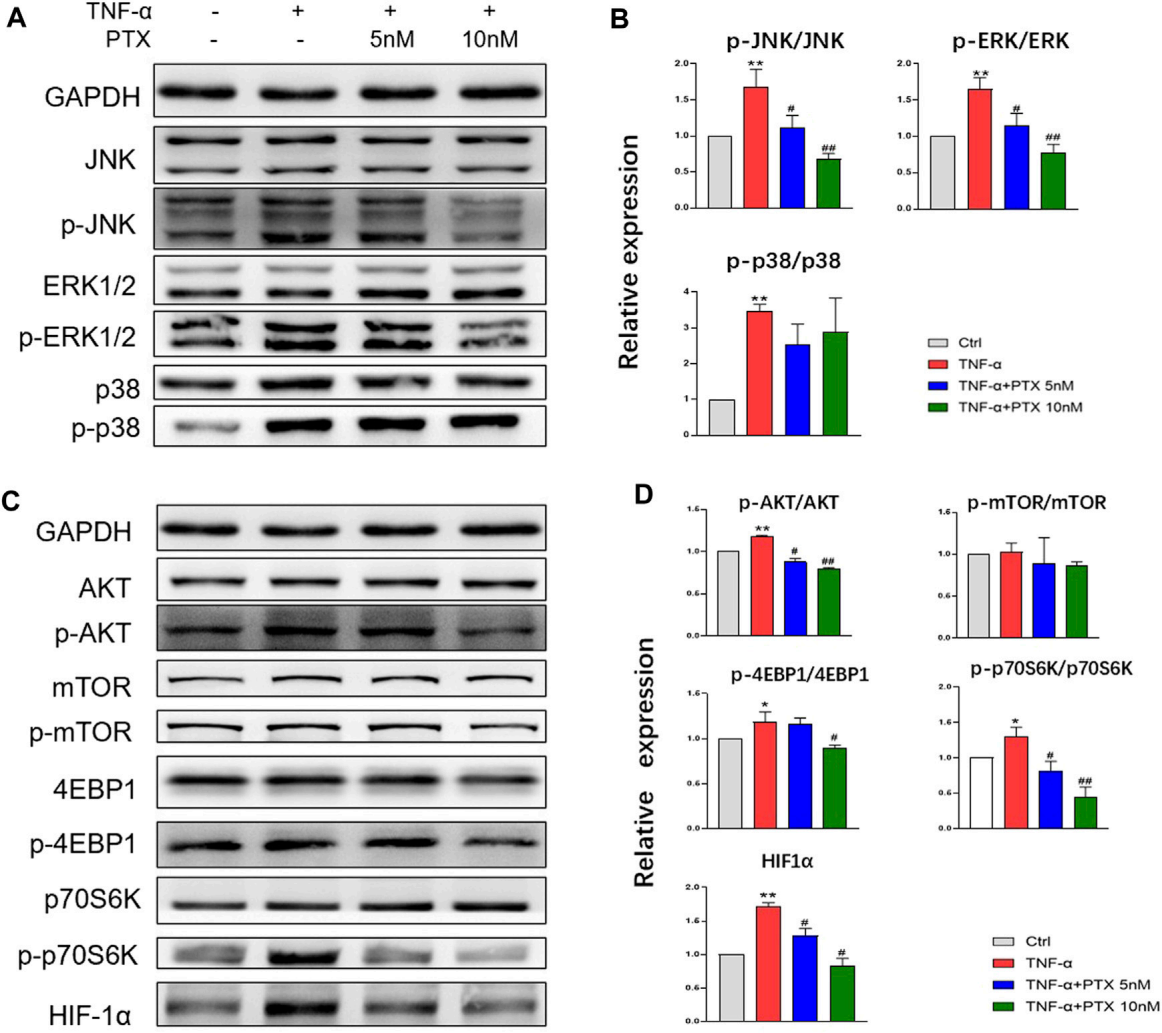
PTX suppresses the activation of the MAPK and AKT/mTOR pathways. **(A**–**B)** MH7A cells were treated with TNF-α and PTX (5 and 10 nM). The protein expression levels of p38, ERK1/2, and JNK and the corresponding phosphorylated proteins were examined by western blotting. **(C**–**D)** MH7A cells were treated with TNF-α and PTX (5 and 10 nM). The protein expression levels of phosphorylated/total AKT, mTOR, p70S6K, 4EBP1, and HIF-1α were examined by Western blot analysis. Data are expressed as mean ± SD (n = 3). ^*^ *p* < 0.05, ^**^ *p* < 0.01 *vs* Ctrl group. ^#^ *p* < 0.05, ^##^ *p* < 0.01 *vs* TNF-α group.

To investigate the protein targets of PTX, expression levels of indicated proteins were detected using western blotting. The MAPK and AKT/mTOR signaling pathways play important roles in the regulation of cell growth, differentiation and some other cellular functions (Huang et al., 2004; Malemud, 2015). The MAPK pathway mainly composed of p38 MAPK, ERK1/2 and JNK. TNF-α significantly promoted the phosphorylation of p38 MAPK, JNK and ERK1/2, whereas PTX significantly decreased the phosphorylation of JNK and ERK1/2, however, PTX had no significant effect on p38 expression (Figures 4A,B). 4E-BP1, p70S6K and HIF-1α are important downstream proteins of the AKT/mTOR pathway. As shown in Figures 4C,D, PTX reduced the expression of TNF-α-induced AKT, 4E-BP1, p70S6K, and HIF-1α in the AKT/mTOR pathway; however, no obvious changes were observed in mTOR expression. Thus, PTX may modulate the activation of the MAPK and AKT/mTOR pathways.

### PTX Attenuates Synovitis in CIA Mice

**FIGURE 5:**
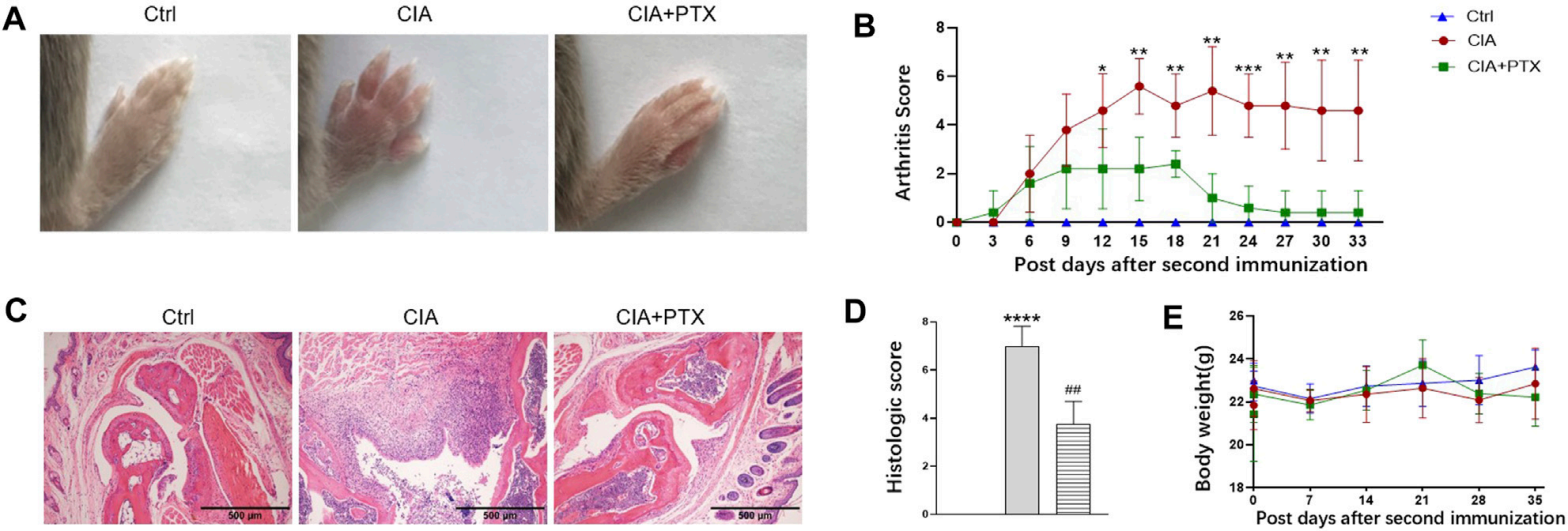
PTX attenuates synovitis in CIA mice. CIA mice were *i.p.* administered 1.5 mg/kg PTX or solvents every 2 days after the second immunization. **(A)** Representative images of the paws of the mice in the three groups. **(B)** The arthritis score was recorded every 3 days after the second immunization. **(C)** H&E staining of paws was used for histological evaluation. **(D)** Histological scores of the paws, based on H&E staining. **(E)** Body weight of mice in the three groups. Data are expressed as mean ± SD (n = 5). ^*^ *p* < 0.05, ^**^ *p* < 0.01, ^***^ *p* < 0.001, ^****^ *p* < 0.0001 *vs* Ctrl group. ^#^ *p* < 0.05, ^##^ *p* < 0.01 *vs* CIA group.

To further determine the therapeutic effect of PTX *in vivo,* we used a mouse model of RA. Compared with the control group, PTX significantly alleviated the redness and swelling of inflamed paws, and the arthritis scores of mice in PTX group were lower than those in CIA group. In addition, we examined pathological changes in paws by H&E staining, the mice in CIA group showed inflammatory infiltration and synovial membrane hyperplasia, and PTX reduced inflammatory infiltration and synovial membrane hyperplasia (Figure 5C). Compared with the model group, histological scores for paws were remarkably decreased in PTX group (Figure 5D). During the experimental period, we found no significant difference in body weight among mice in the three groups (Figure 5E).

### PTX Protects Against Bone Destruction in CIA Mice

**FIGURE 6:**
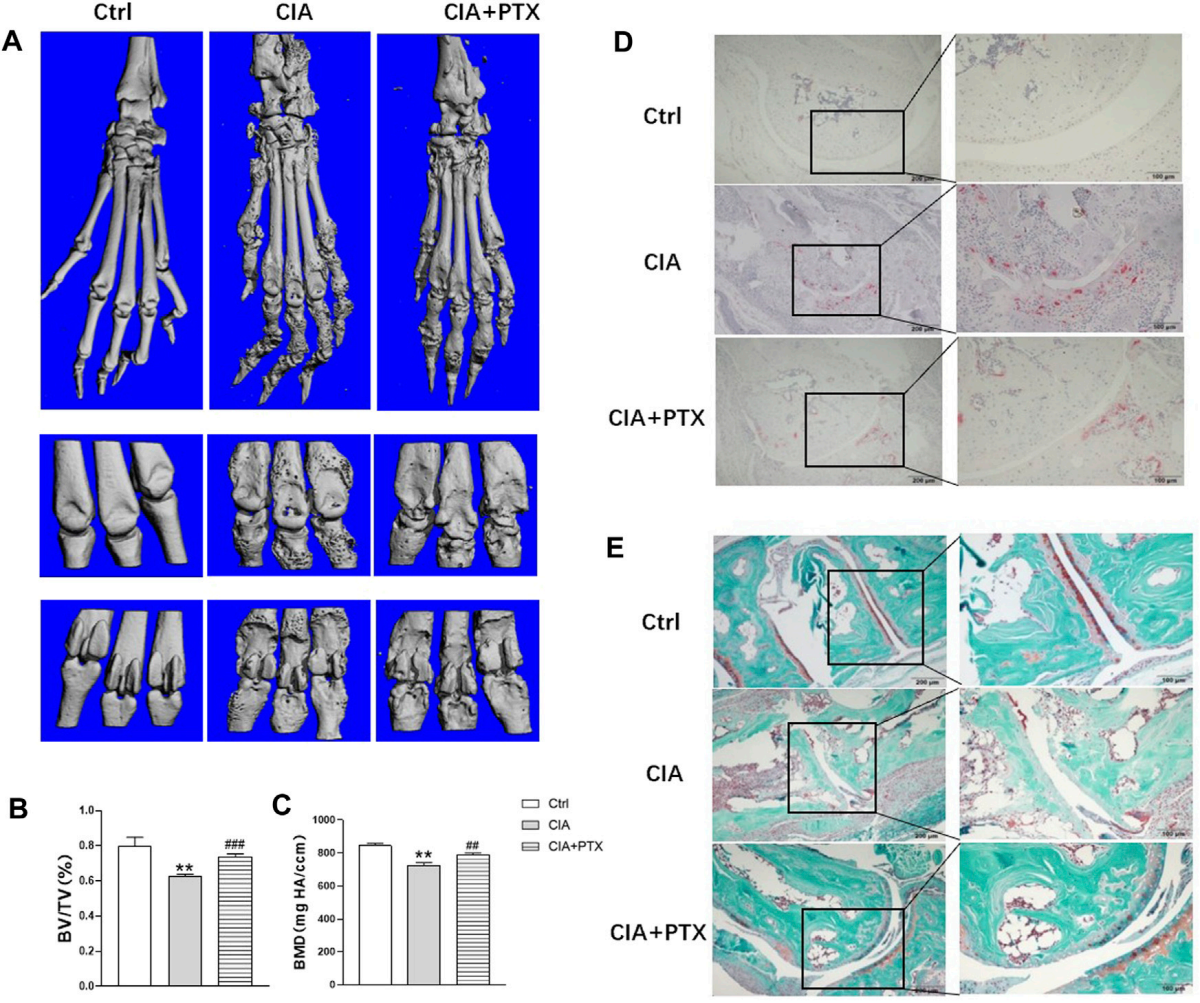
PTX protects against bone destruction in CIA mice. **(A)** Micro-CT scanning images of paws and joints. **(B**–**C)** The bone volume/tissue volume (BV/TV) and bone mineral density (BMD) were analyzed. **(D)** TRAP staining was used to evaluate osteoclasts. **(E)** Safranine-O/fast green staining was employed to assess cartilage damage. Data are expressed as mean ± SD (n = 5). ^*^ *p* < 0.05, ^**^ *p* < 0.01 *vs* Ctrl group. ^#^ *p* < 0.05, ^##^ *p* < 0.01 *vs* CIA group.

Furthermore, we evaluated the effect of PTX on bone destruction in mice. Micro-CT was employed and bone parameters, such as bone mineral density (BMD) and bone volume/tissue volume (BV/TV) were obtained to evaluate bone destruction of paws. Compared with the control group, mice in CIA group suffered significant bone damage, and mice in PTX group showed less bone erosion (Figure 6A). Meanwhile, BMD and BV/TV in the CIA group were lower than the control group, while these parameters were higher in the PTX group than in the CIA group (Figures 6B,C). In addition, TRAP staining was performed to identify osteoclasts, the TRAP-positive (+) cells were stained red. As shown in Figure 6D, osteoclasts in PTX group were notably reduced compared with CIA group, which suggest that PTX could significantly reduce the number of osteoclasts. Safranin-O/fast green staining was also performed, staining the bone tissue in turquoise (green) and the cartilage tissue in red. The results showed an increase in the cartilage area after PTX treatment (Figures 6D,E).

### PTX Alleviates Inflammation of the Spleen and Liver

**FIGURE 7:**
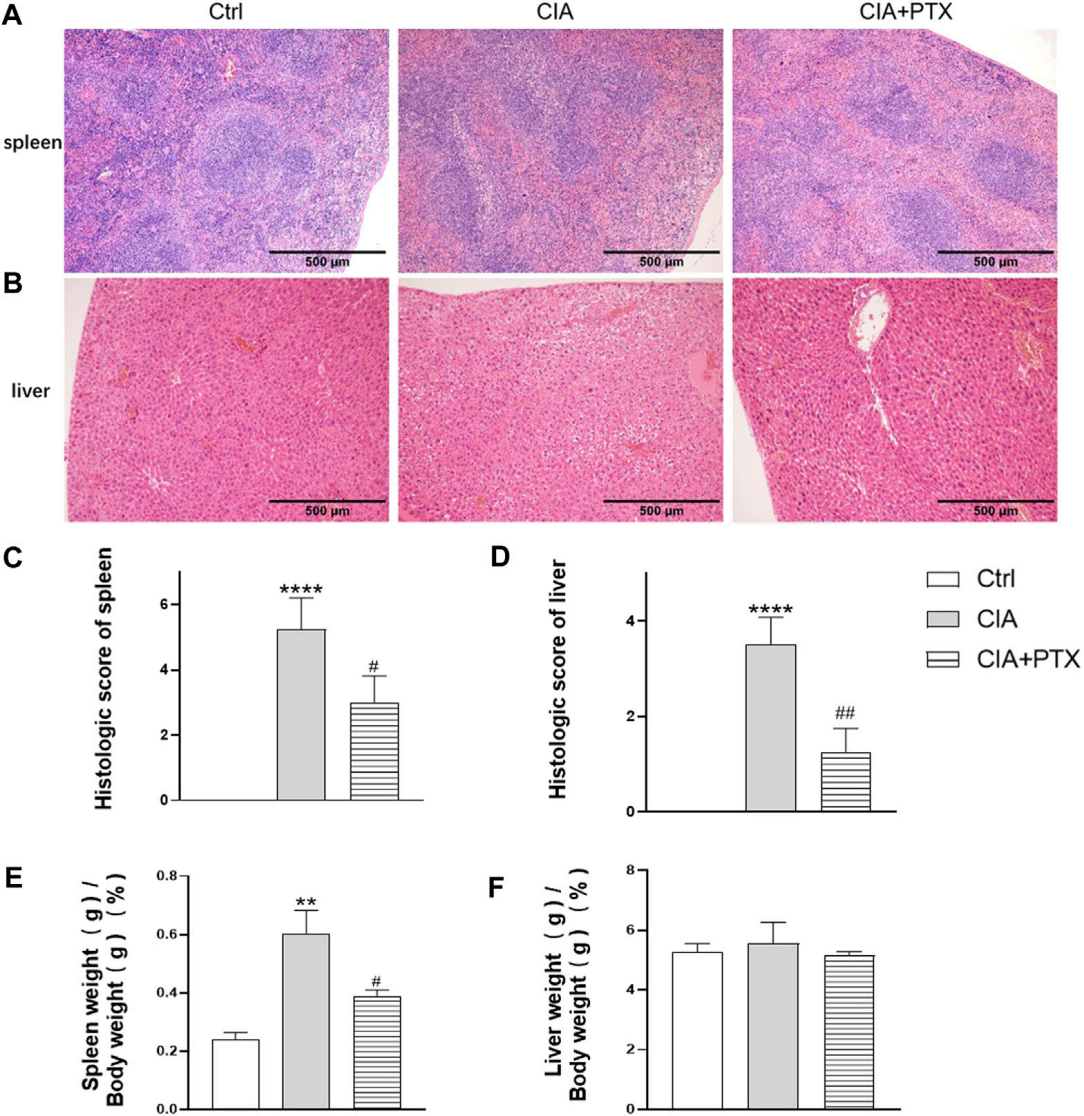
PTX alleviates inflammation of the spleen and liver of CIA mice. **(A**–**B)** Hematoxylin–eosin staining of the liver and spleen was used for pathological evaluation. **(C**–**D)** The histologic score of the spleen and liver. **(E**–**F)** The spleen and liver indexes are shown as spleen or liver weight (g)/body weight (g) × 100%. Data are expressed as mean ± SD (n = 5). ^**^ *p* < 0.01, ^****^ *p* < 0.0001 *vs* Ctrl group. ^#^ *p* < 0.05, ^##^ *p* < 0.01 *vs* CIA group.

Except for joint inflammation, CIA mice are often show systematic immune response, while liver and spleen are important immune organs, our previous studies have revealed that mice with CIA have significantly increased liver and spleen index and pathological changes. To determine the effect of PTX on liver and spleen inflammation, H&E staining was used for pathological evaluation. Compared with the CIA group, the ratio of spleen white pulp and inflammatory cells, such as neutrophils, in the PTX group was significantly reduced (Figure 7A). The liver cells in the PTX group had less hepatocyte edema, as shown in Figure 7B, and the hepatic portal area was less dilated and congested.

## Discussion

In this study, we demonstrated that PTX could inhibit the migration and expression of inflammatory cytokines in RA-FLS, which may be related to the inhibition of the MAPK and AKT/mTOR pathways. Furthermore, *in vivo* experiments indicated that PTX could alleviate synovitis and bone destruction based on collagen-induced arthritis.

RA-FLS migration and invasion play important role in synovitis and bone destruction. Synoviocytes not only migrate locally, but also invade distant areas and joints through the bloodstream. At the cartilage-bone junction where the synovium meets the cartilage surface, when the cartilage surface is damaged, ECM is exposed and RA-FLSs are recruited for migration to the site. RA-FLSs secrete MMPs that further aggravate the matrix degradation of cartilage, ultimately resulting in bone erosion. Studies have shown that PTX can inhibit the migration of tumor cells (Sun K. et al., 2015; Shetti et al., 2019). The current study also showed that PTX inhibited RA-FLS migration in a dose-dependent manner.

In RA patients, innate immunity and adaptive immunity are activated, inducing the release of a variety of cytokines. Such release promotes FLS expressing inflammatory mediators, such as IL-6 and IL-8, ultimately forming a cascading inflammatory response (Scott et al., 2010). These proinflammatory factors are widely involved in synovitis, angiogenesis, and cartilage and bone destruction. TNF-α is one of the central proinflammatory cytokines in RA (Clark, 2007). Recent studies have shown that synoviocytes retain pathogenic features in the absence of stimulation and they could secrete cytokines and MMPs in culture (Bartok and Firestein, 2010; Bottini and Firestein, 2013). Therefore, we treated RA-FLSs with PTX alone or together with TNF-α, and detected the relative cytokines and MMPs.

We found that PTX decreased the expression of IL-6, IL-8, and inhibited TNF-α-induced production of *IL-1β, IL-8, MMP8,* and *MMP9*; moreover, PTX upregulated the expression of *OPG*, while down-regulating *RANKL* expression, which suggests that it may alleviate not only synovitis but also cartilage destruction. PTX reduced TNF-α-induced *IL-1β* mRNA expression significantly, whereas it had no effect on *IL-1β* mRNA expression. We speculate that PTX modulates the expression of *IL-1β* after the activation of TNF-α. We also tried to detect inflammation cytokine in RA-FLS supernatant cultured *in vitro* by ELISA, but found negative result. It is speculated that the inflammation cytokine in supernatant was below the detection limitation, which need to be further research.

There are several types of cell death, including apoptosis, necrosis, and autophagy. Apoptosis is widely accepted as a process of programmed cell death. Studies have shown that PTX and its agent can induce apoptosis of tumor cells, such as giant cells, sarcoma cells, lung cancer cells, breast cancer cells, and liver cancer cells (Ren et al., 2018; Xiao et al., 2019; Li et al., 2020; Xiaomeng et al., 2020). Therefore, we investigated whether PTX induces FLS apoptosis. We considered that upon treatment of cells with low concentrations of PTX, no significant differences would be observed, despite the occurrence of apoptosis; therefore, we used 100 and 200 nM PTX to treat cells. However, even high concentrations of PTX did not promote cell apoptosis. Therefore, we hypothesized that PTX causes cell death via other mechanisms.

To clarify the pharmacological mechanism of PTX, we investigated protein expression in related signaling pathways. MAPKs are key regulators of cell growth and survival in physiological and pathological processes. The downstream MAPK pathway is mainly composed of p38 MAPK, ERK1/2, and JNK (Sun Y. et al., 2015). The AKT/mTOR pathway also plays a dominant role in RA. AKT can phosphorylate the mTOR complex 1, which in turn promotes the activation of the downstream proteins, 4EBP1, P70S6K, and HIF-1α (Laragione and Gulko, 2010). Many studies have demonstrated that TNF-α can activate the MAPK and AKT/mTOR pathways (Görtz et al., 2005). In this study, PTX was found to downregulate the phosphorylation of TNF-α induced ERK1/2 and JNK; however, p38 expression was not significantly changed. Although p38 MAPK is thought to be highly associated with RA inflammation, this study suggests that PTX may act not through p38 but by blocking the activation of ERK1/2 and JNK. The results also showed that PTX inhibited TNF-α induced expression of AKT, 4EBP1, P70S6K, and HIF-1α. Nevertheless, TNF-α did not promote mTOR phosphorylation and there was no significant change in mTOR after PTX intervention. We hypothesized that the failure of TNF-α to promote mTOR activation is related to the cellular state. Since AKT could phosphorylates mTOR indirectly, we hypothesized that PTX may decrease the expression of 4EBP1, P70S6K, and HIF-1α by conducting AKT to other proteins rather than mTOR.

Interestingly, the present study found that PTX decreased the expression of HIF-1α in RA-FLS, which is consistent with our previous findings (Xu et al., 2019). As we known, HIF-1α is closely related to hypoxia-induced inflammation. In this study, we also found that TNF-α promoted the expression of HIF-1α, which may be related to hypoxia aggravated by the inflammatory environment.

Other studies have reported that PTX inhibits canine mammary gland tumor cells by inhibiting the AKT and MAPK signaling pathways (Ren et al., 2018). Other reports have shown that PTX inhibits the phosphorylation of ERK1/2, p38, and AKT in breast cancer cells, which is consistent with our results (Li et al., 2020). These results indicate that PTX may target molecules of the PI3K and MAPK pathways to modulate cell function.

Various animal models are available for studying RA, including TNF-α-transgenic mice, K/BxN mice, collagen-induced arthritis (CIA), and antigen-induced arthritis (AIA). Among these, CIA are the most widely used. Studies have shown that, compared with C57BL mice, a higher CIA induction success rate is obtained when using DBA/1 mice (Pietrosimone et al., 2015). A dose of 1.5 mg/kg PTX was used based on our previous research (Xu et al., 2019). Our results suggest that PTX can not only inhibit synovitis, cartilage and bone destruction, but also relieve inflammation of the liver and spleen. Taken together with the *in vitro* results, it was speculated that PTX alleviated synovitis might be related to the inhibition of cell migration and the expression of IL-1β, IL-6, and IL-8. The mechanism by which PTX alleviates cartilage and bone destruction may own to the reduction of MMPs expression.

Of note, inflammation of the synovium, liver, and spleen occurred in parallel, suggesting that PTX can relieve synovitis and systemic immune response. Although the data indicate that the spleen index of the PTX group decreased, there was no significant difference in the liver index among the three groups. However, our previous research proved that the index of liver and spleen increased remarkably in C57BL/6 mice with antigen-induced arthritis and decreased after treatment (Du et al., 2020). Therefore, we speculate that it may be related to the species of mice and the method used to induce arthritis. On the other hand, liver index presents limitations as an indicator to evaluate inflammation, while the pathological evaluation of liver and spleen is more convincing.

In conclusion, PTX can inhibit the migration of RA-FLS and the expression of inflammatory factors, which may be related to the inhibition of the MAPK and AKT/mTOR signaling pathways. We further confirmed the relief effect of PTX on RA *in vivo*. In addition to basic experimental research, PTX was also conducted in a phase 2 clinical study in RA patients, although the results were not published (Clinicaltrials, 2008). A case report suggested that PTX may be useful in patients with RA (Yoo and Baek, 2000). In addition, PTX is being studied in patients with other autoimmune inflammatory diseases. For example, a completed clinical trial shown that micellar PTX has therapeutic activity in patients with severe psoriasis (Ehrlich et al., 2004). Moreover, PTX seems promising in the prevention of renal and hepatic fibrosis, inflammation, and coronary artery restenosis (Zhang et al., 2014). Although the therapeutic potential of paclitaxel extends beyond cancer, further studies are required to determine its other therapeutic uses.

In summary, our study showed that PTX may be a new therapeutic option for RA treatment. Further studies should focus on the serological detection of inflammatory factors, and identify the specific target of PTX on synoviocytes.

## Data Availability

The raw data supporting the conclusions of this article will be made available by the authors, without undue reservation.
