# Supplementary figures and images for: Paclitaxel Inhibits Synoviocyte Migration and Inflammatory Mediator Production in Rheumatoid Arthritis (part 1 of 2)
Source: Front Pharmacol. 2021 Sep 9;12:714566. doi: 10.3389/fphar.2021.714566 (PMC8458635; doi:10.3389/fphar.2021.714566)

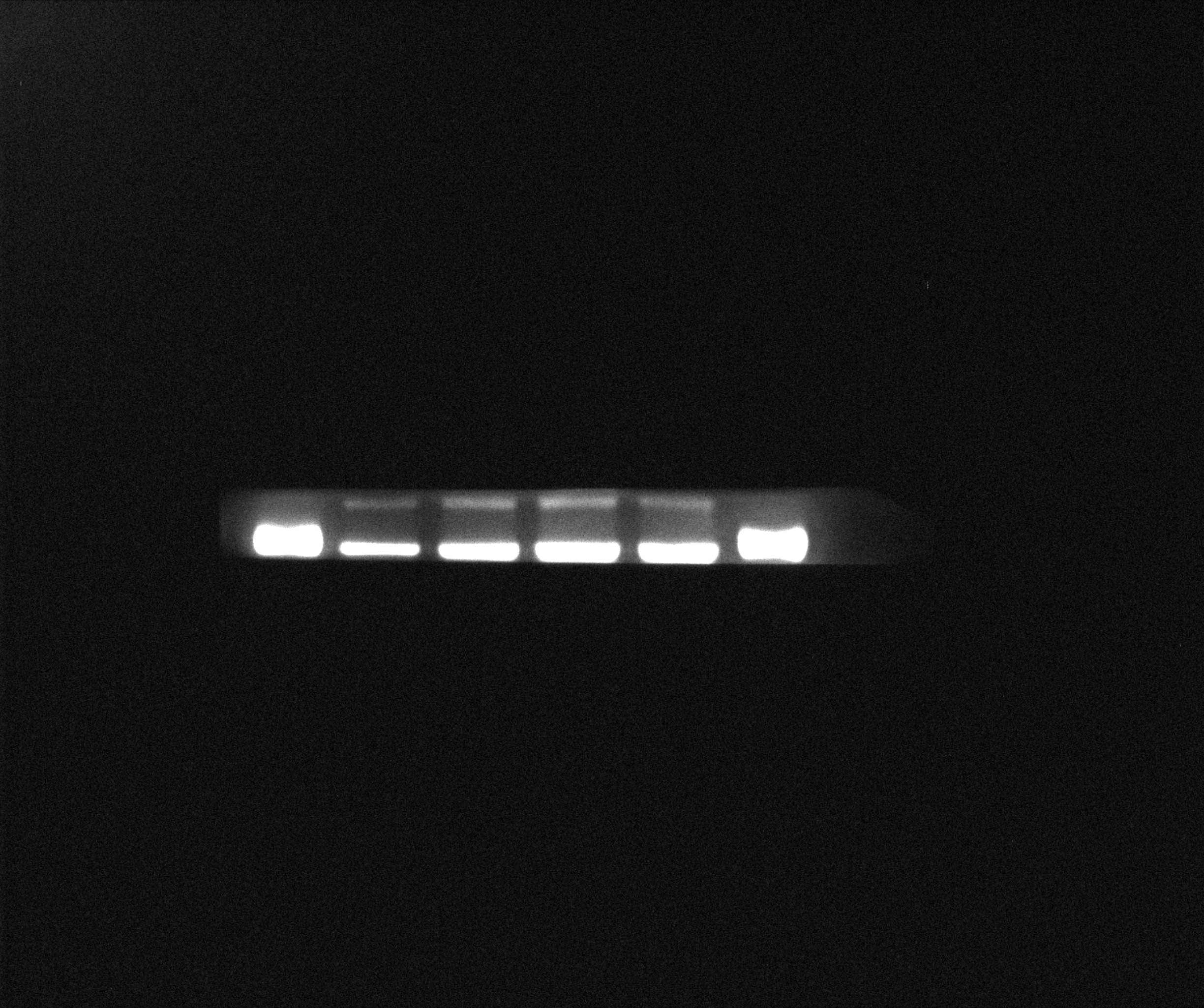

Supplement: Supplementary file 1 [file DataSheet3.ZIP › P70S6K/P70s6k-B-2.jpg]

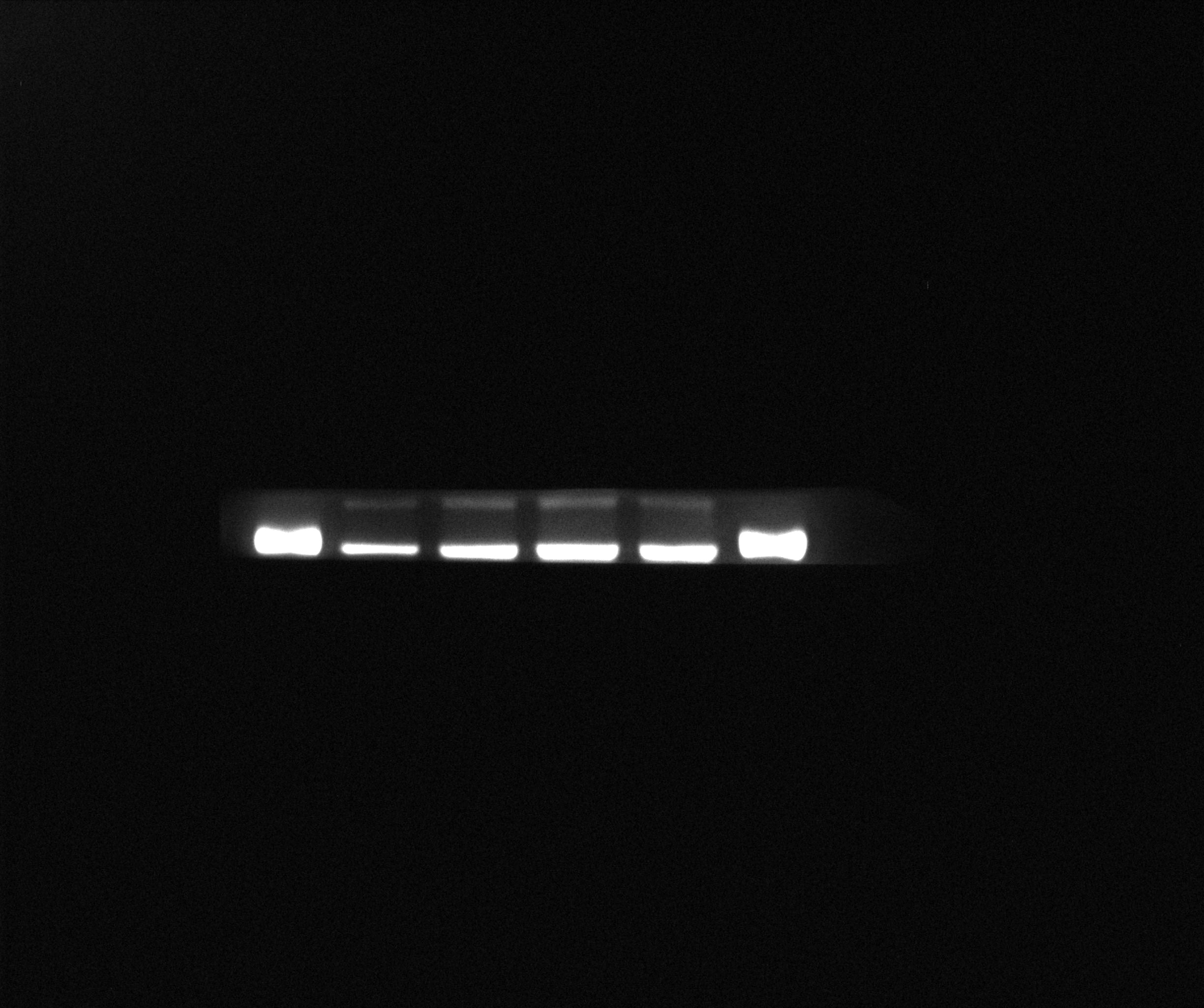

Supplement: Supplementary file 1 [file DataSheet3.ZIP › P70S6K/P70s6k-B-3.jpg]

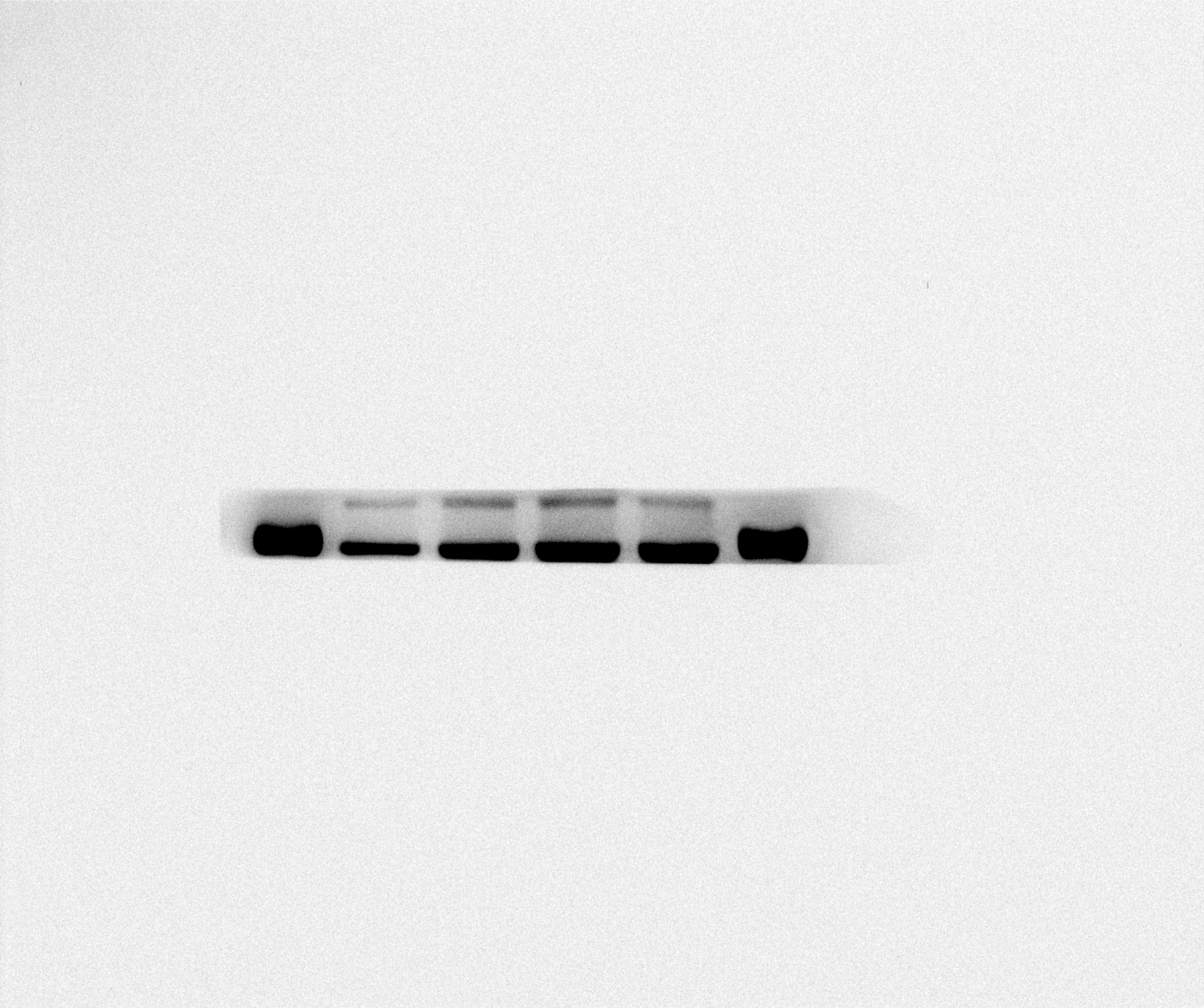

Supplement: Supplementary file 1 [file DataSheet3.ZIP › P70S6K/P70s6k-F-2.jpg]

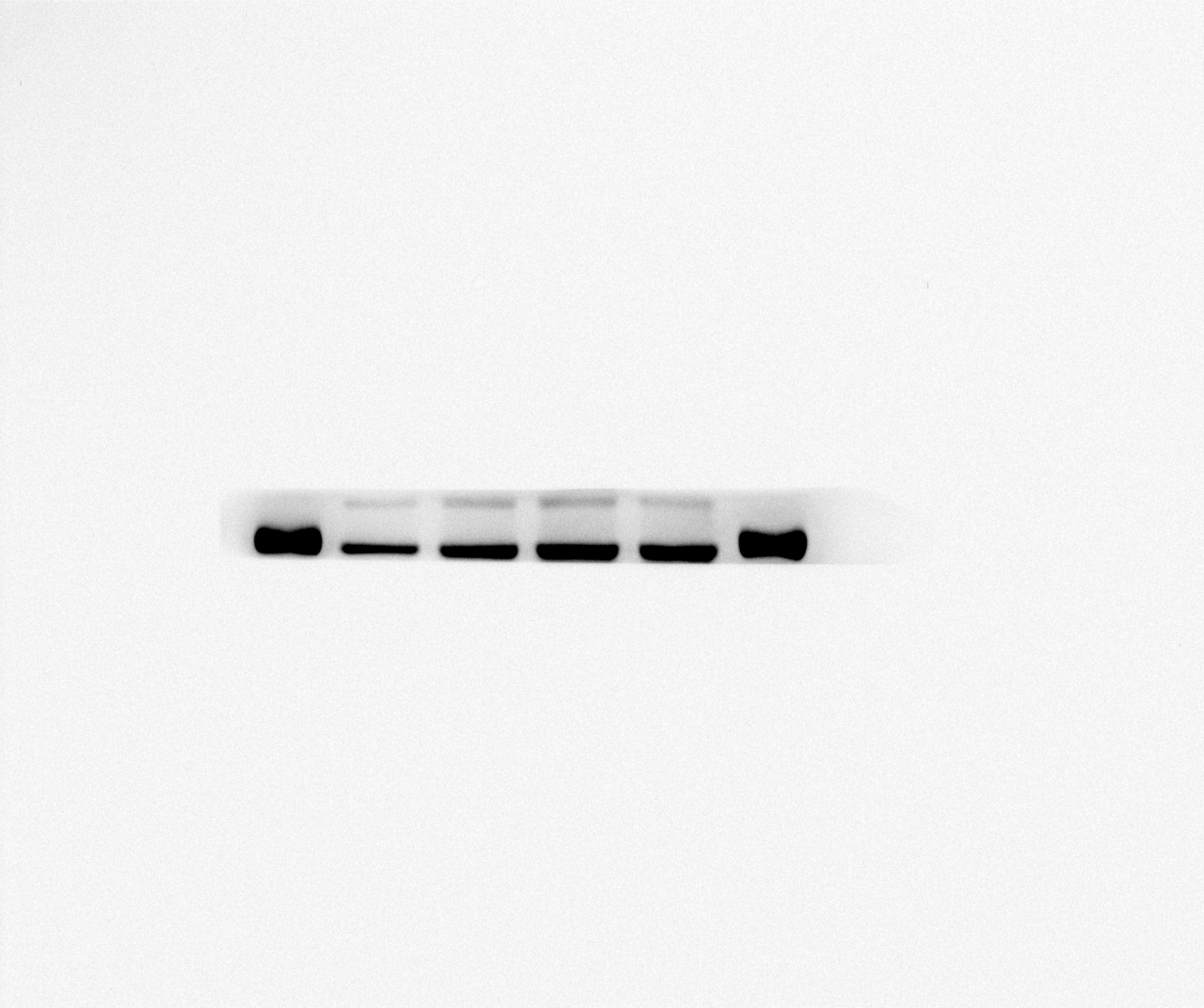

Supplement: Supplementary file 1 [file DataSheet3.ZIP › P70S6K/P70s6k-F-3.jpg]

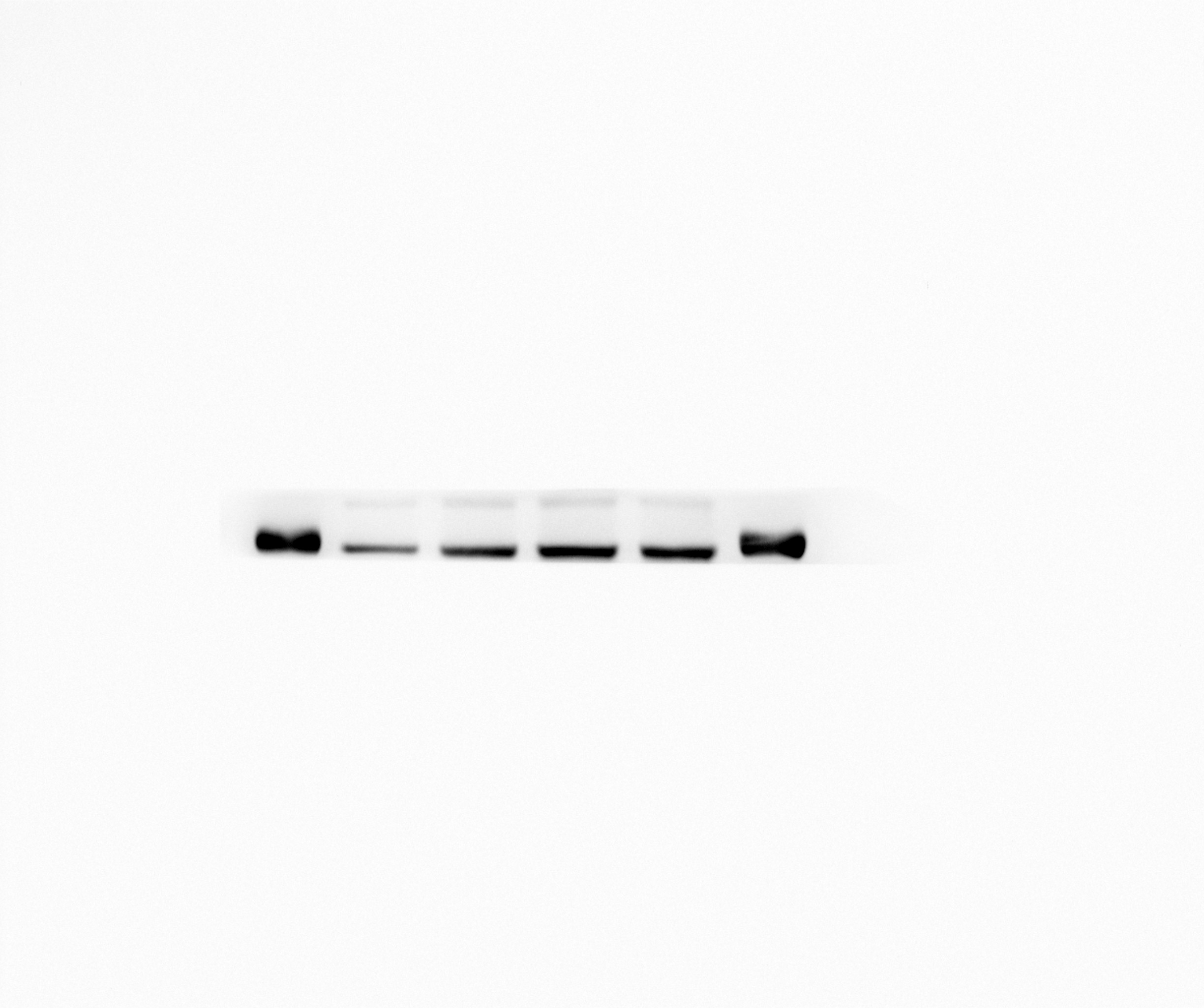

Supplement: Supplementary file 1 [file DataSheet3.ZIP › P70S6K/P70s6k.jpg]

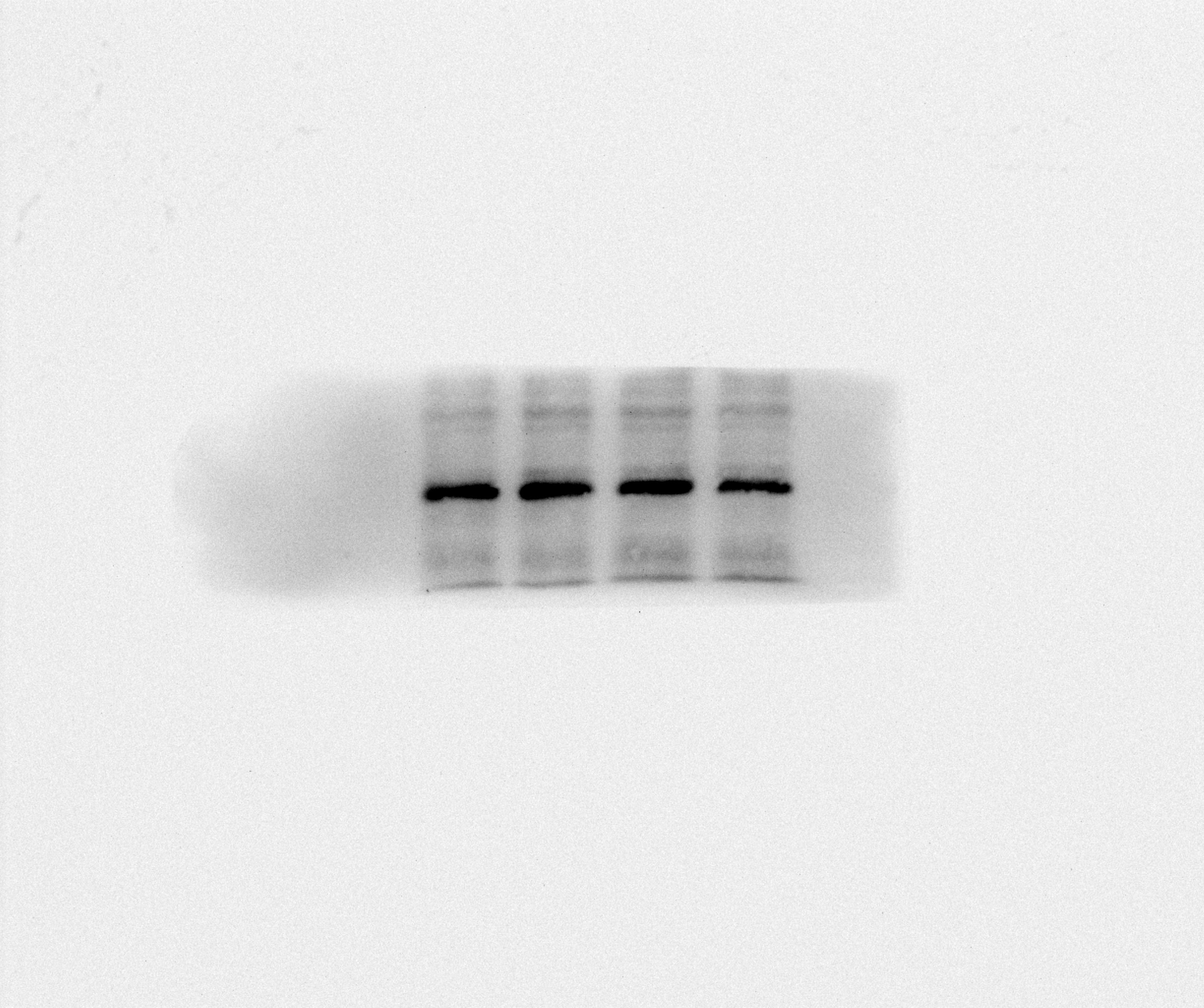

Supplement: Supplementary file 1 [file DataSheet3.ZIP › p-4EBP1/P-4EBP1-2-F-2.jpg]

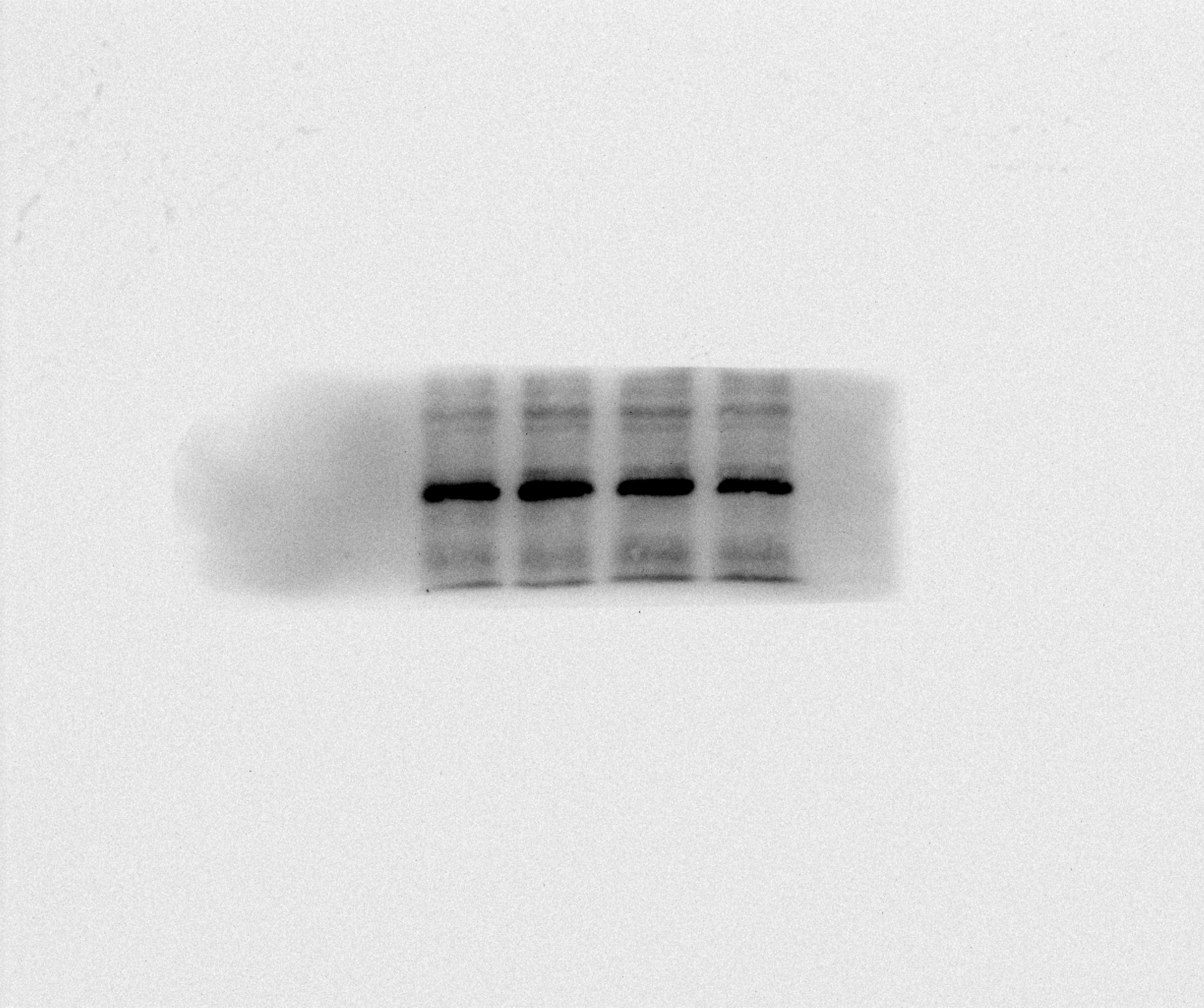

Supplement: Supplementary file 1 [file DataSheet3.ZIP › p-4EBP1/P-4EBP1-2-F.jpg]

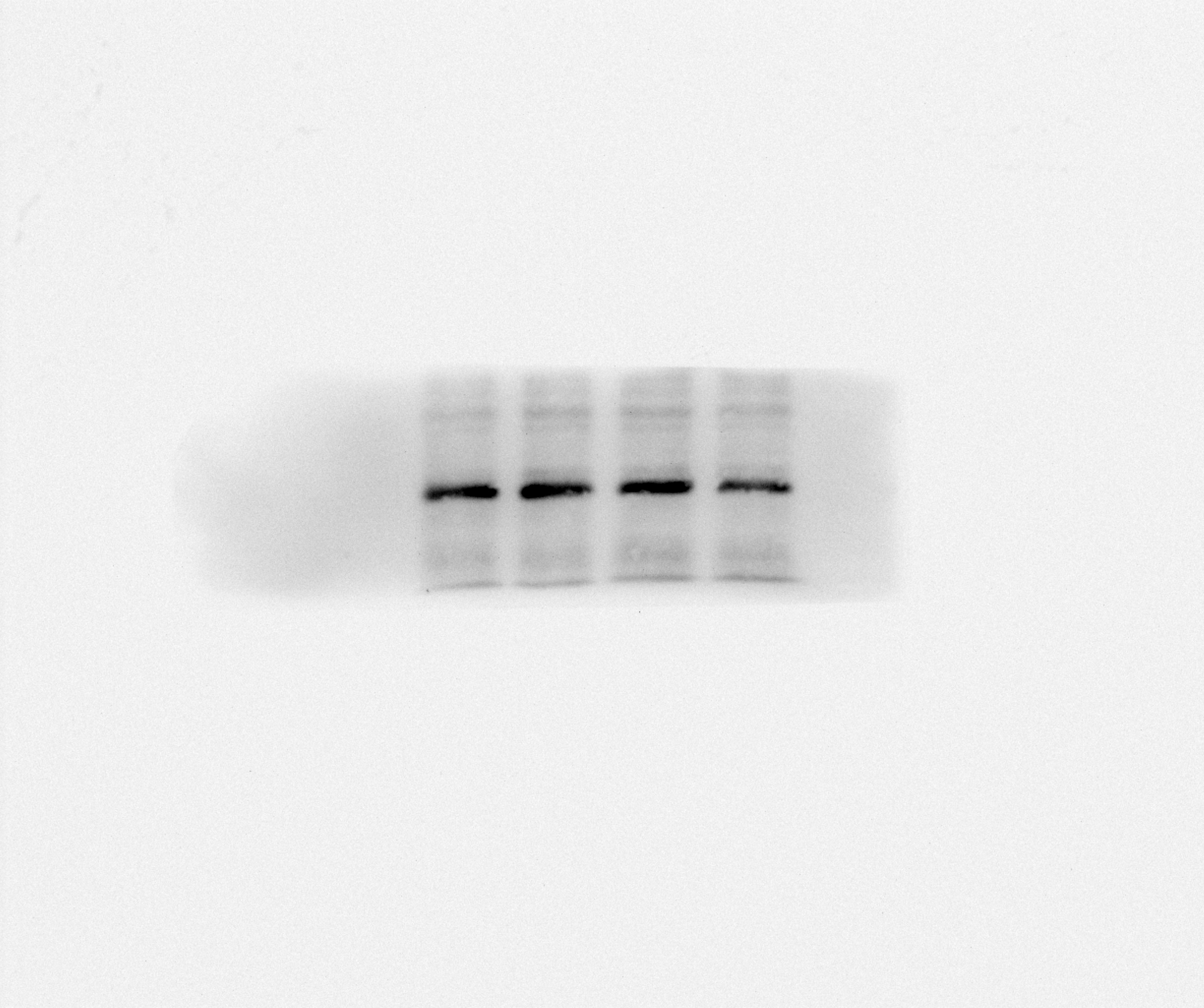

Supplement: Supplementary file 1 [file DataSheet3.ZIP › p-4EBP1/P-4EBP1-2.jpg]

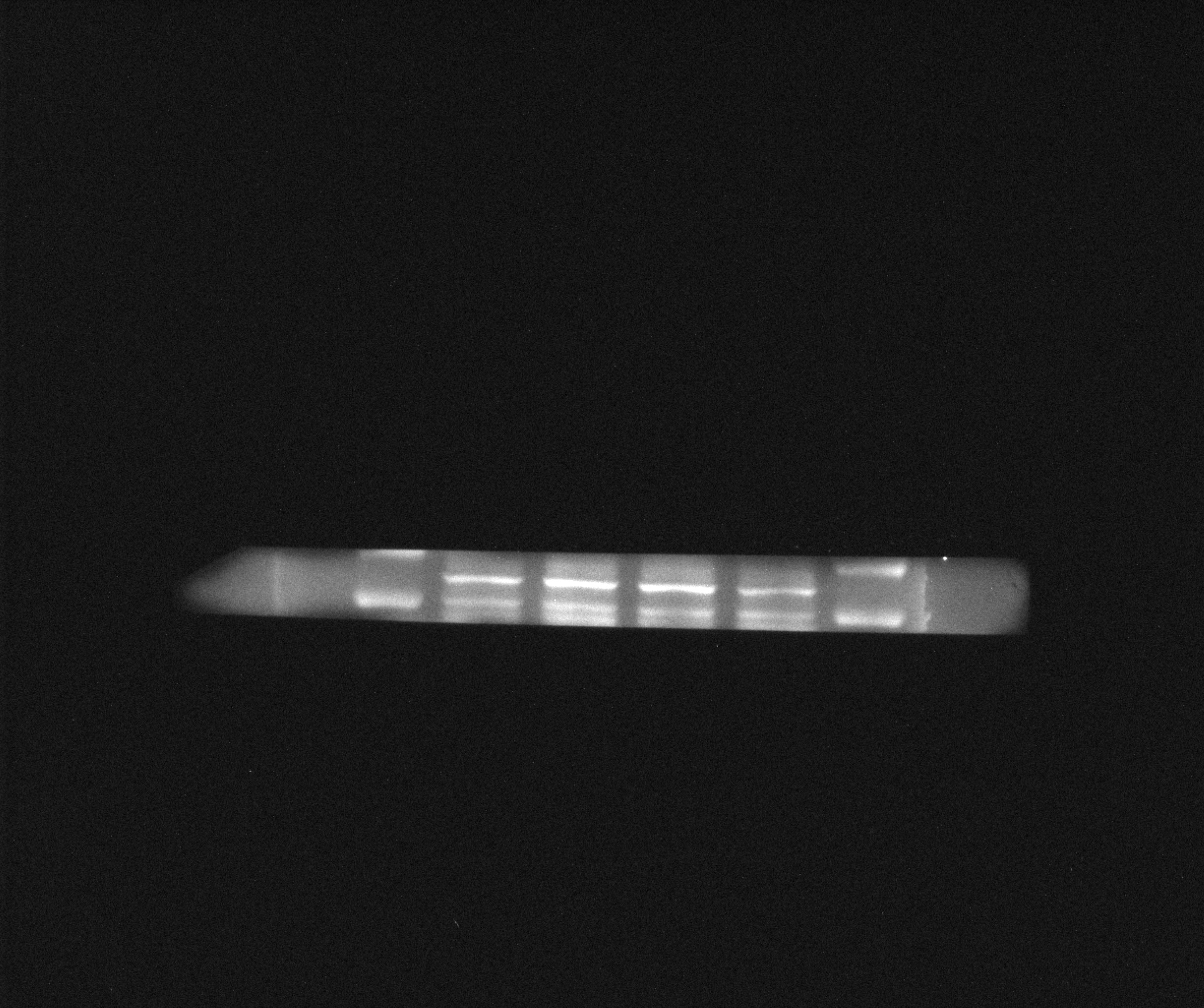

Supplement: Supplementary file 1 [file DataSheet3.ZIP › p-AKT/p-AKT-B-2.jpg]

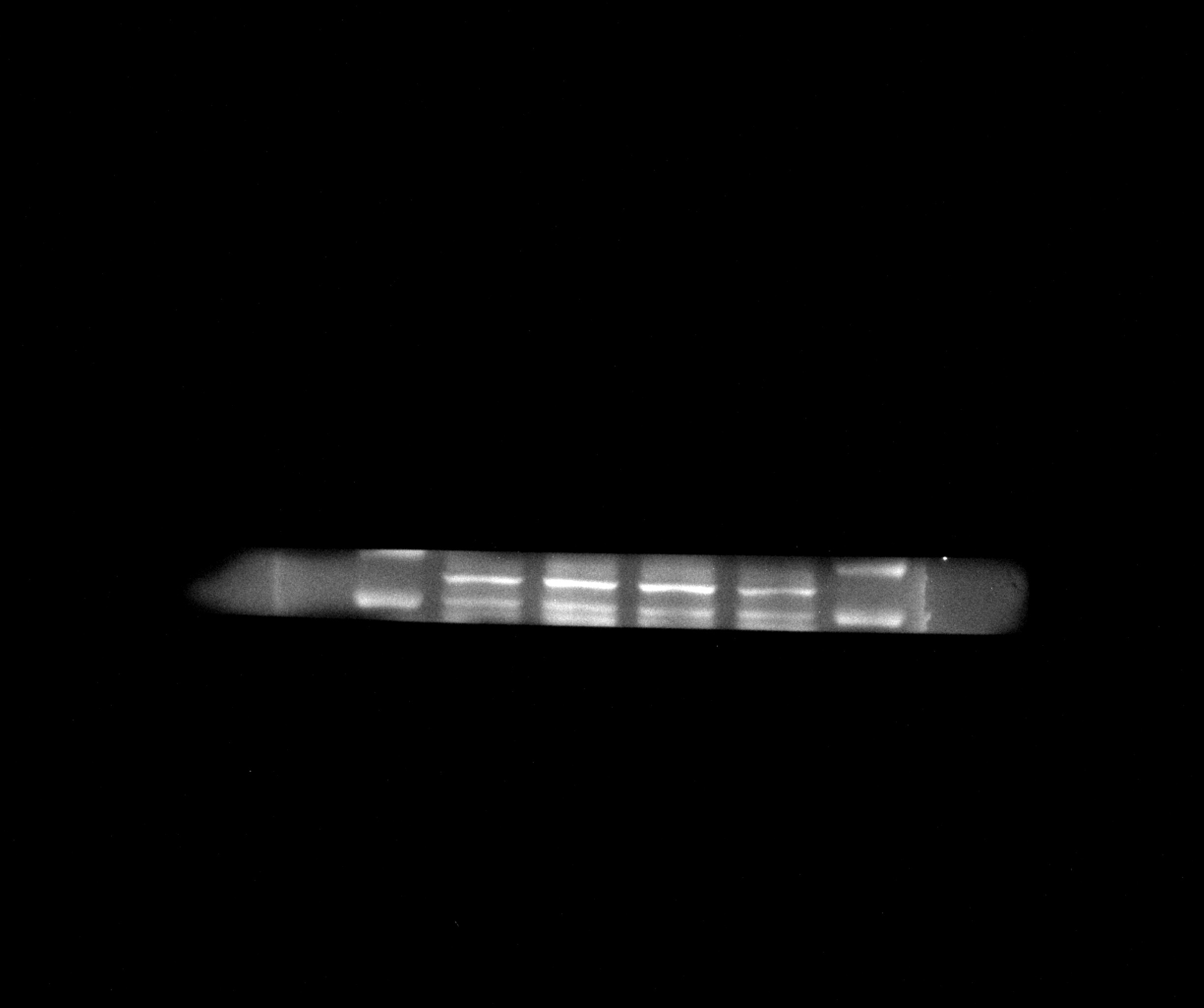

Supplement: Supplementary file 1 [file DataSheet3.ZIP › p-AKT/p-AKT-B.jpg]

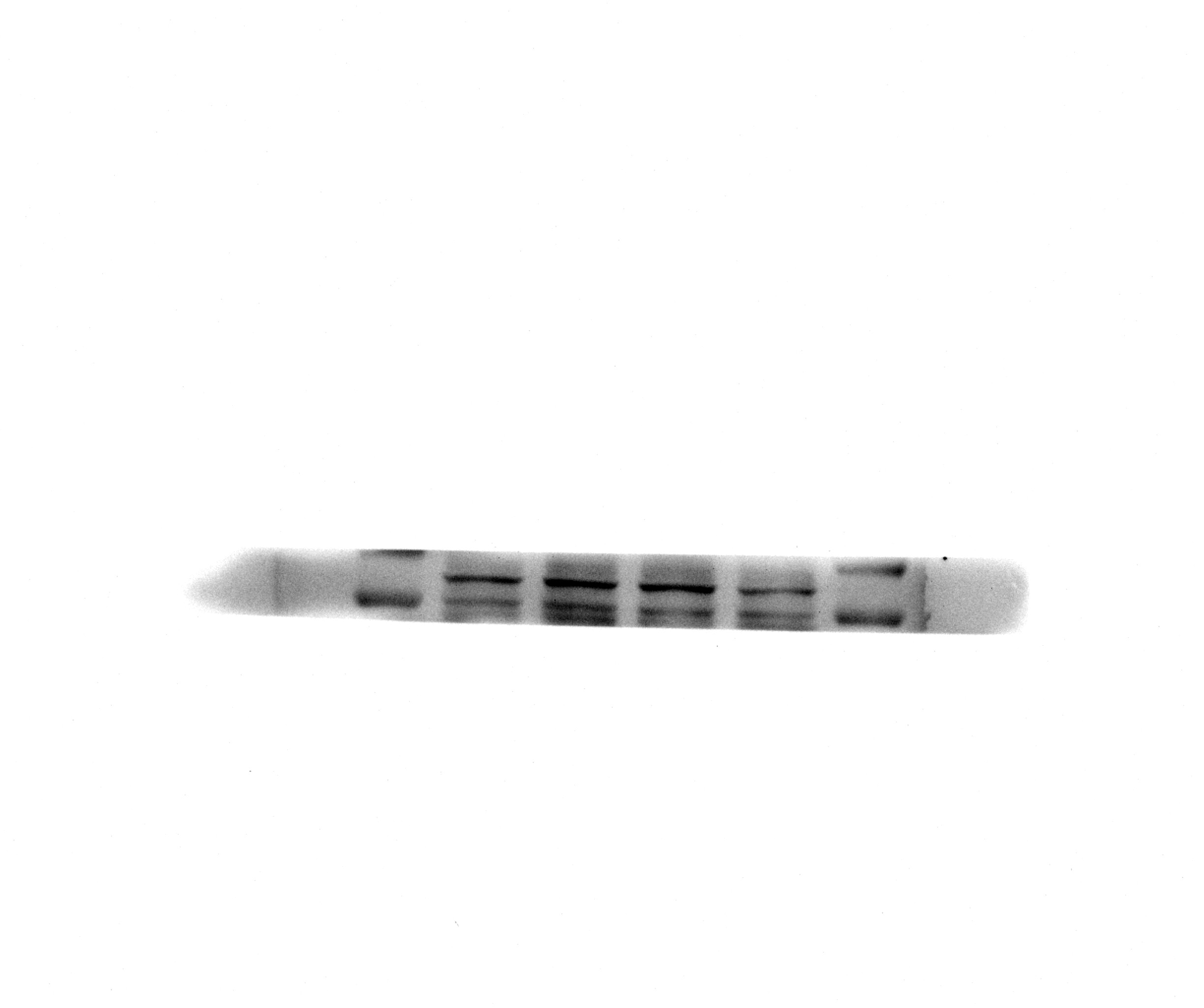

Supplement: Supplementary file 1 [file DataSheet3.ZIP › p-AKT/p-AKT-F-2.jpg]

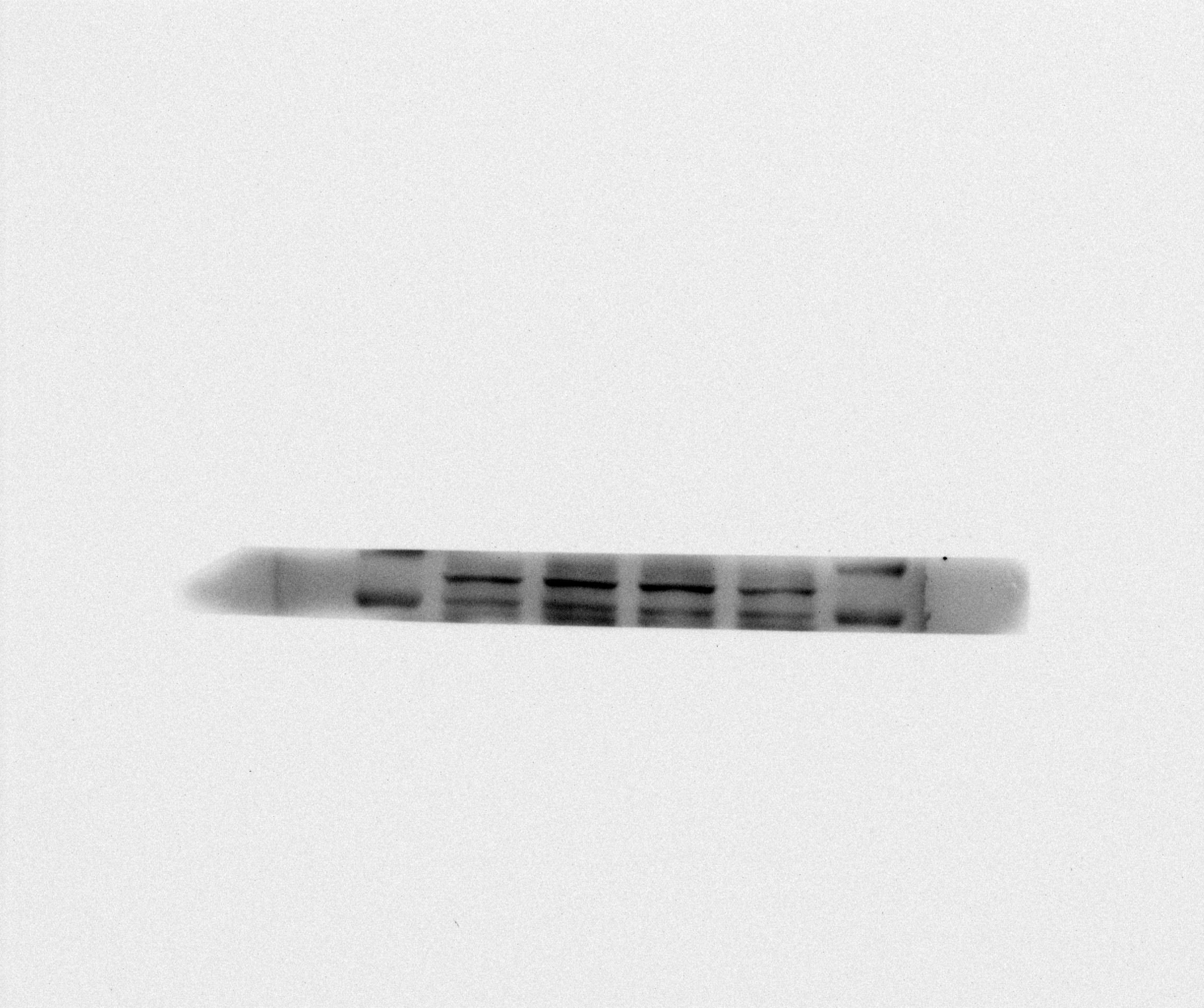

Supplement: Supplementary file 1 [file DataSheet3.ZIP › p-AKT/p-AKT-F.jpg]

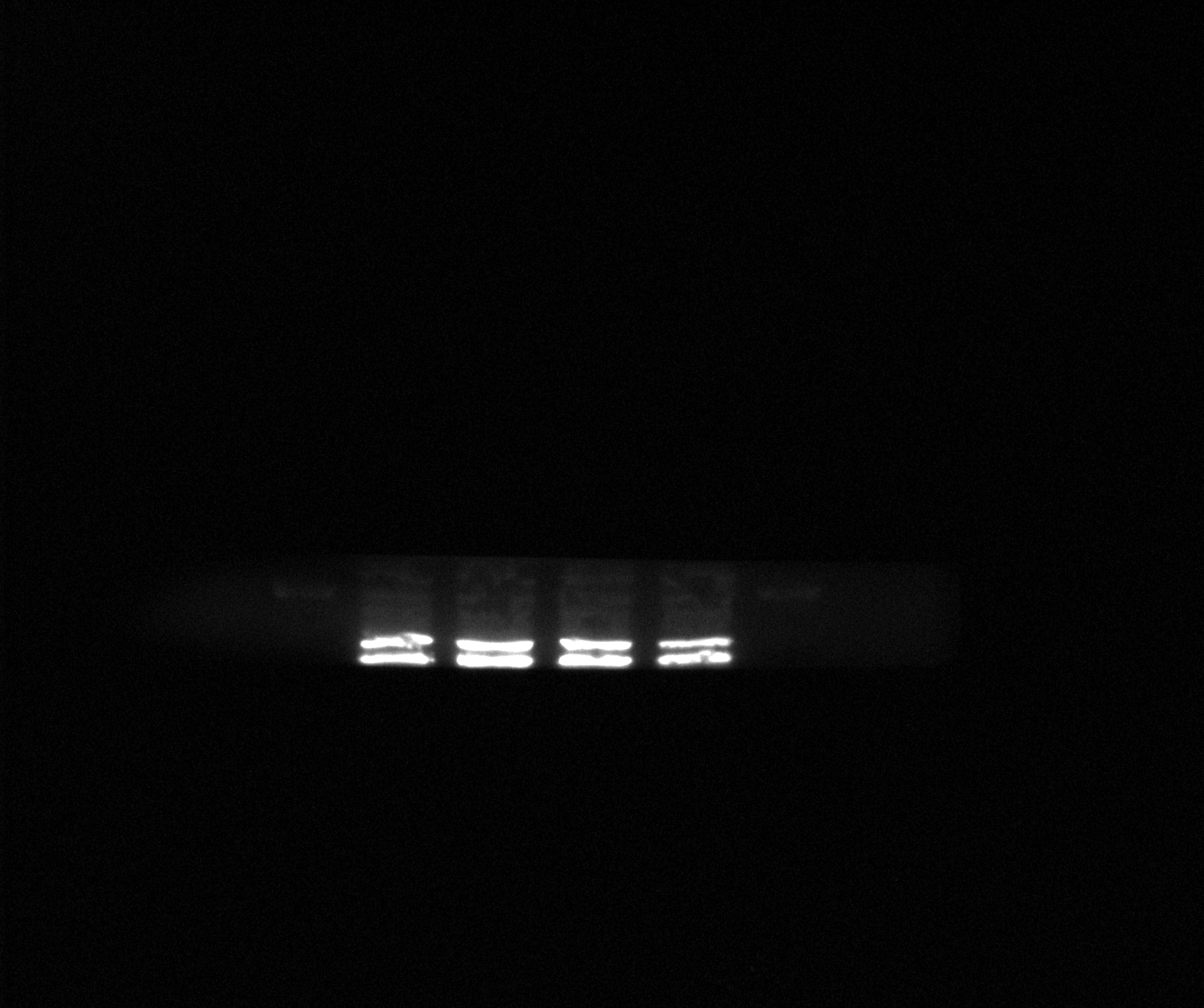

Supplement: Supplementary file 1 [file DataSheet3.ZIP › p-ERK/P-ERK-B-2.jpg]

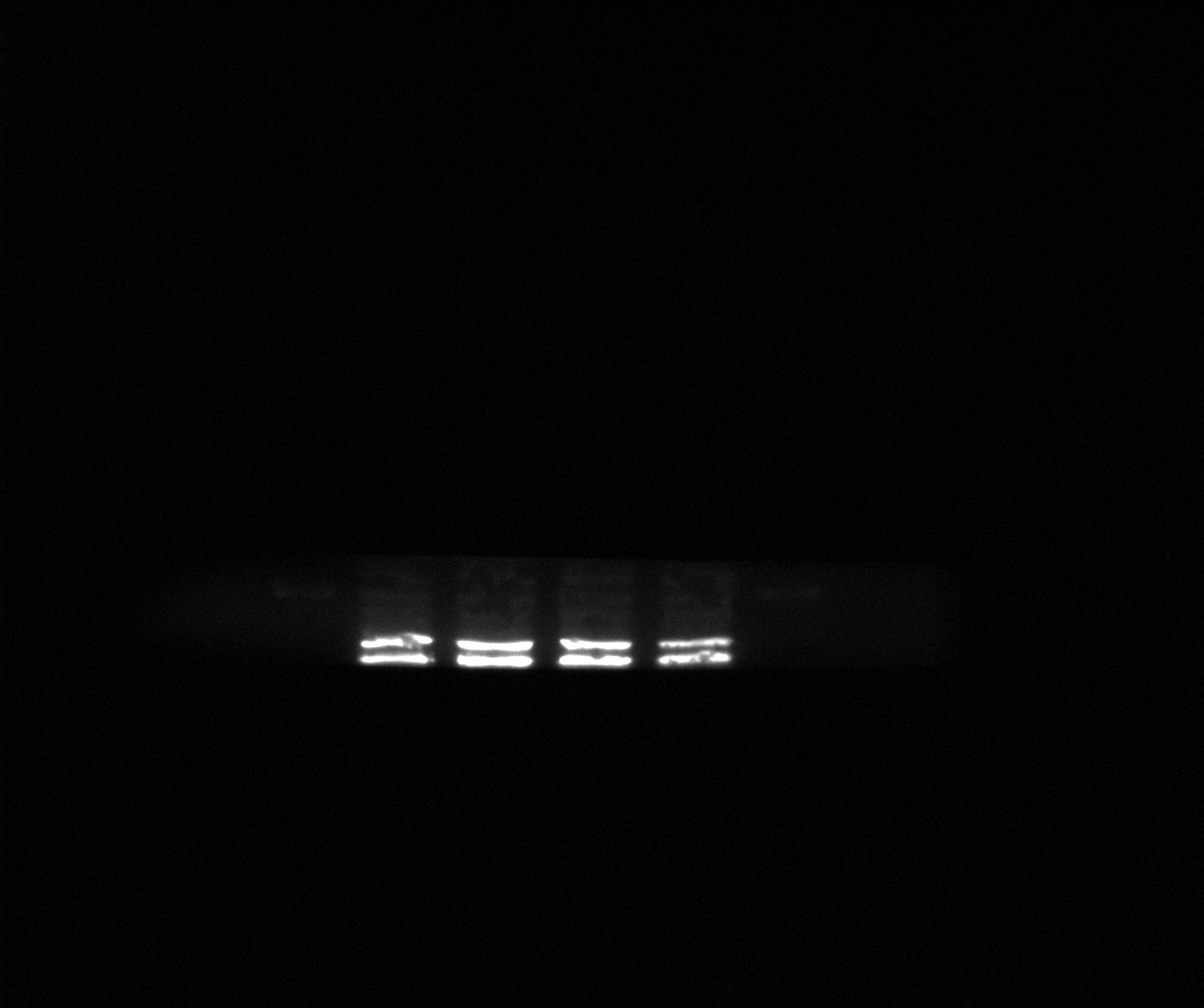

Supplement: Supplementary file 1 [file DataSheet3.ZIP › p-ERK/P-ERK-B.jpg]

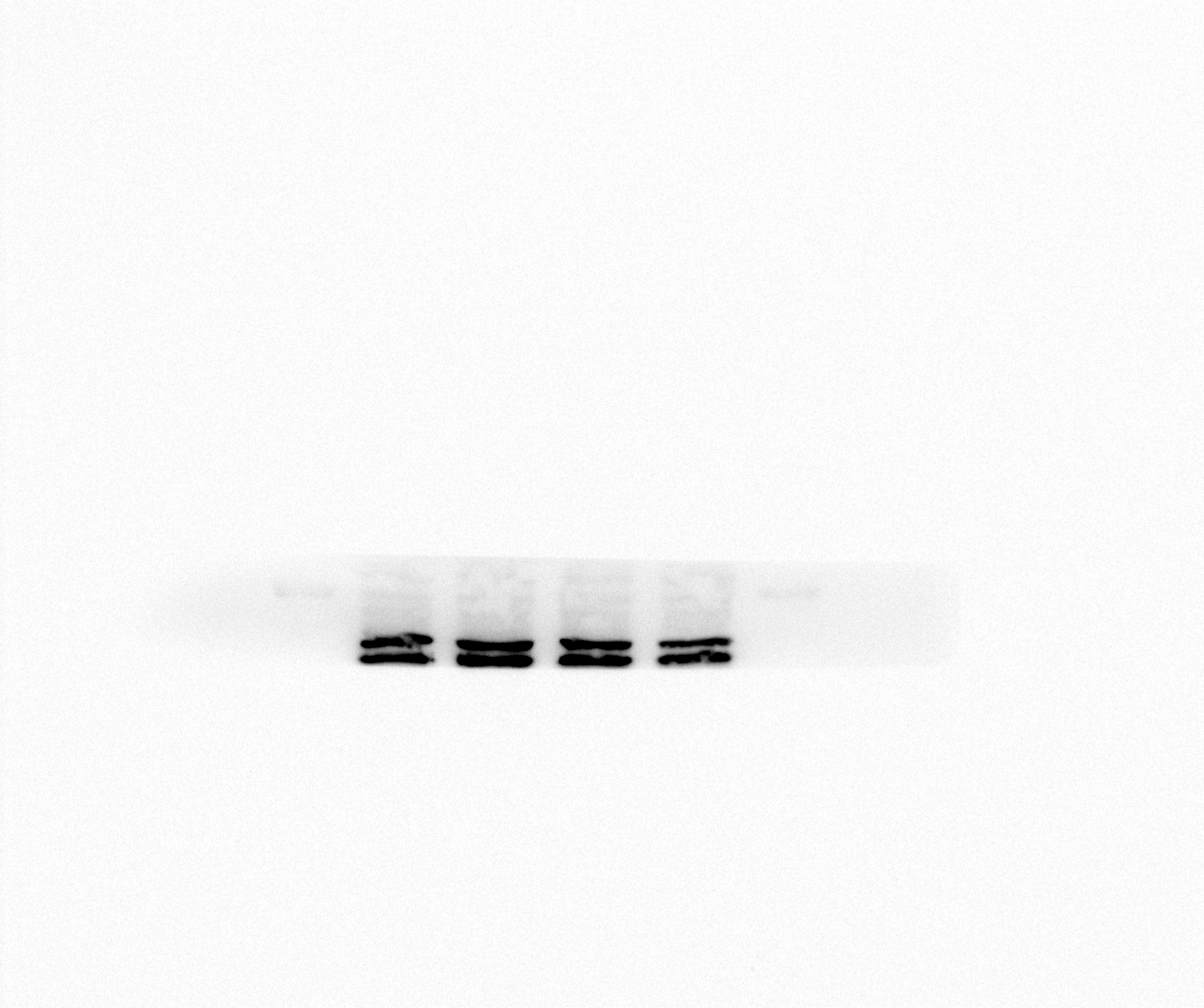

Supplement: Supplementary file 1 [file DataSheet3.ZIP › p-ERK/P-ERK-F-2.jpg]

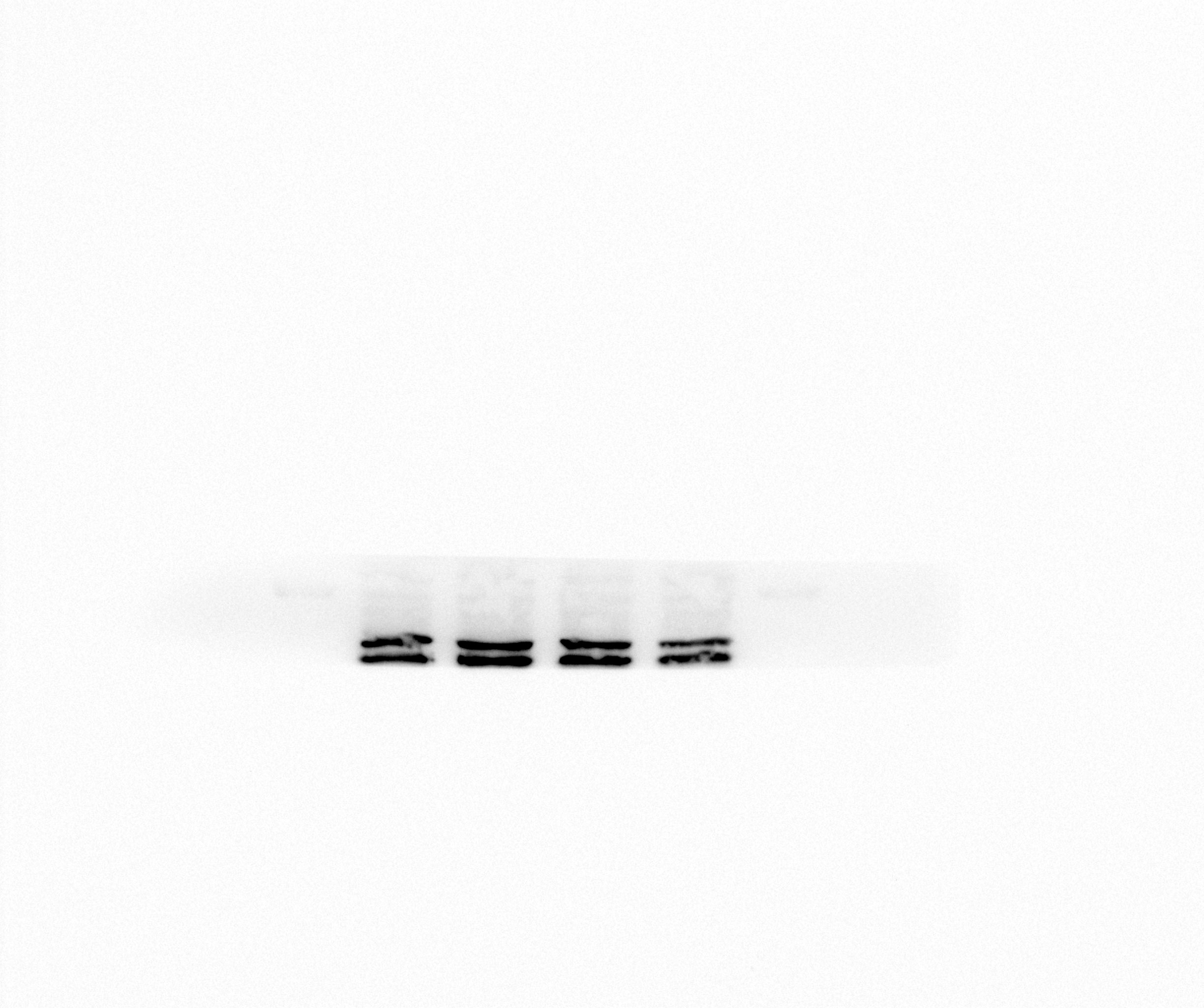

Supplement: Supplementary file 1 [file DataSheet3.ZIP › p-ERK/P-ERK-F.jpg]

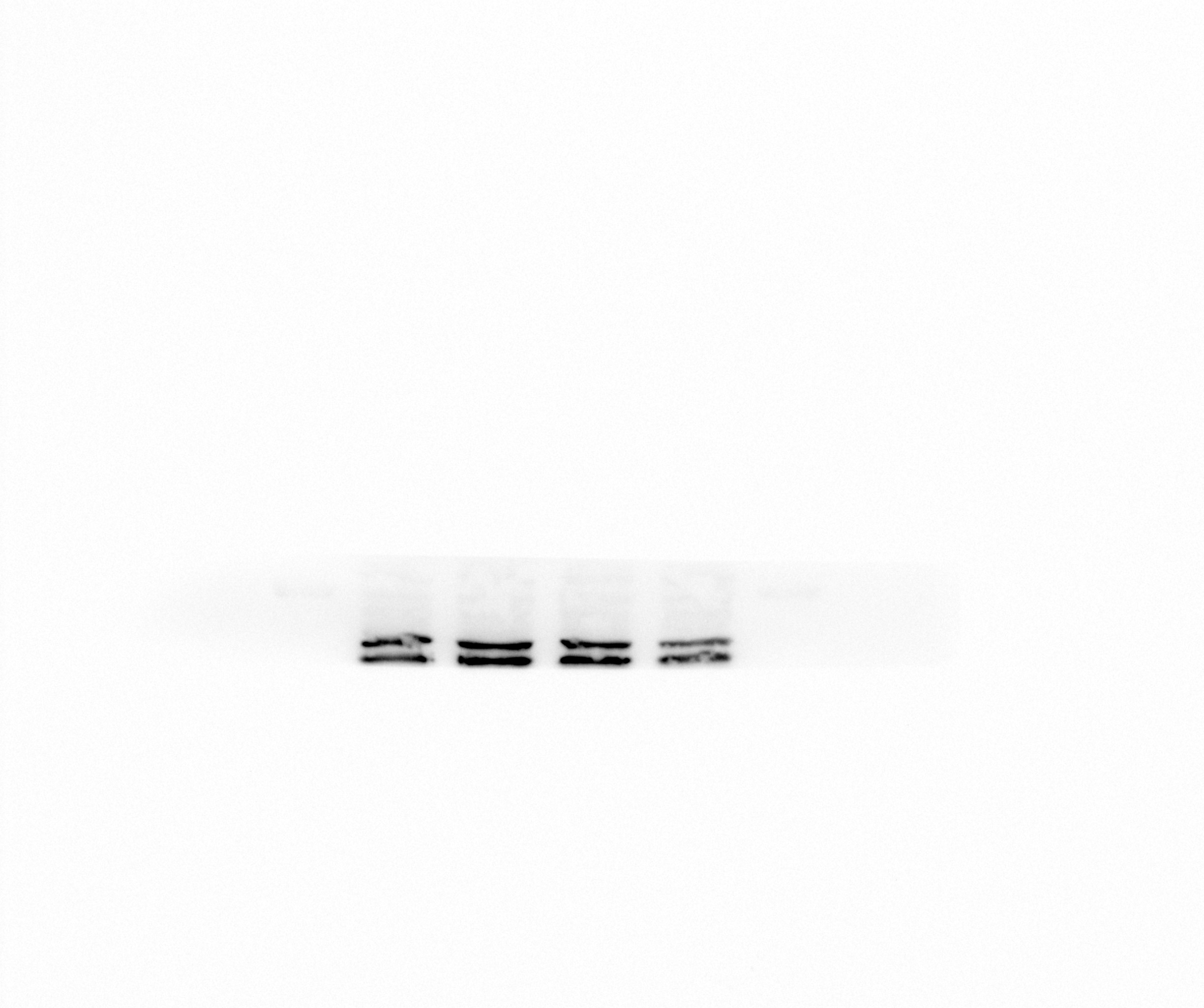

Supplement: Supplementary file 1 [file DataSheet3.ZIP › p-ERK/P-ERK.jpg]

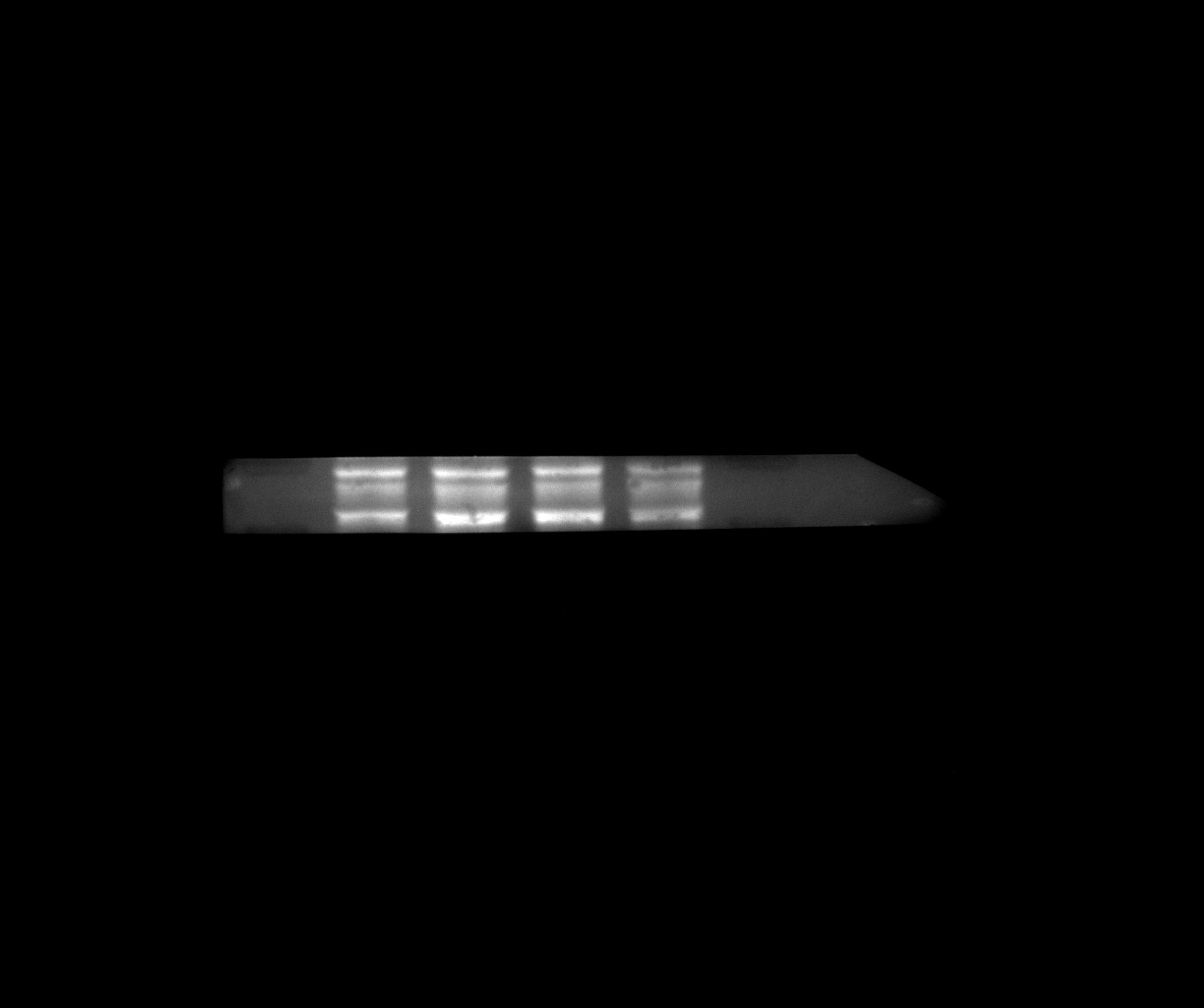

Supplement: Supplementary file 1 [file DataSheet3.ZIP › p-JNK/P-JNK-2-B-2.jpg]

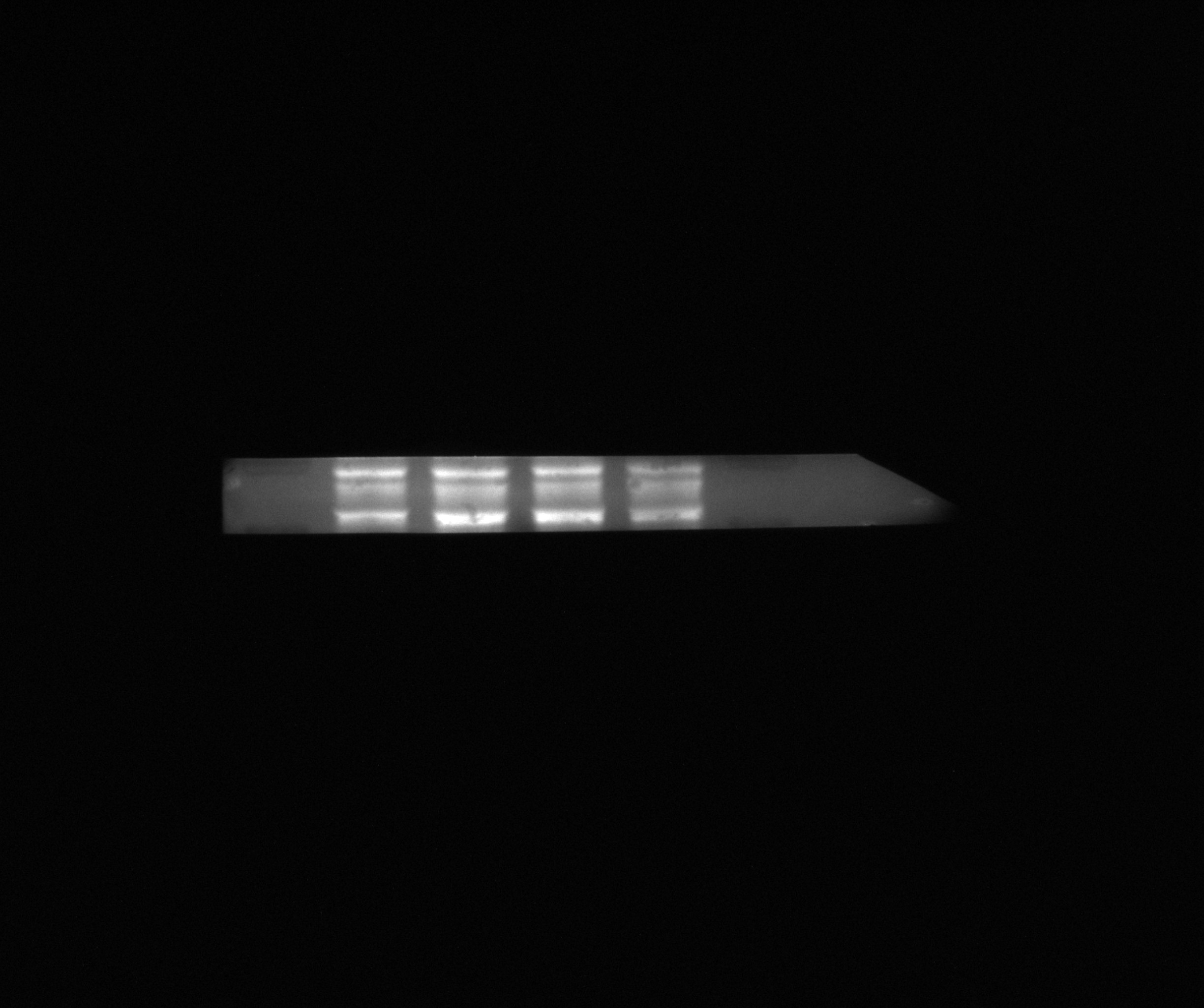

Supplement: Supplementary file 1 [file DataSheet3.ZIP › p-JNK/P-JNK-2-B.jpg]

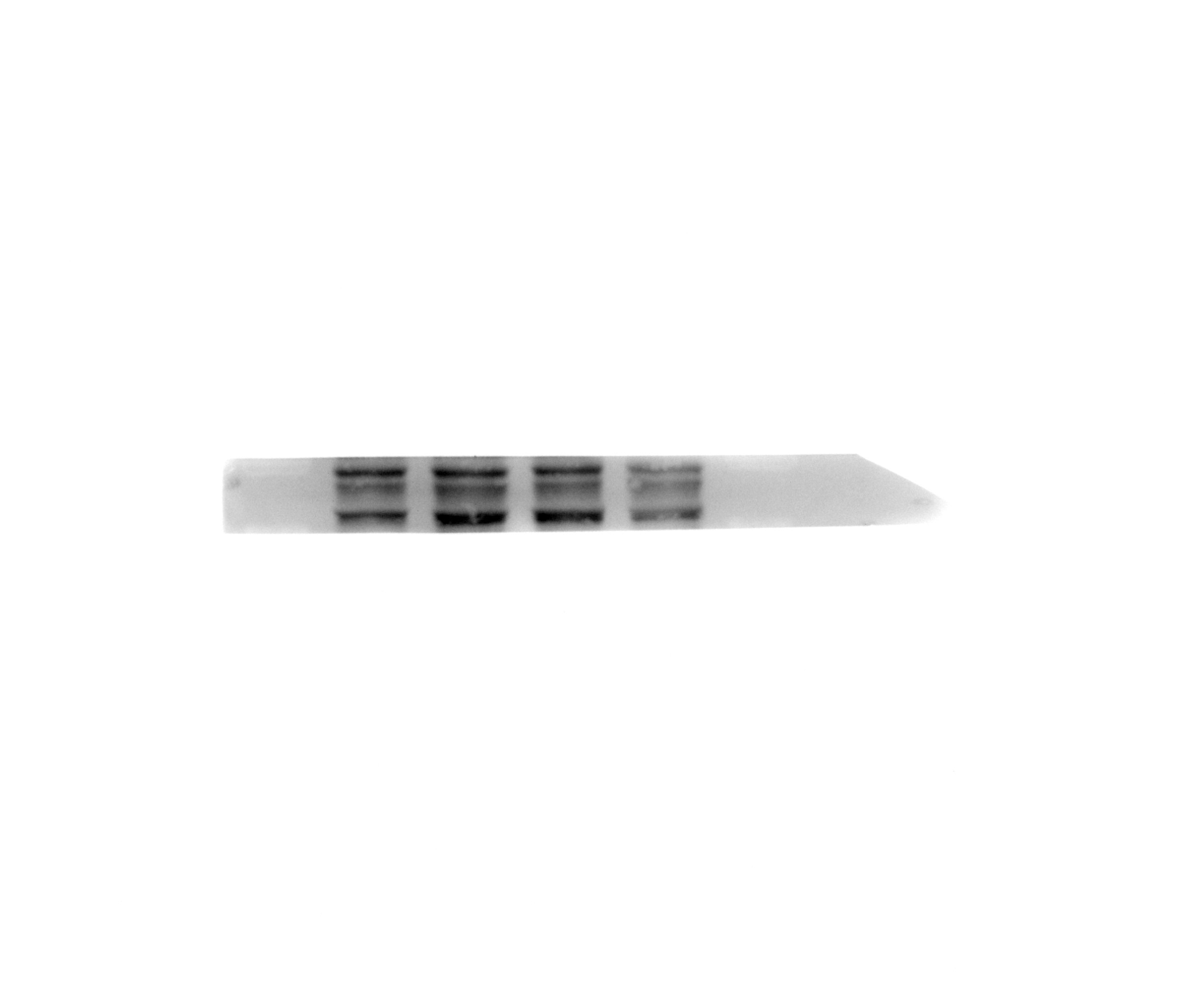

Supplement: Supplementary file 1 [file DataSheet3.ZIP › p-JNK/P-JNK-2-F-2.jpg]

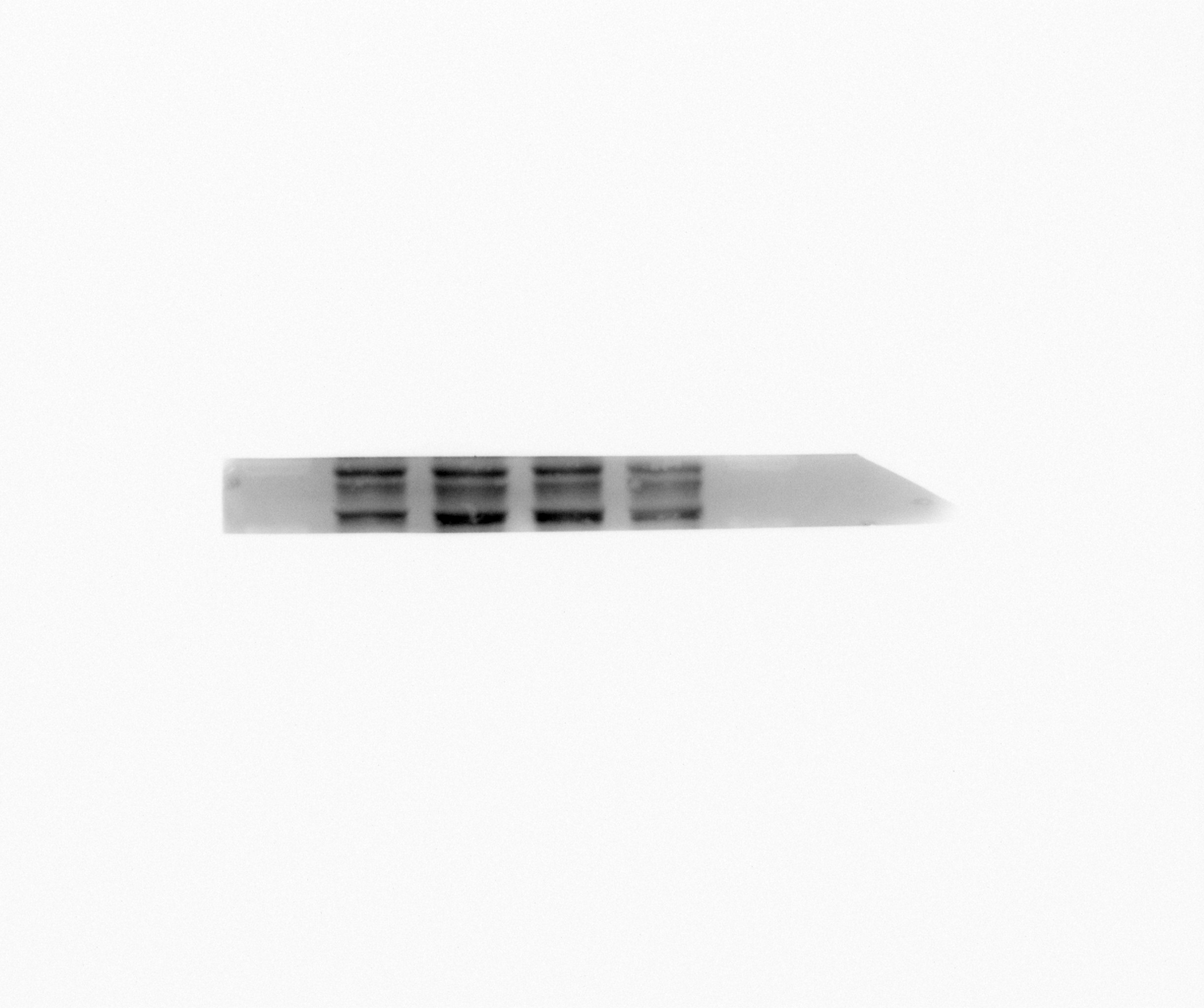

Supplement: Supplementary file 1 [file DataSheet3.ZIP › p-JNK/P-JNK-2-F.jpg]

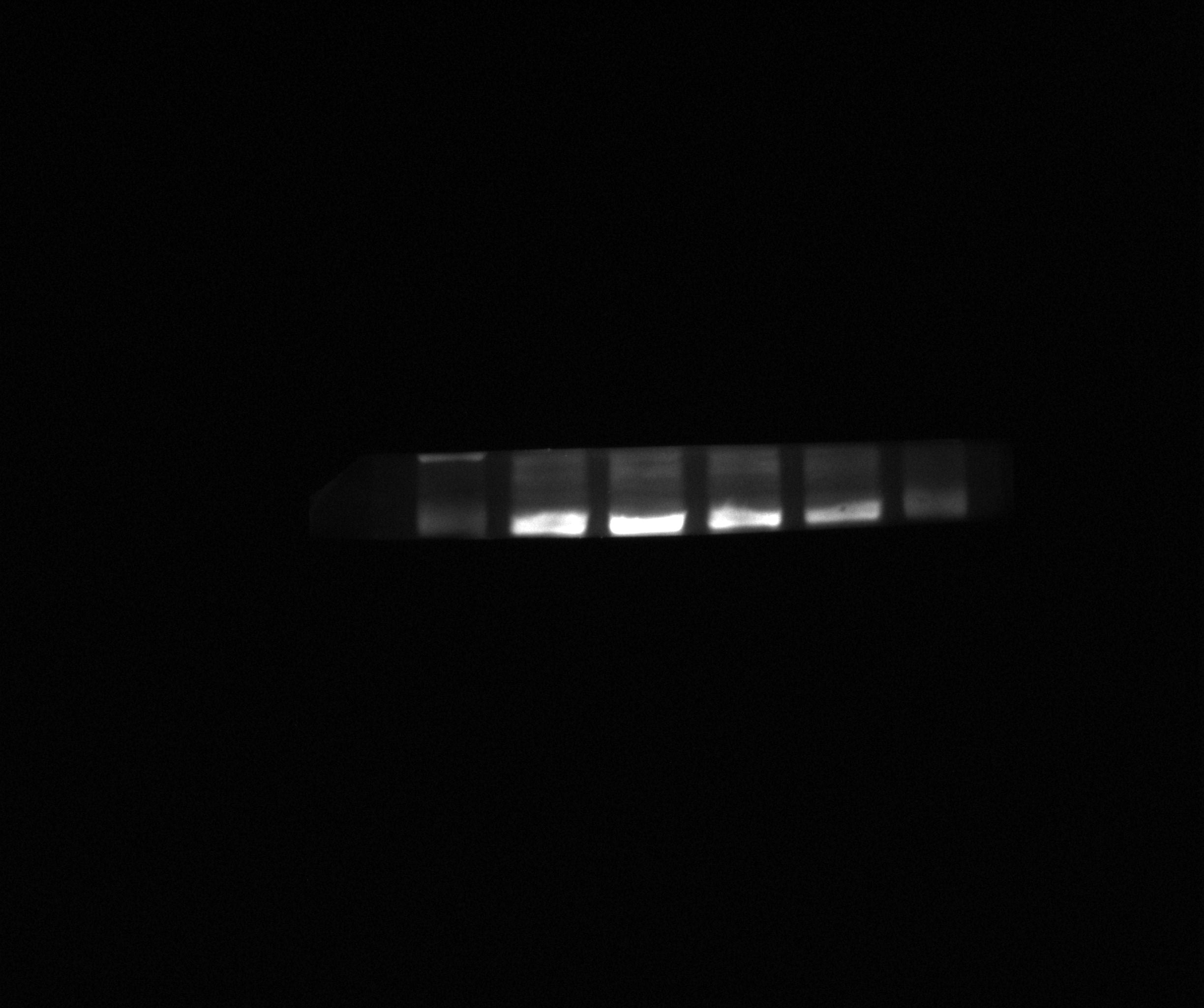

Supplement: Supplementary file 1 [file DataSheet3.ZIP › p-P70S6K/p-P70s6k-B.jpg]

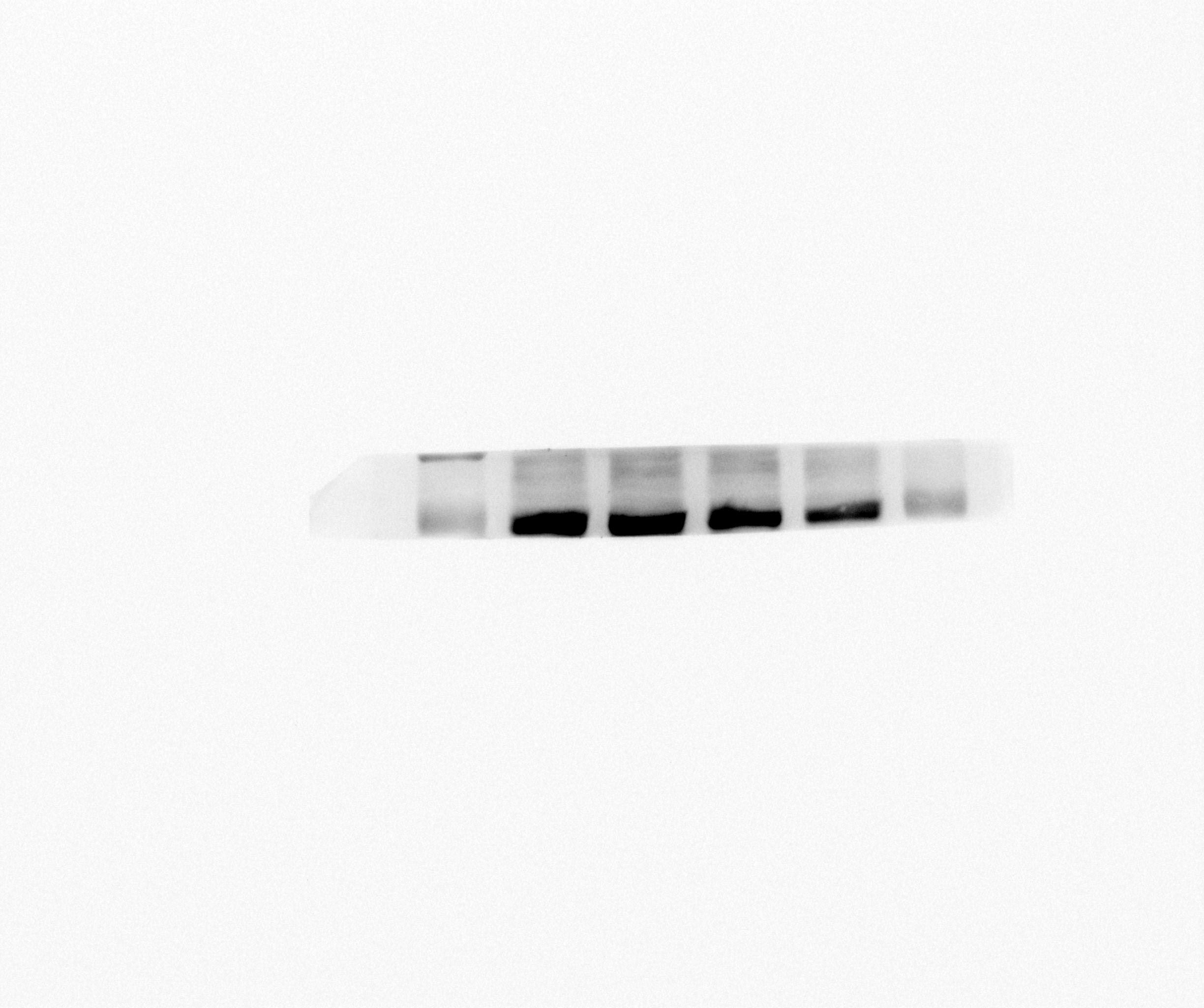

Supplement: Supplementary file 1 [file DataSheet3.ZIP › p-P70S6K/p-P70s6k-F-2.jpg]

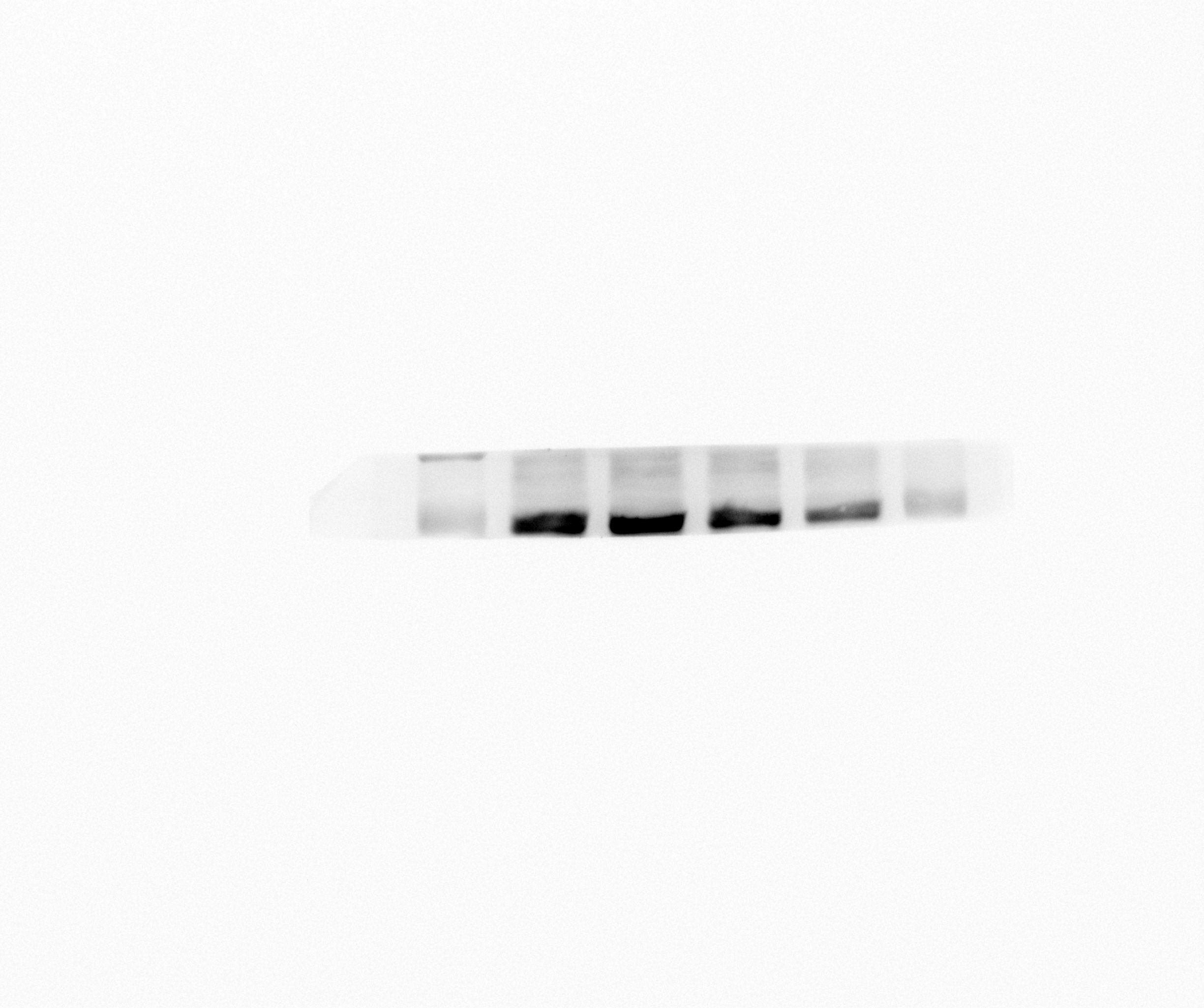

Supplement: Supplementary file 1 [file DataSheet3.ZIP › p-P70S6K/p-P70s6k-F.jpg]

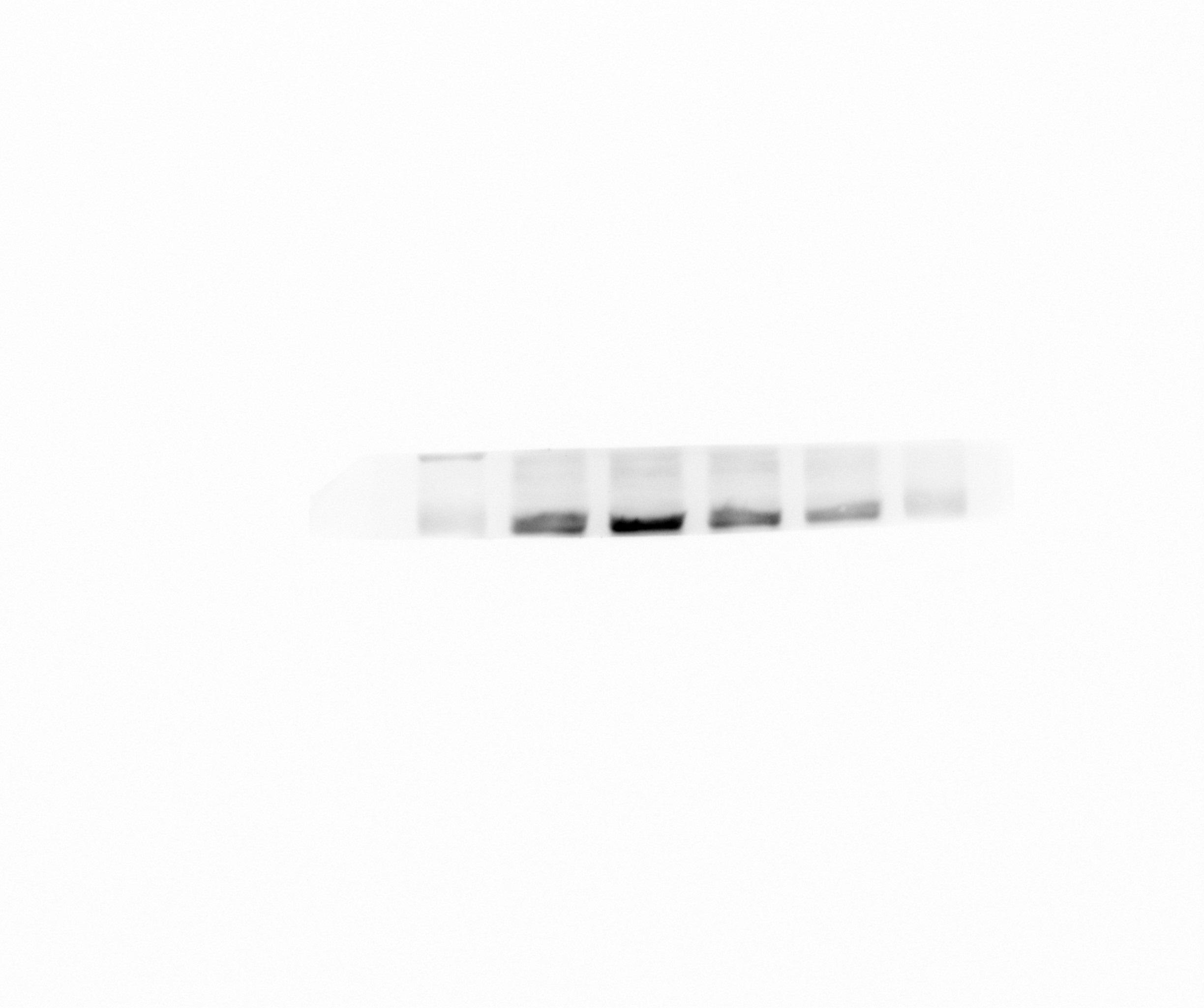

Supplement: Supplementary file 1 [file DataSheet3.ZIP › p-P70S6K/p-P70s6k.jpg]

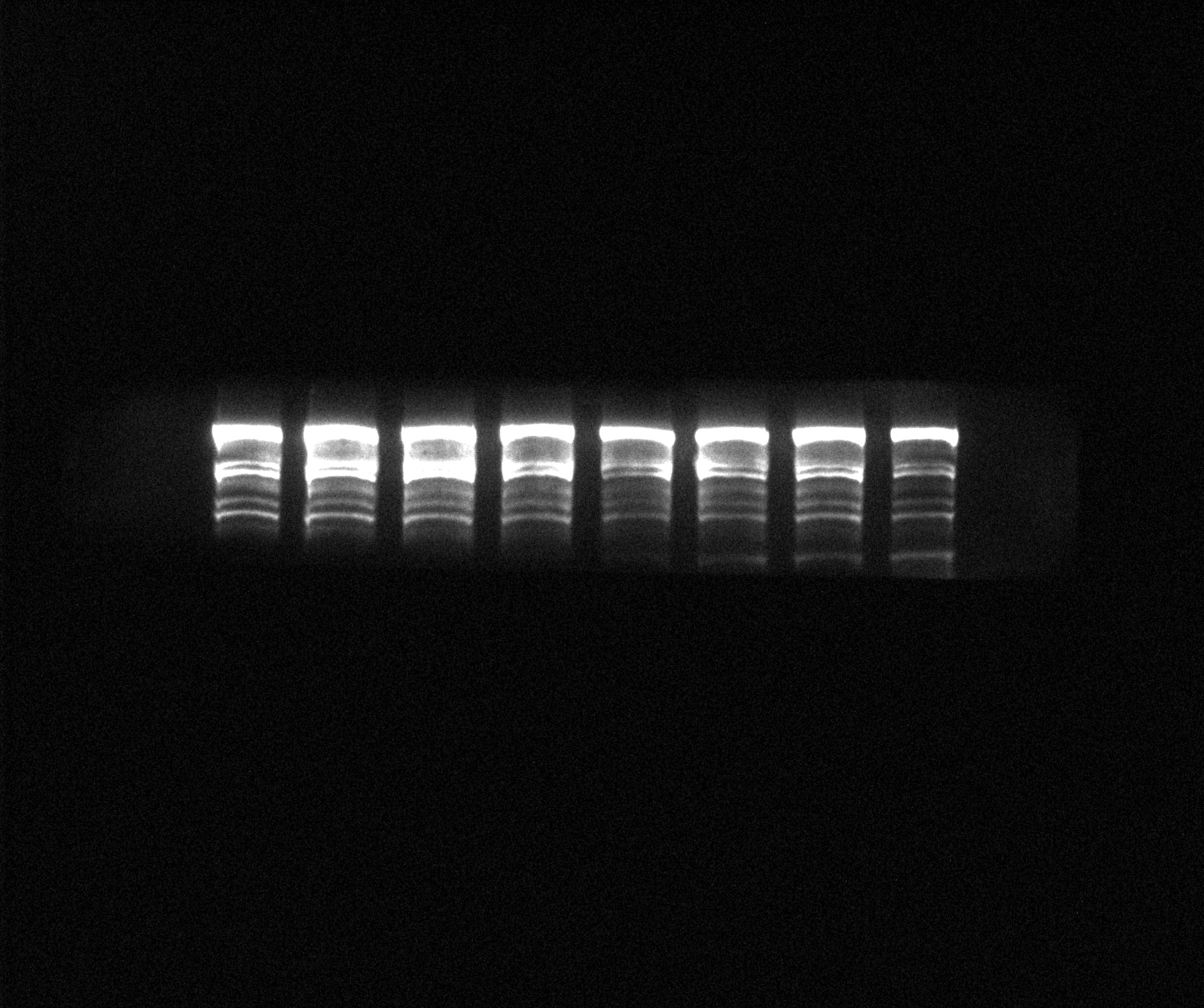

Supplement: Supplementary file 1 [file DataSheet3.ZIP › p-mTOR/P-mTOR-B-2.jpg]

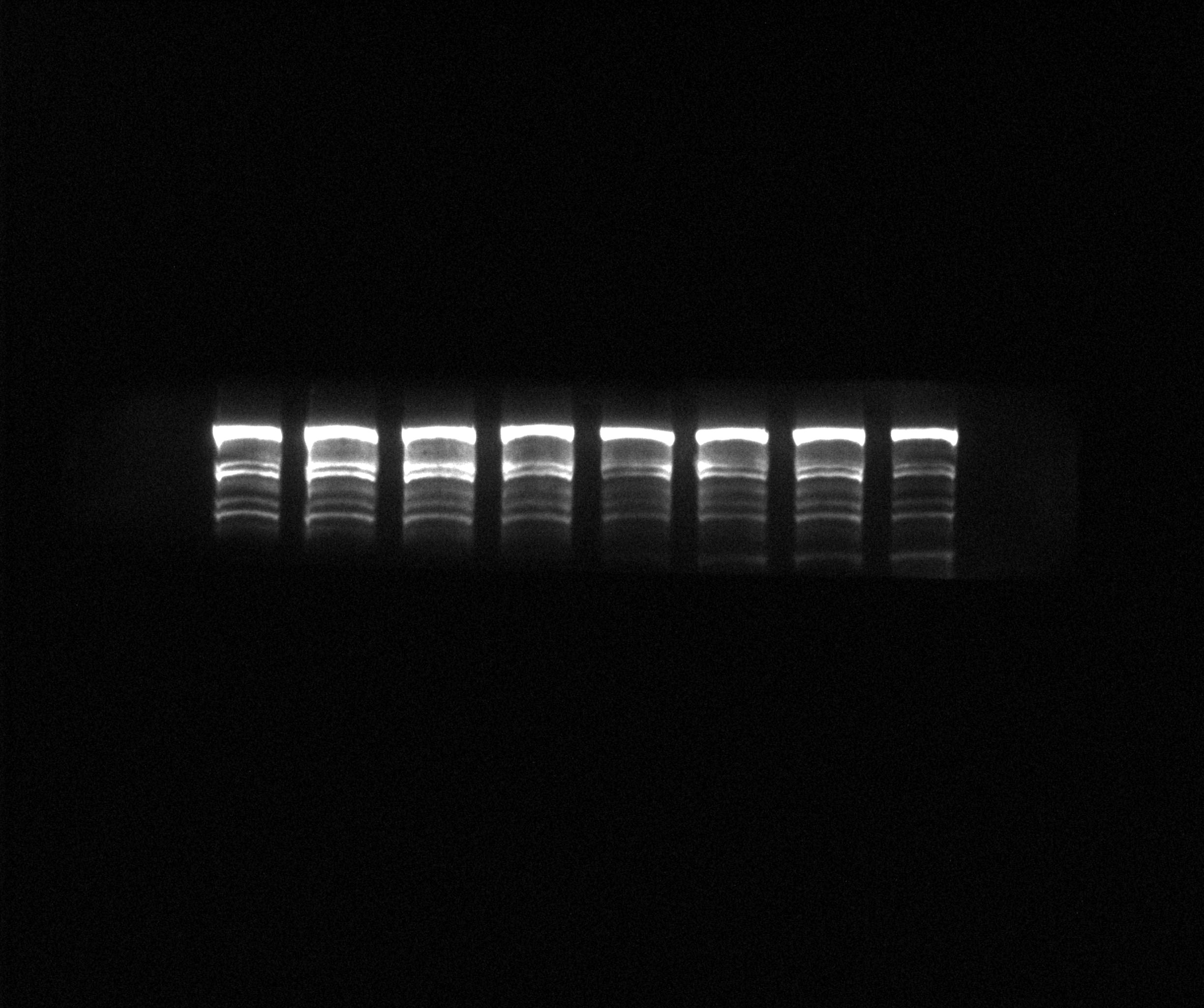

Supplement: Supplementary file 1 [file DataSheet3.ZIP › p-mTOR/P-mTOR-B-3.jpg]

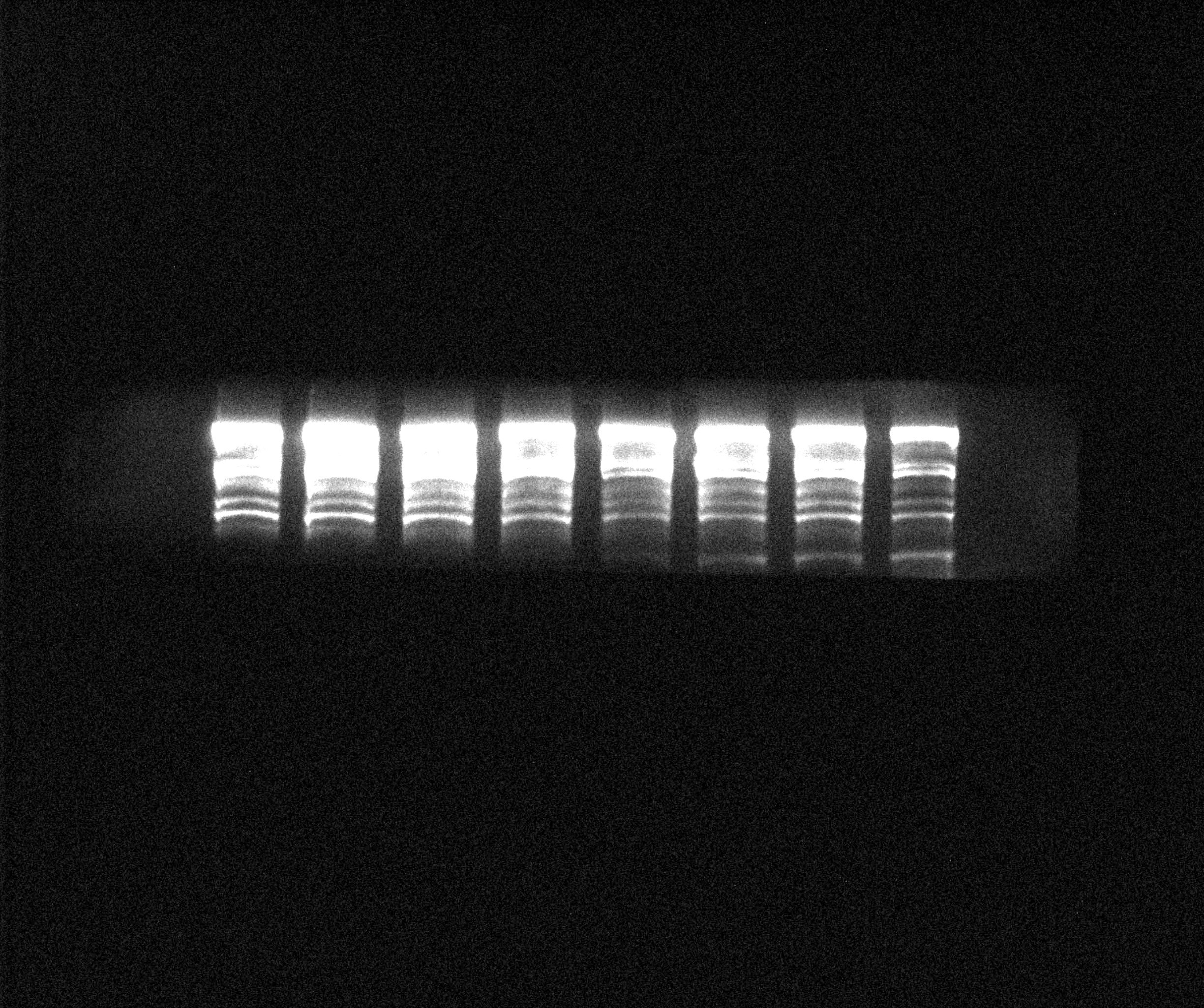

Supplement: Supplementary file 1 [file DataSheet3.ZIP › p-mTOR/P-mTOR-B-4.jpg]

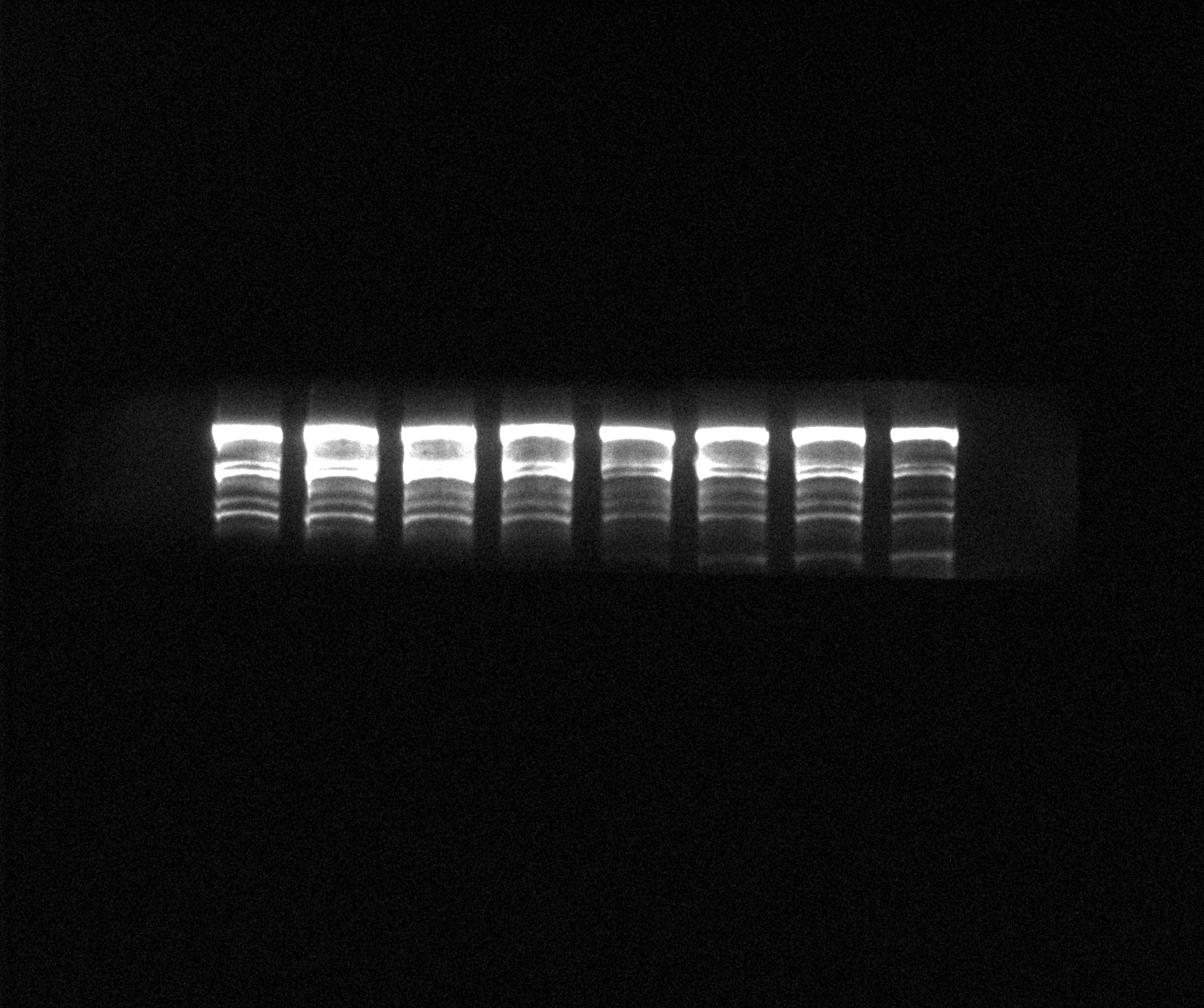

Supplement: Supplementary file 1 [file DataSheet3.ZIP › p-mTOR/P-mTOR-B.jpg]

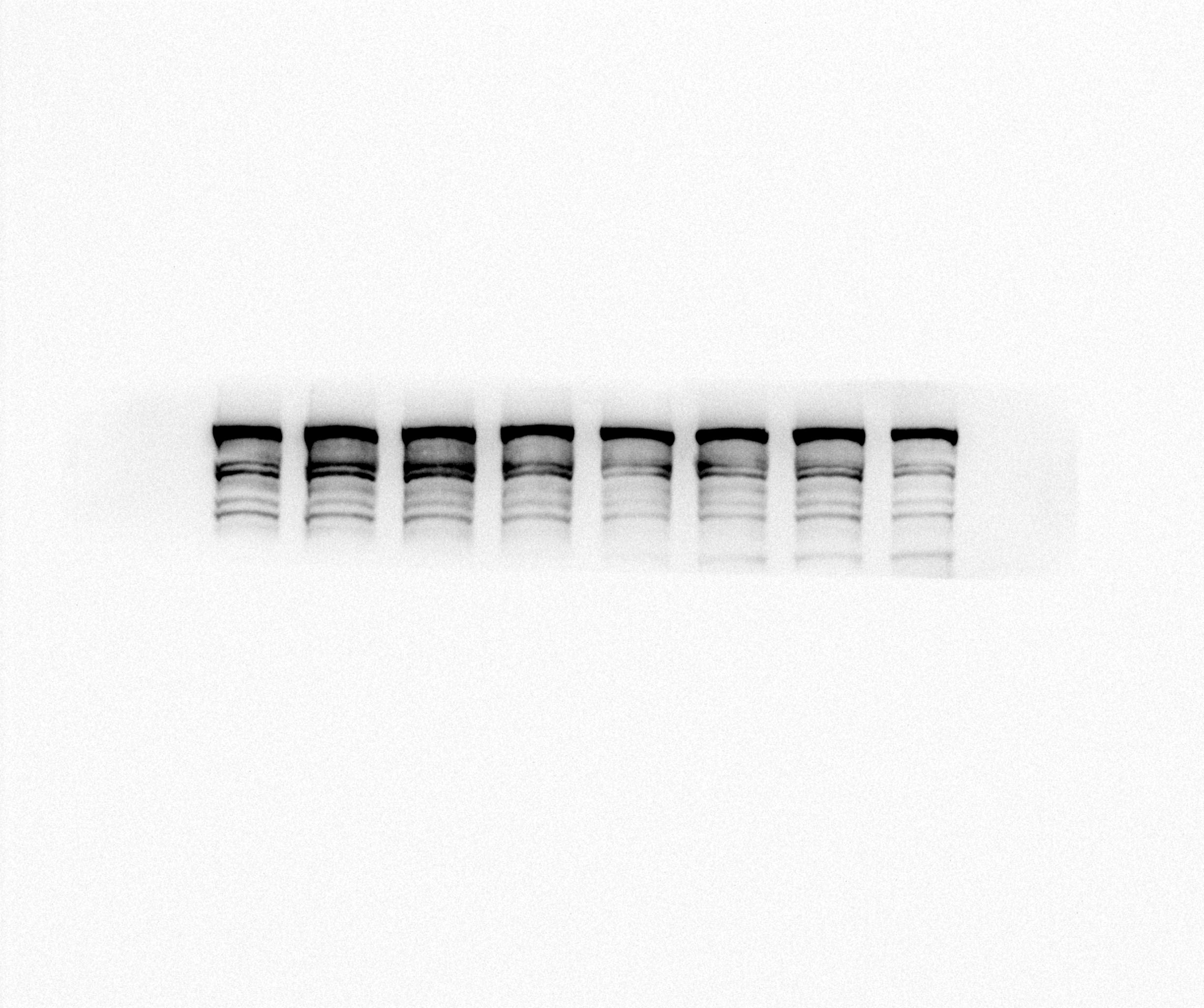

Supplement: Supplementary file 1 [file DataSheet3.ZIP › p-mTOR/P-mTOR-F-2.jpg]

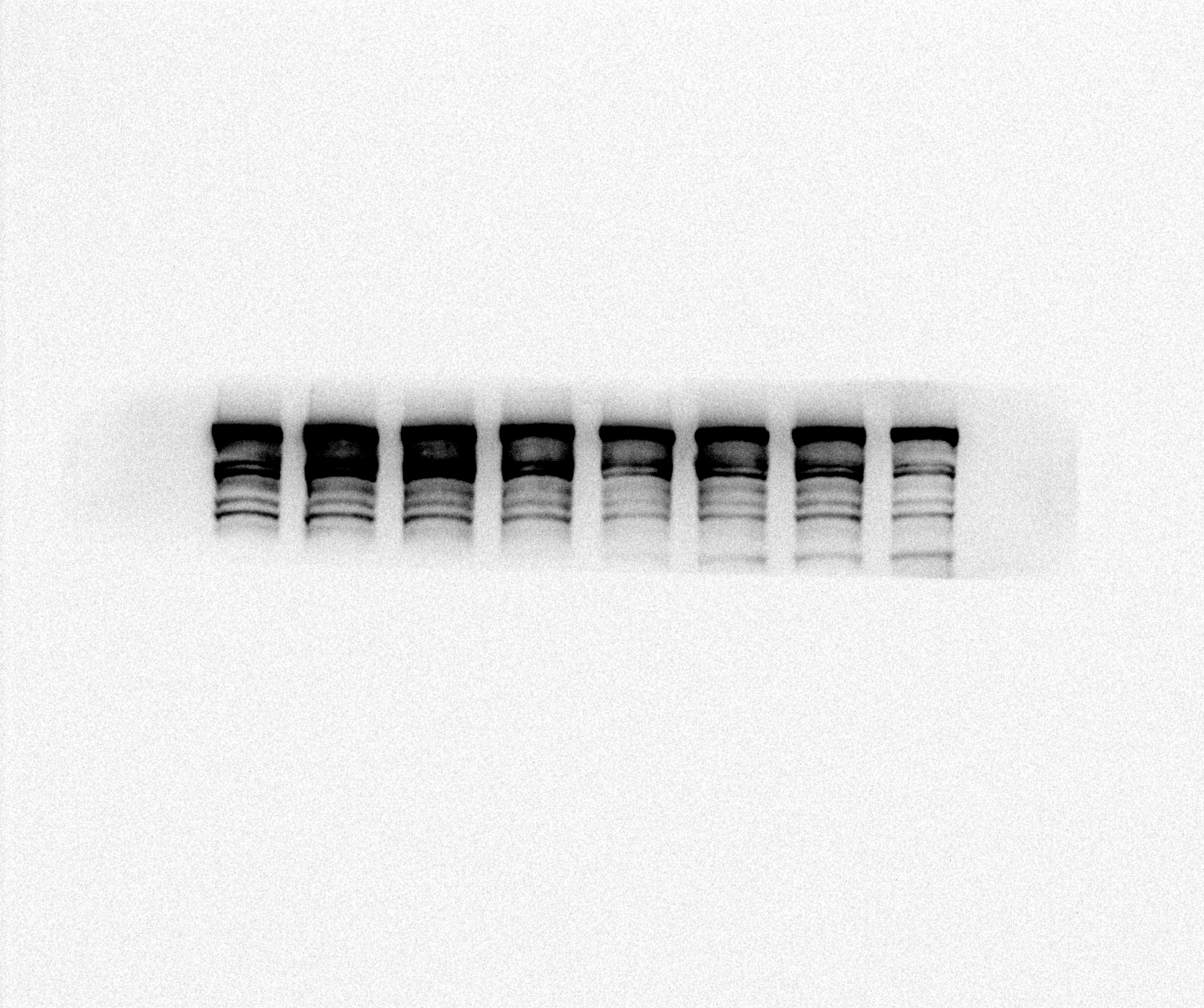

Supplement: Supplementary file 1 [file DataSheet3.ZIP › p-mTOR/P-mTOR-F-3.jpg]

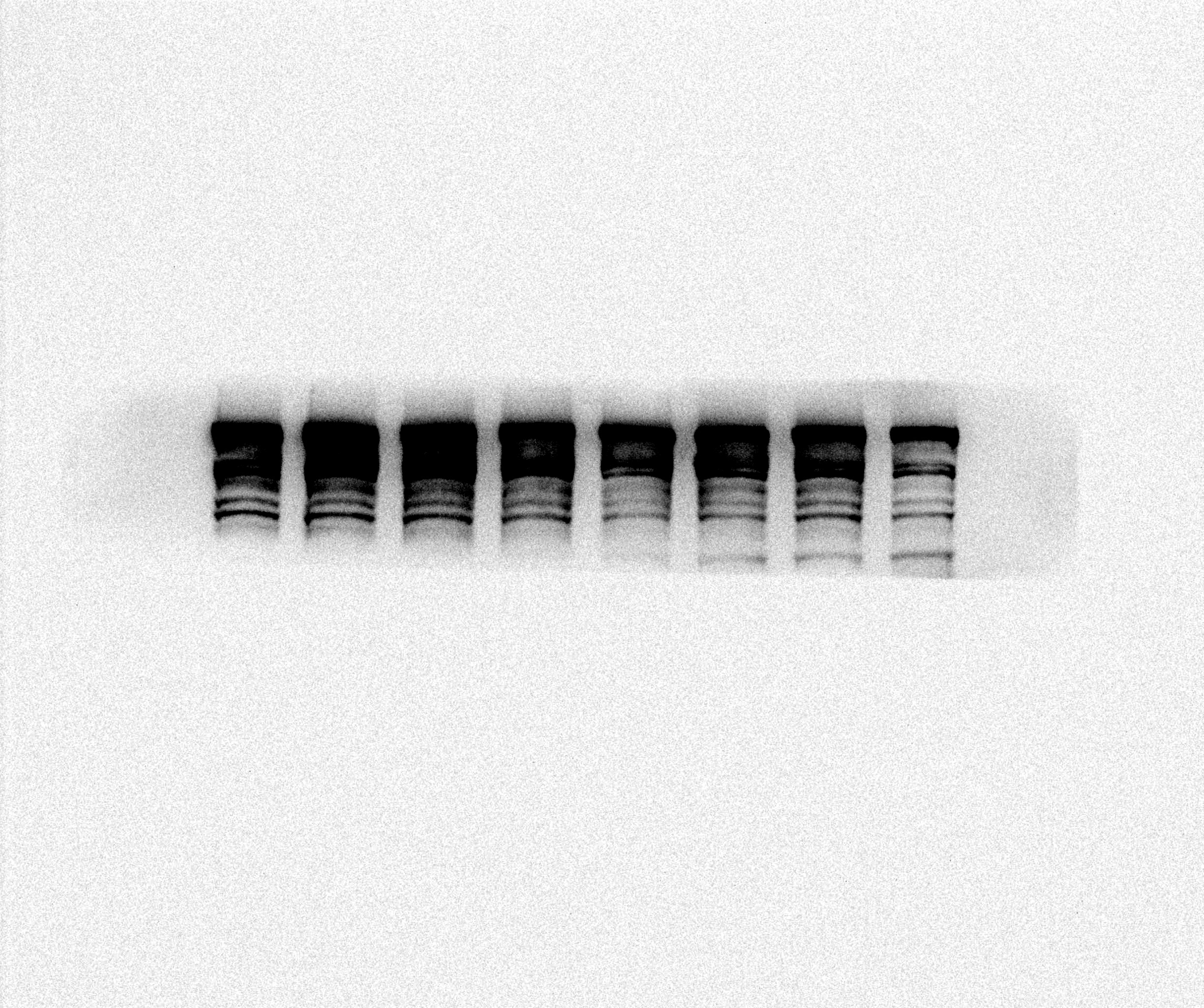

Supplement: Supplementary file 1 [file DataSheet3.ZIP › p-mTOR/P-mTOR-F-4.jpg]

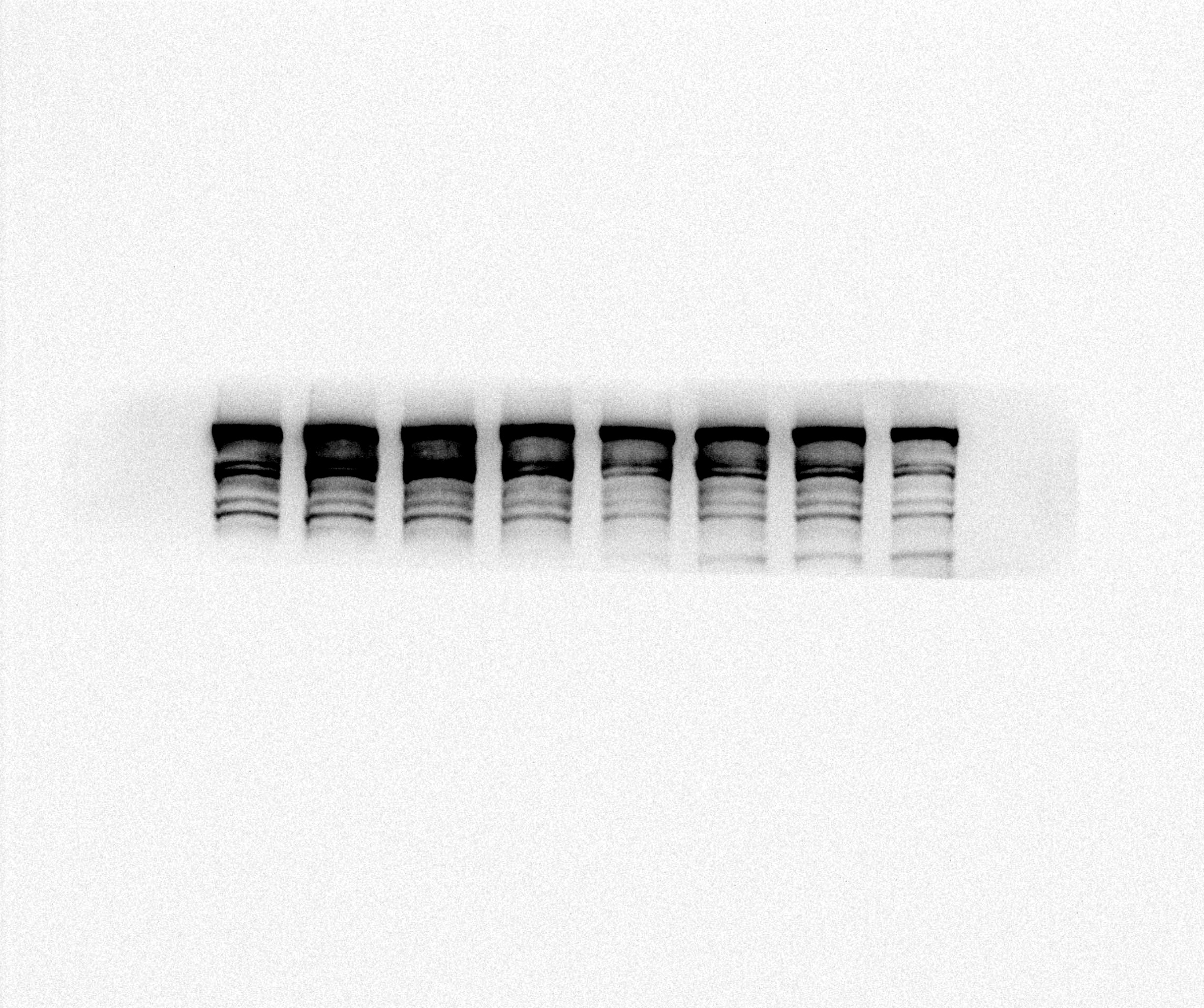

Supplement: Supplementary file 1 [file DataSheet3.ZIP › p-mTOR/P-mTOR-F.jpg]

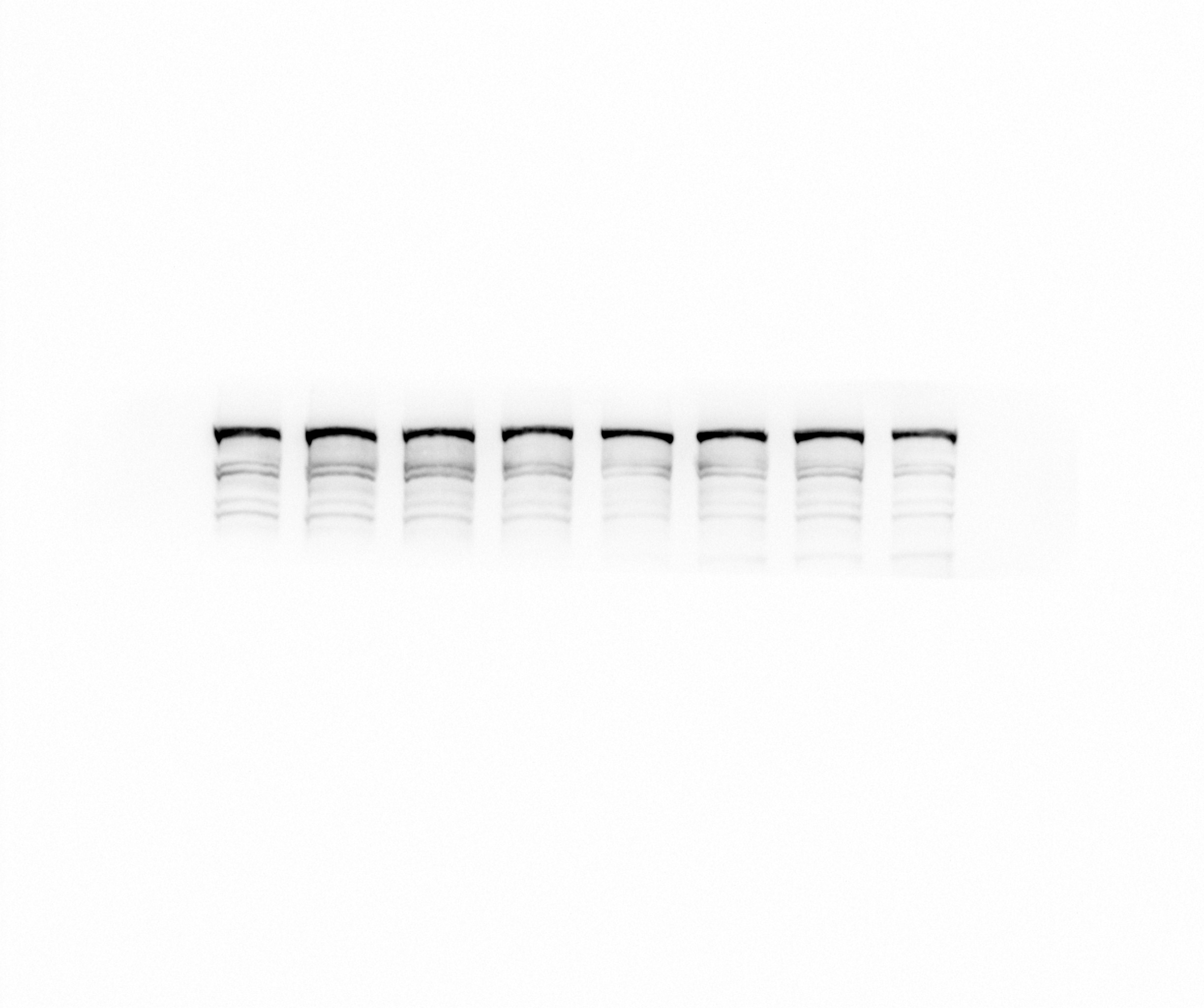

Supplement: Supplementary file 1 [file DataSheet3.ZIP › p-mTOR/P-mTOR.jpg]

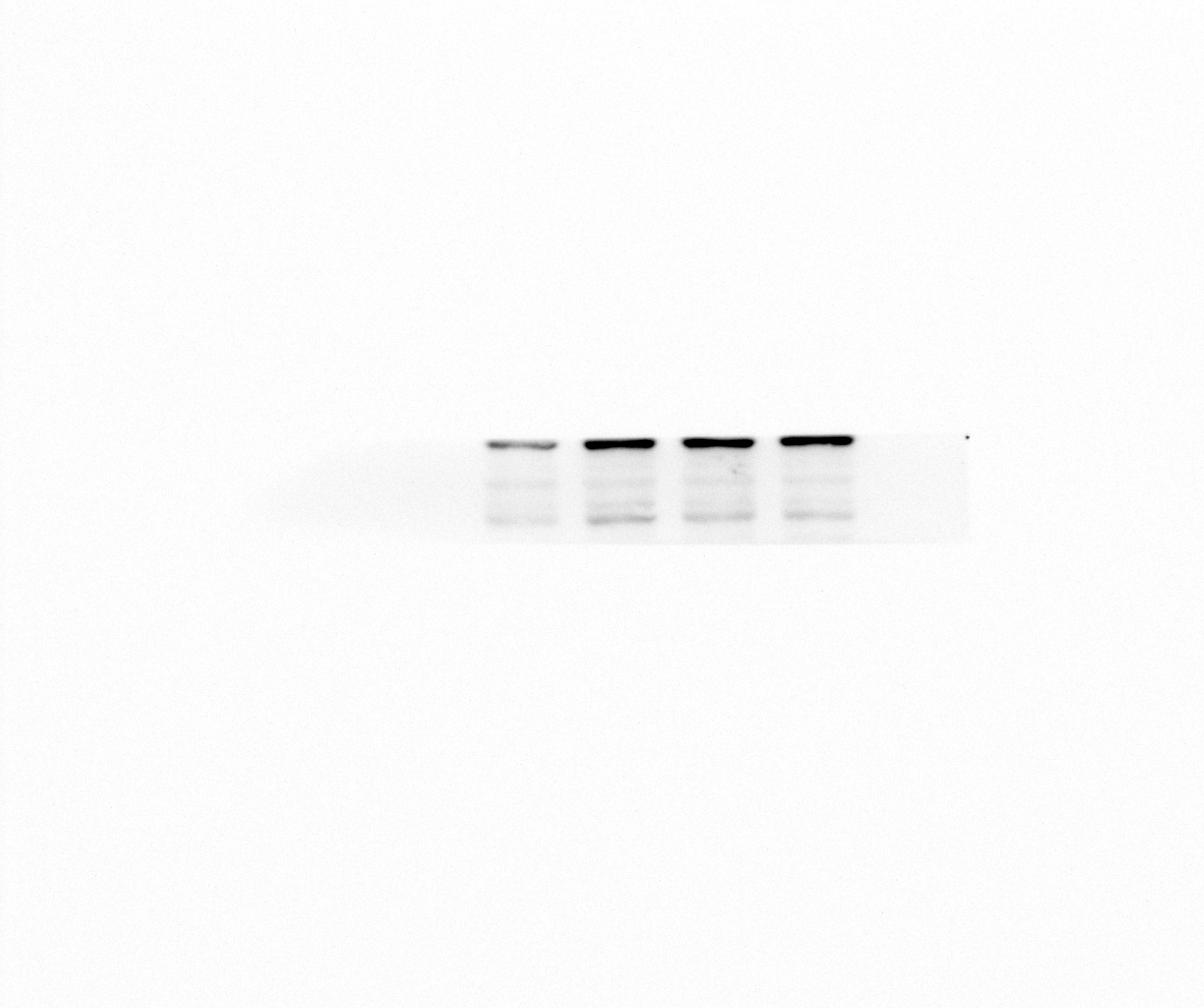

Supplement: Supplementary file 1 [file DataSheet3.ZIP › p-p38/P-P38-2.jpg]

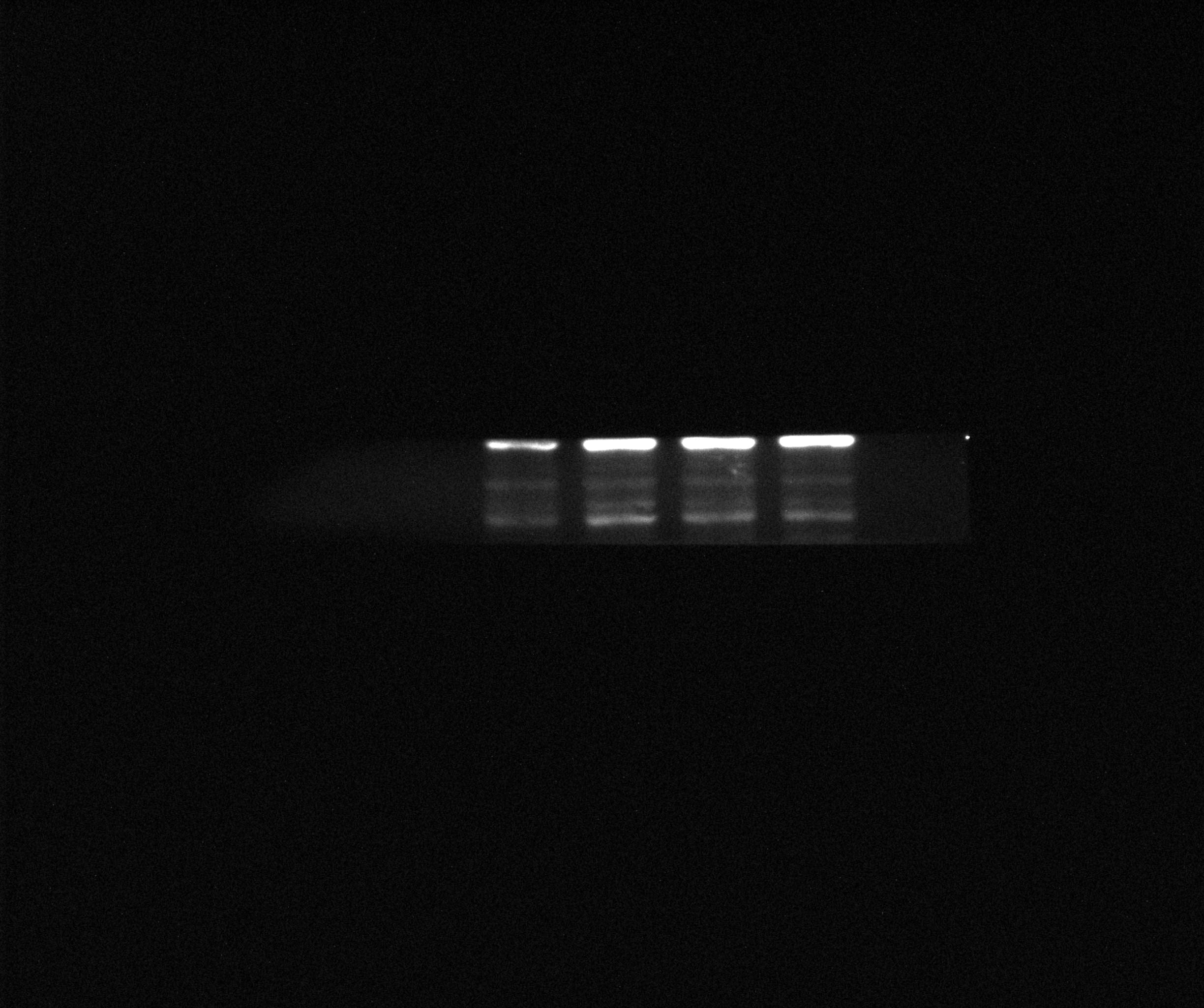

Supplement: Supplementary file 1 [file DataSheet3.ZIP › p-p38/P-P38-B-2.jpg]

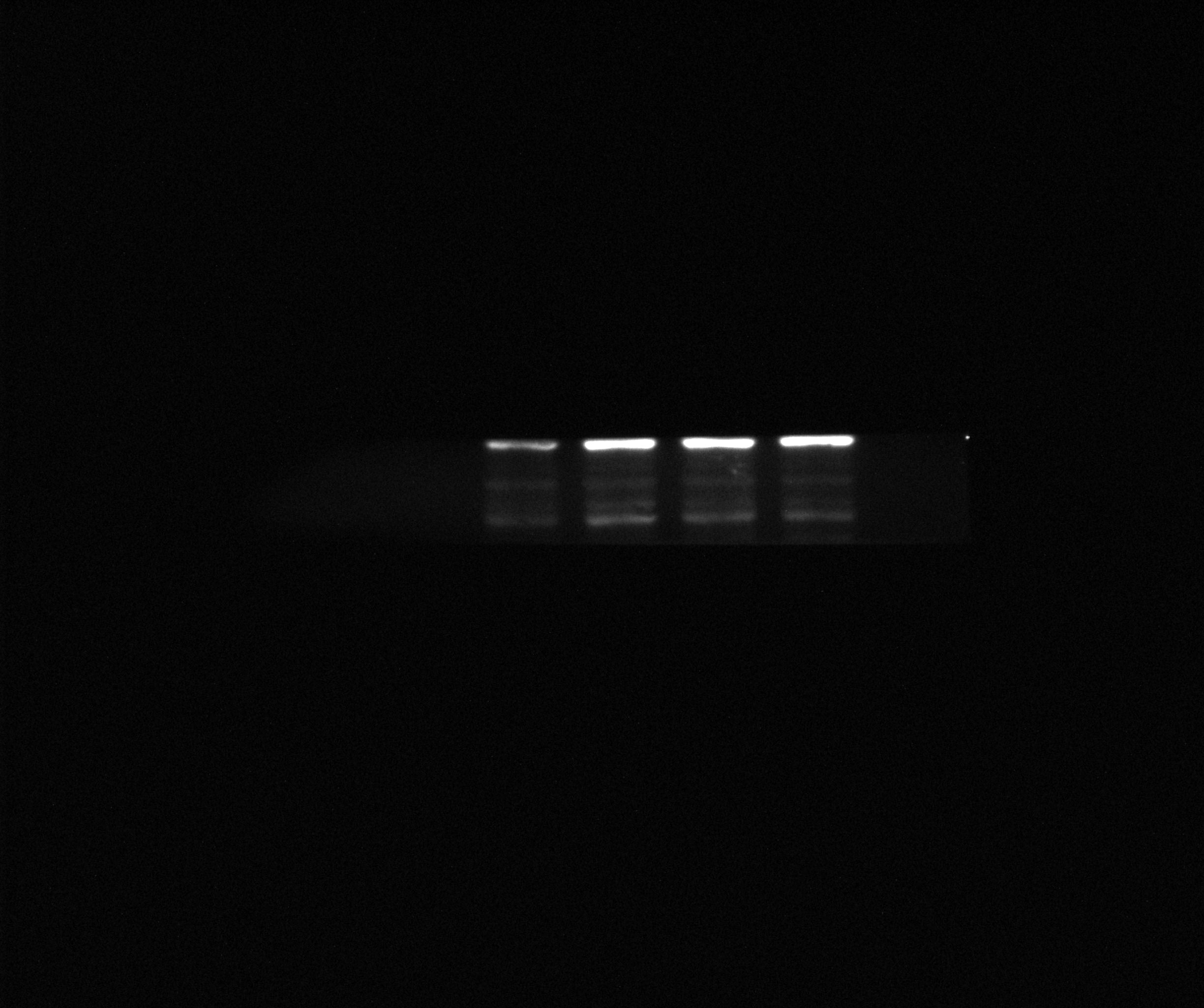

Supplement: Supplementary file 1 [file DataSheet3.ZIP › p-p38/P-P38-B.jpg]

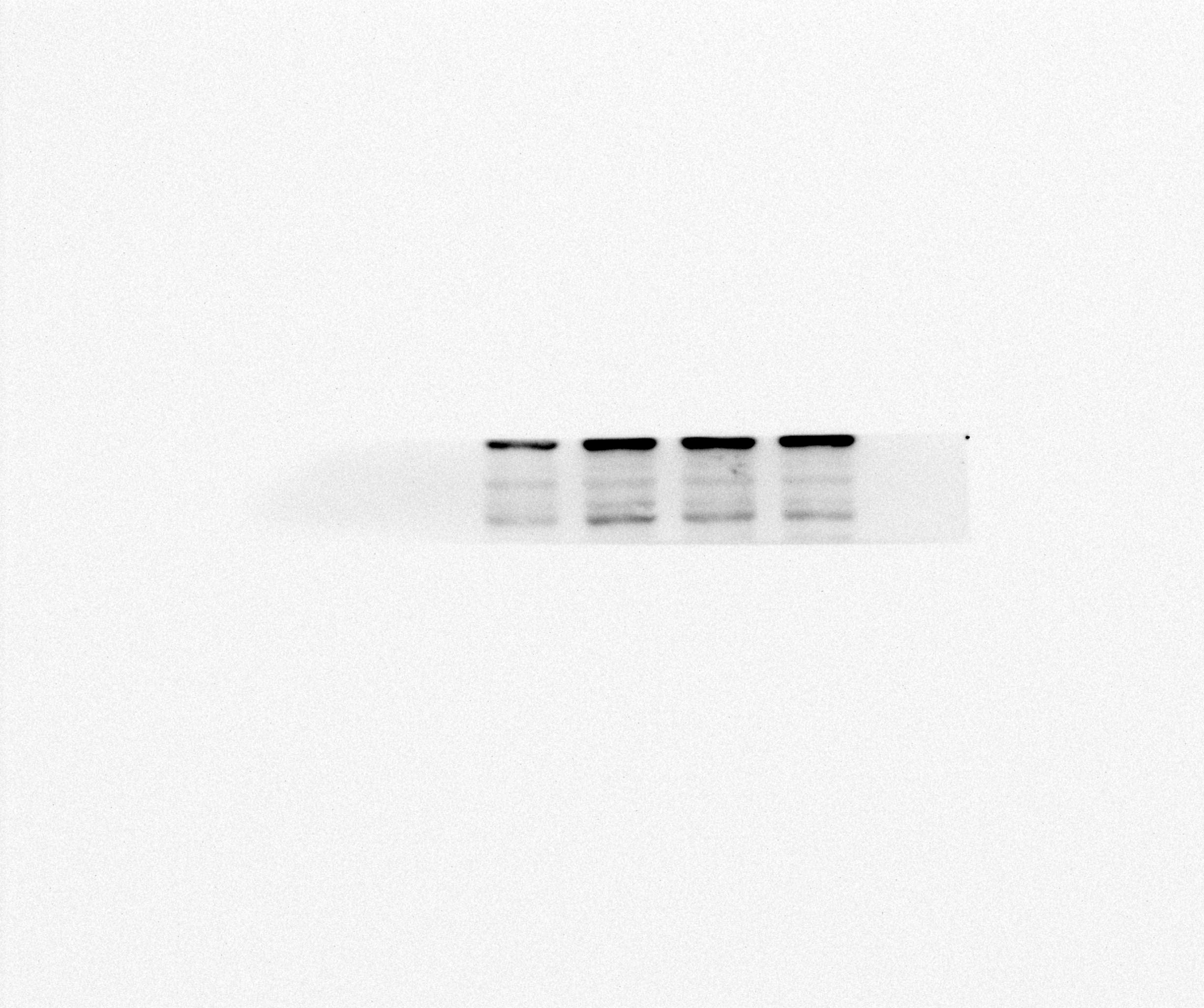

Supplement: Supplementary file 1 [file DataSheet3.ZIP › p-p38/P-P38-F-2.jpg]

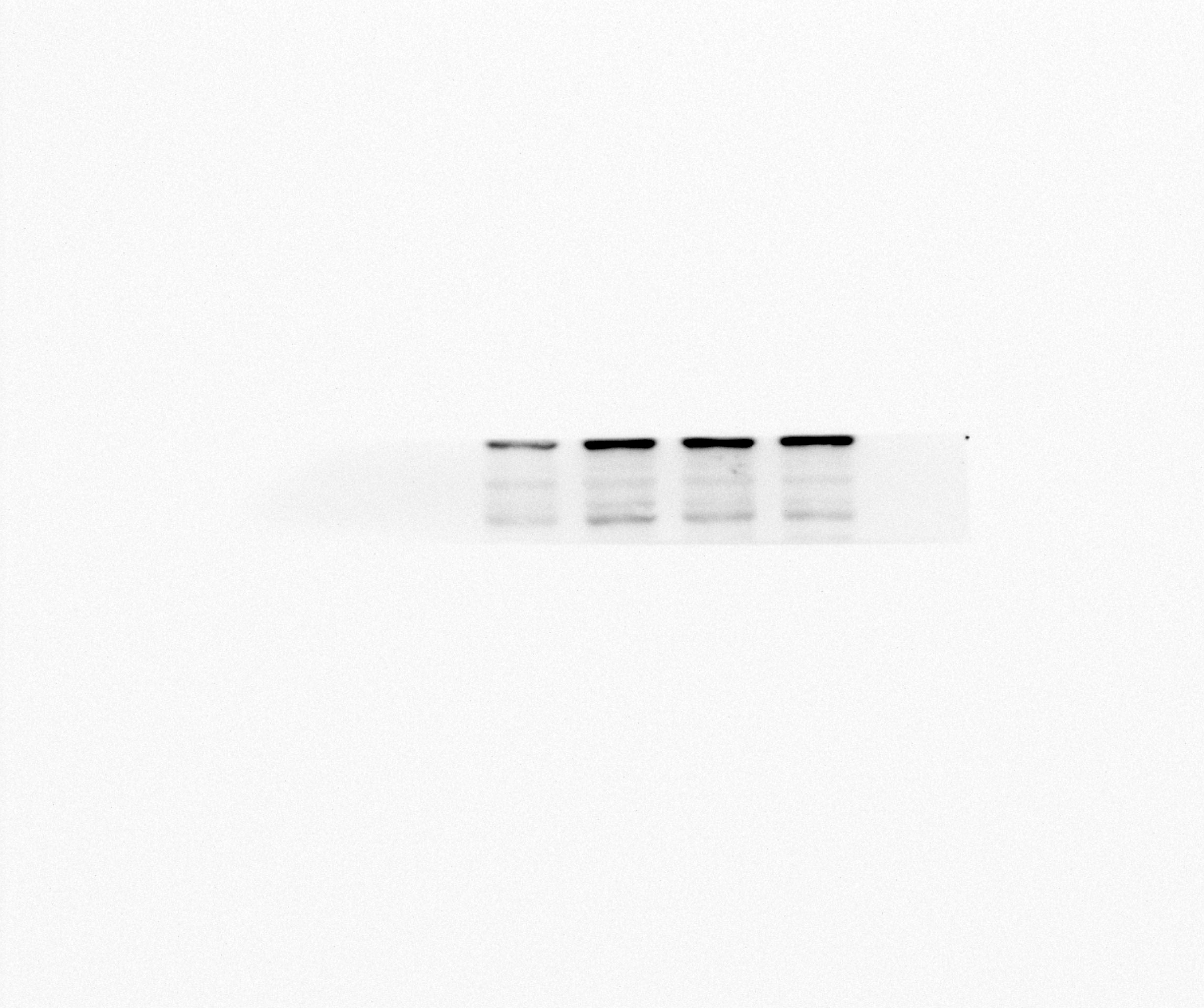

Supplement: Supplementary file 1 [file DataSheet3.ZIP › p-p38/P-P38-F.jpg]

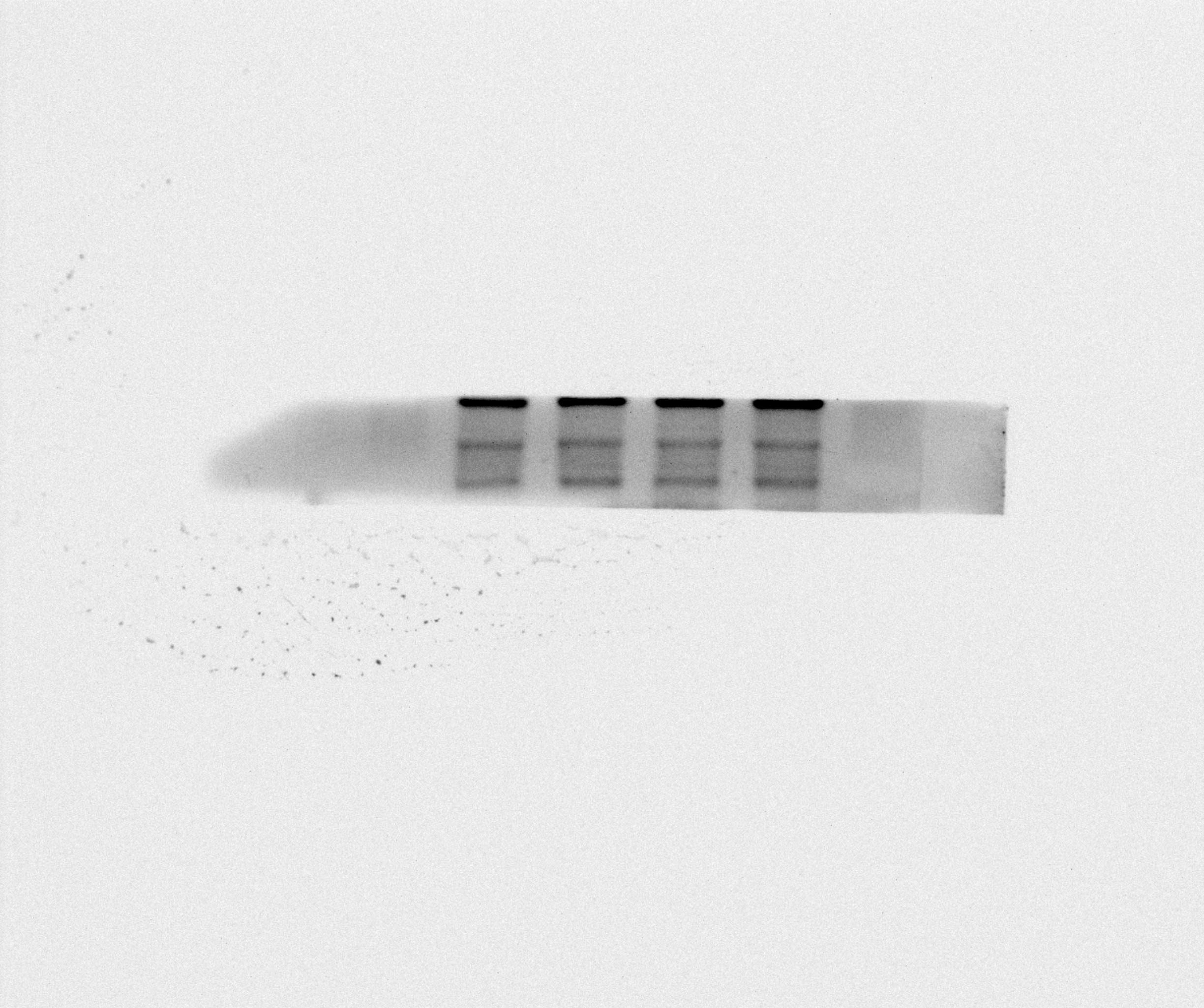

Supplement: Supplementary file 1 [file DataSheet3.ZIP › p38/P38-2.jpg]

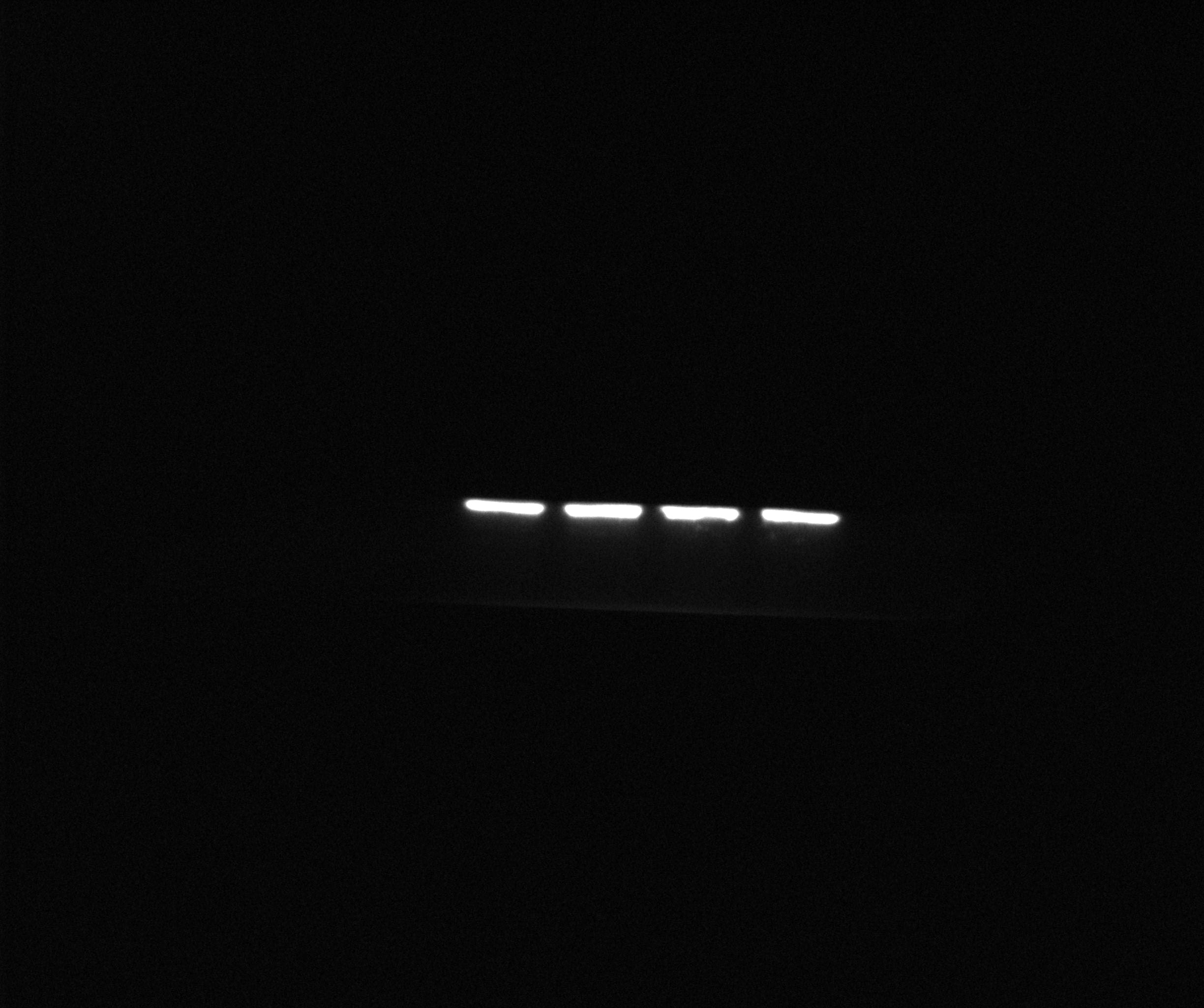

Supplement: Supplementary file 1 [file DataSheet3.ZIP › p38/P38-B-2.jpg]

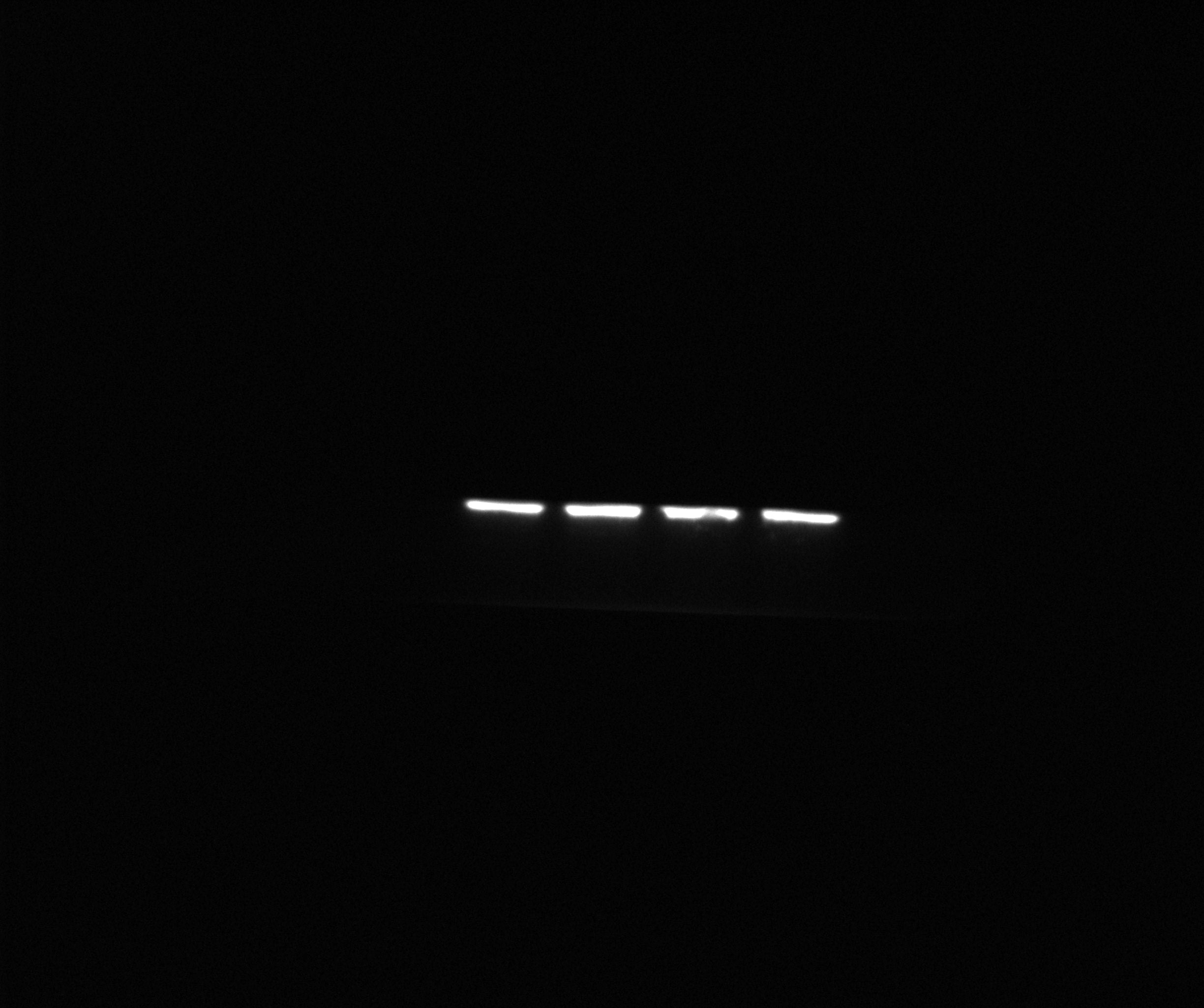

Supplement: Supplementary file 1 [file DataSheet3.ZIP › p38/P38-B.jpg]

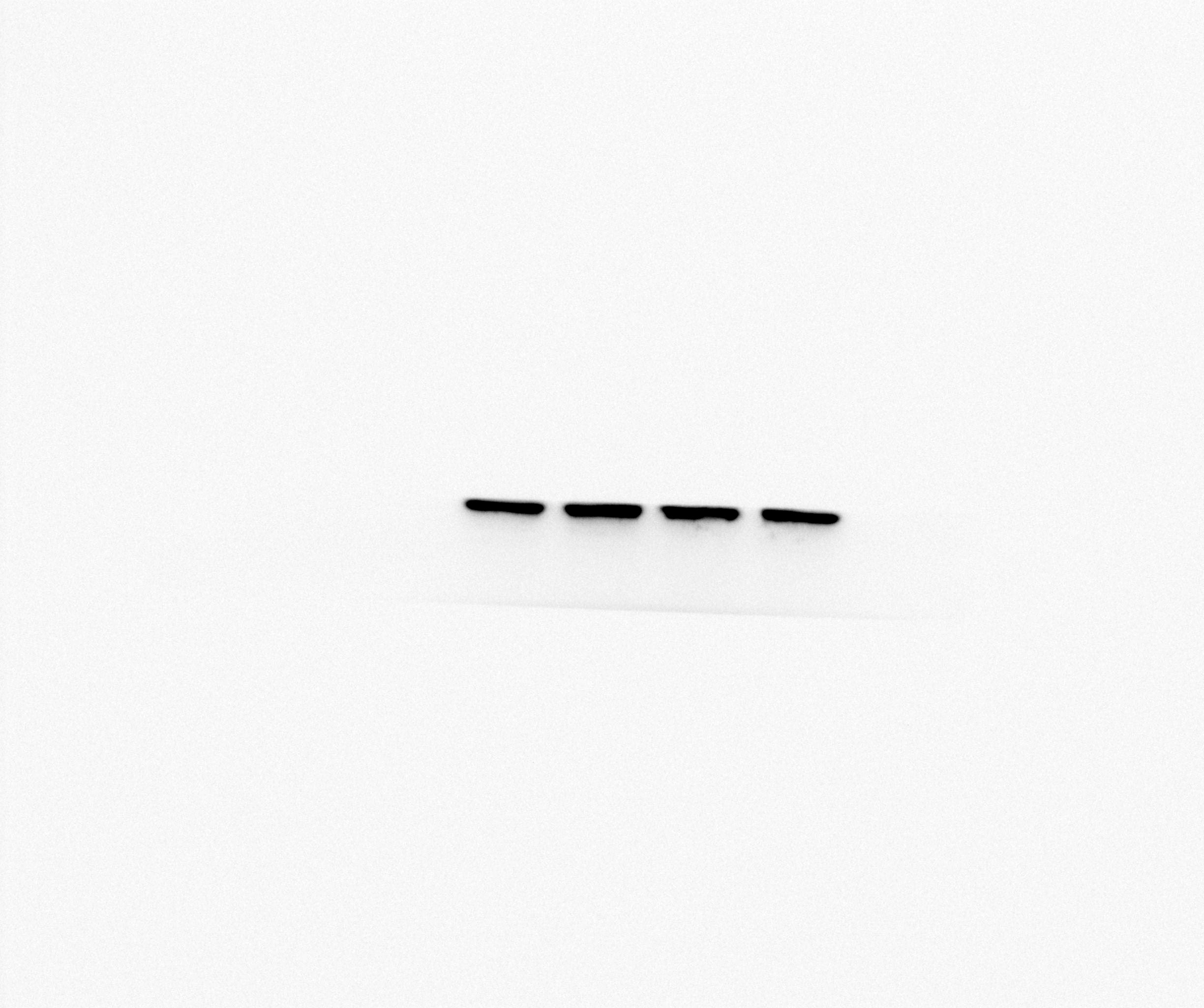

Supplement: Supplementary file 1 [file DataSheet3.ZIP › p38/P38-F-2.jpg]

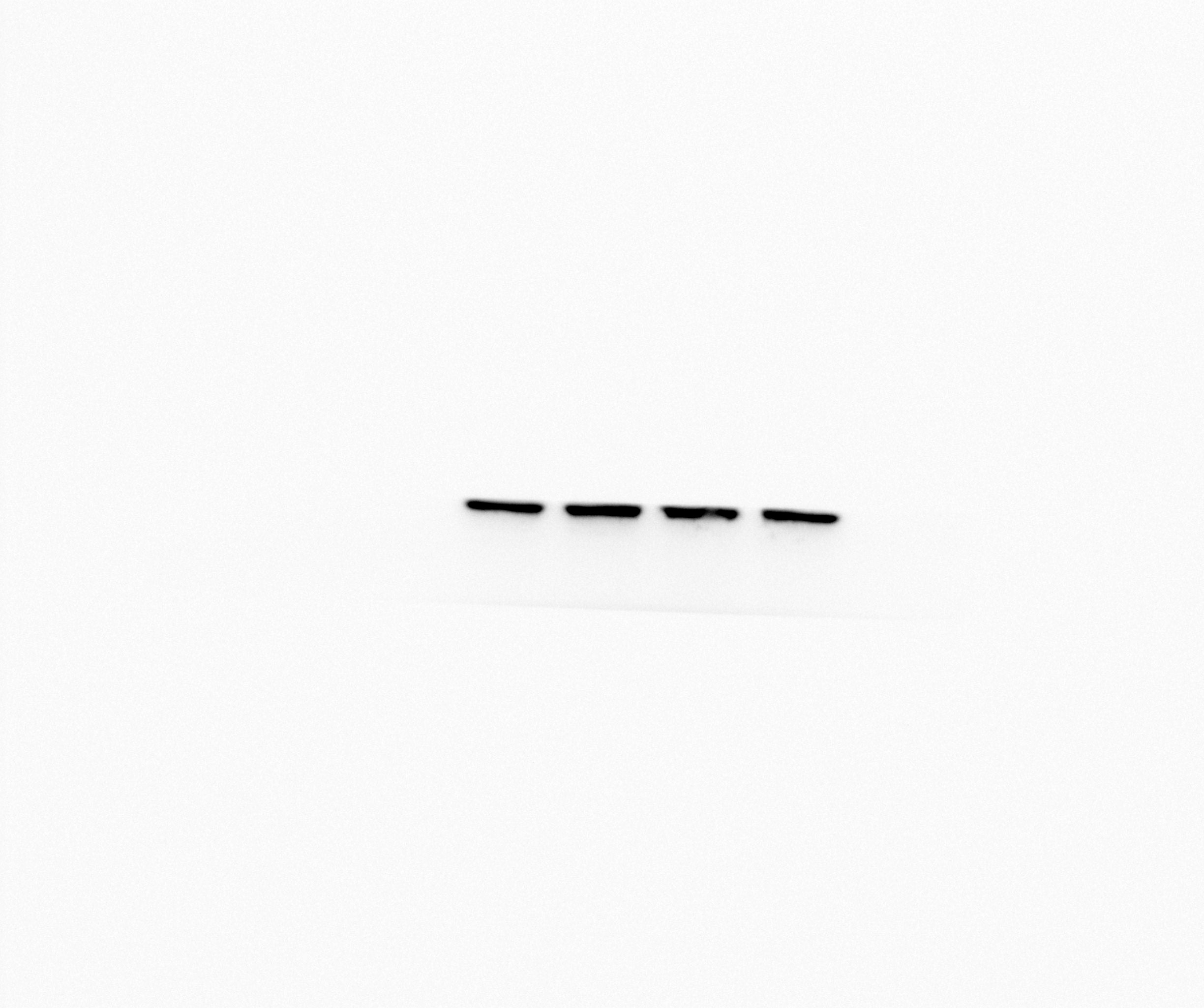

Supplement: Supplementary file 1 [file DataSheet3.ZIP › p38/P38-F.jpg]

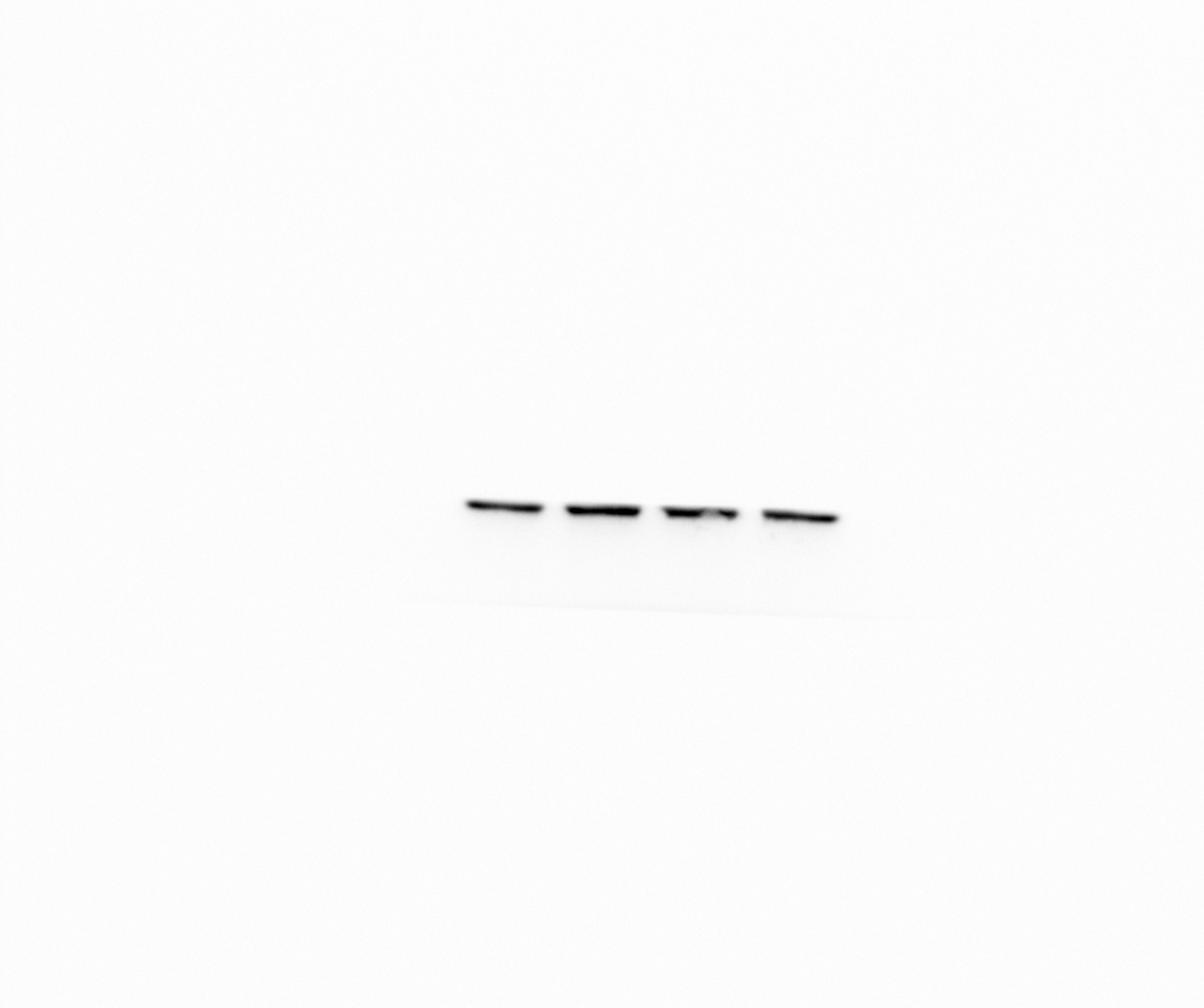

Supplement: Supplementary file 1 [file DataSheet3.ZIP › p38/P38.jpg]

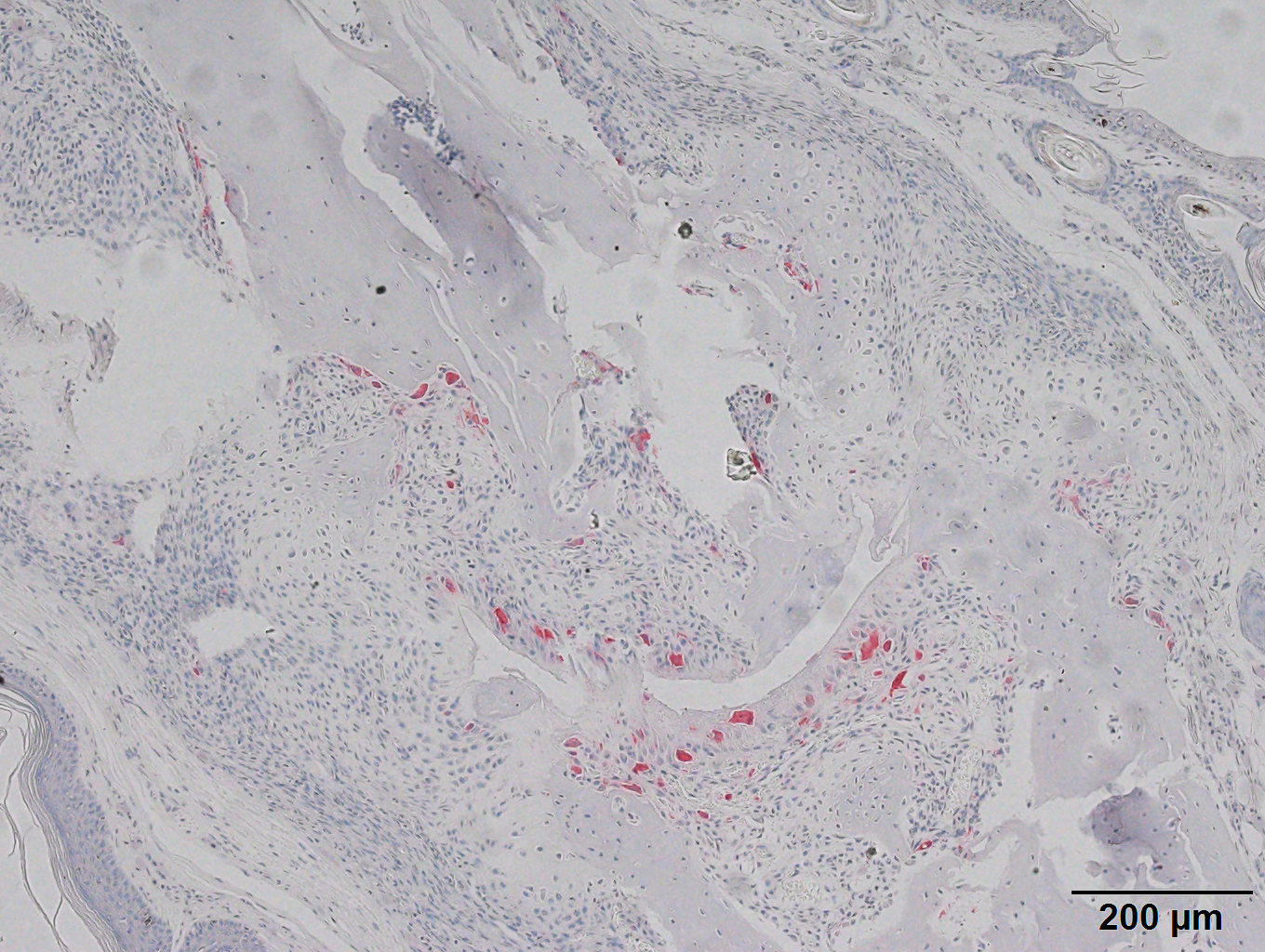

Supplement: Supplementary file 2 [file DataSheet8.ZIP › Fig 6D/CIA (1).tif]

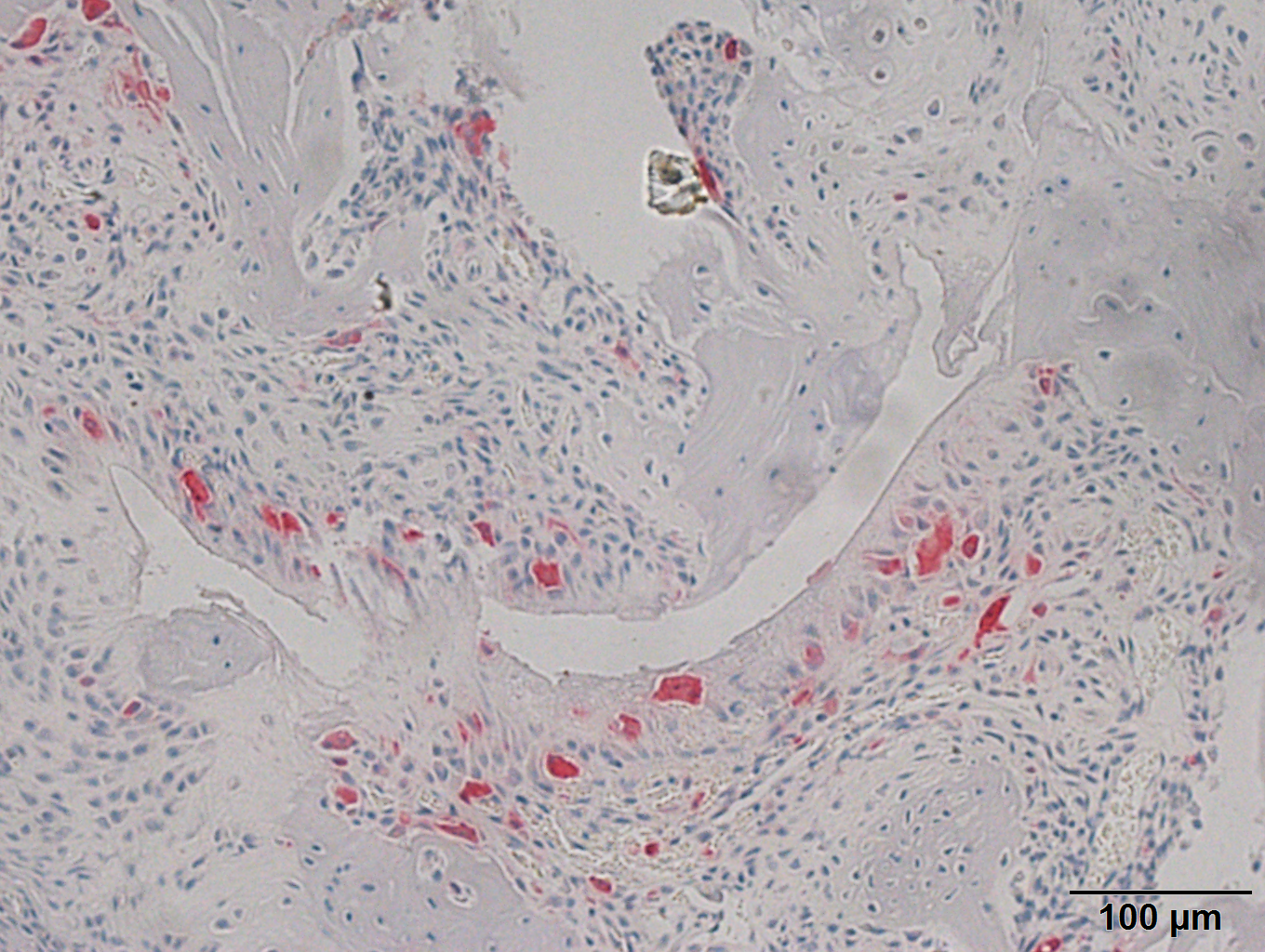

Supplement: Supplementary file 2 [file DataSheet8.ZIP › Fig 6D/CIA (2).tif]

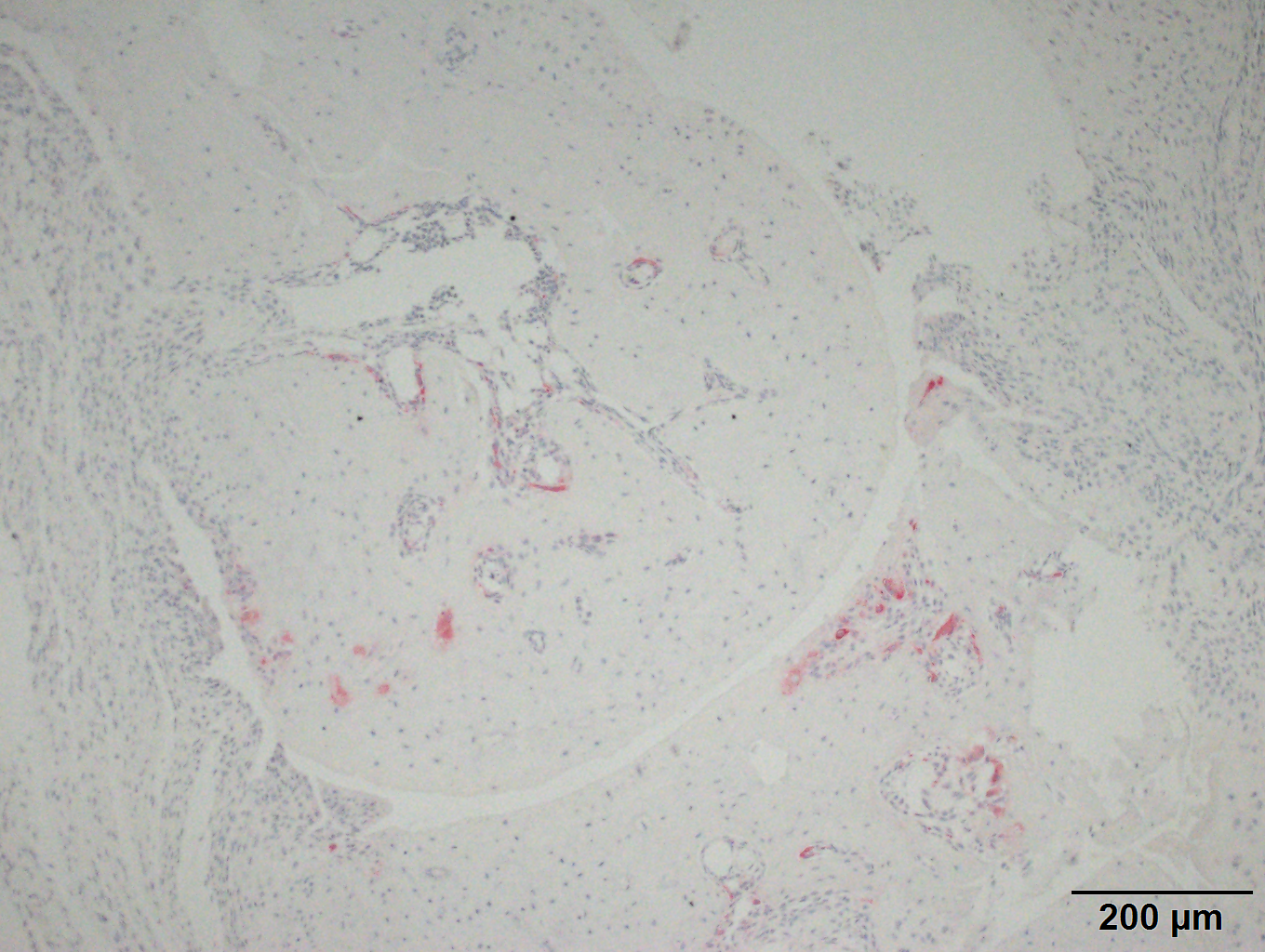

Supplement: Supplementary file 2 [file DataSheet8.ZIP › Fig 6D/CIA+PTX (1).tif]

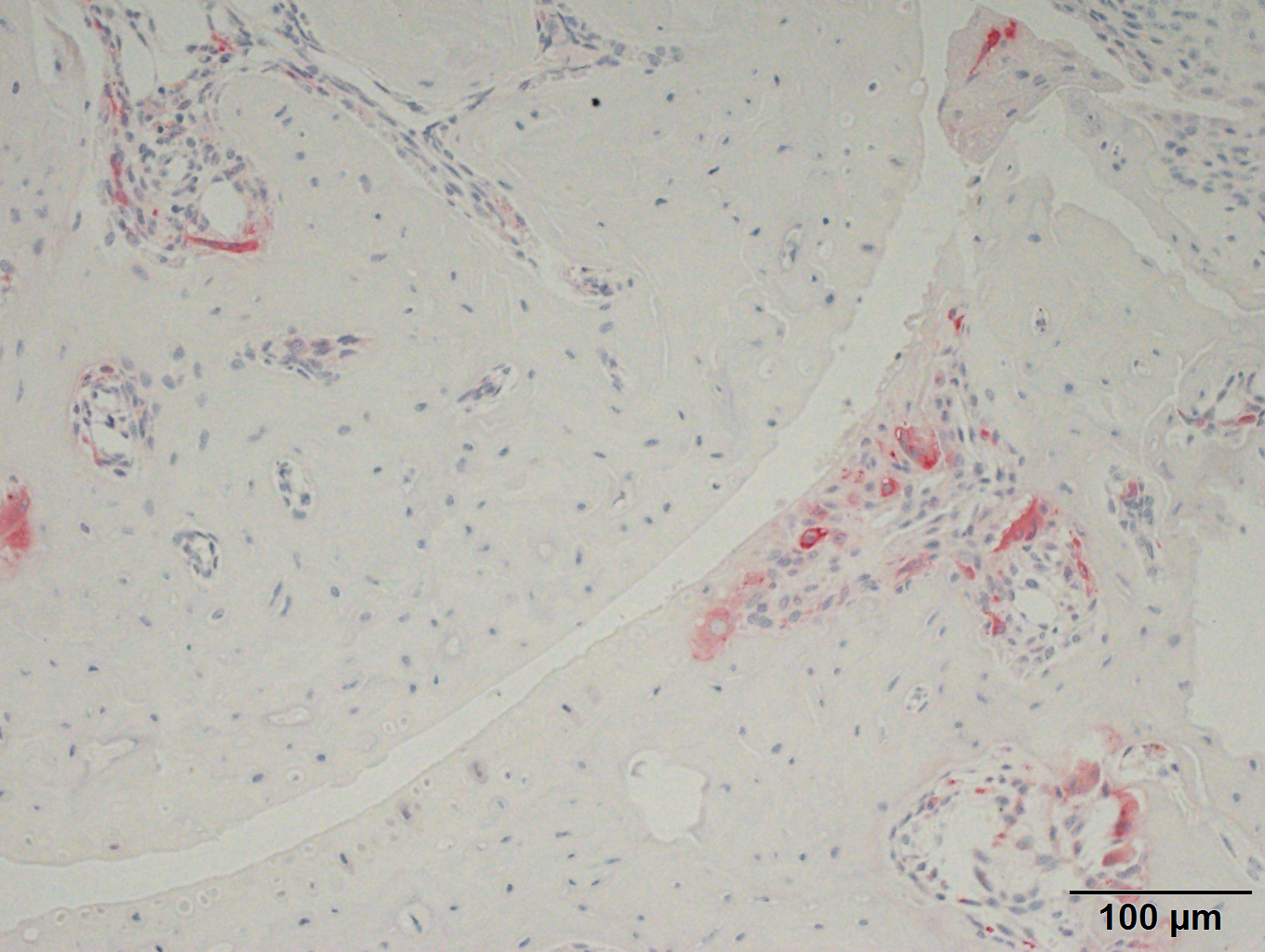

Supplement: Supplementary file 2 [file DataSheet8.ZIP › Fig 6D/CIA+PTX (2).tif]

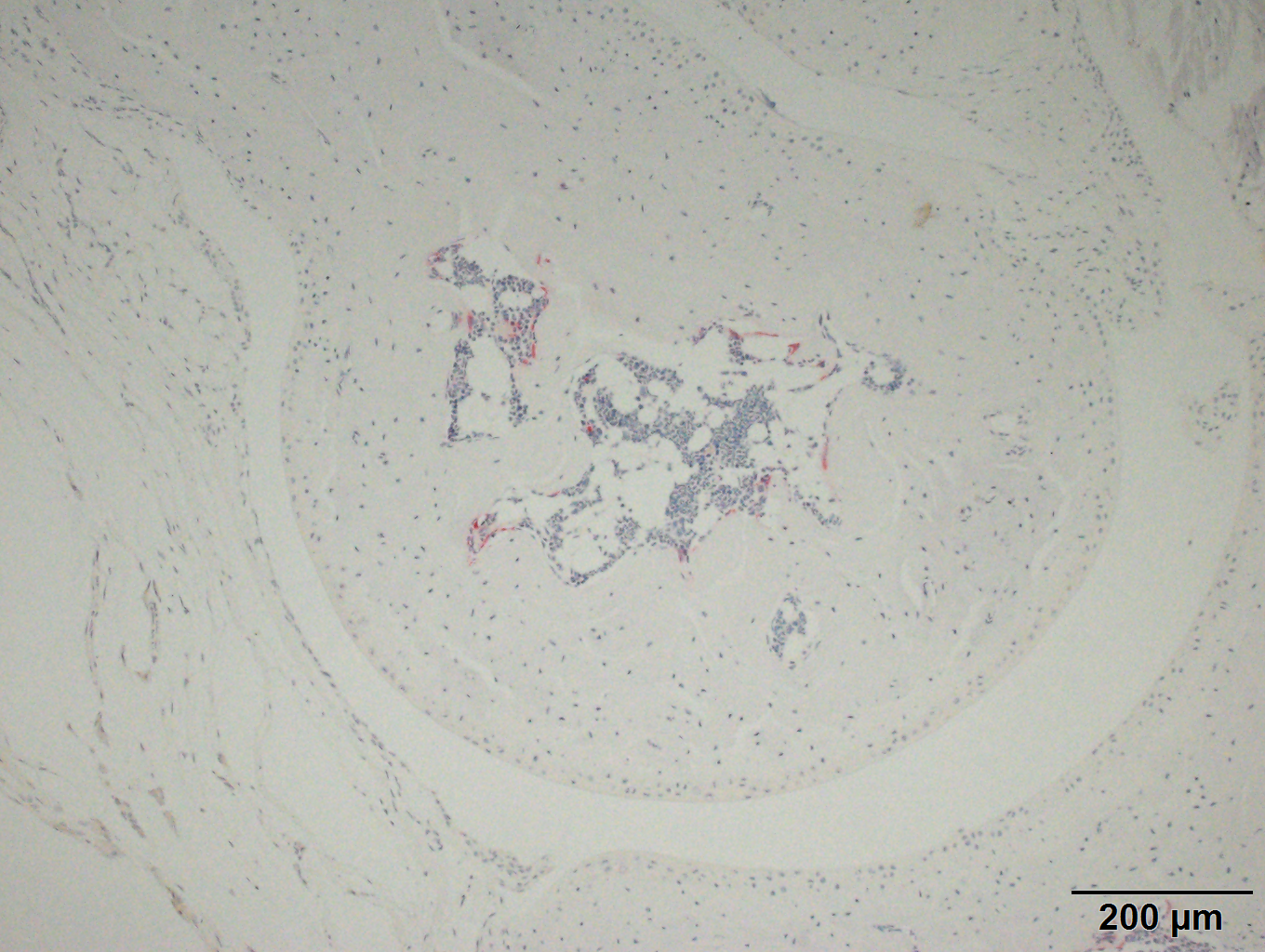

Supplement: Supplementary file 2 [file DataSheet8.ZIP › Fig 6D/Ctrlú¿1ú⌐.tif]

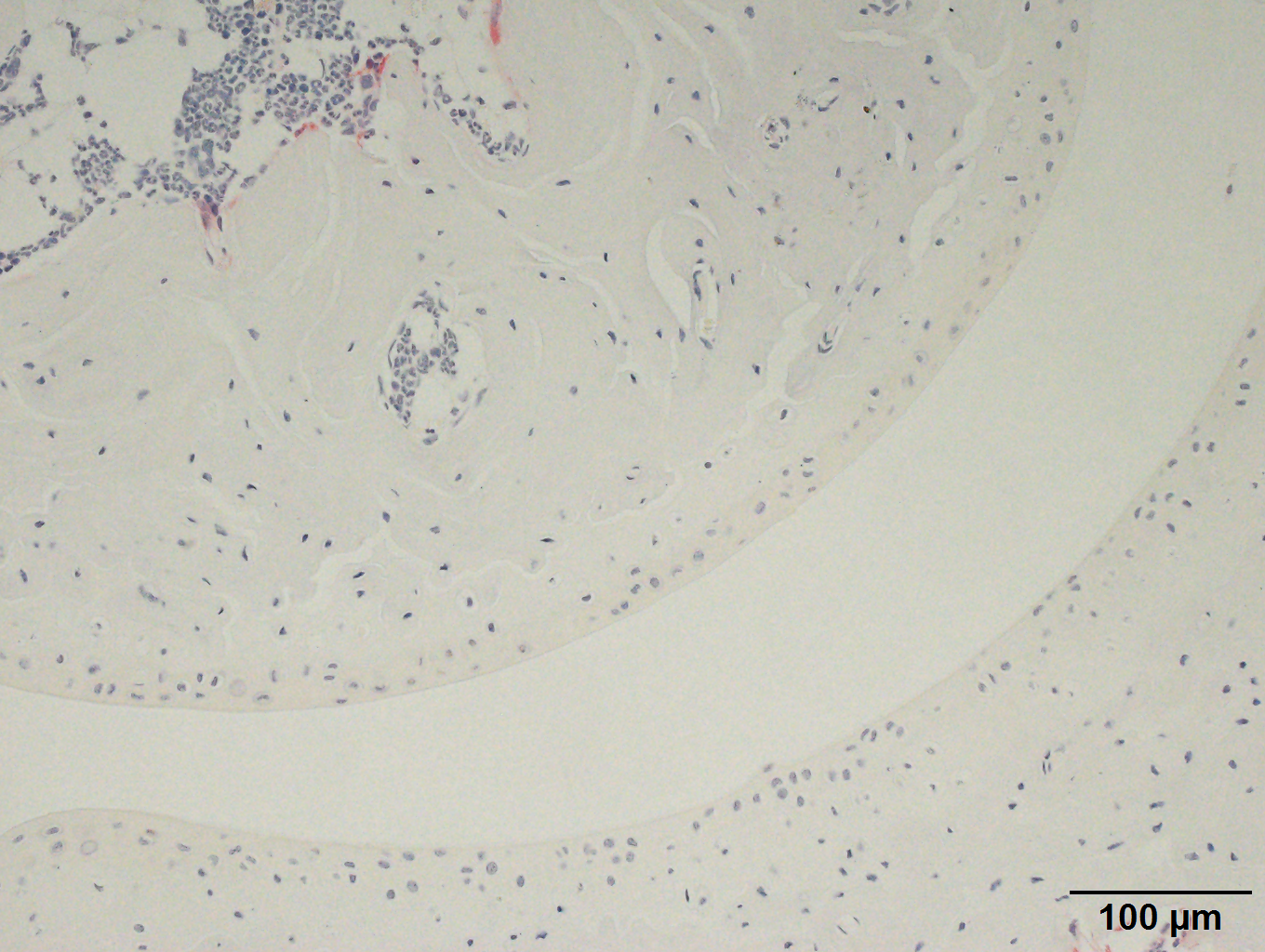

Supplement: Supplementary file 2 [file DataSheet8.ZIP › Fig 6D/Ctrlú¿2ú⌐.tif]

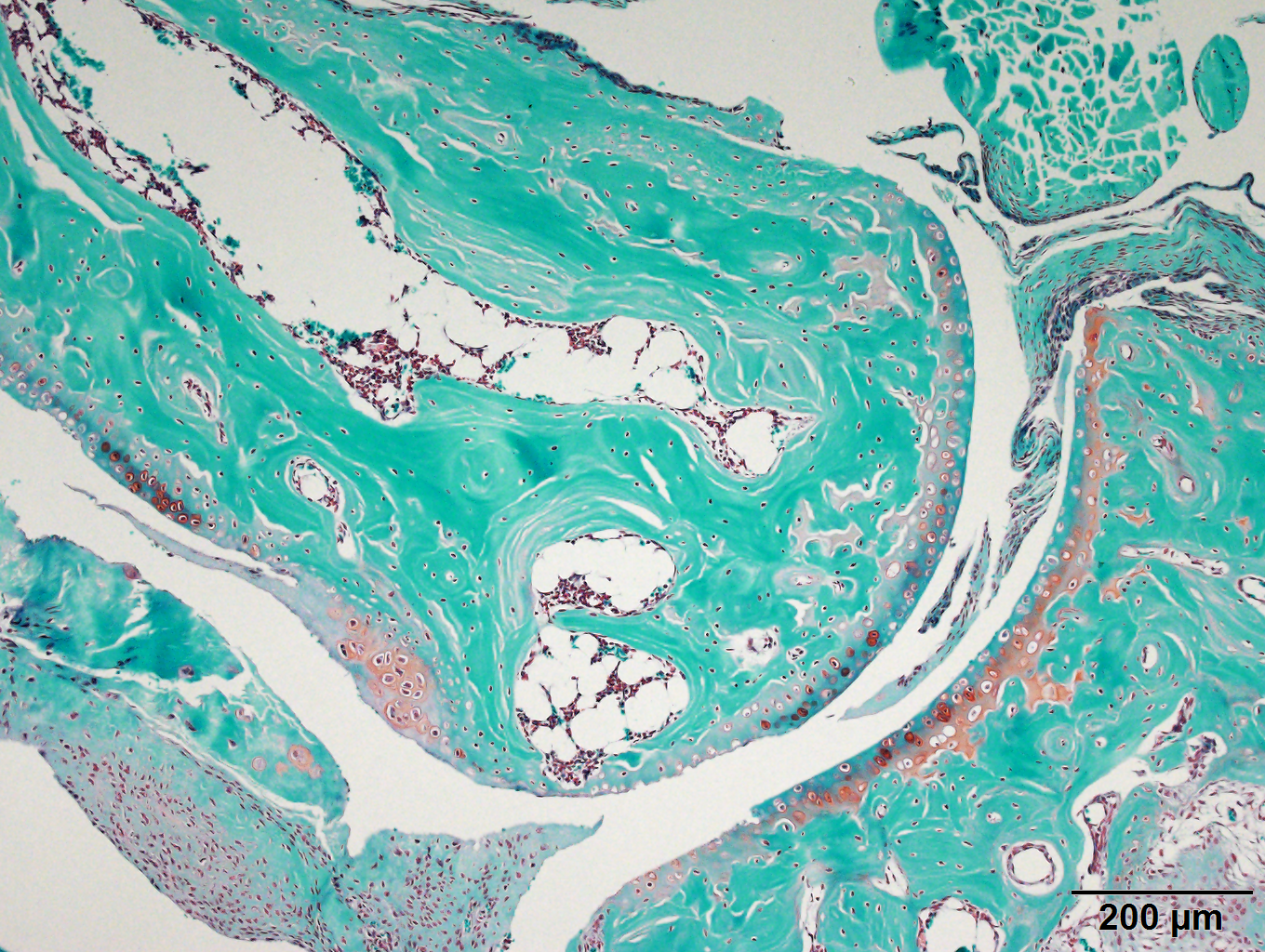

Supplement: Supplementary file 3 [file DataSheet9.ZIP › Fig 6E/CIA+PTXú¿1ú⌐.tif]

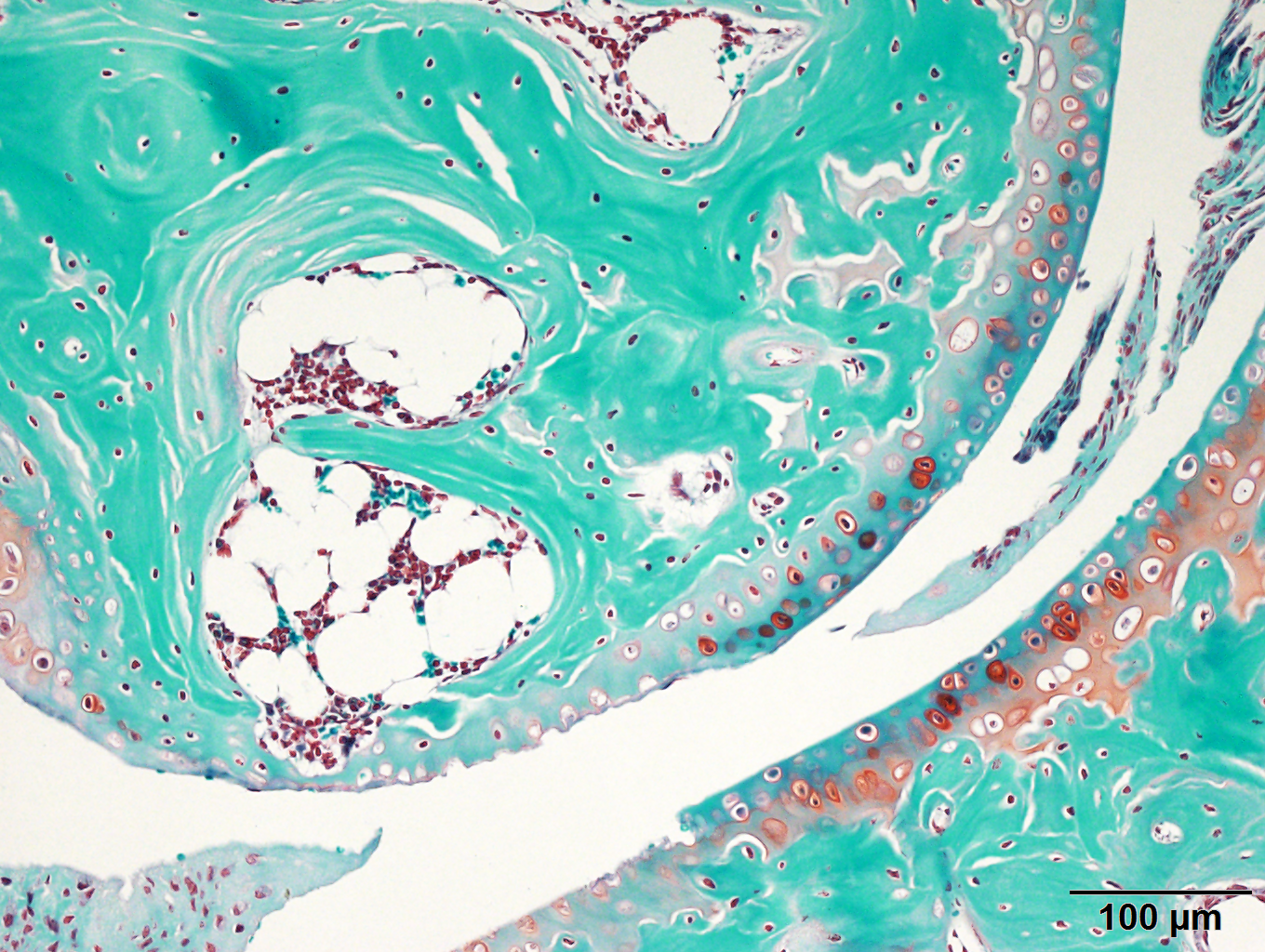

Supplement: Supplementary file 3 [file DataSheet9.ZIP › Fig 6E/CIA+PTXú¿2ú⌐.tif]

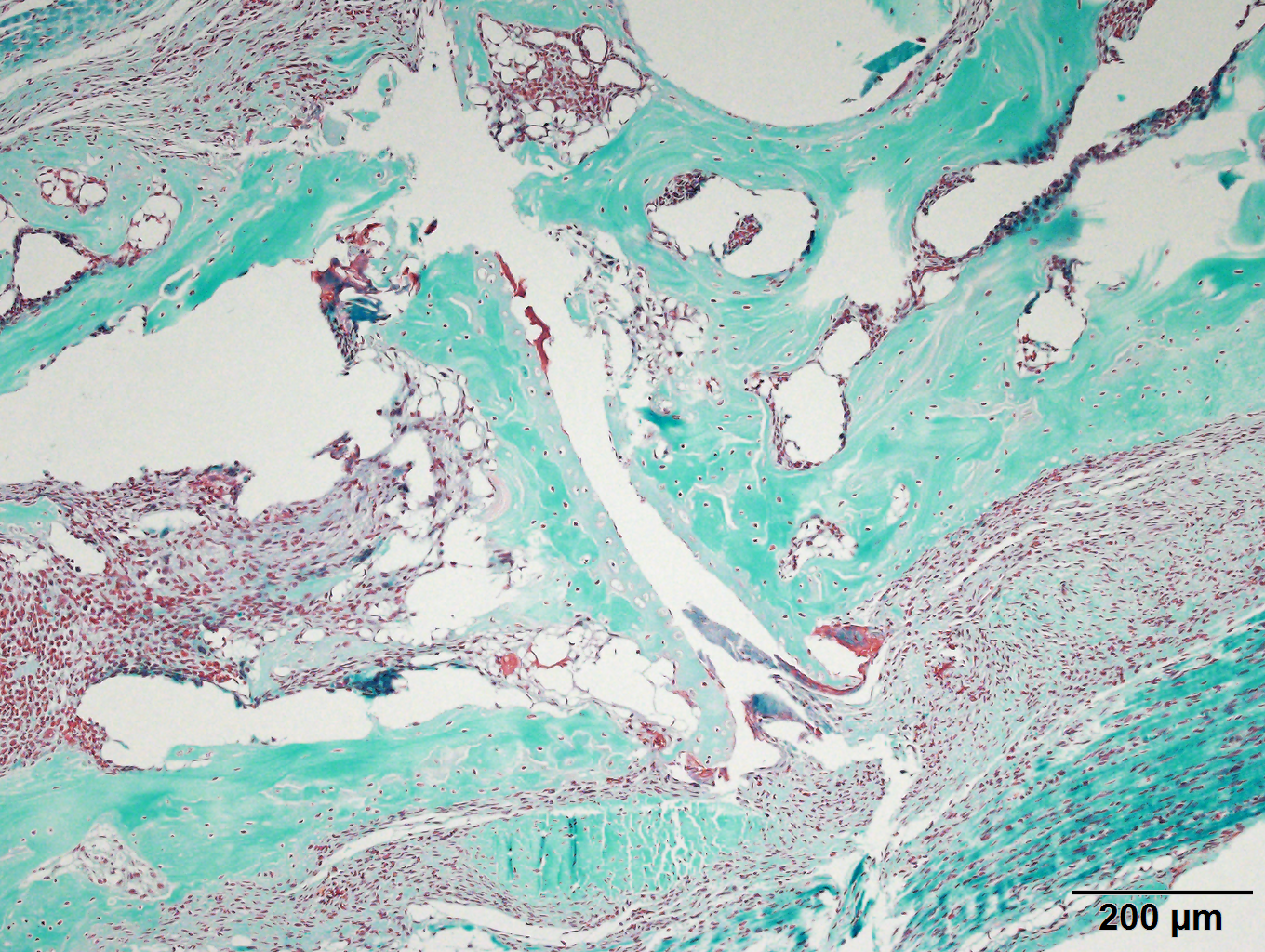

Supplement: Supplementary file 3 [file DataSheet9.ZIP › Fig 6E/CIAú¿1ú⌐.tif]

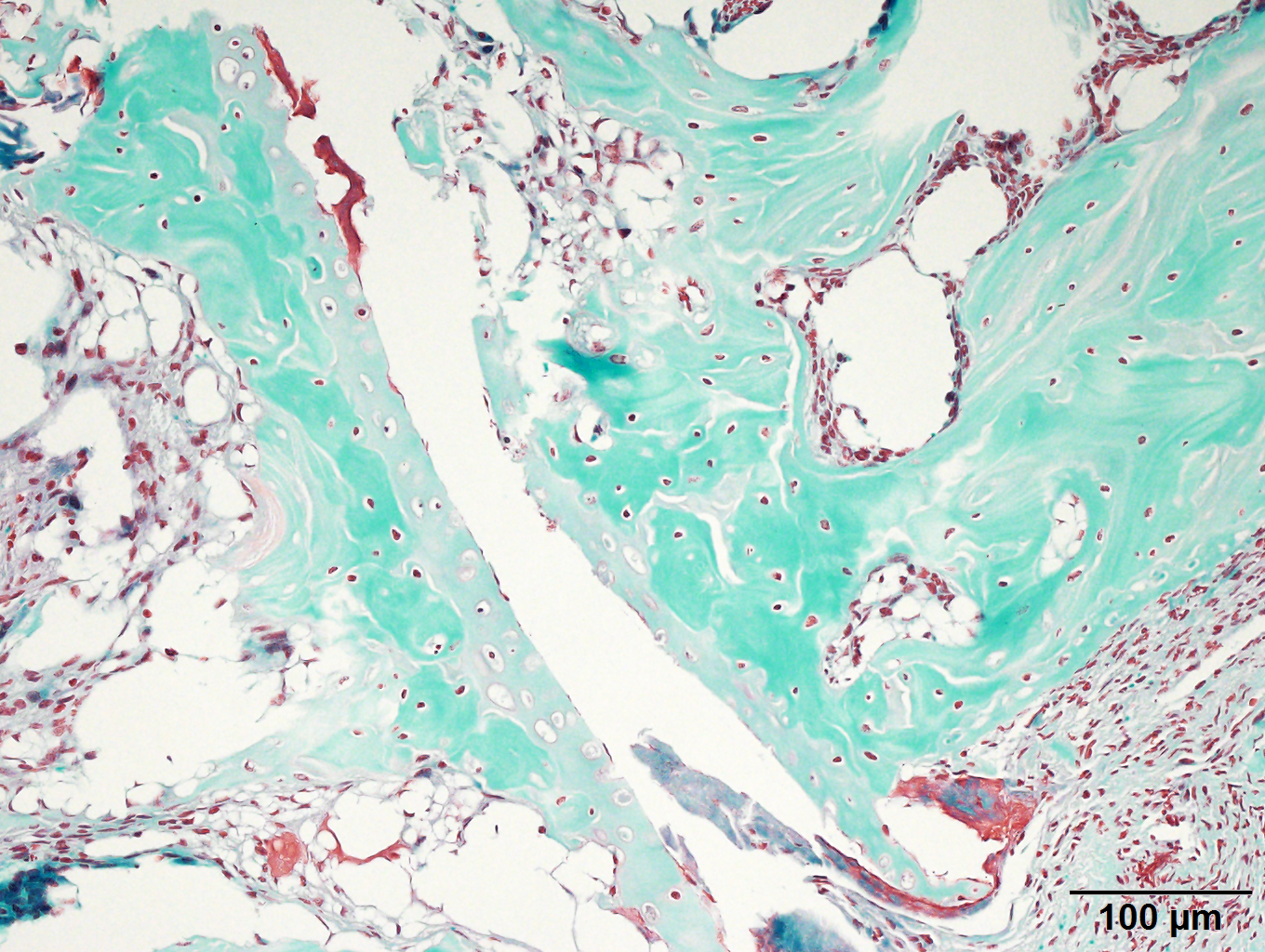

Supplement: Supplementary file 3 [file DataSheet9.ZIP › Fig 6E/CIAú¿2ú⌐.tif]

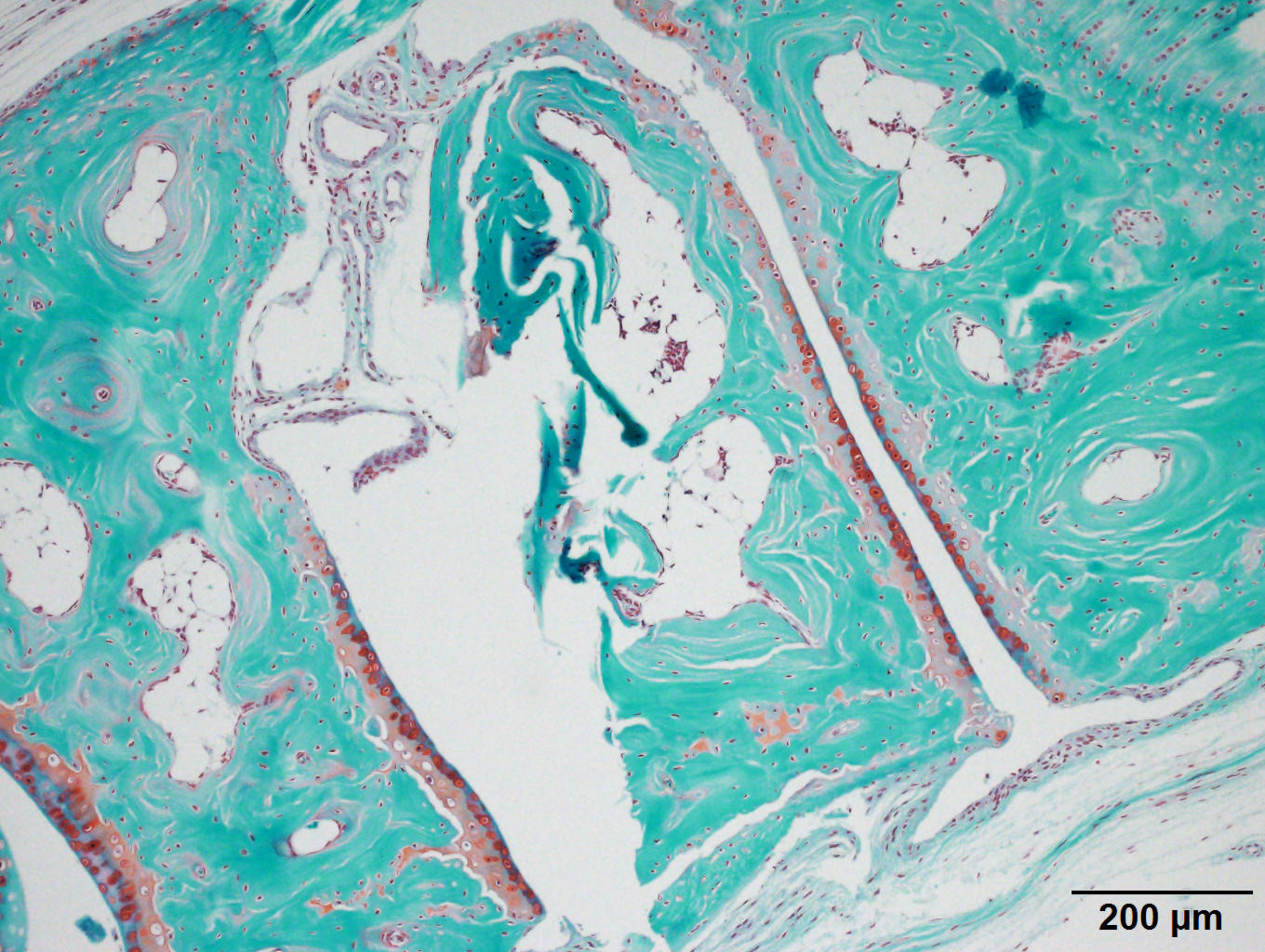

Supplement: Supplementary file 3 [file DataSheet9.ZIP › Fig 6E/Ctrl (1).tif]

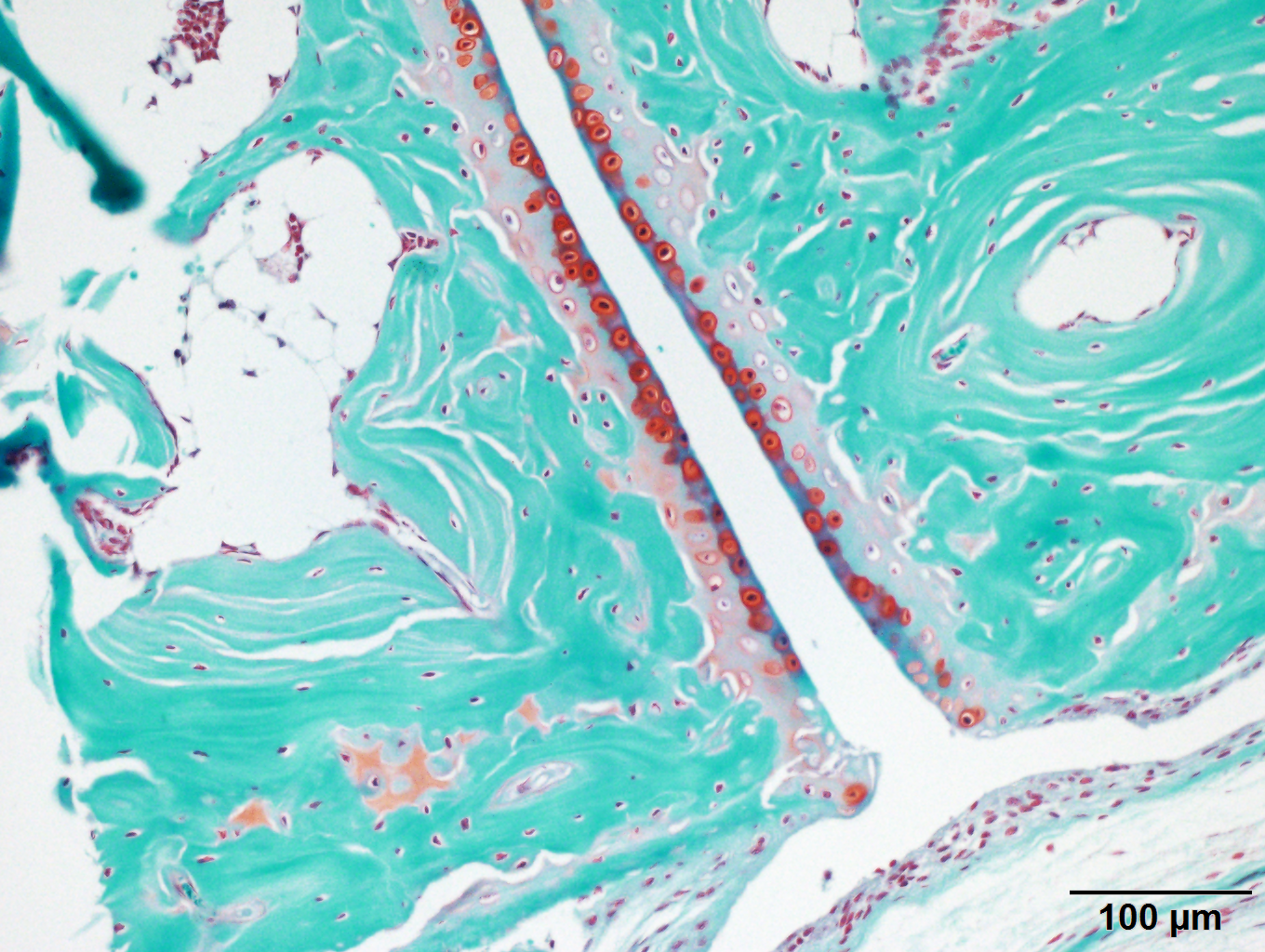

Supplement: Supplementary file 3 [file DataSheet9.ZIP › Fig 6E/Ctrlú¿2ú⌐.tif]

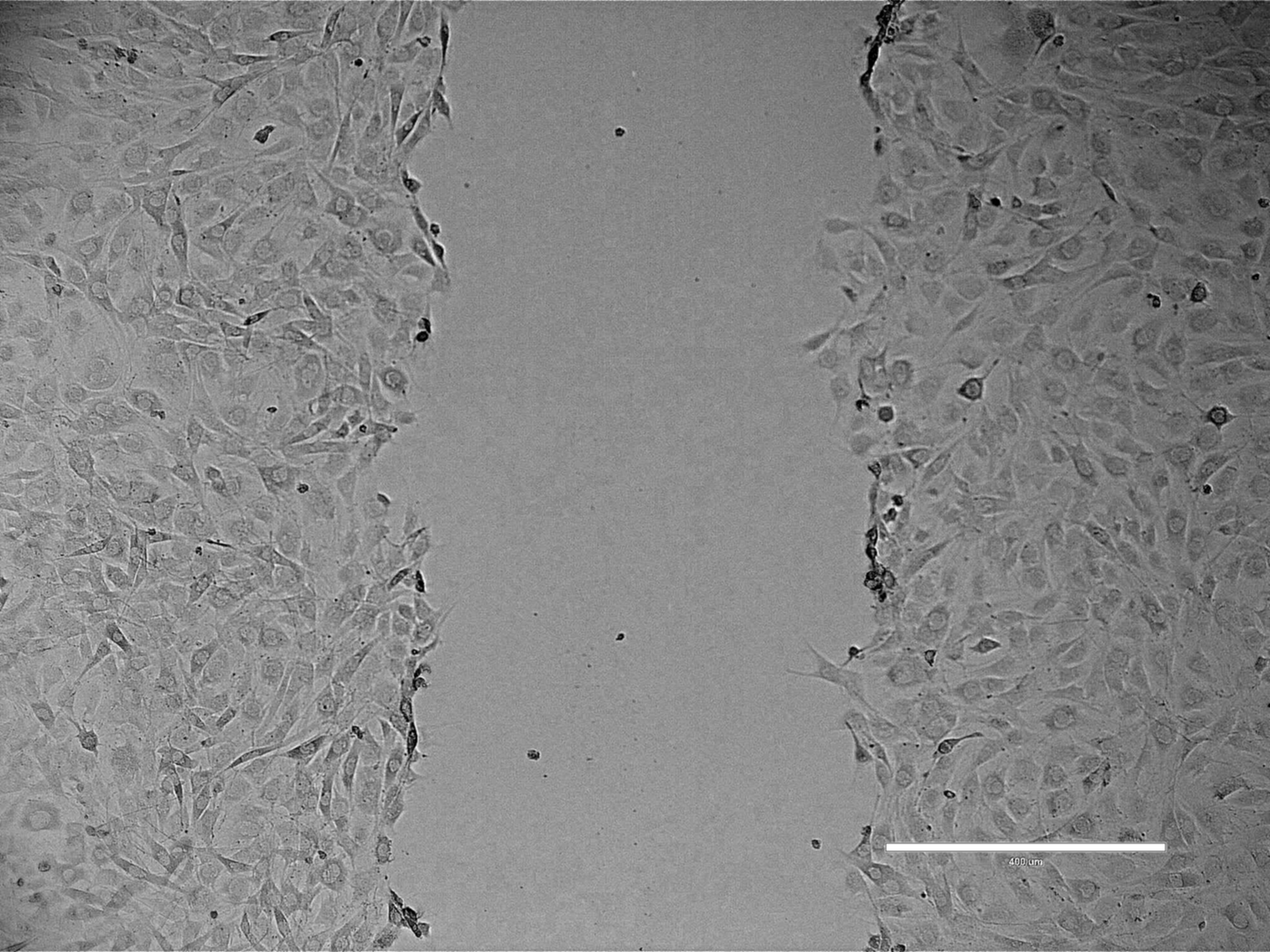

Supplement: Supplementary file 4 [file DataSheet4.ZIP › Fig 1C/Ctrl-0h.tif]

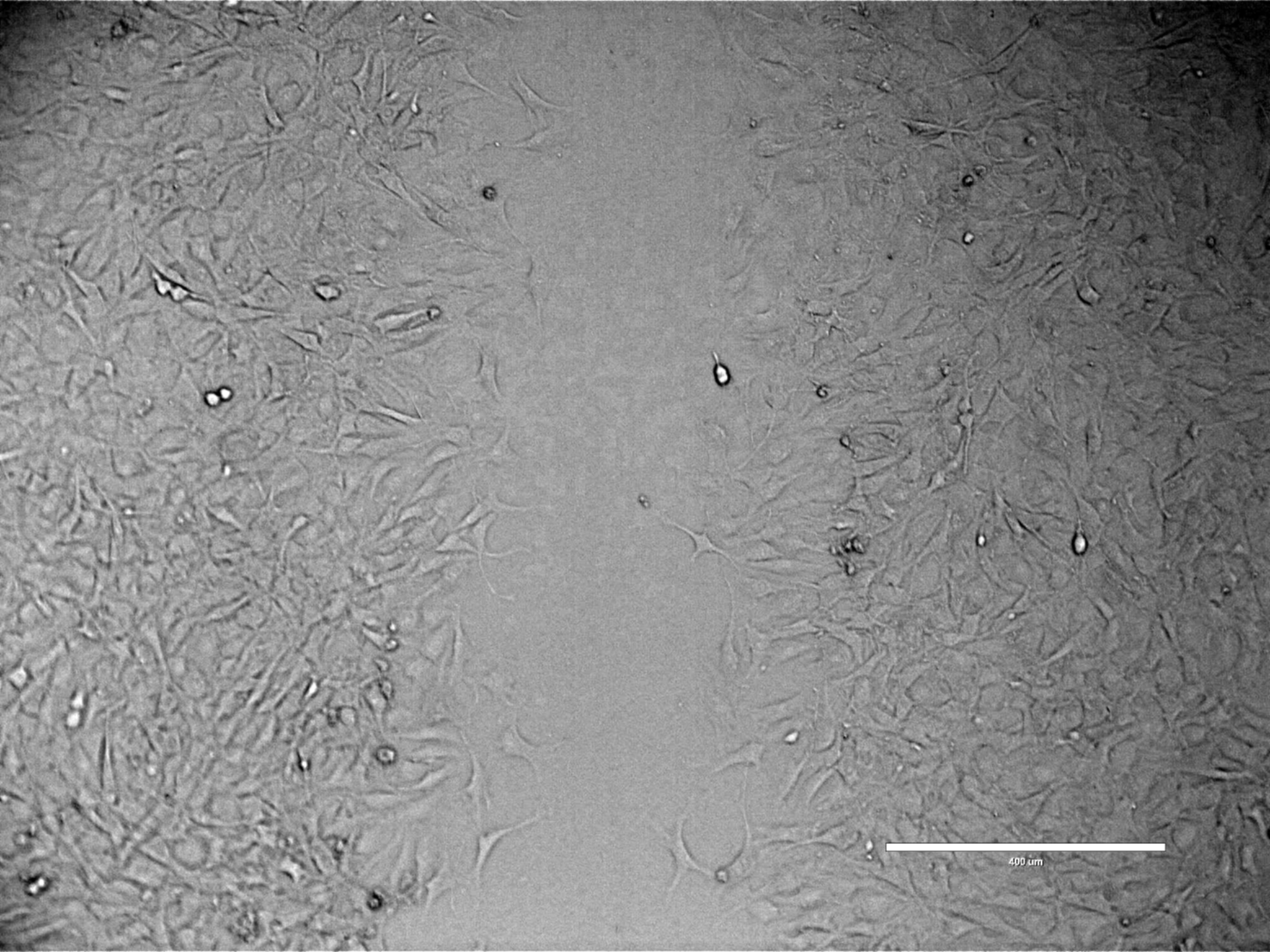

Supplement: Supplementary file 4 [file DataSheet4.ZIP › Fig 1C/Ctrl-12h.tif]

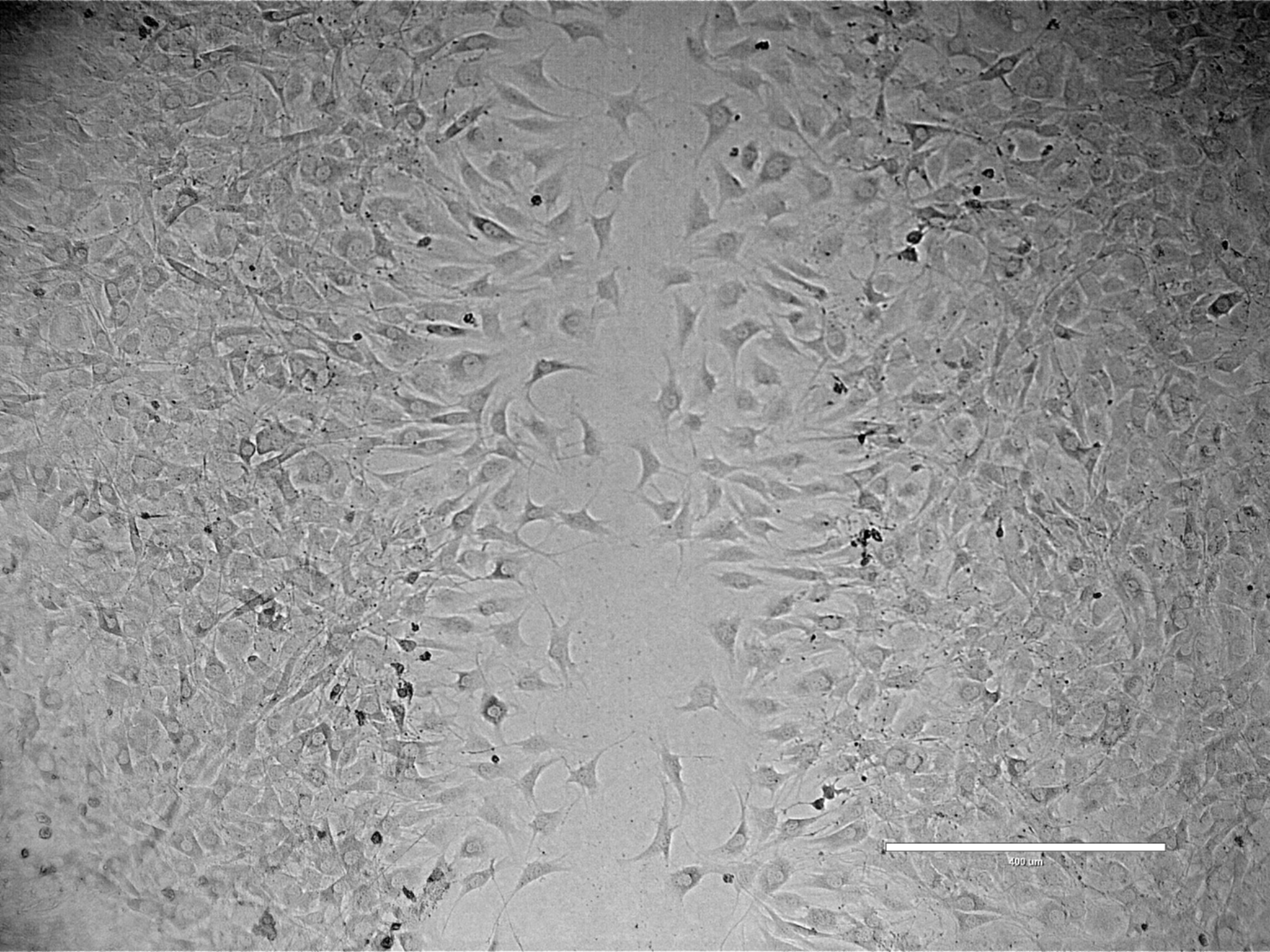

Supplement: Supplementary file 4 [file DataSheet4.ZIP › Fig 1C/Ctrl-24h.tif]

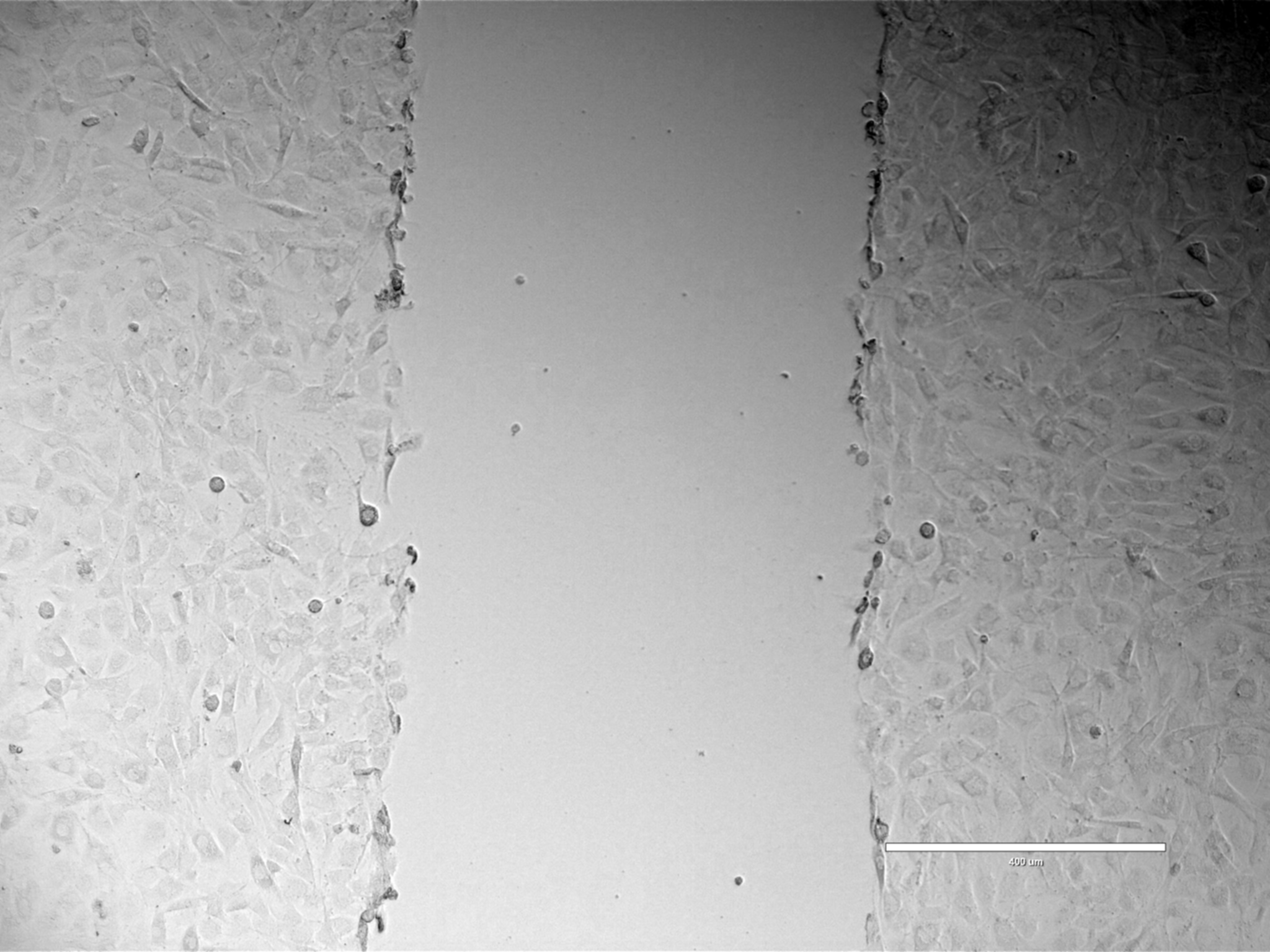

Supplement: Supplementary file 4 [file DataSheet4.ZIP › Fig 1C/PTX 10nM-0h.tif]

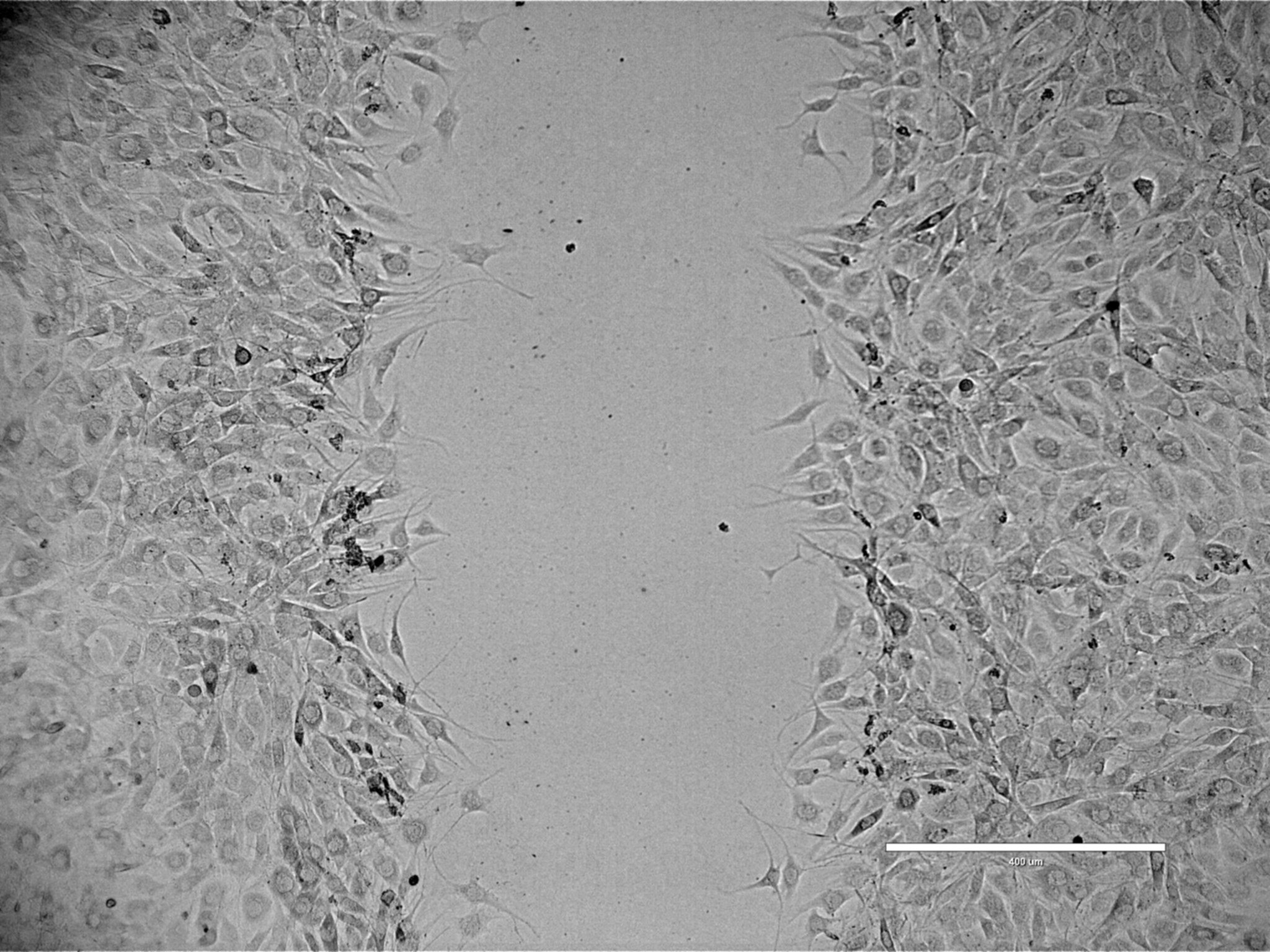

Supplement: Supplementary file 4 [file DataSheet4.ZIP › Fig 1C/PTX 10nM-12h.tif]

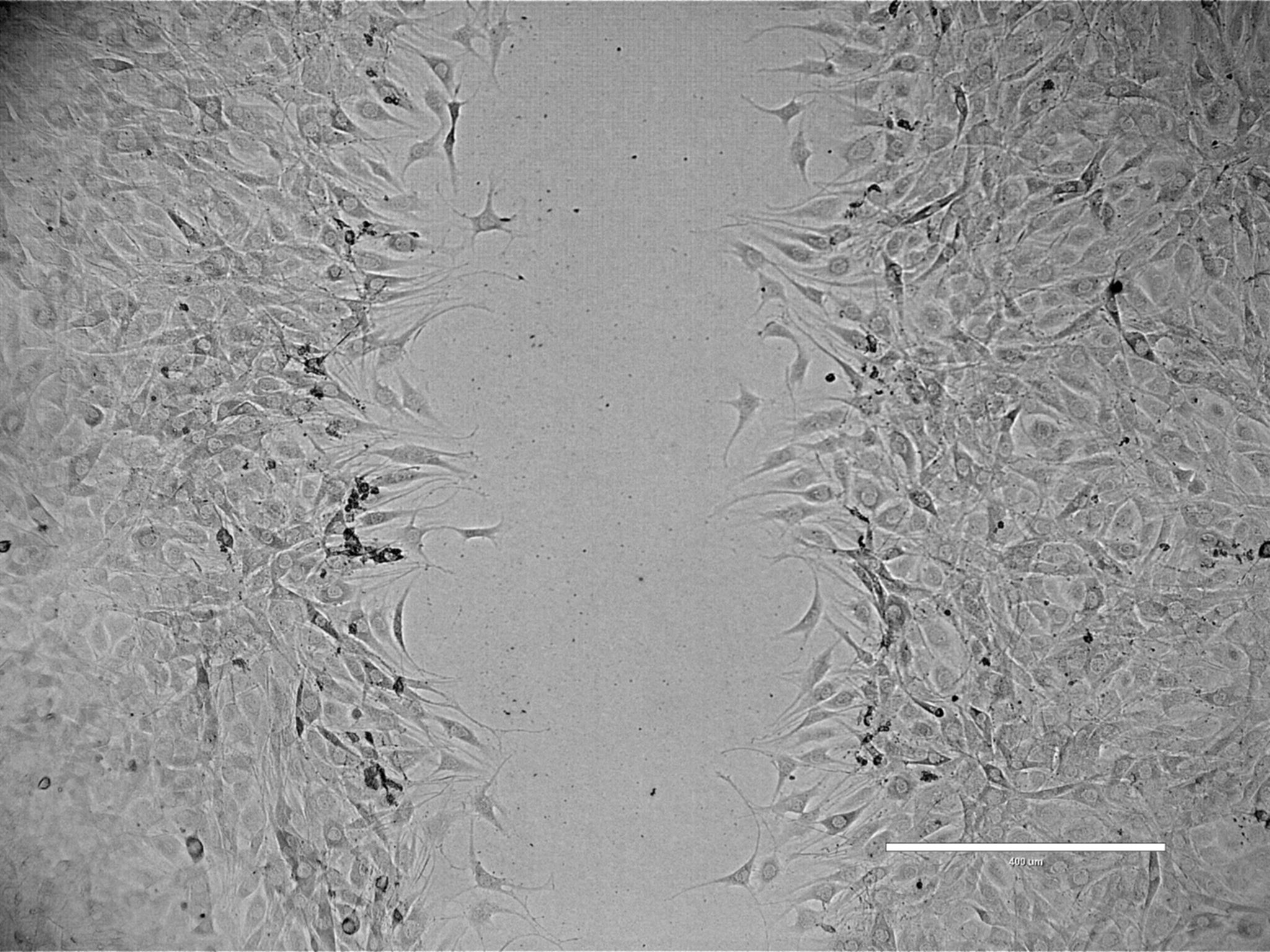

Supplement: Supplementary file 4 [file DataSheet4.ZIP › Fig 1C/PTX 10nM-24h.tif]

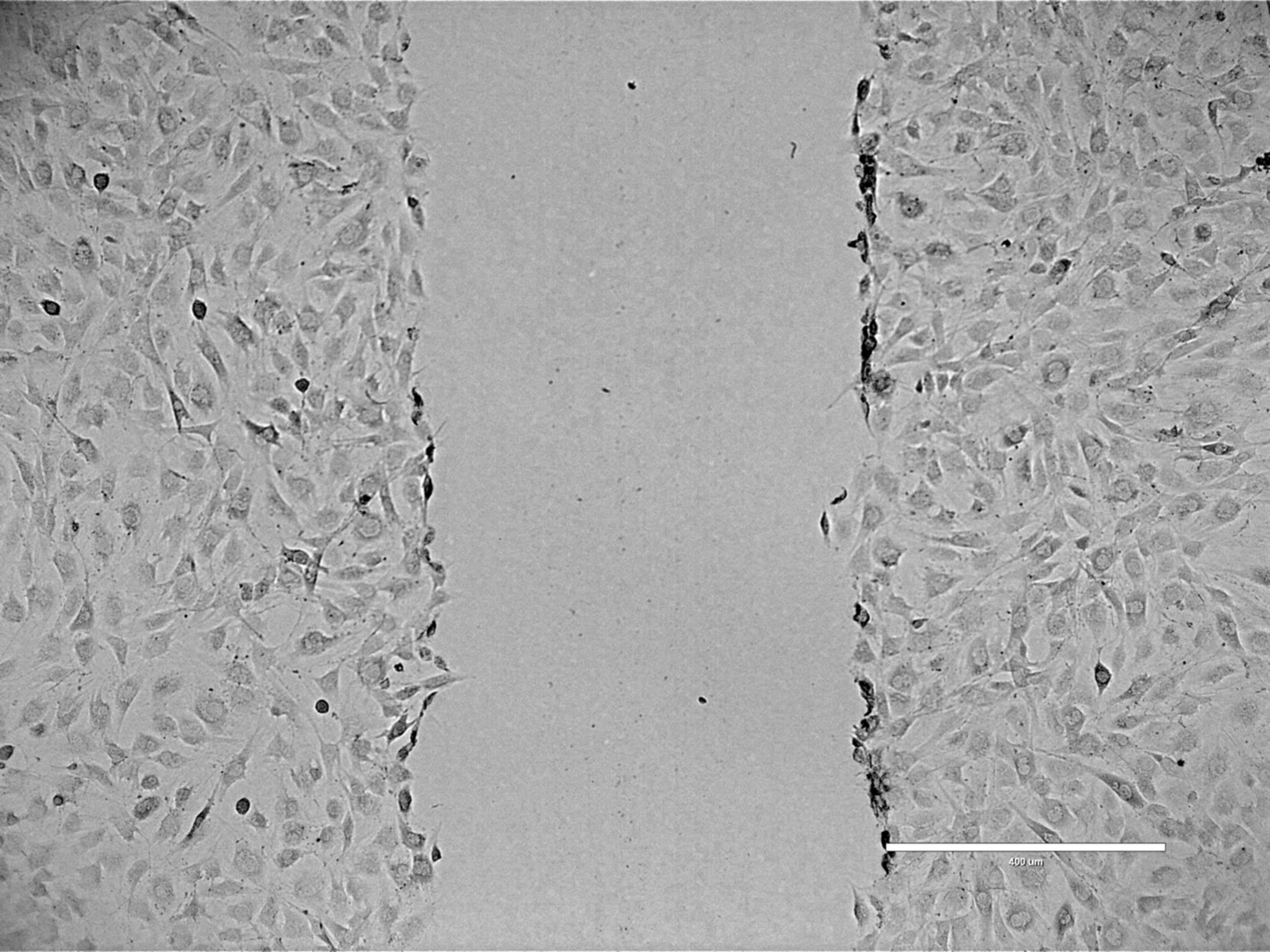

Supplement: Supplementary file 4 [file DataSheet4.ZIP › Fig 1C/PTX 2.5nM-0h.tif]

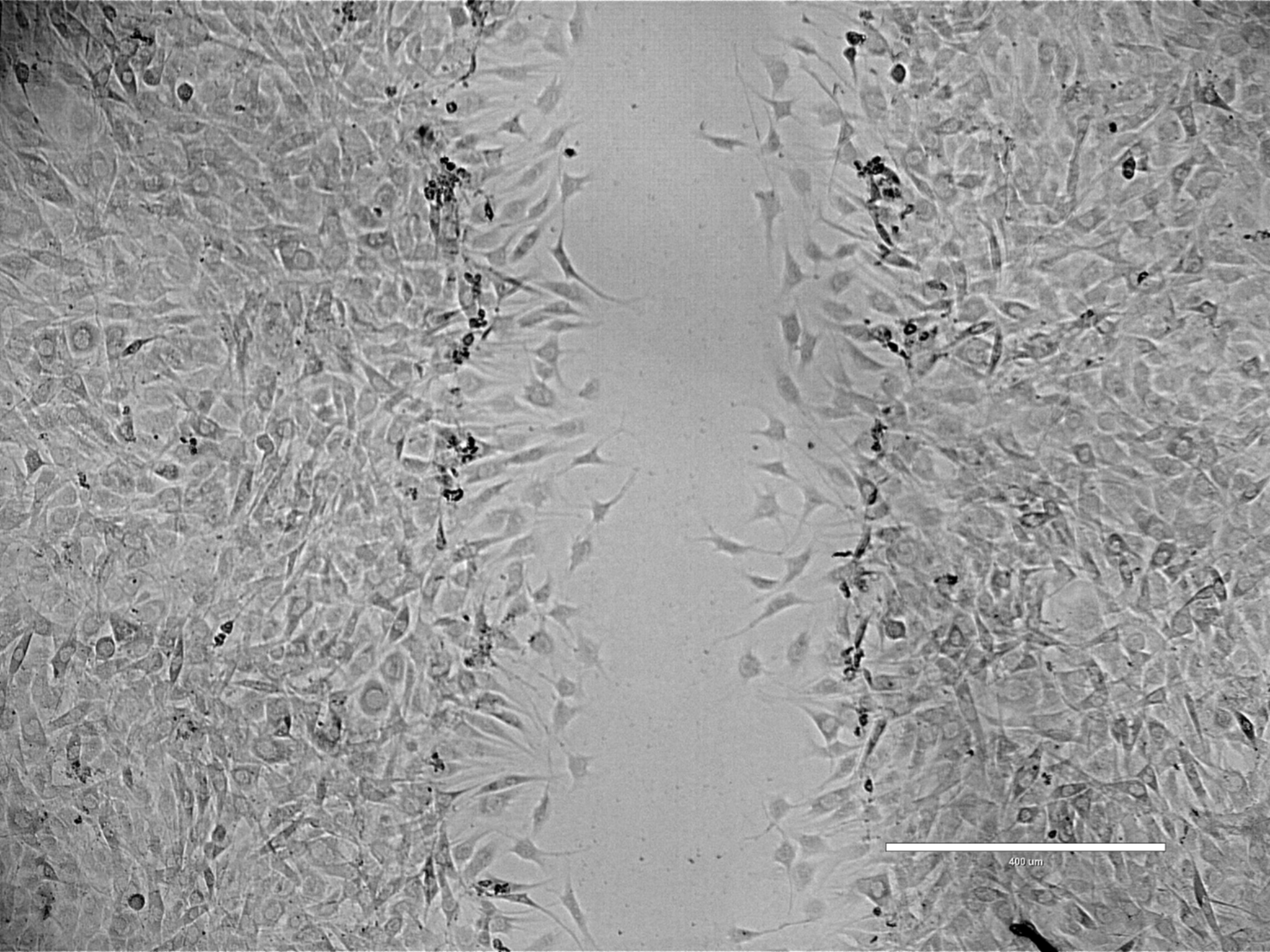

Supplement: Supplementary file 4 [file DataSheet4.ZIP › Fig 1C/PTX 2.5nM-12h.tif]

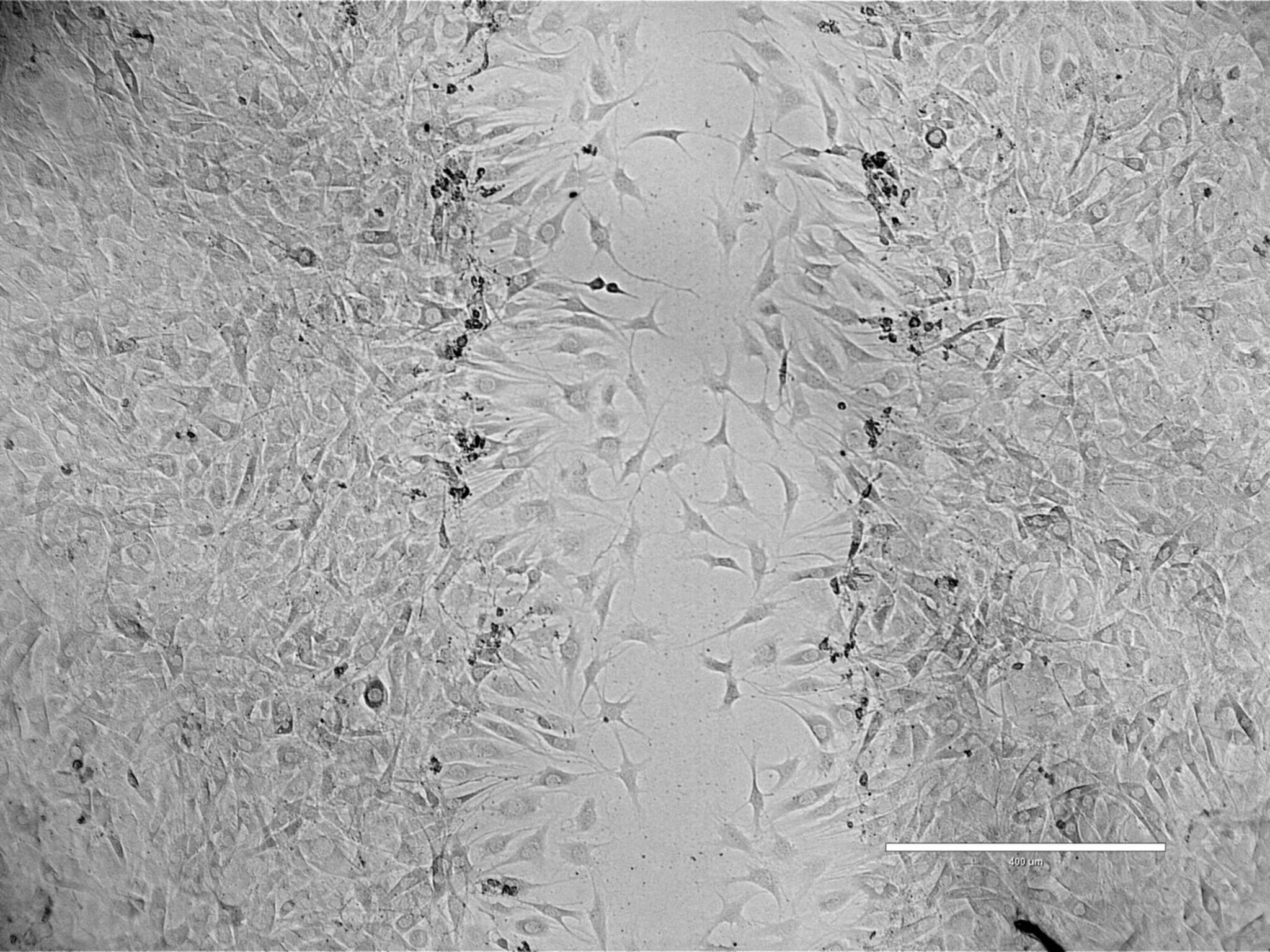

Supplement: Supplementary file 4 [file DataSheet4.ZIP › Fig 1C/PTX 2.5nM-24h.tif]

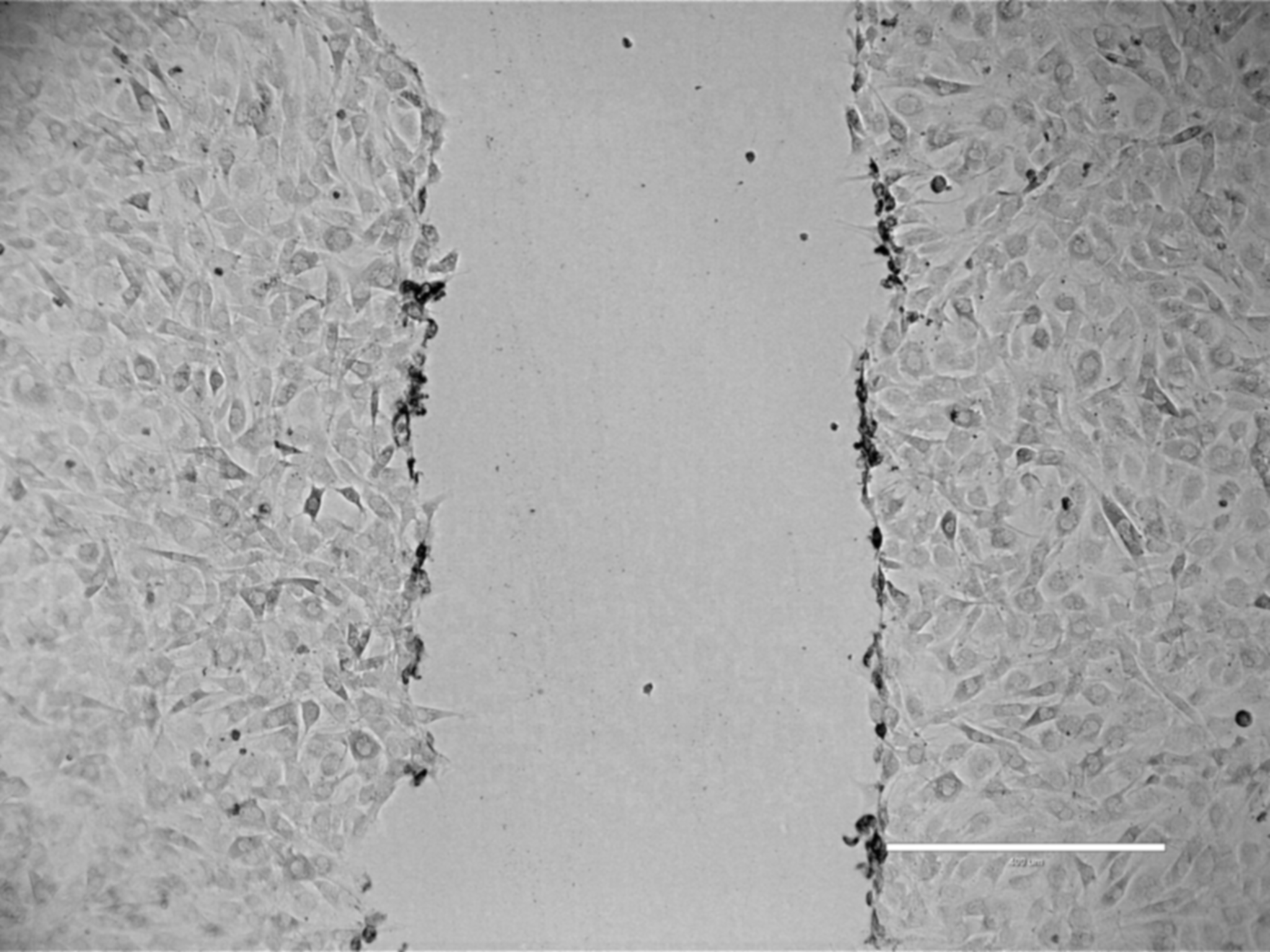

Supplement: Supplementary file 4 [file DataSheet4.ZIP › Fig 1C/PTX 5nM-0h.tif]

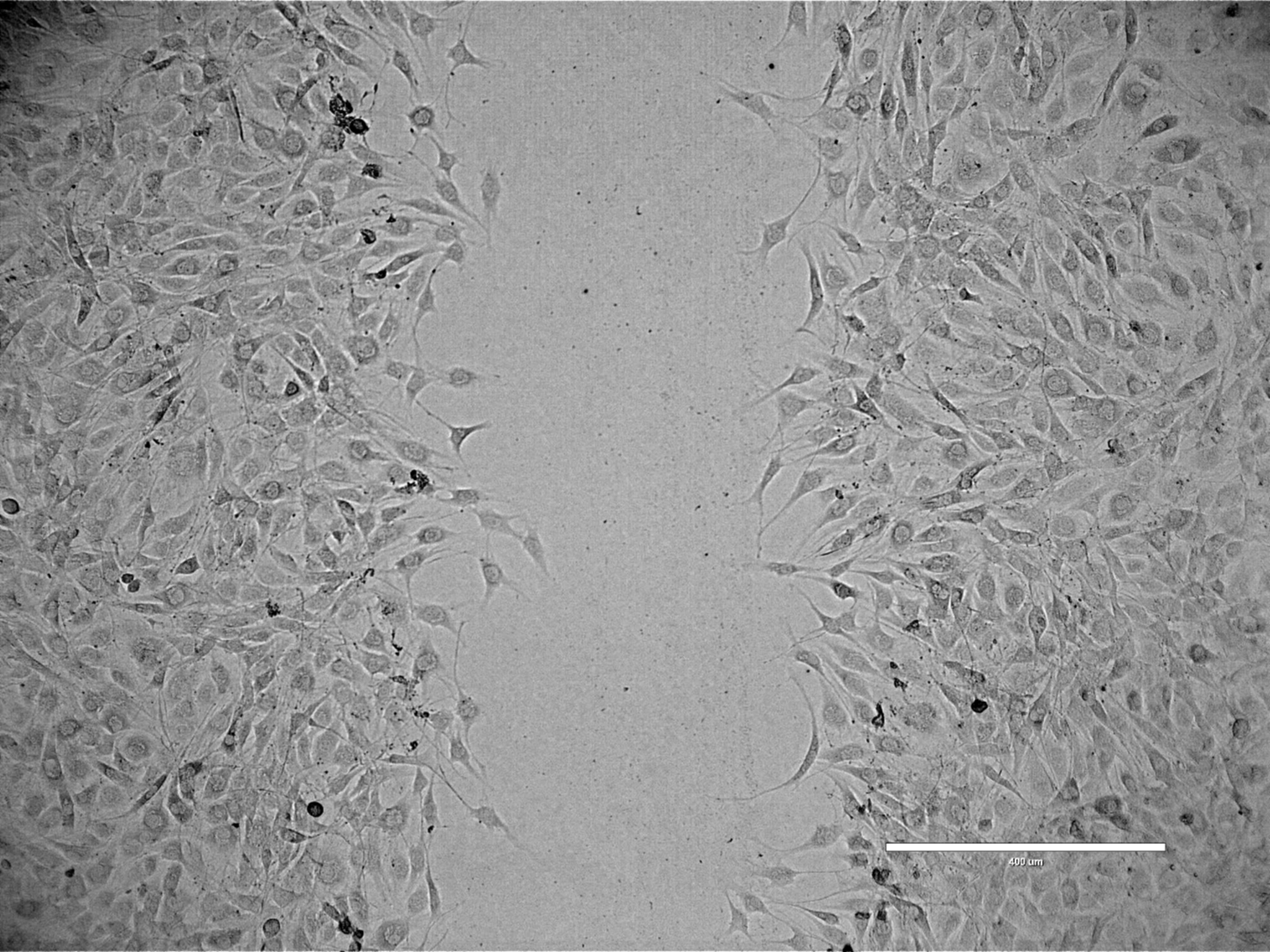

Supplement: Supplementary file 4 [file DataSheet4.ZIP › Fig 1C/PTX 5nM-12h.tif]

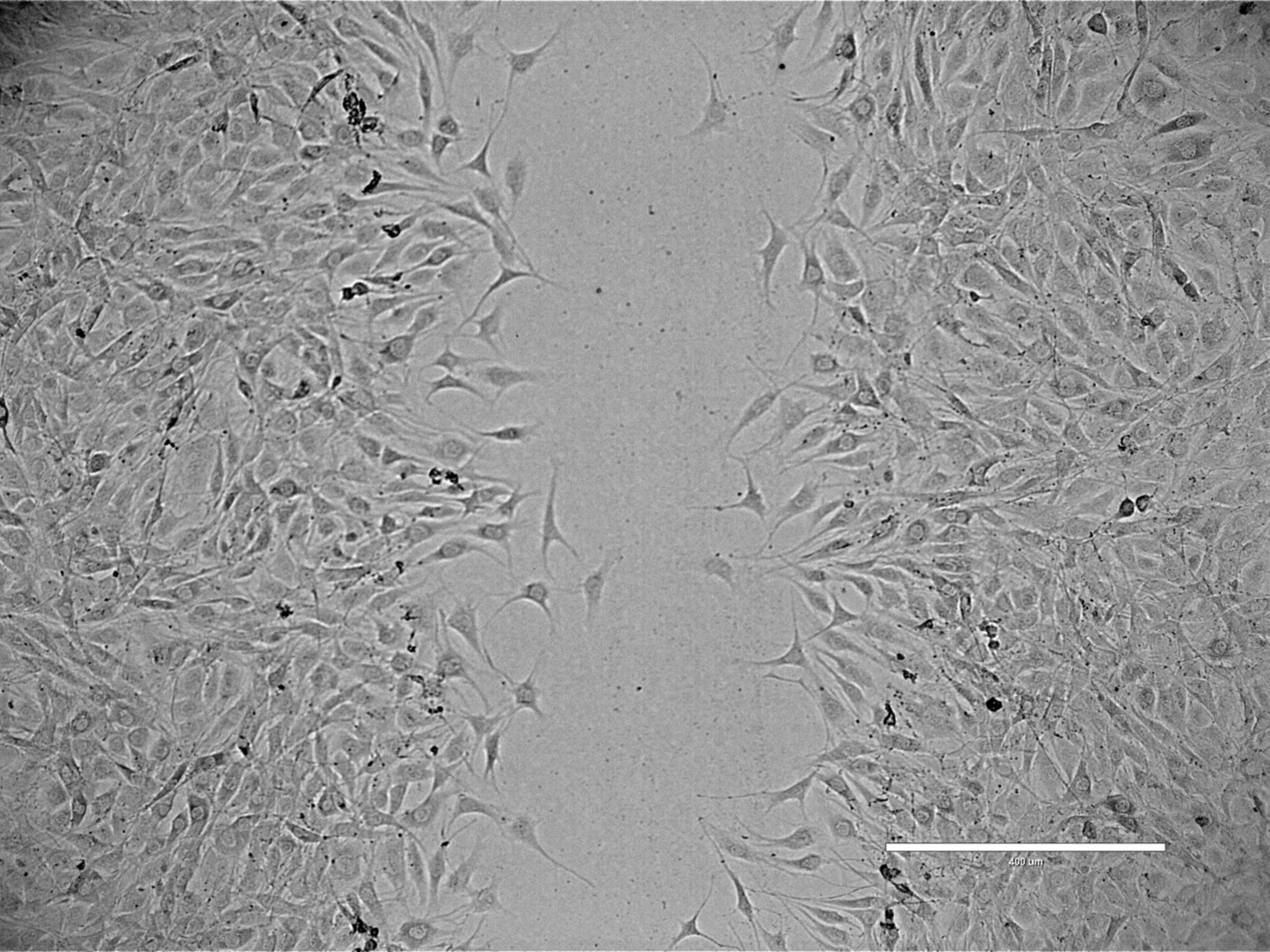

Supplement: Supplementary file 4 [file DataSheet4.ZIP › Fig 1C/PTX 5nM-24h.tif]

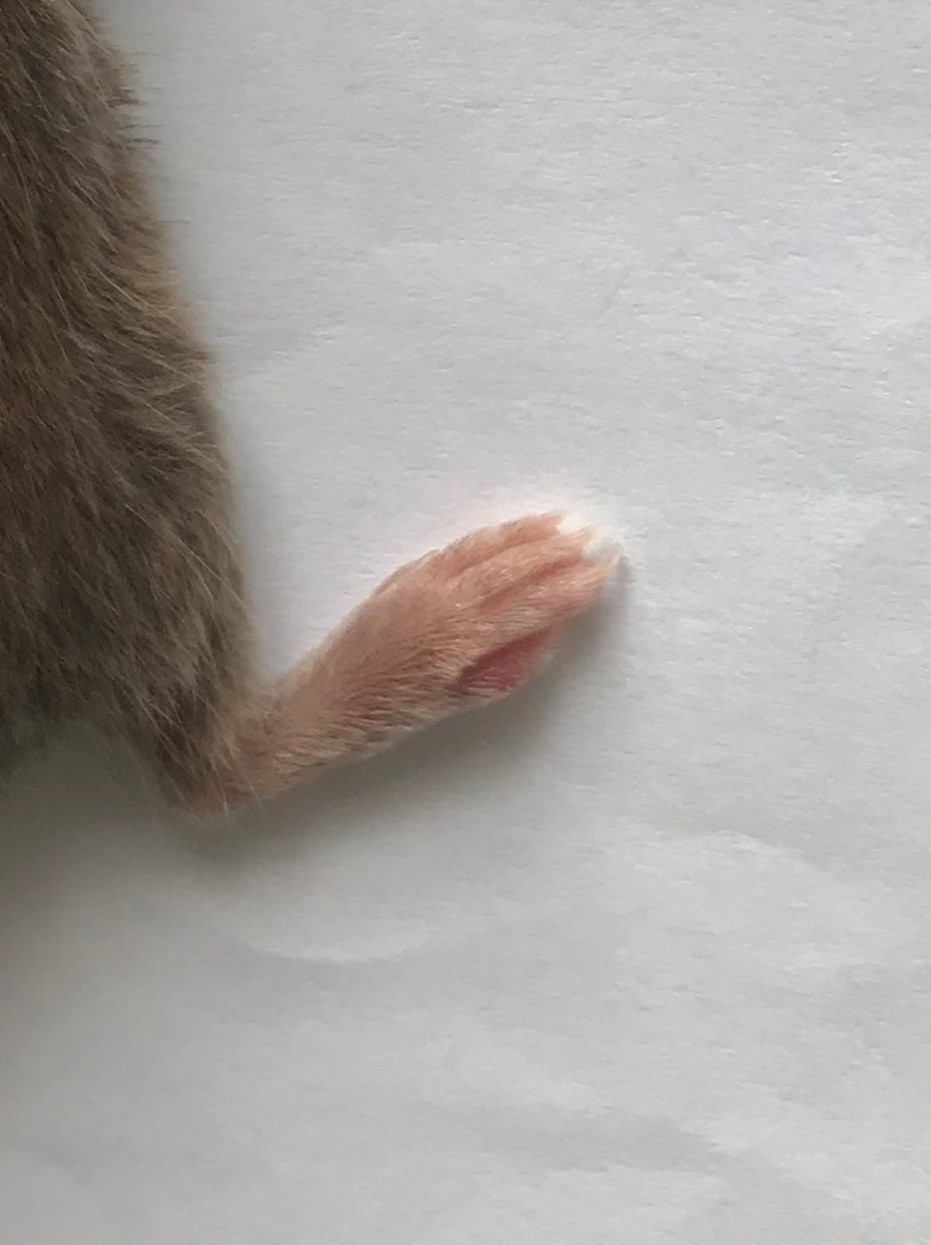

Supplement: Supplementary file 6 [file DataSheet6.ZIP › Fig 5A/CIA+PTX.jpg]

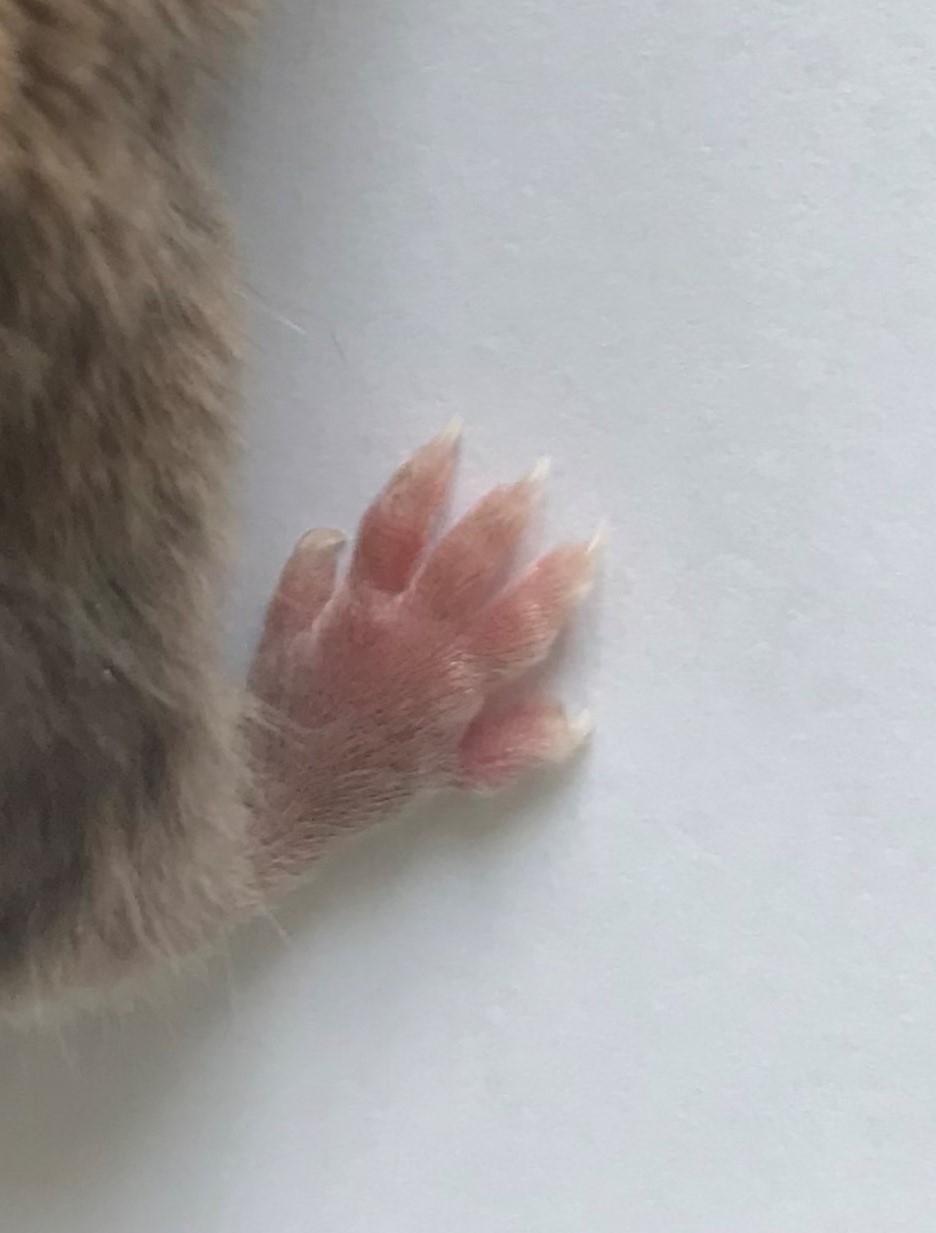

Supplement: Supplementary file 6 [file DataSheet6.ZIP › Fig 5A/CIA.jpg]

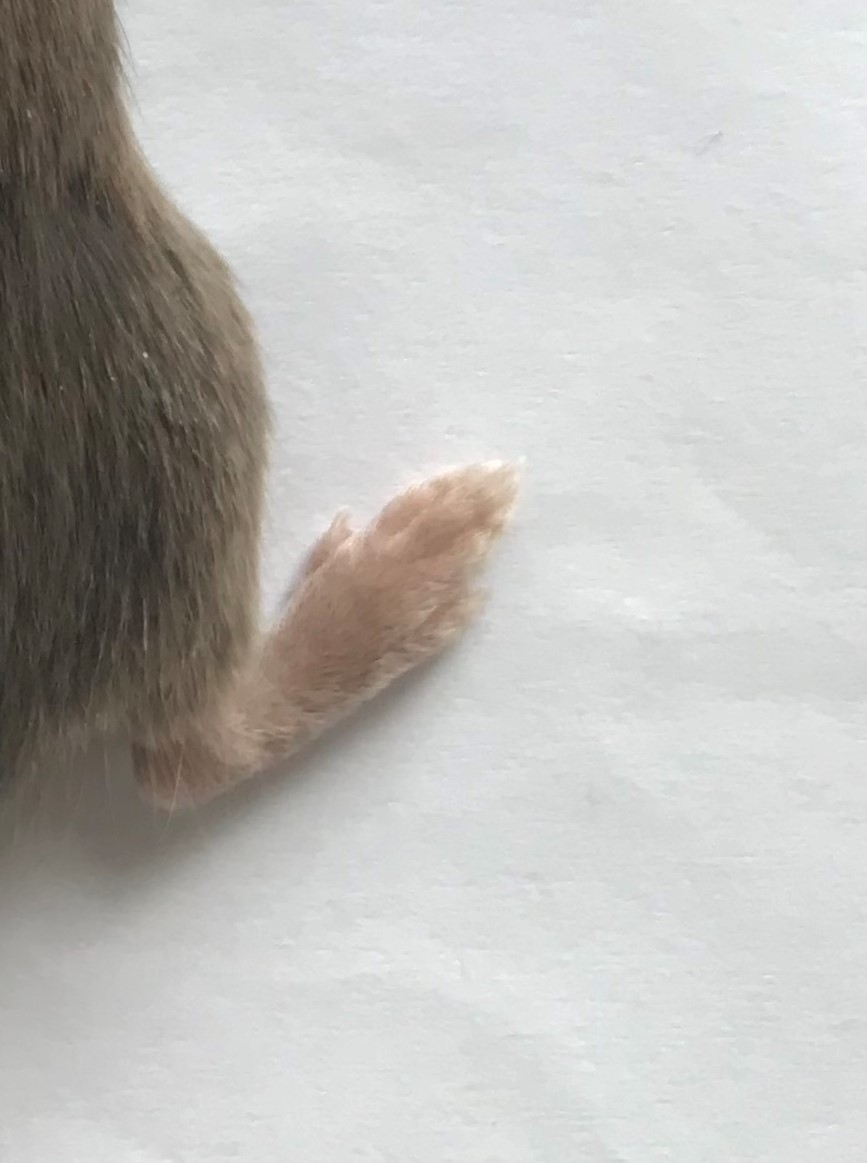

Supplement: Supplementary file 6 [file DataSheet6.ZIP › Fig 5A/Ctrl.jpg]

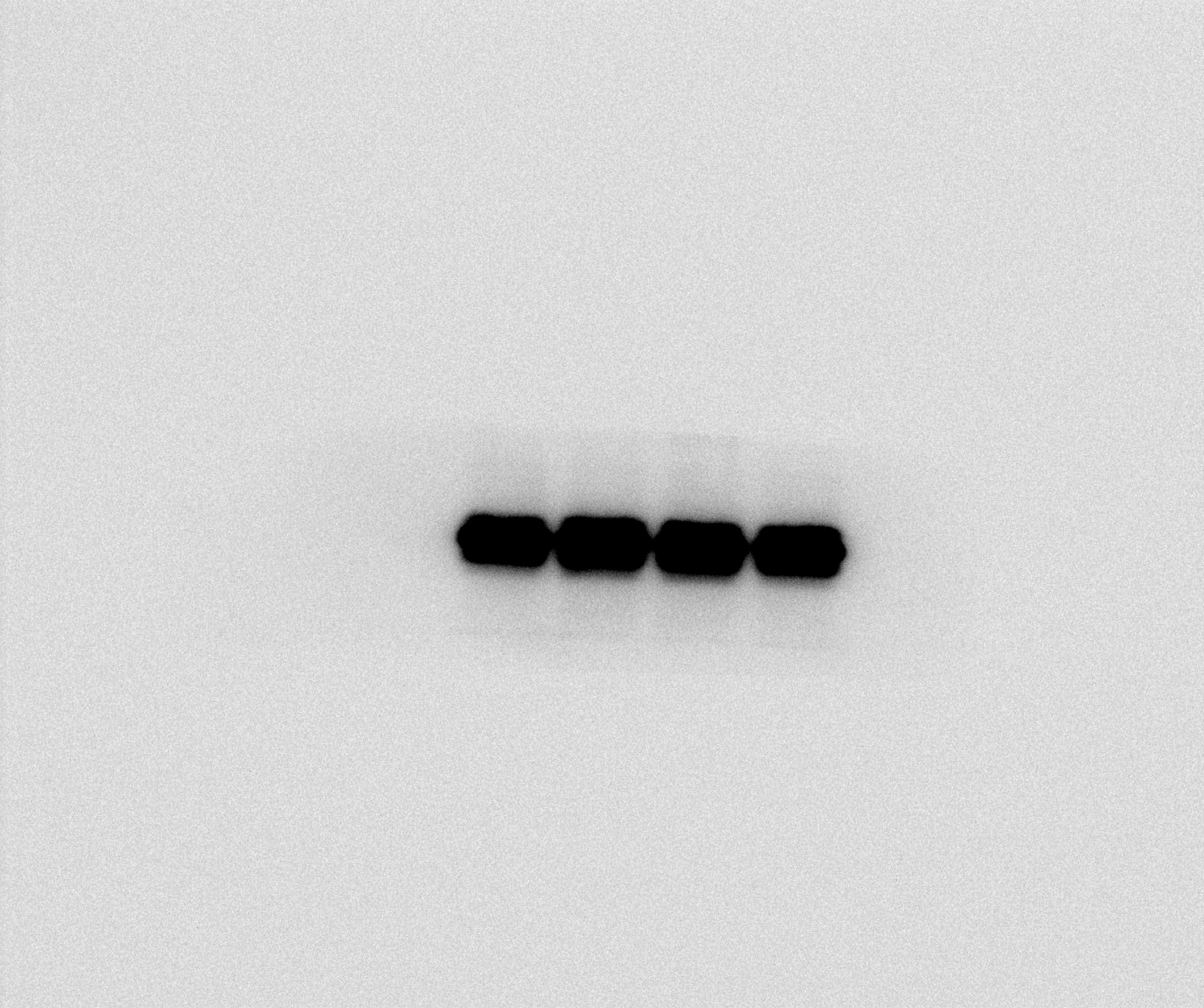

Supplement: Supplementary file 7 [file DataSheet2.ZIP › 4EBP1/4EBP1-F-2.jpg]

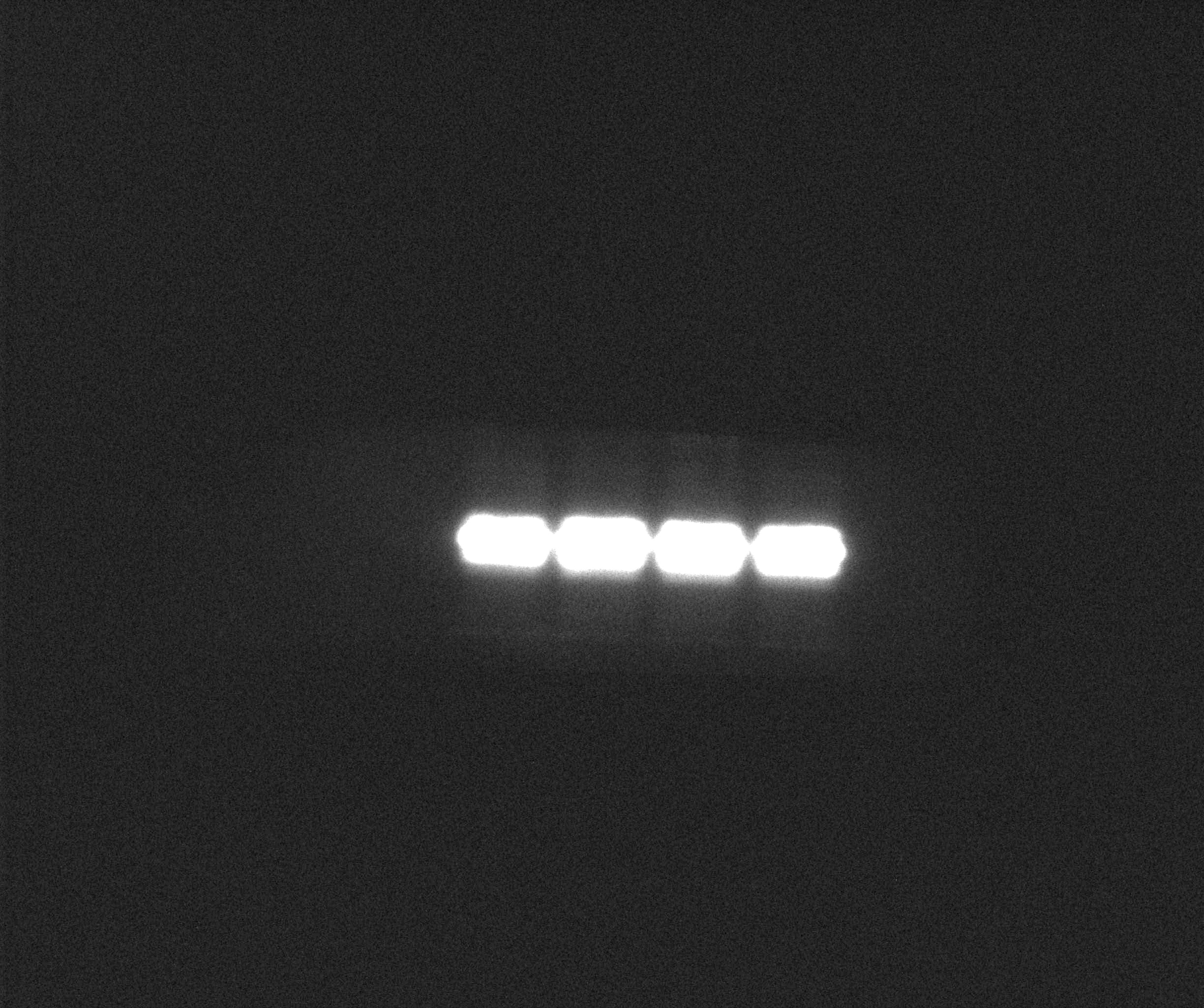

Supplement: Supplementary file 7 [file DataSheet2.ZIP › 4EBP1/4EBP1-F.jpg]

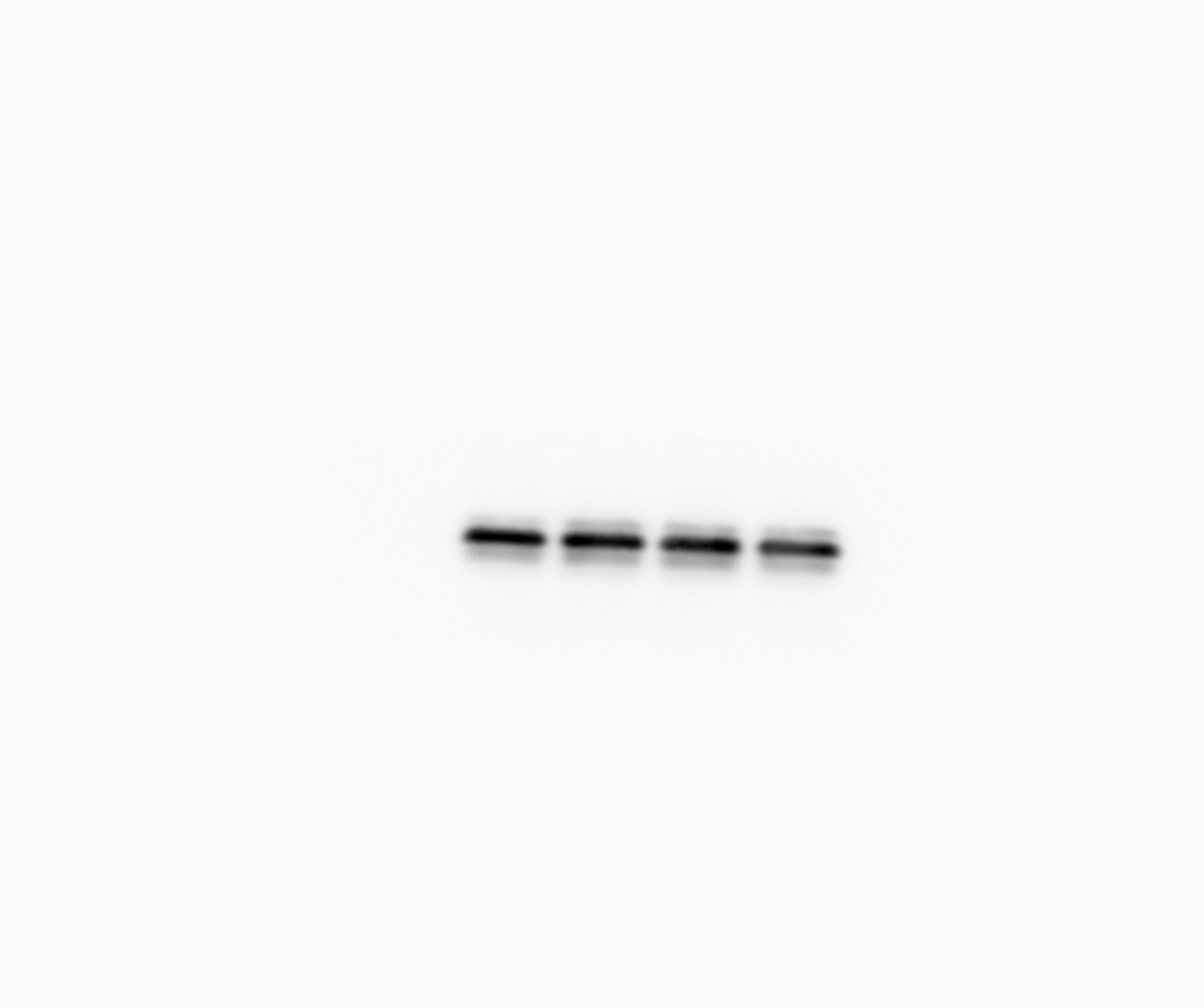

Supplement: Supplementary file 7 [file DataSheet2.ZIP › 4EBP1/4EBP1.jpg]

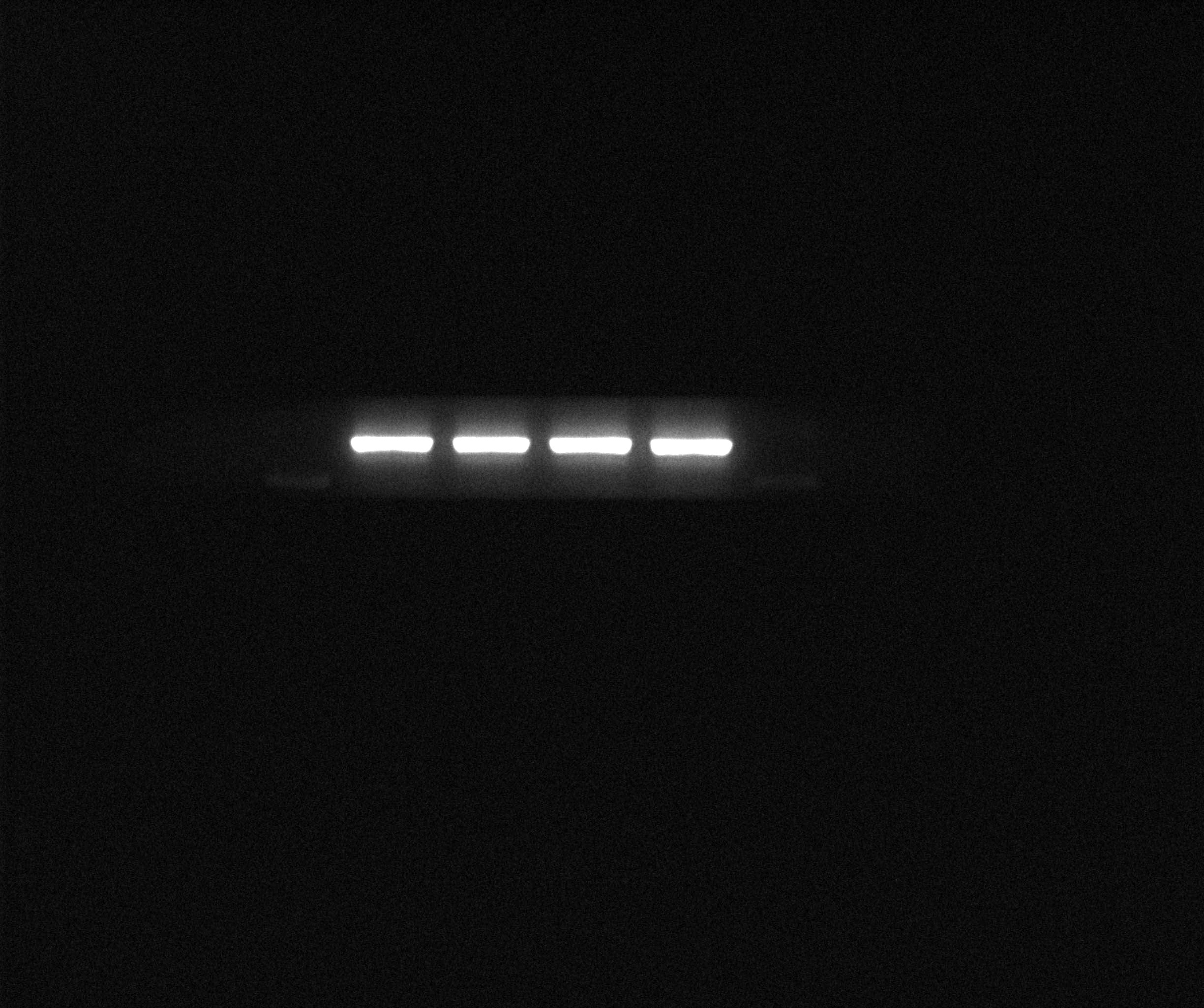

Supplement: Supplementary file 7 [file DataSheet2.ZIP › AKT/AKT-1-B.jpg]

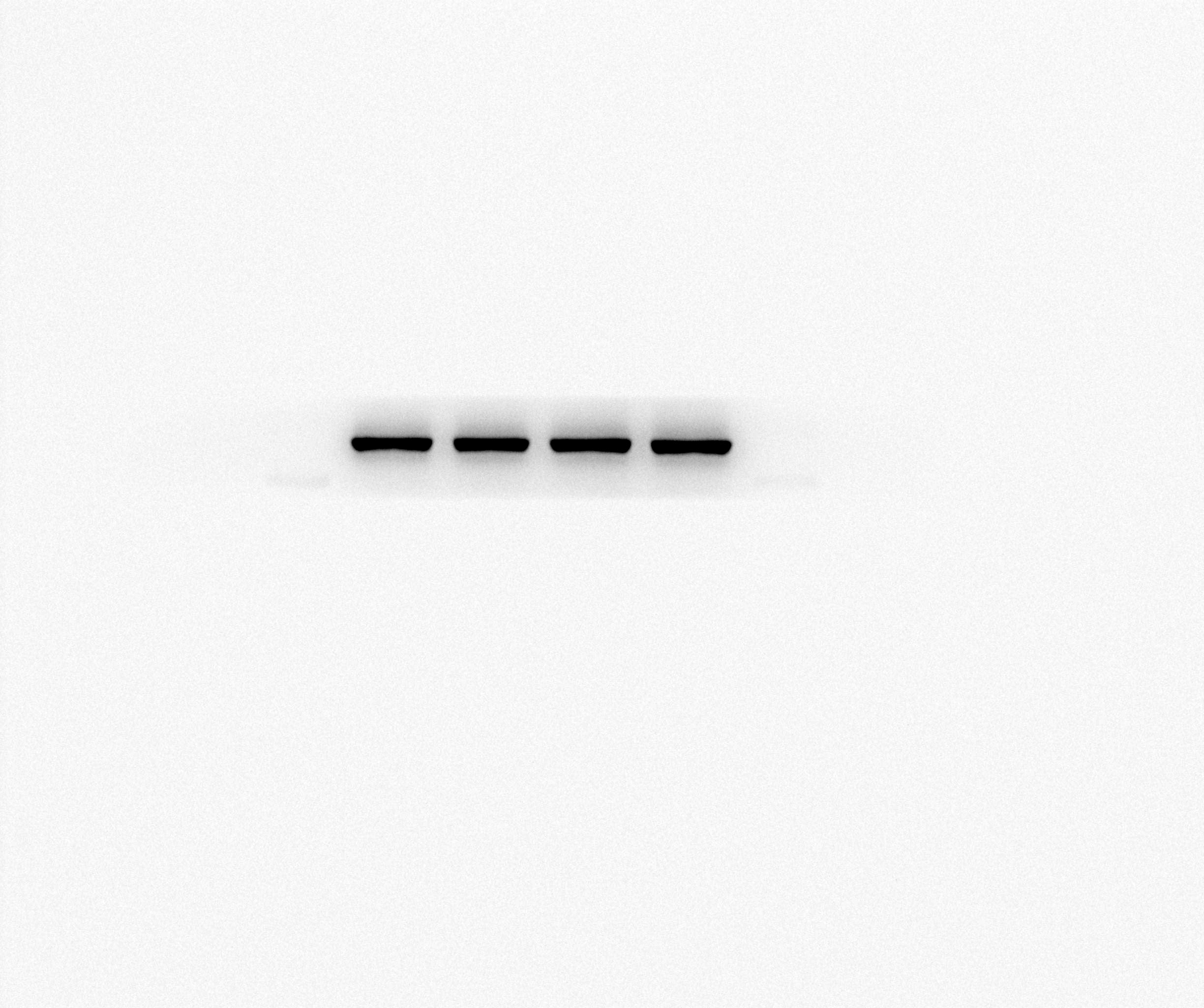

Supplement: Supplementary file 7 [file DataSheet2.ZIP › AKT/AKT-1-F-2.jpg]

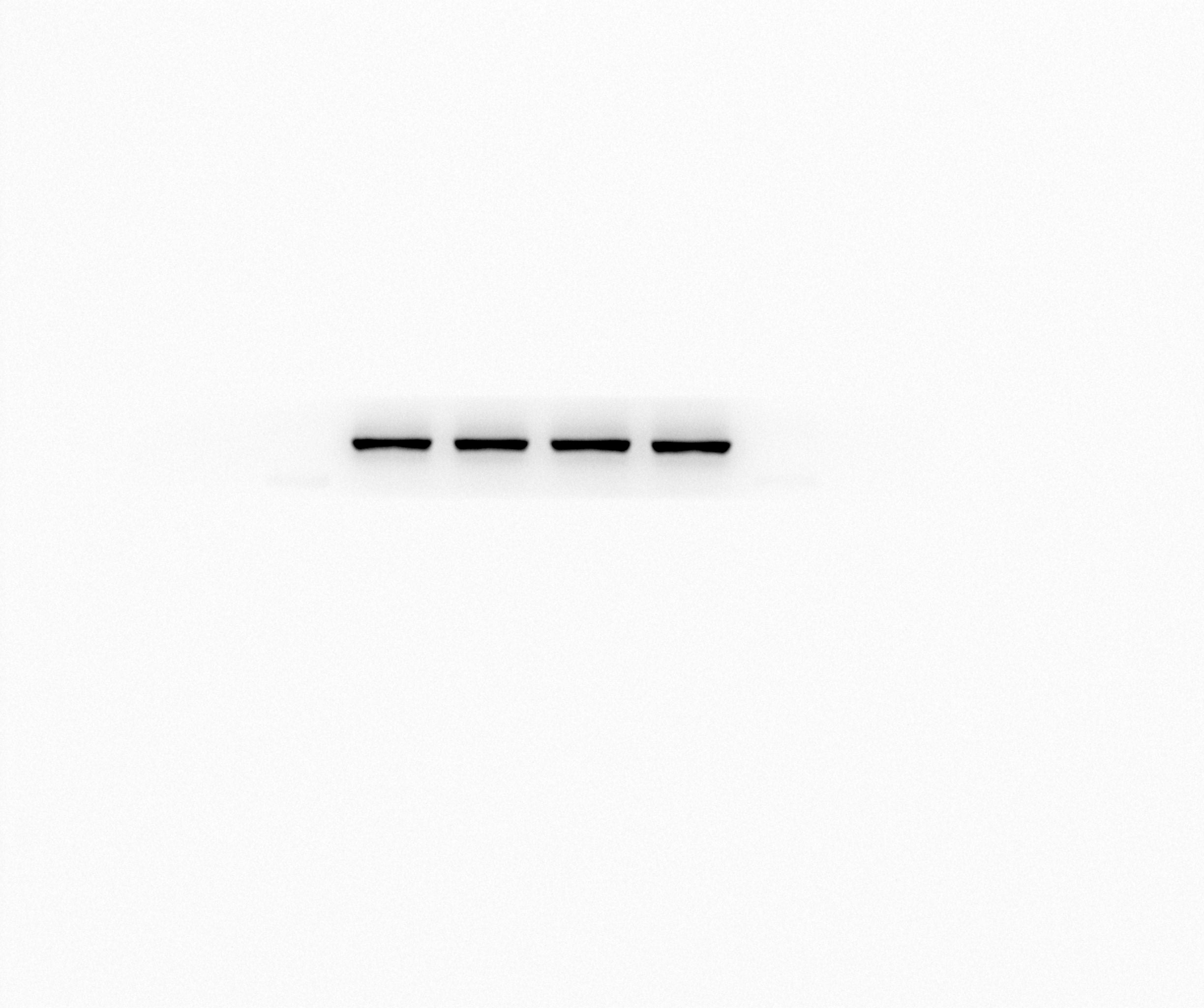

Supplement: Supplementary file 7 [file DataSheet2.ZIP › AKT/AKT-1-F.jpg]

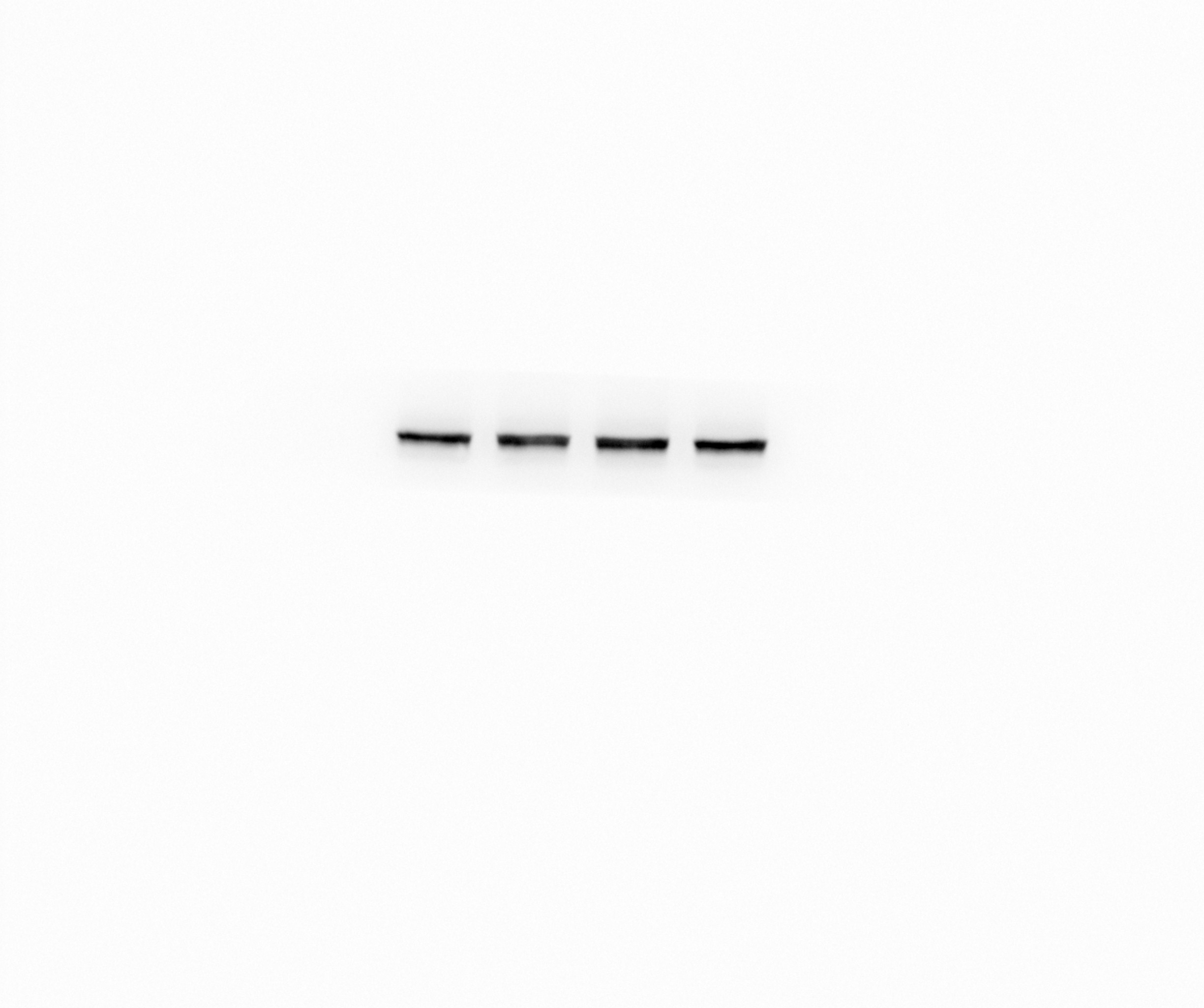

Supplement: Supplementary file 7 [file DataSheet2.ZIP › AKT/AKT.jpg]

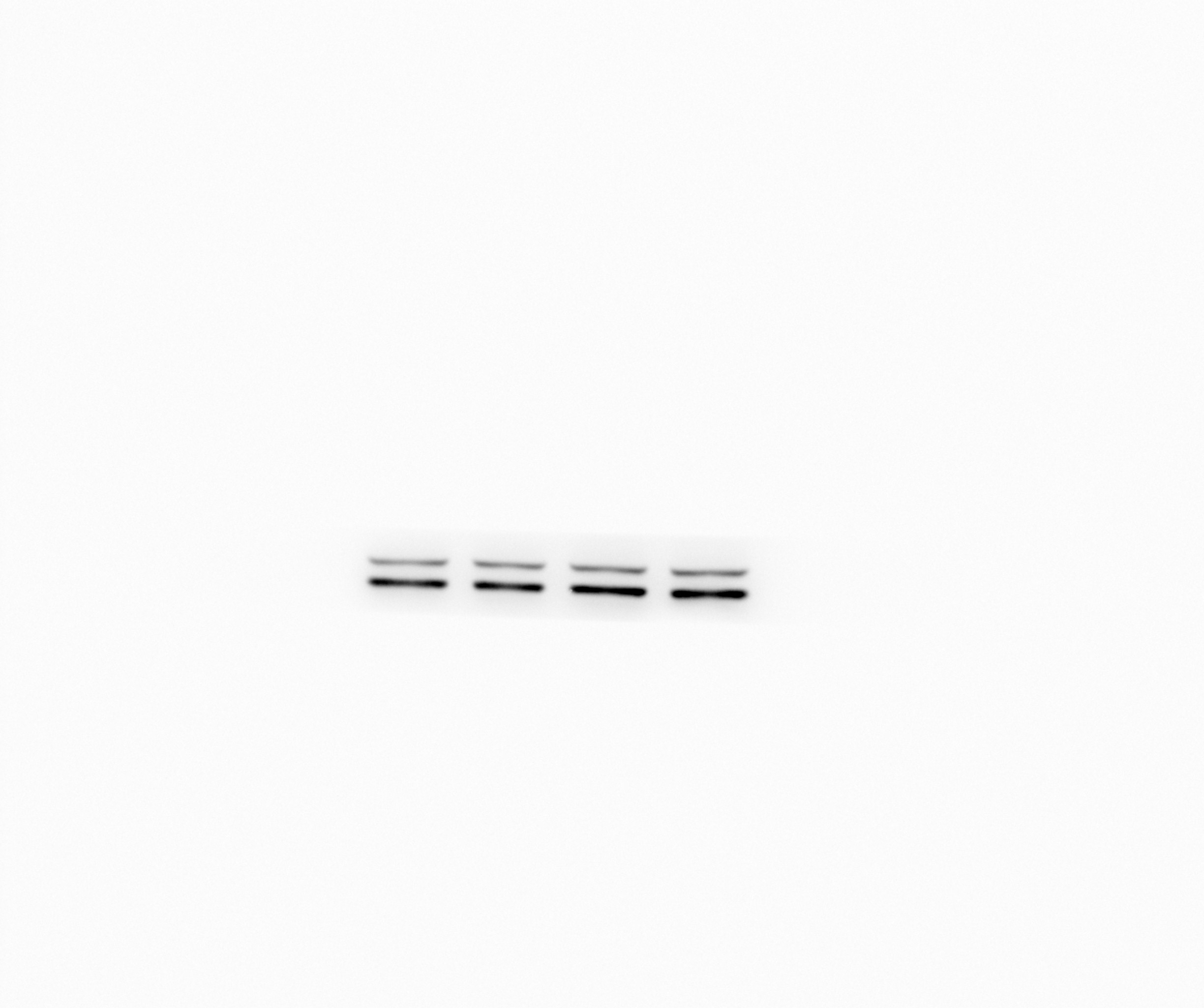

Supplement: Supplementary file 7 [file DataSheet2.ZIP › ERK/ERK-1.jpg]

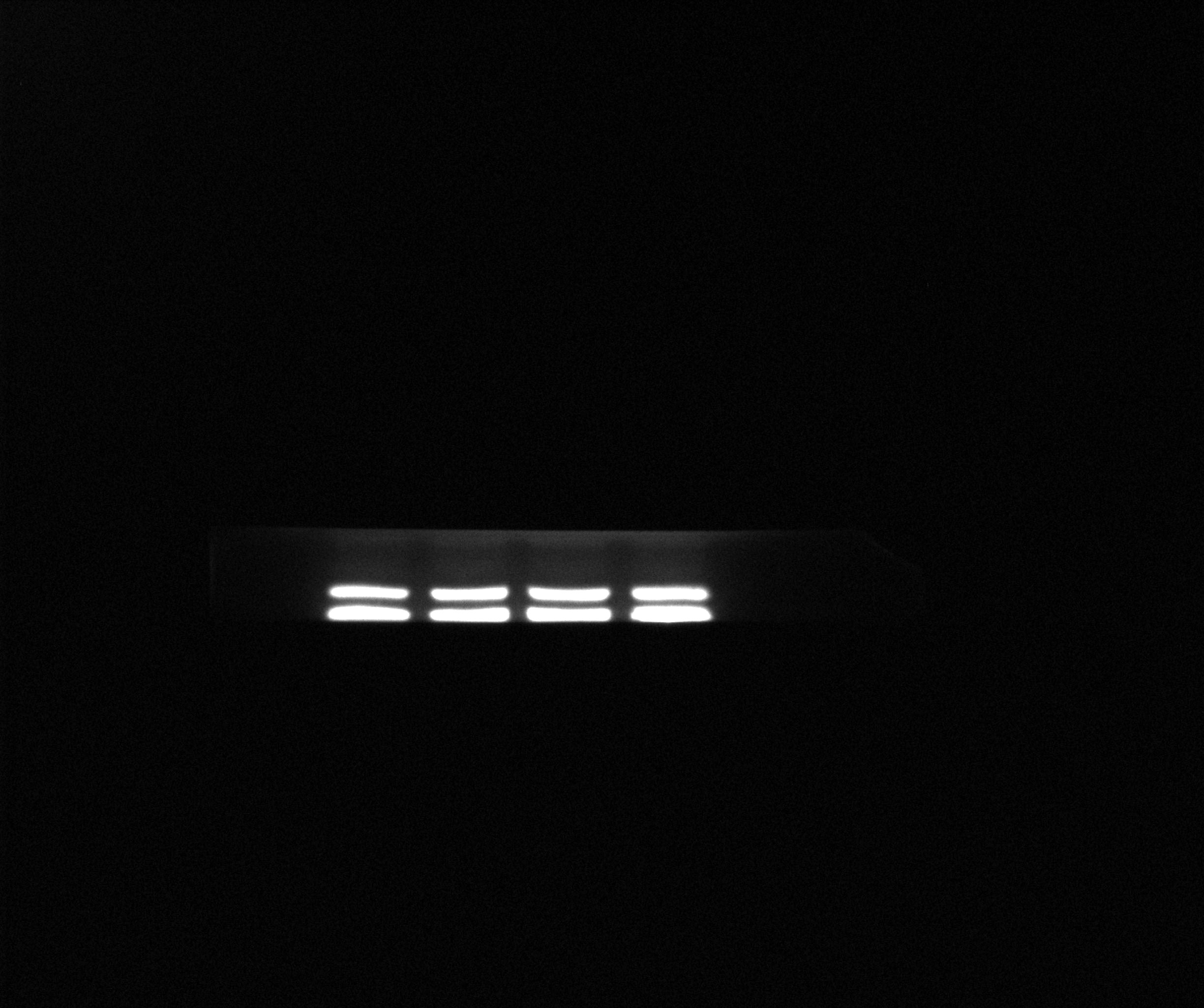

Supplement: Supplementary file 7 [file DataSheet2.ZIP › ERK/ERK-B.jpg]

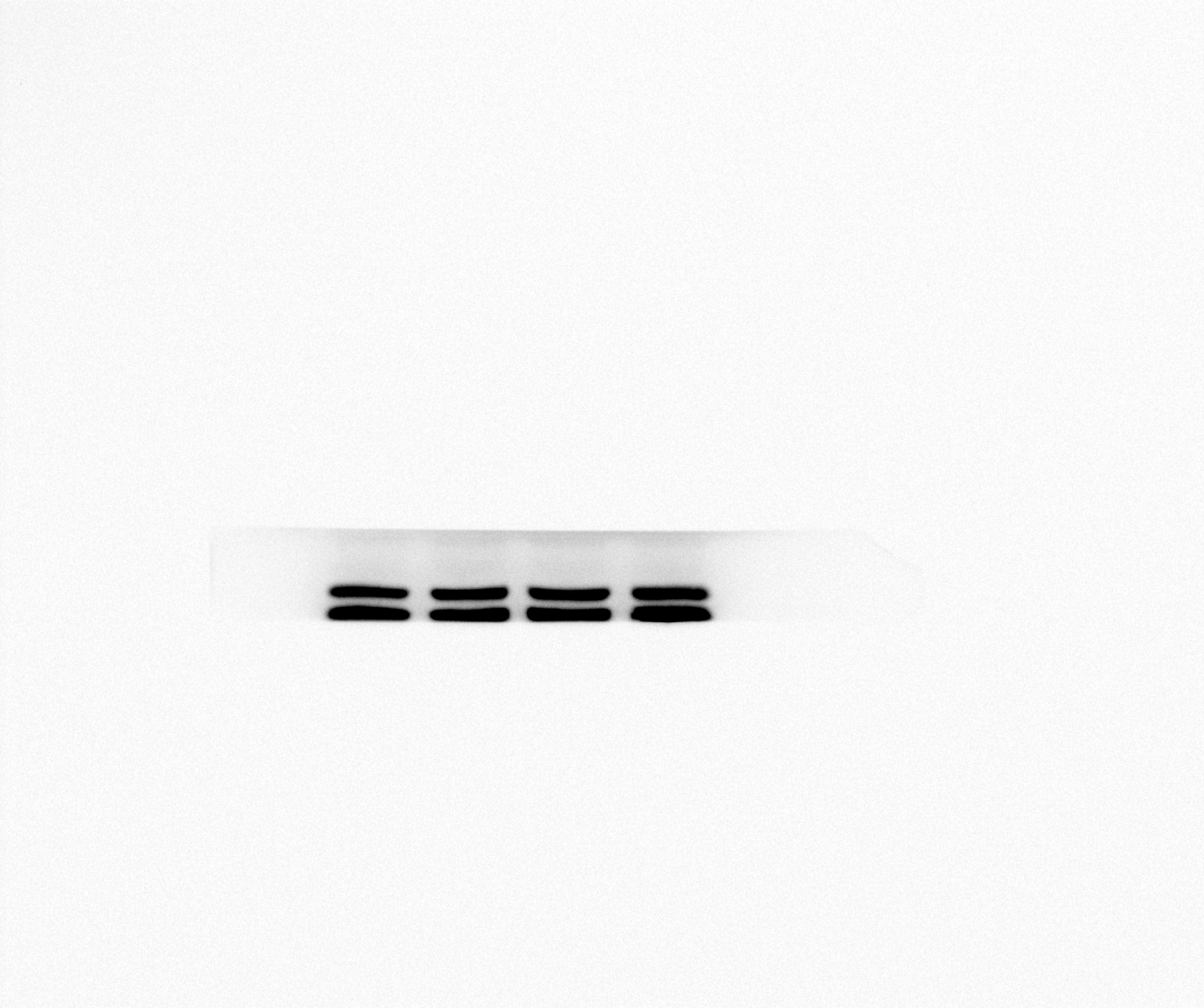

Supplement: Supplementary file 7 [file DataSheet2.ZIP › ERK/ERK-F-2.jpg]

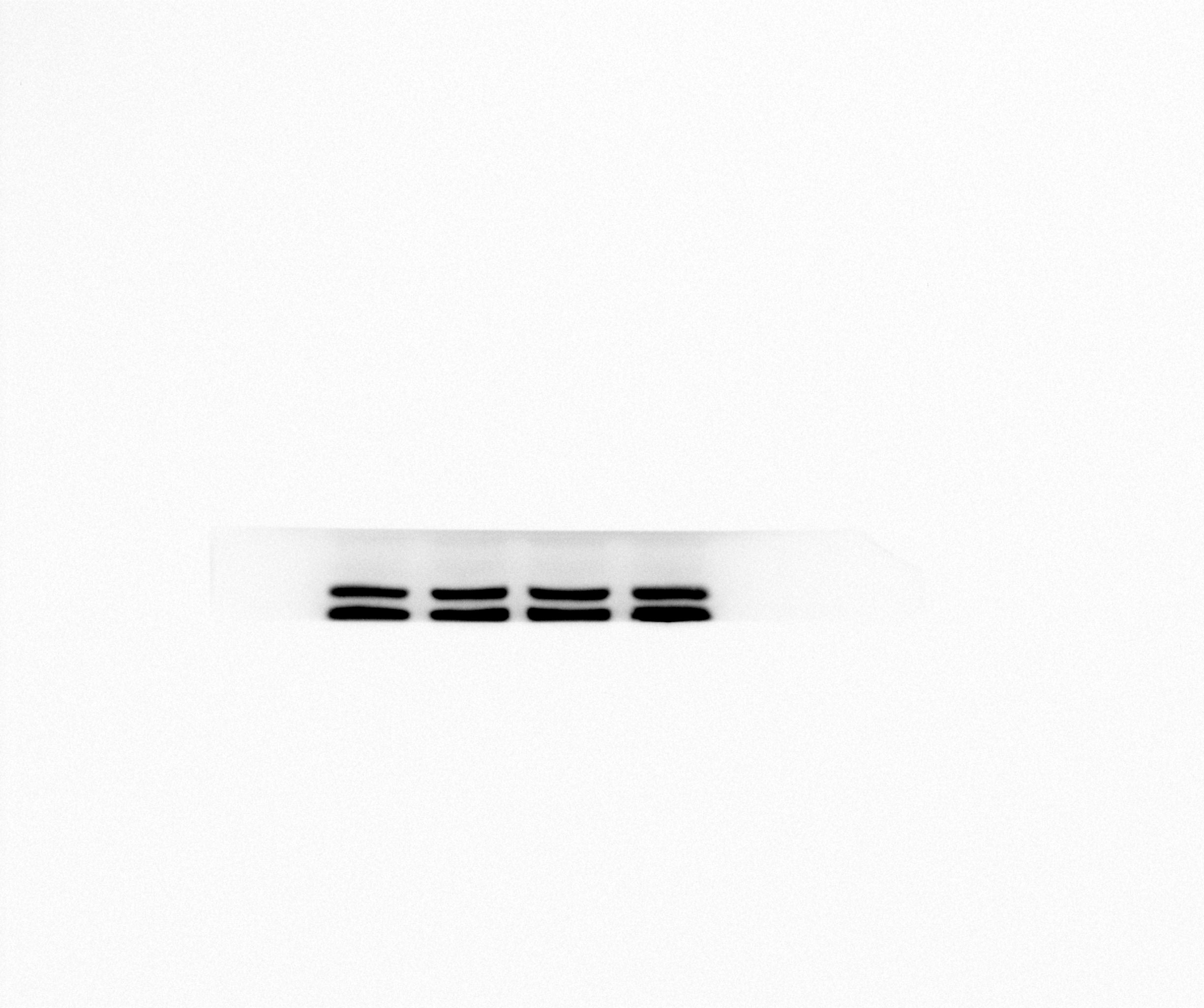

Supplement: Supplementary file 7 [file DataSheet2.ZIP › ERK/ERK-F.jpg]

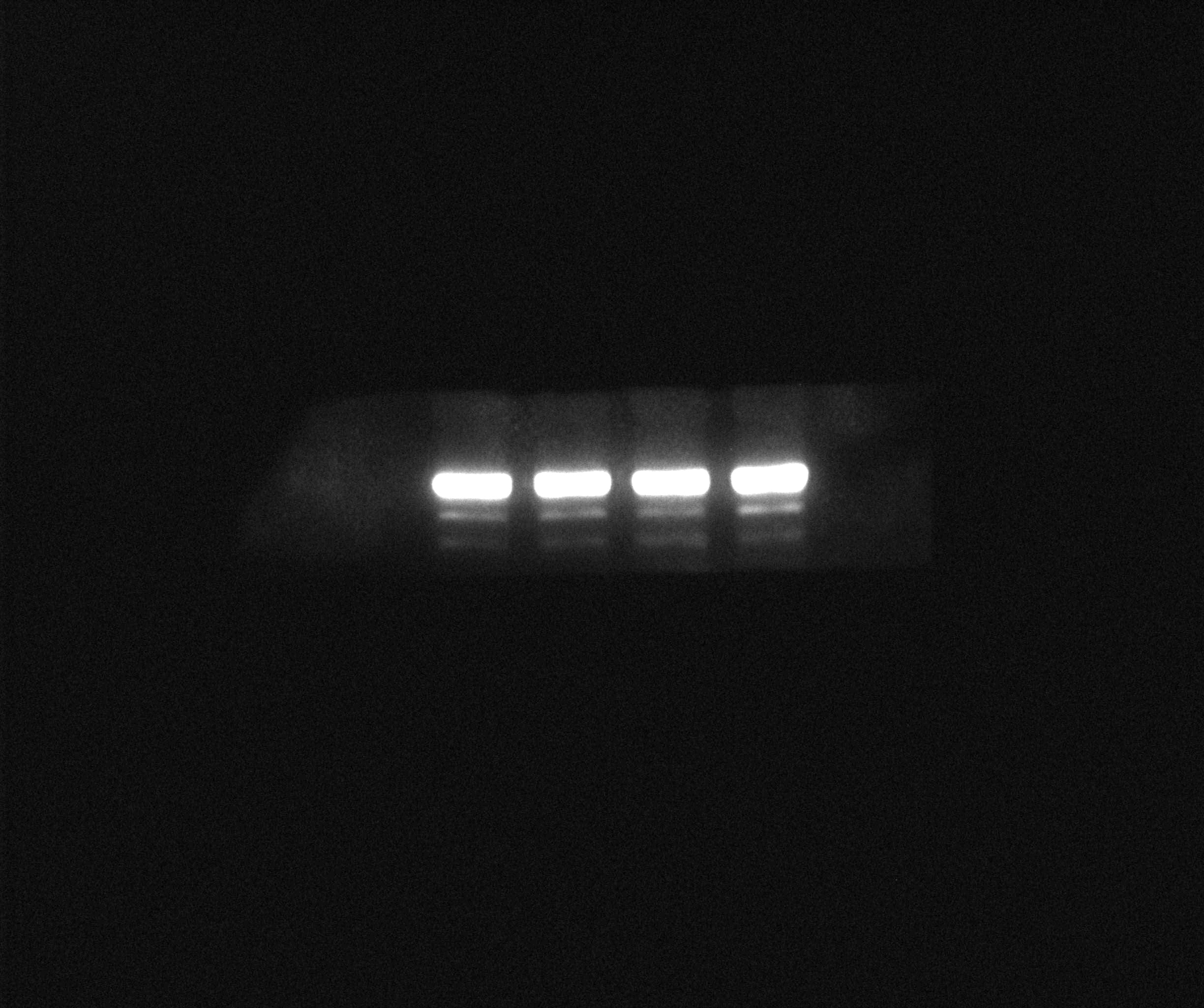

Supplement: Supplementary file 7 [file DataSheet2.ZIP › GAPDH/GAPDH-1 (2)-B-2.jpg]

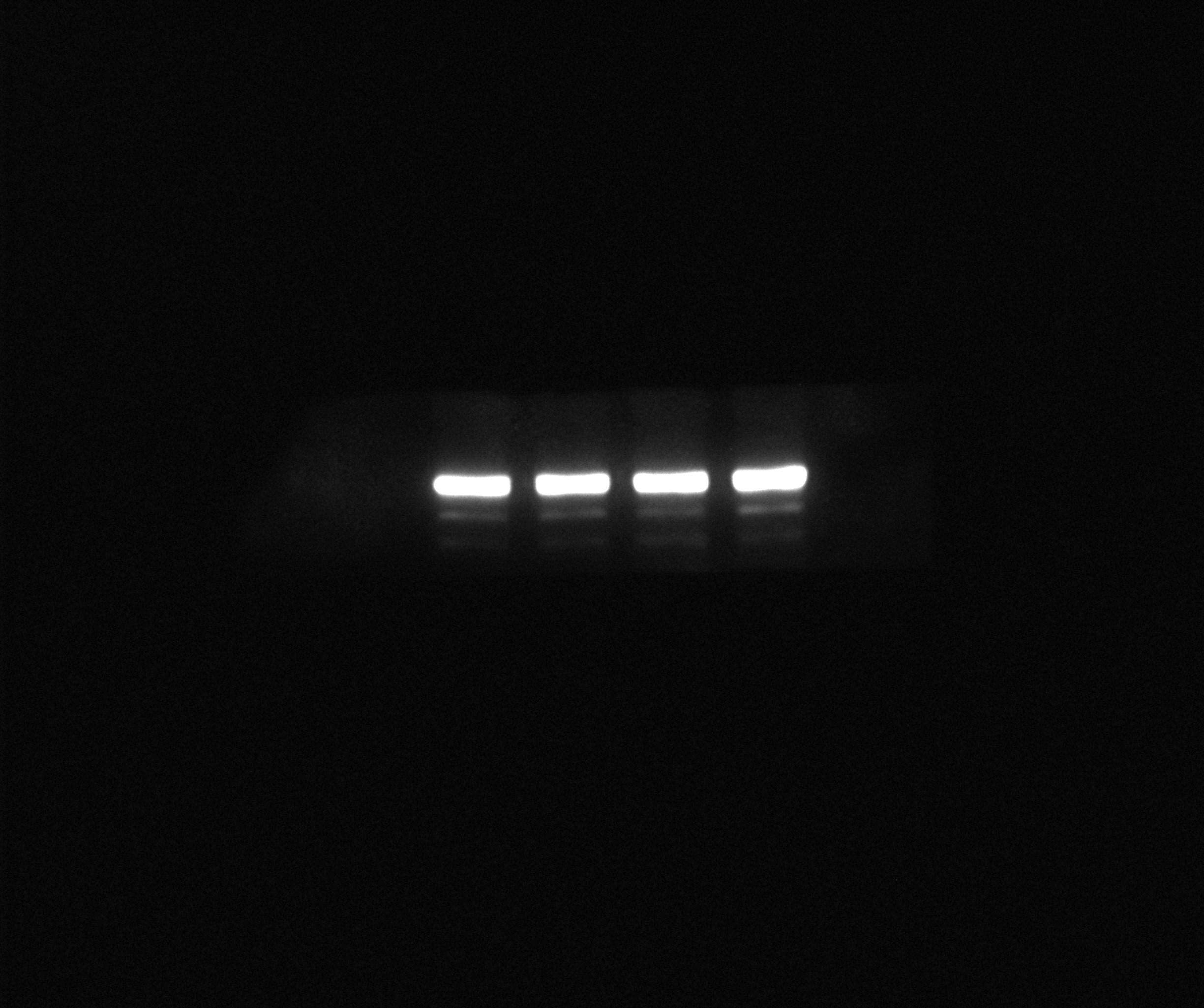

Supplement: Supplementary file 7 [file DataSheet2.ZIP › GAPDH/GAPDH-1 (2)-B.jpg]

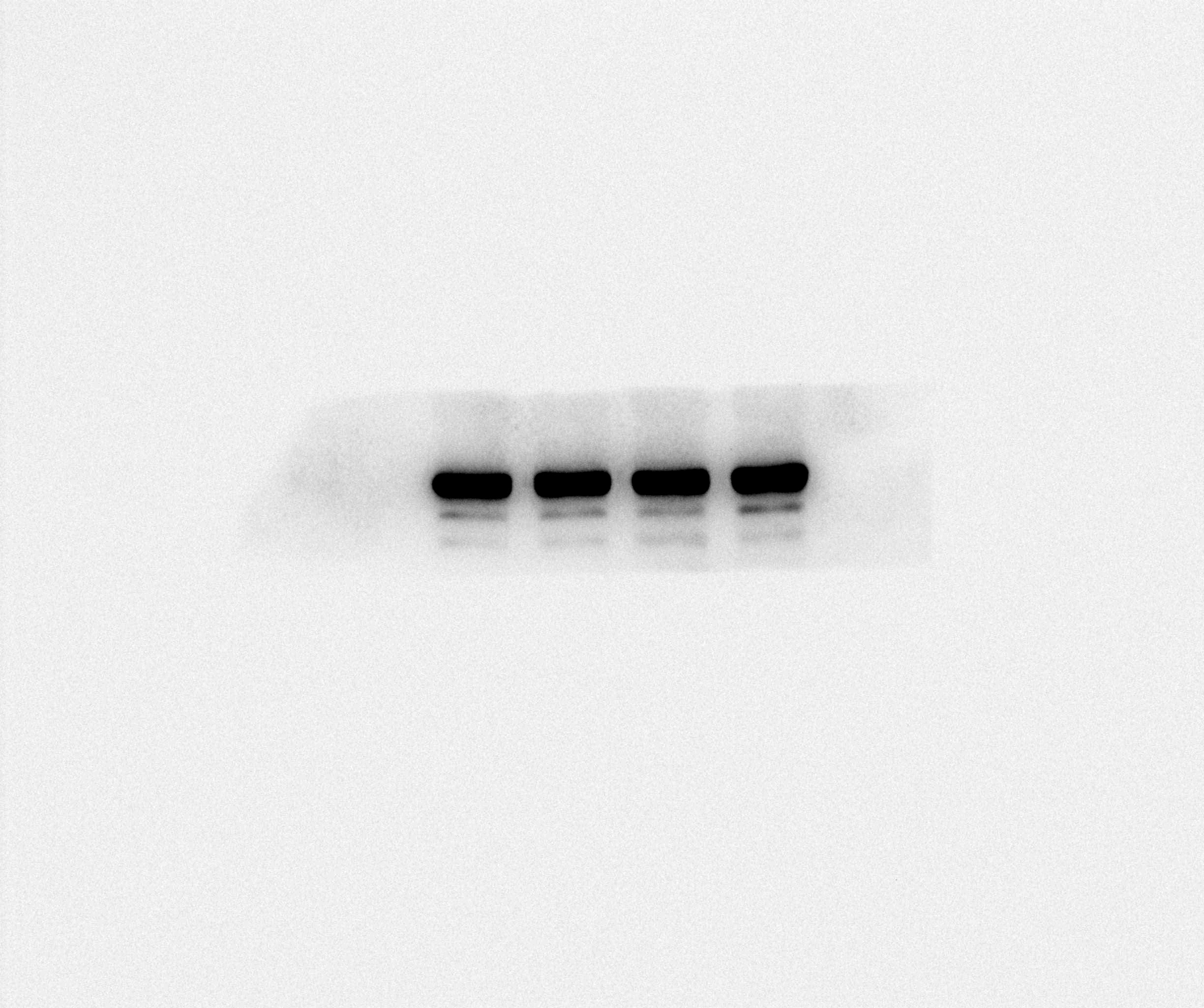

Supplement: Supplementary file 7 [file DataSheet2.ZIP › GAPDH/GAPDH-1 (2)-F-2.jpg]

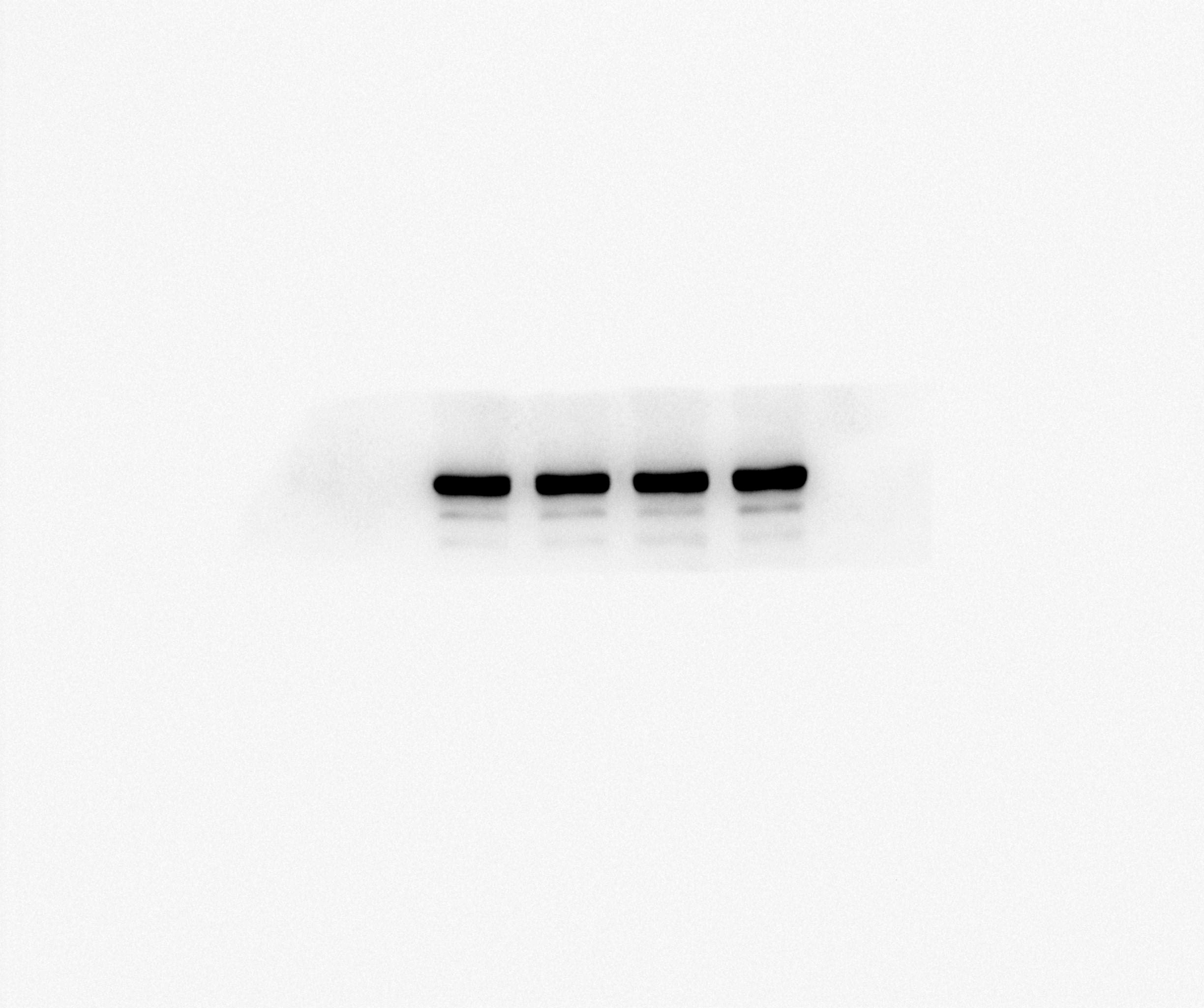

Supplement: Supplementary file 7 [file DataSheet2.ZIP › GAPDH/GAPDH-1 (2)-F.jpg]

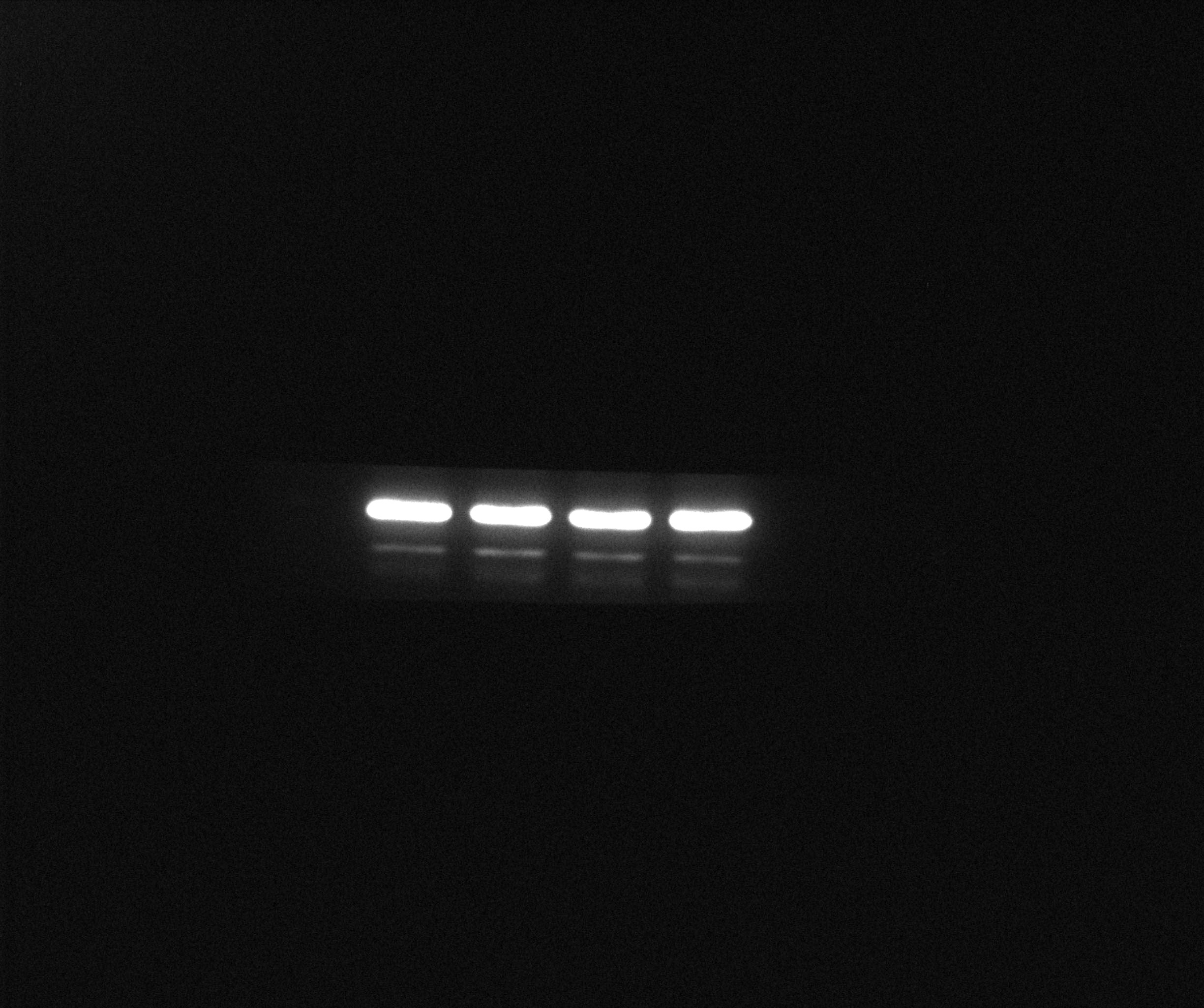

Supplement: Supplementary file 7 [file DataSheet2.ZIP › GAPDH/GAPDH-1(3)-B-2.jpg]

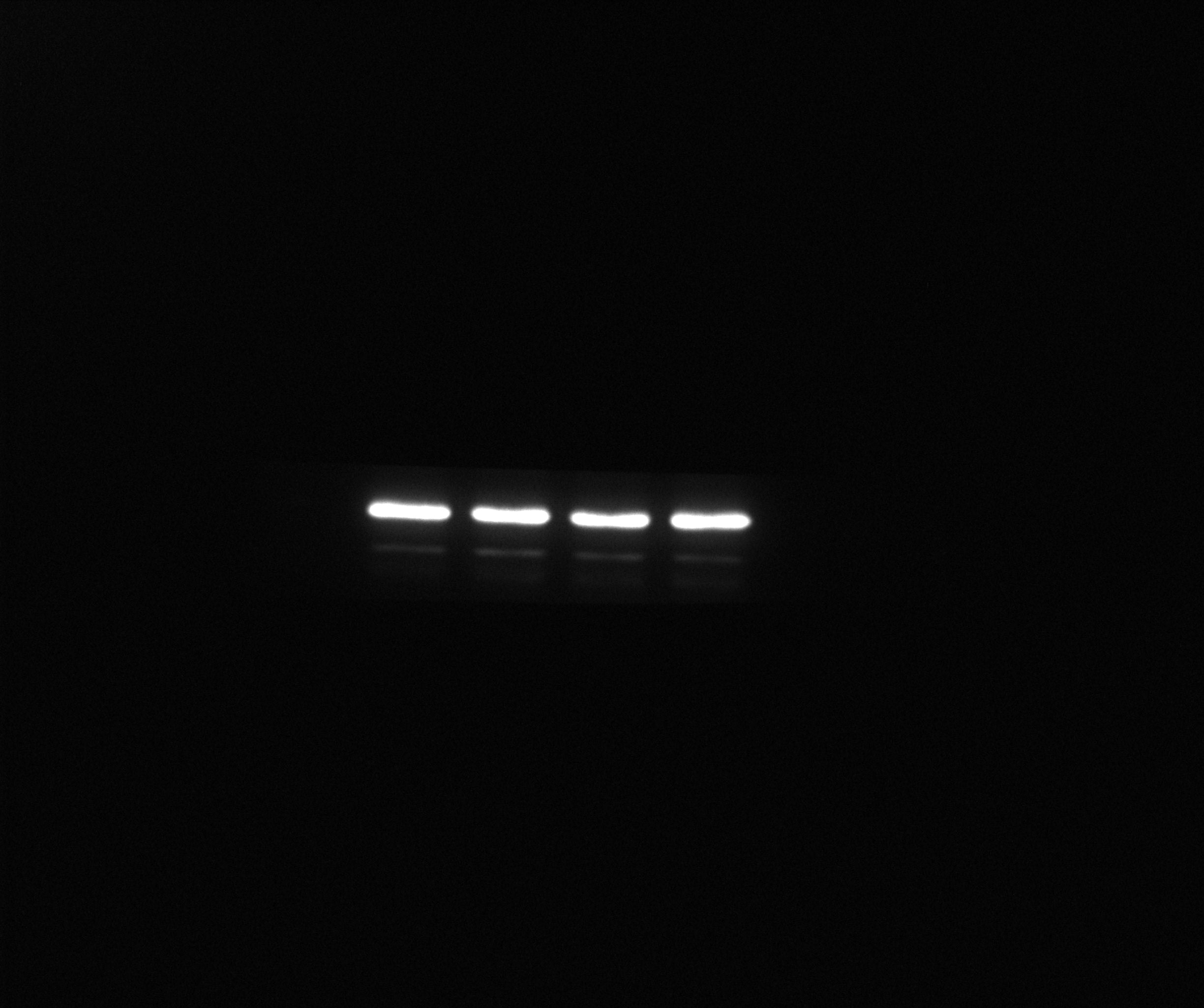

Supplement: Supplementary file 7 [file DataSheet2.ZIP › GAPDH/GAPDH-1(3)-B.jpg]

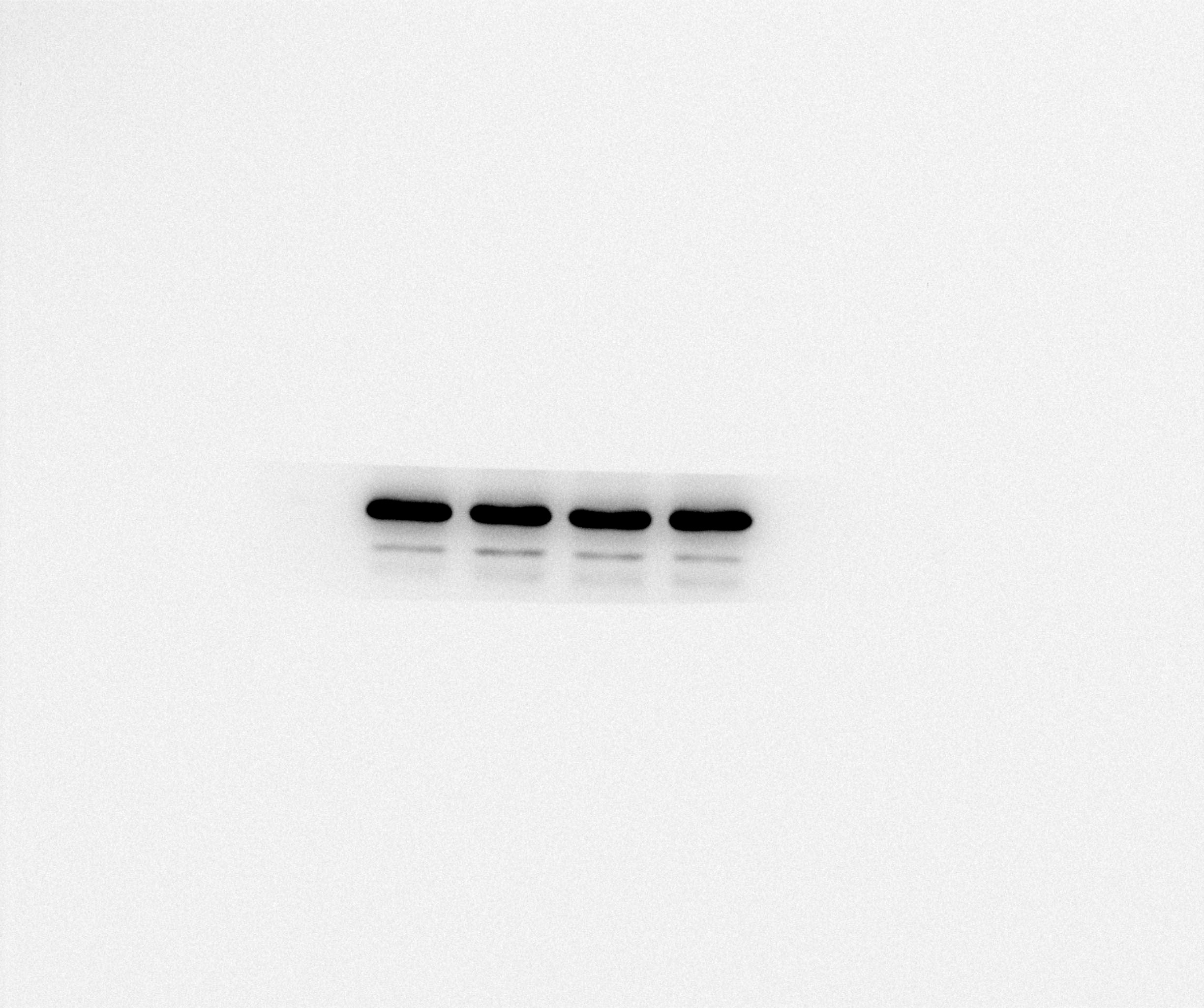

Supplement: Supplementary file 7 [file DataSheet2.ZIP › GAPDH/GAPDH-1(3)-F-2.jpg]

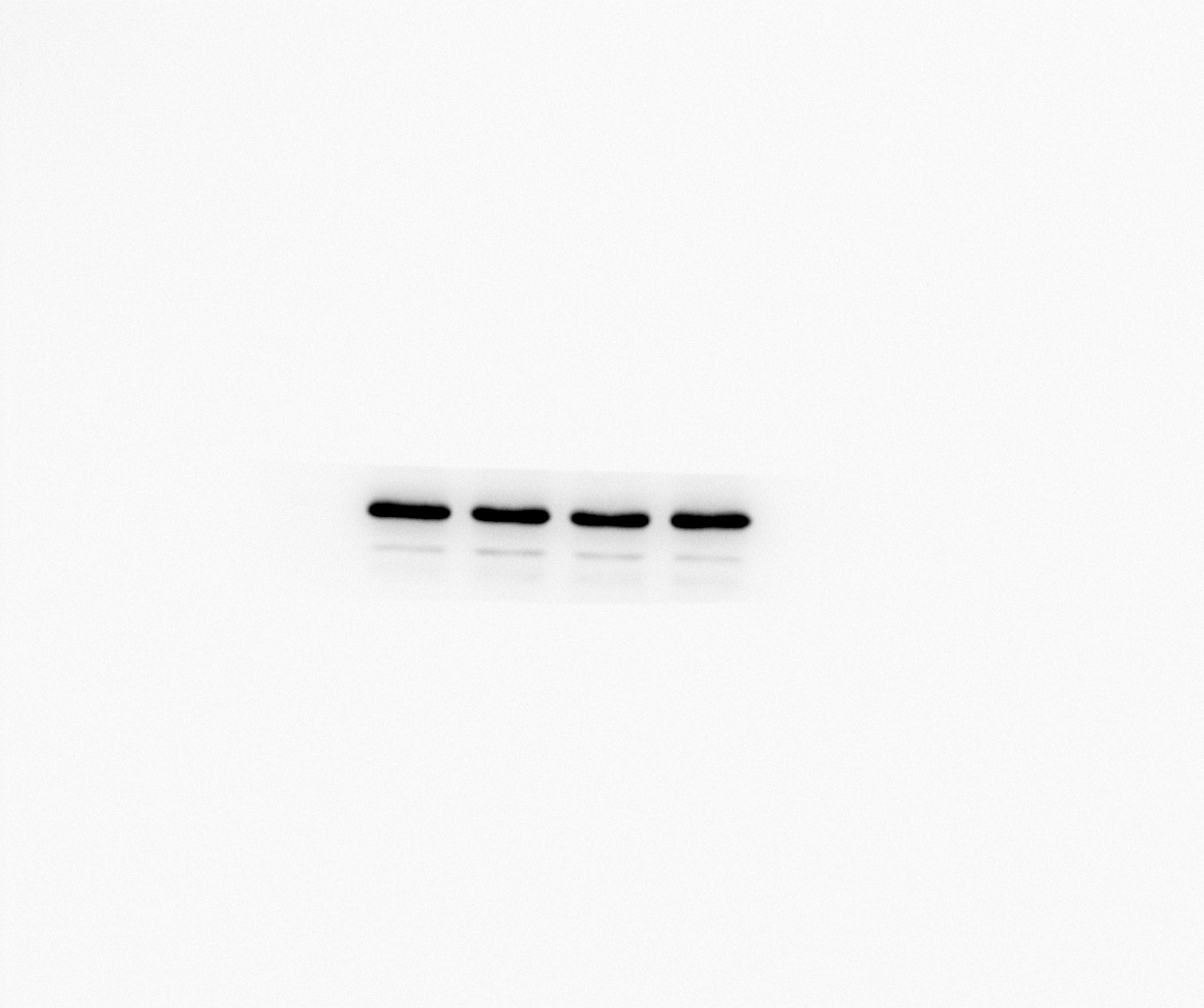

Supplement: Supplementary file 7 [file DataSheet2.ZIP › GAPDH/GAPDH-1(3)-F.jpg]

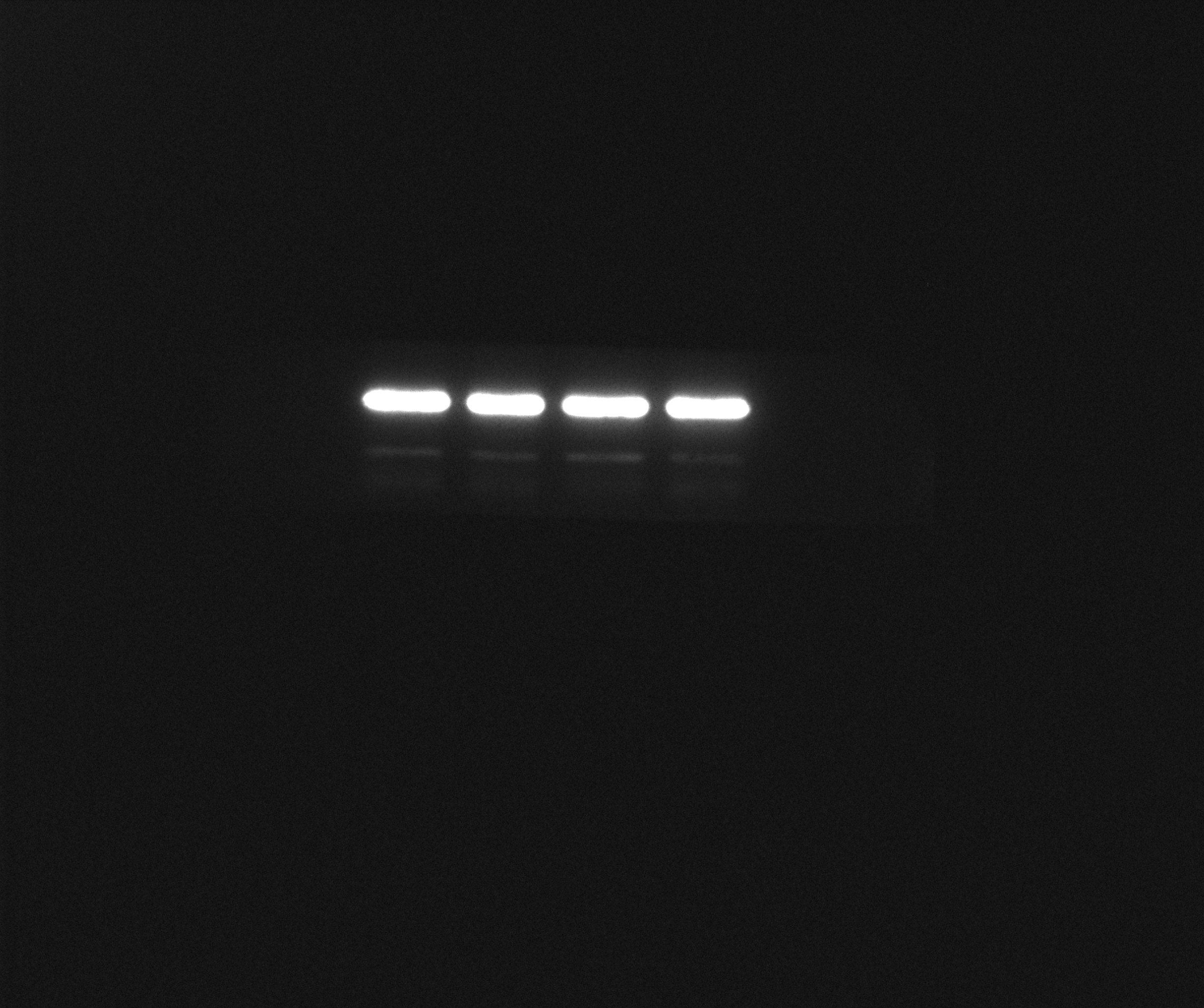

Supplement: Supplementary file 7 [file DataSheet2.ZIP › GAPDH/GAPDH-1-B-2.jpg]

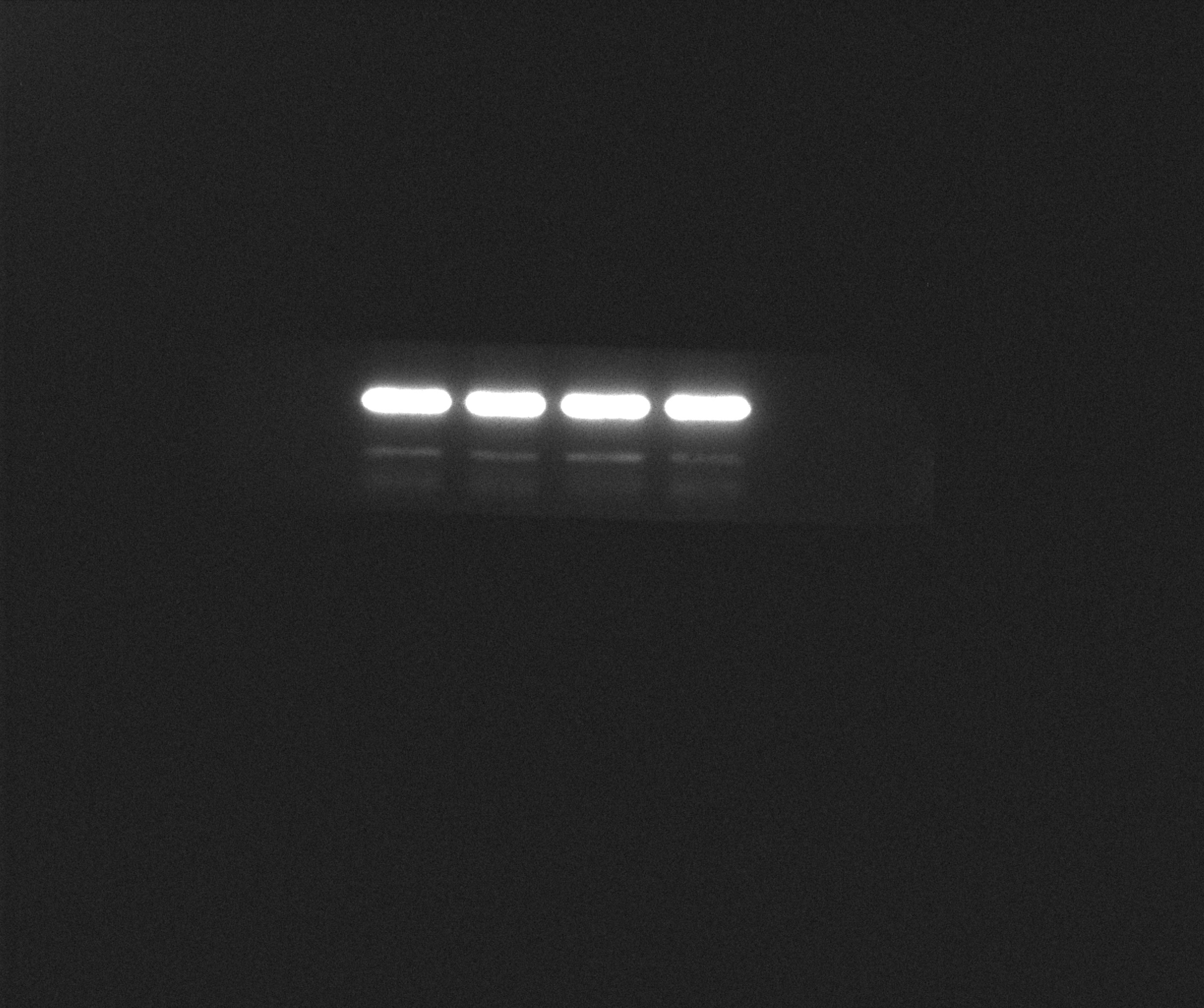

Supplement: Supplementary file 7 [file DataSheet2.ZIP › GAPDH/GAPDH-1-B.jpg]

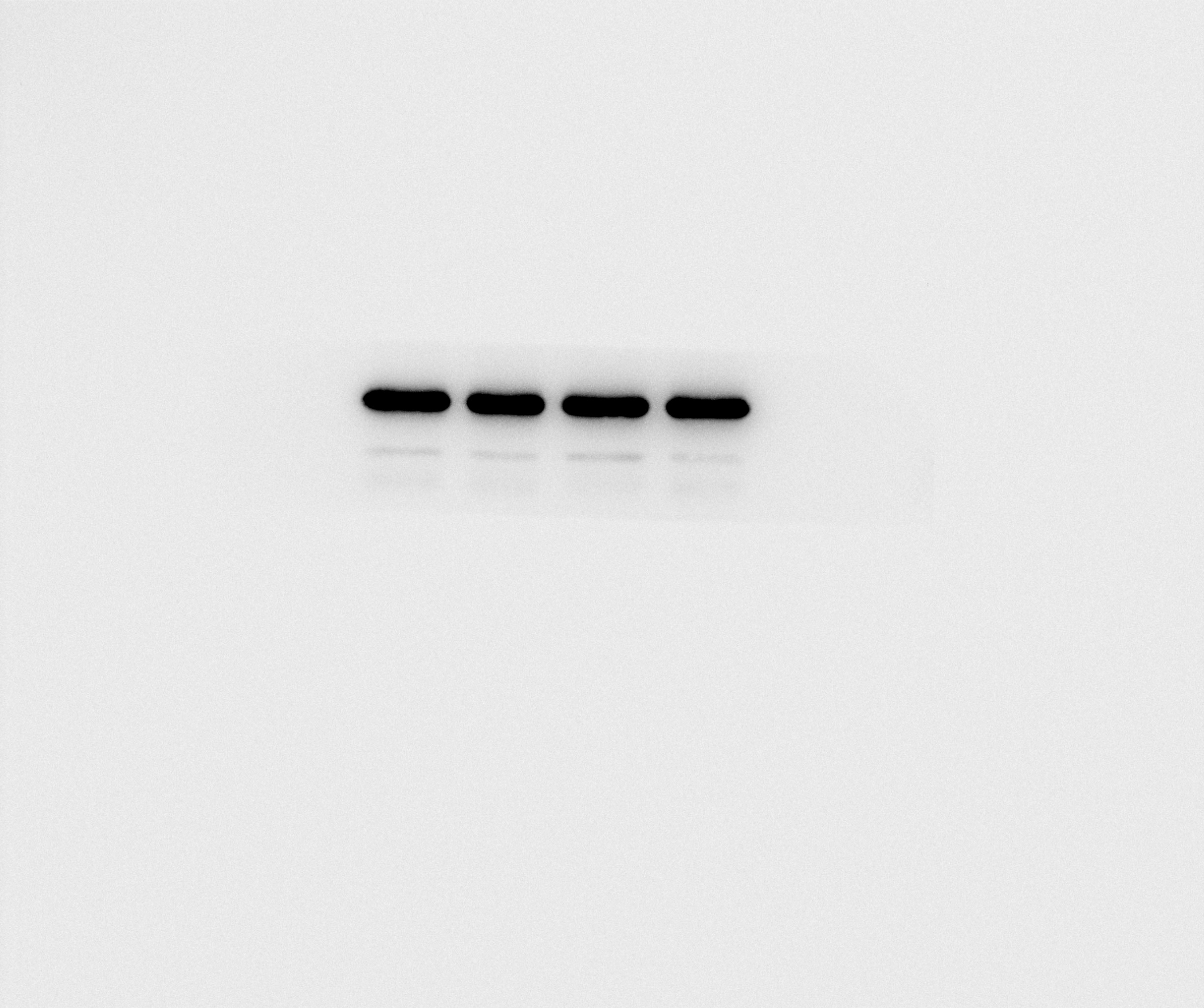

Supplement: Supplementary file 7 [file DataSheet2.ZIP › GAPDH/GAPDH-1-F-2.jpg]

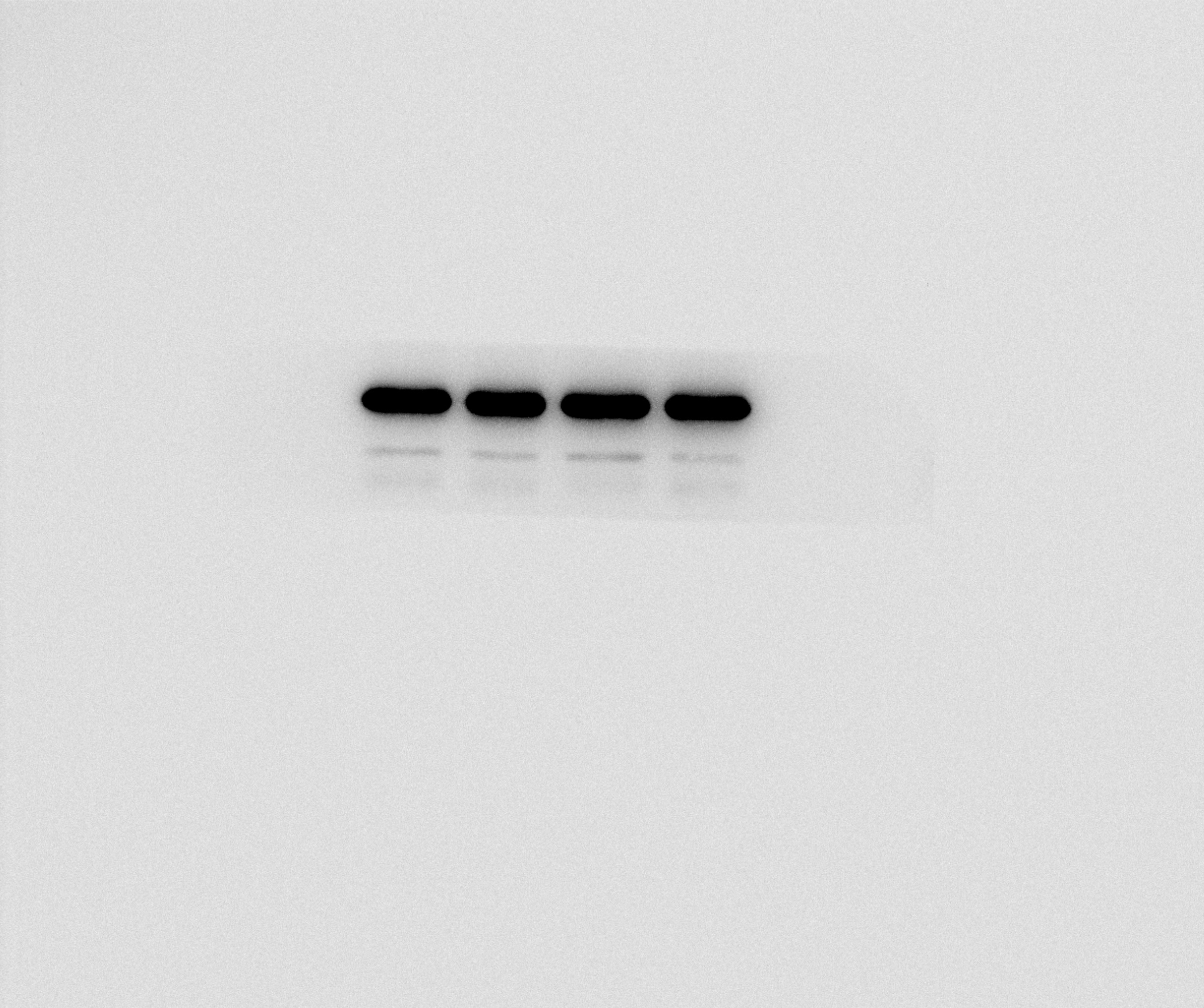

Supplement: Supplementary file 7 [file DataSheet2.ZIP › GAPDH/GAPDH-1-F.jpg]

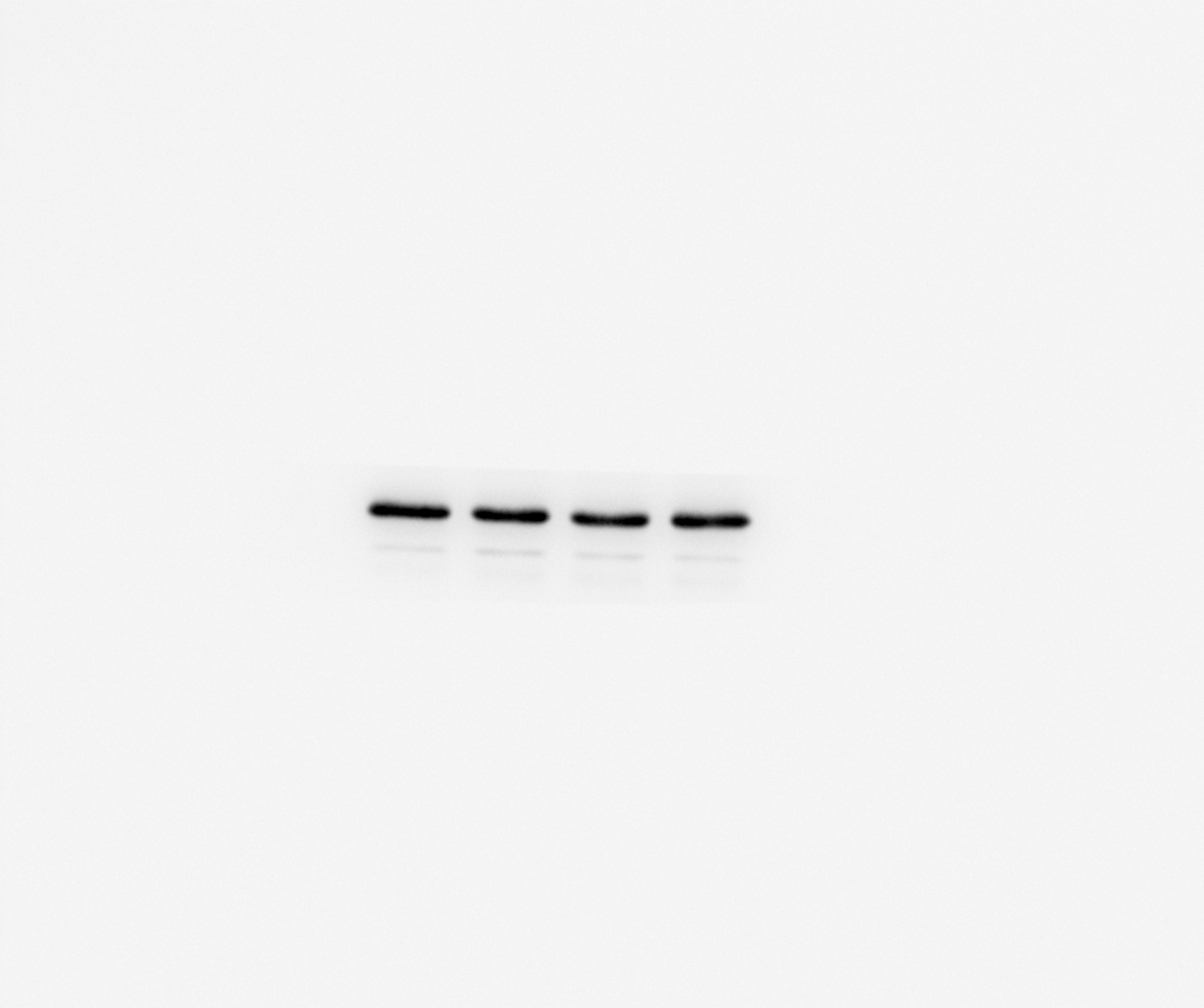

Supplement: Supplementary file 7 [file DataSheet2.ZIP › GAPDH/GAPDH-1.jpg]

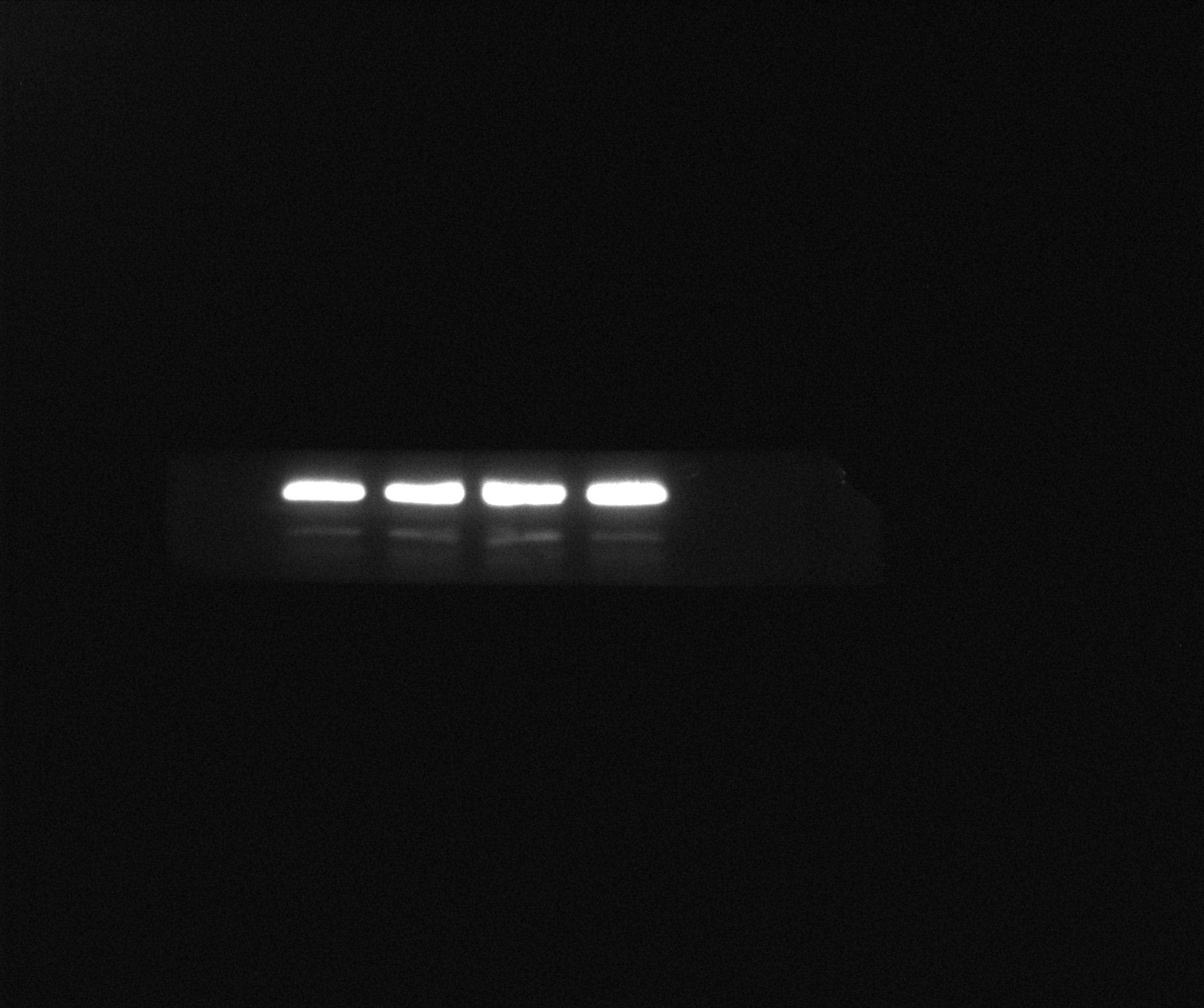

Supplement: Supplementary file 7 [file DataSheet2.ZIP › GAPDH/GAPDH-2-B-2.jpg]

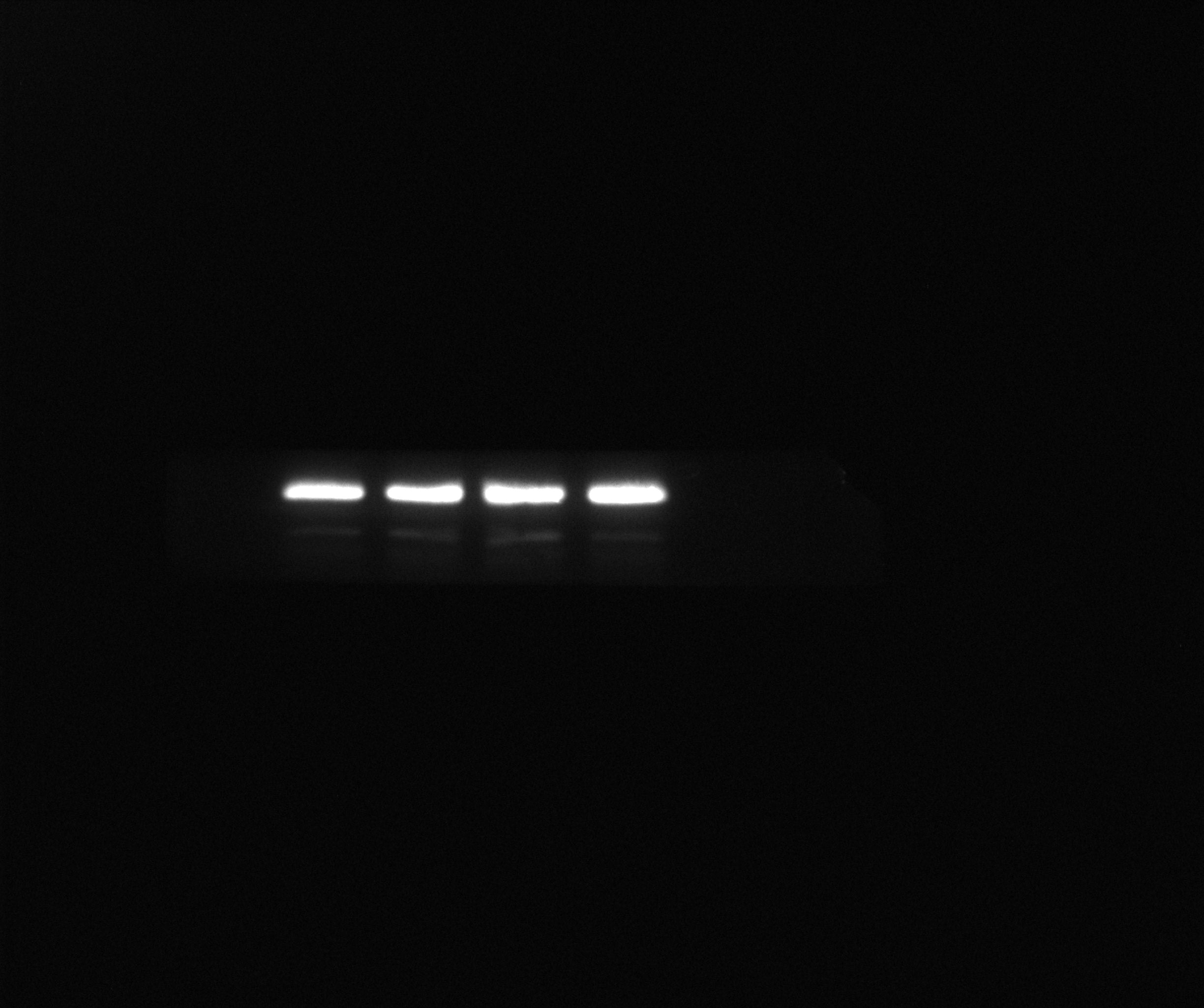

Supplement: Supplementary file 7 [file DataSheet2.ZIP › GAPDH/GAPDH-2-B.jpg]

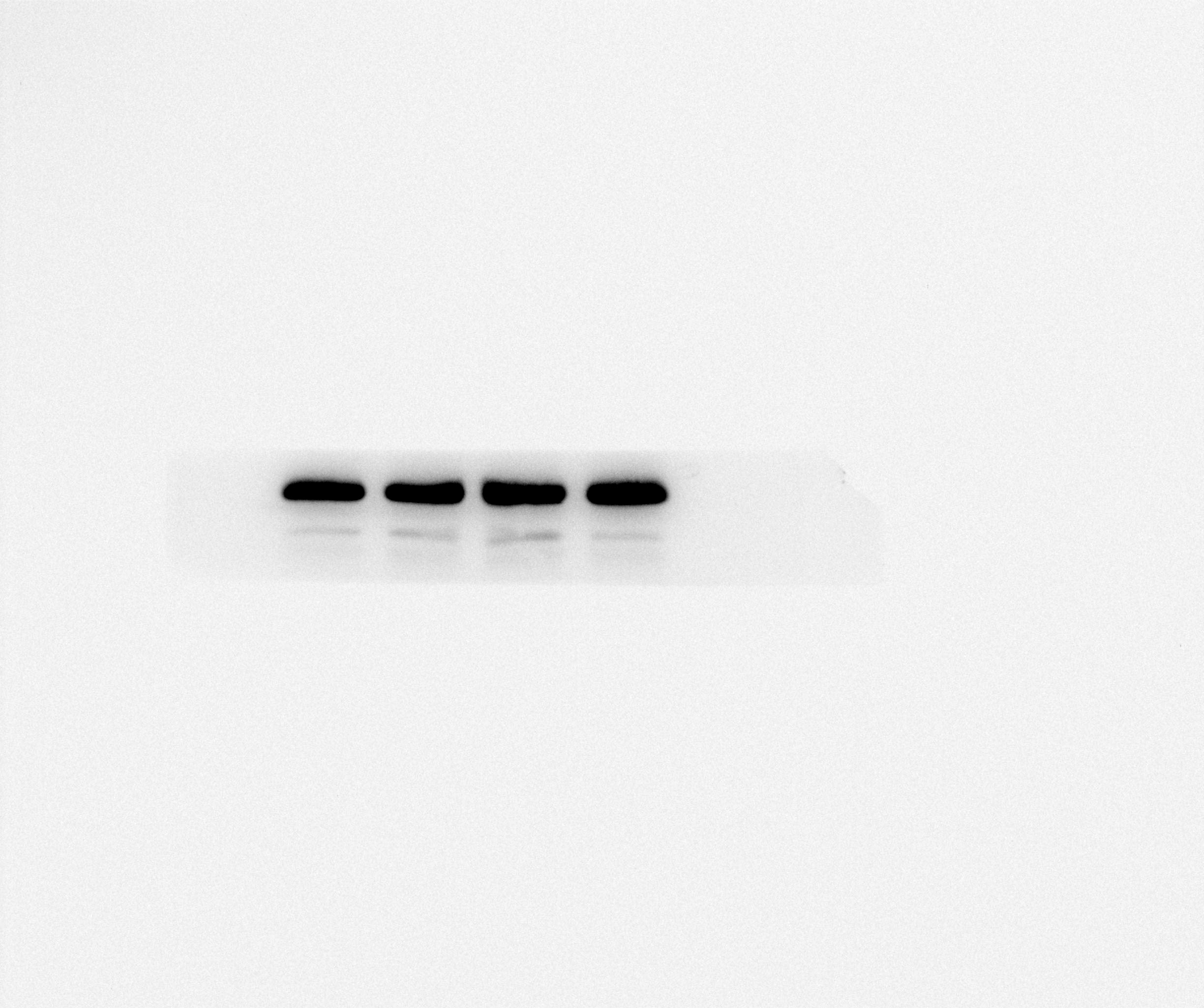

Supplement: Supplementary file 7 [file DataSheet2.ZIP › GAPDH/GAPDH-2-F-2.jpg]

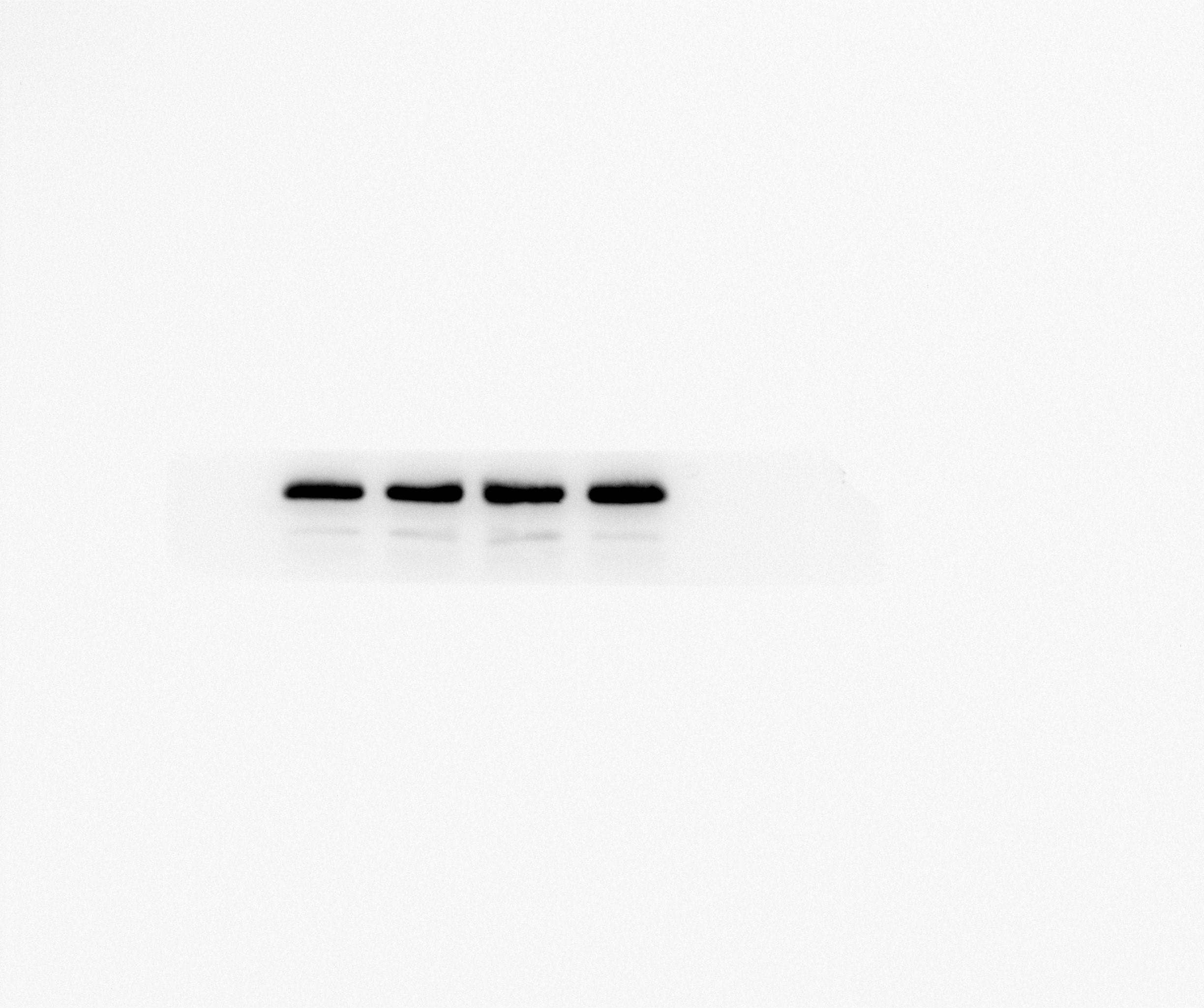

Supplement: Supplementary file 7 [file DataSheet2.ZIP › GAPDH/GAPDH-2-F.jpg]
