# Supplementary figures and images for: Paclitaxel Inhibits Synoviocyte Migration and Inflammatory Mediator Production in Rheumatoid Arthritis (part 2 of 2)
Source: Front Pharmacol. 2021 Sep 9;12:714566. doi: 10.3389/fphar.2021.714566 (PMC8458635; doi:10.3389/fphar.2021.714566)

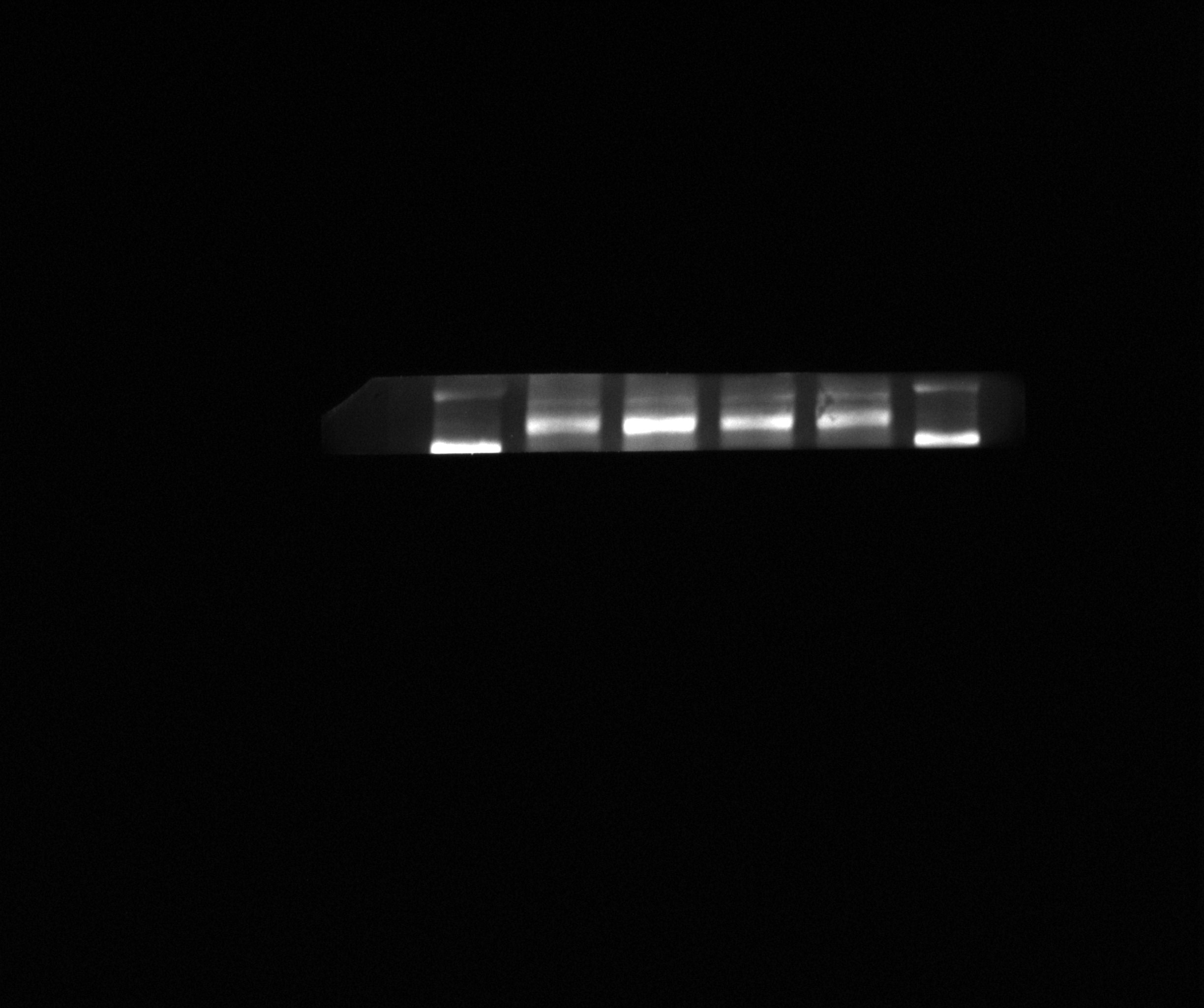

Supplement: Supplementary file 7 [file DataSheet2.ZIP › HIF-1a┴/HIF1a-1;500-B.jpg]

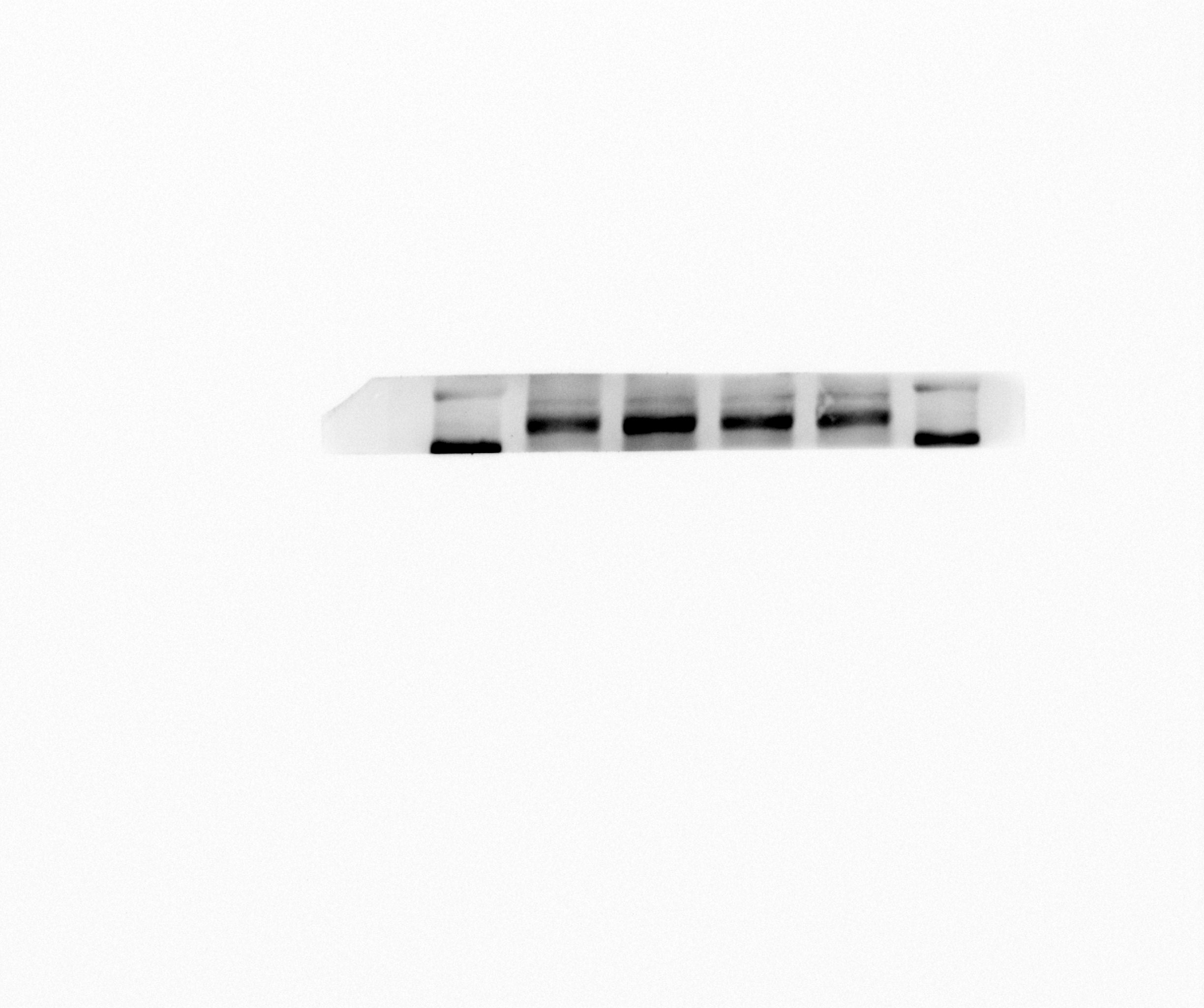

Supplement: Supplementary file 7 [file DataSheet2.ZIP › HIF-1a┴/HIF1a-1;500-F-2.jpg]

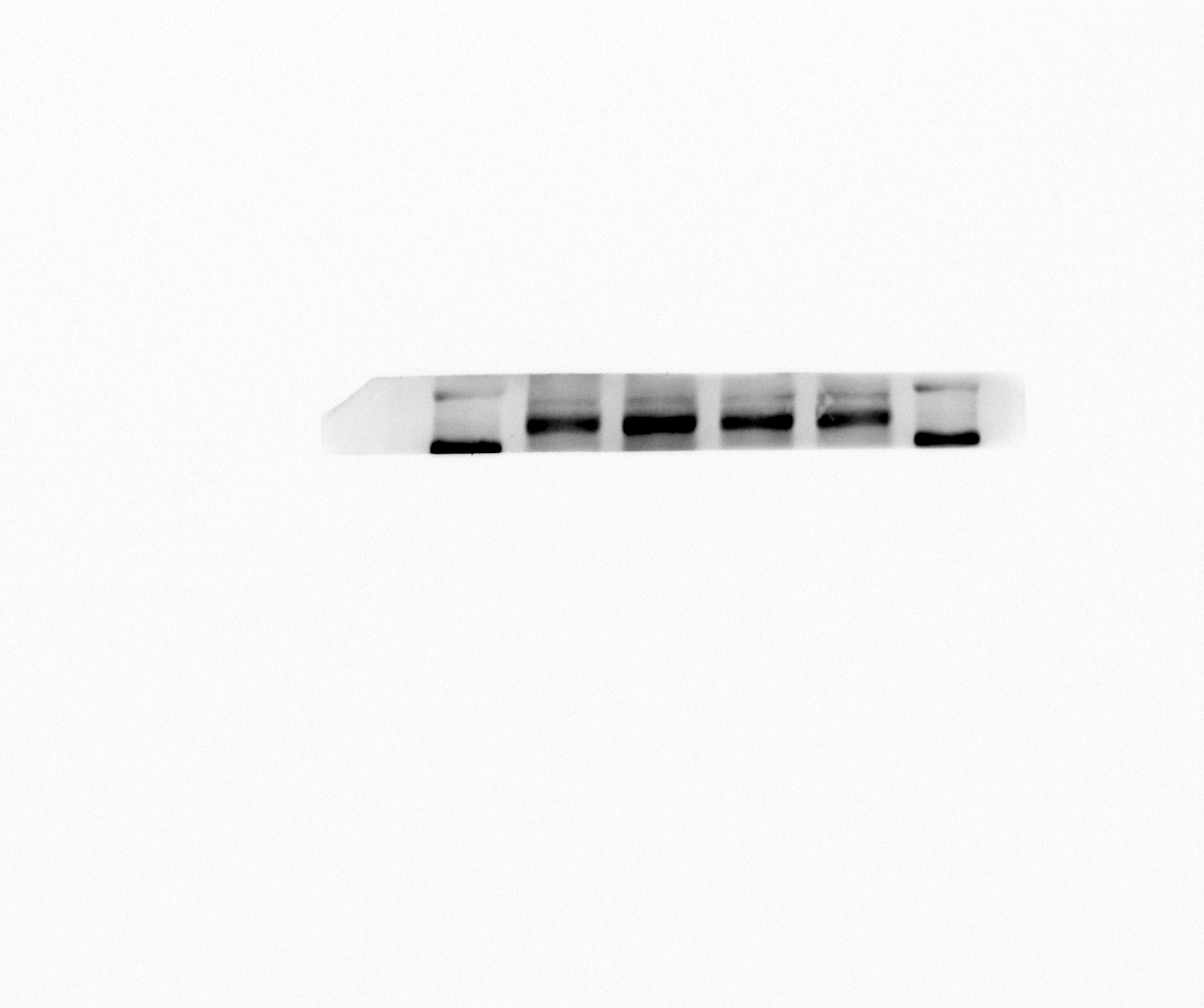

Supplement: Supplementary file 7 [file DataSheet2.ZIP › HIF-1a┴/HIF1a-1;500-F.jpg]

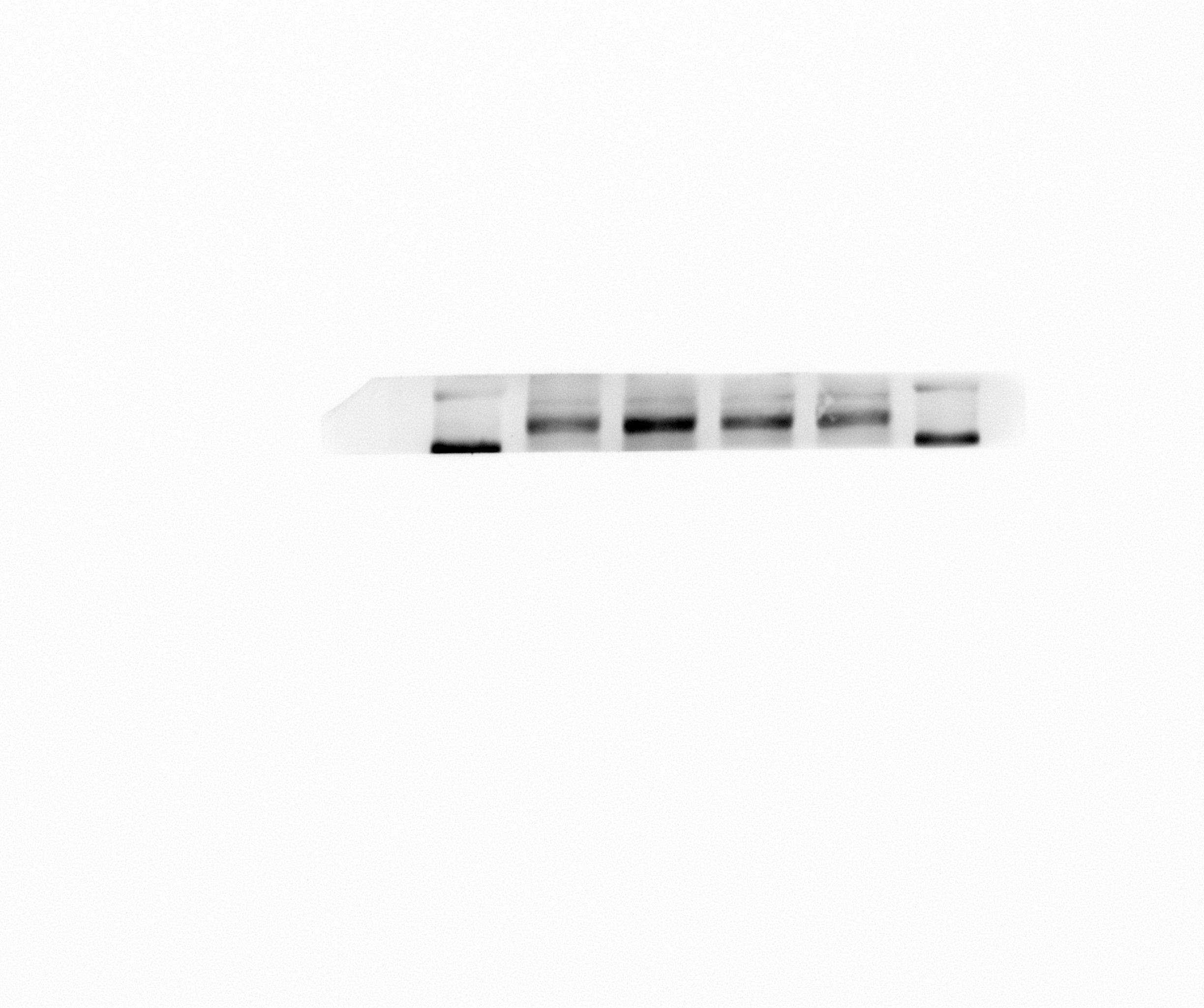

Supplement: Supplementary file 7 [file DataSheet2.ZIP › HIF-1a┴/HIF1a-1;500.jpg]

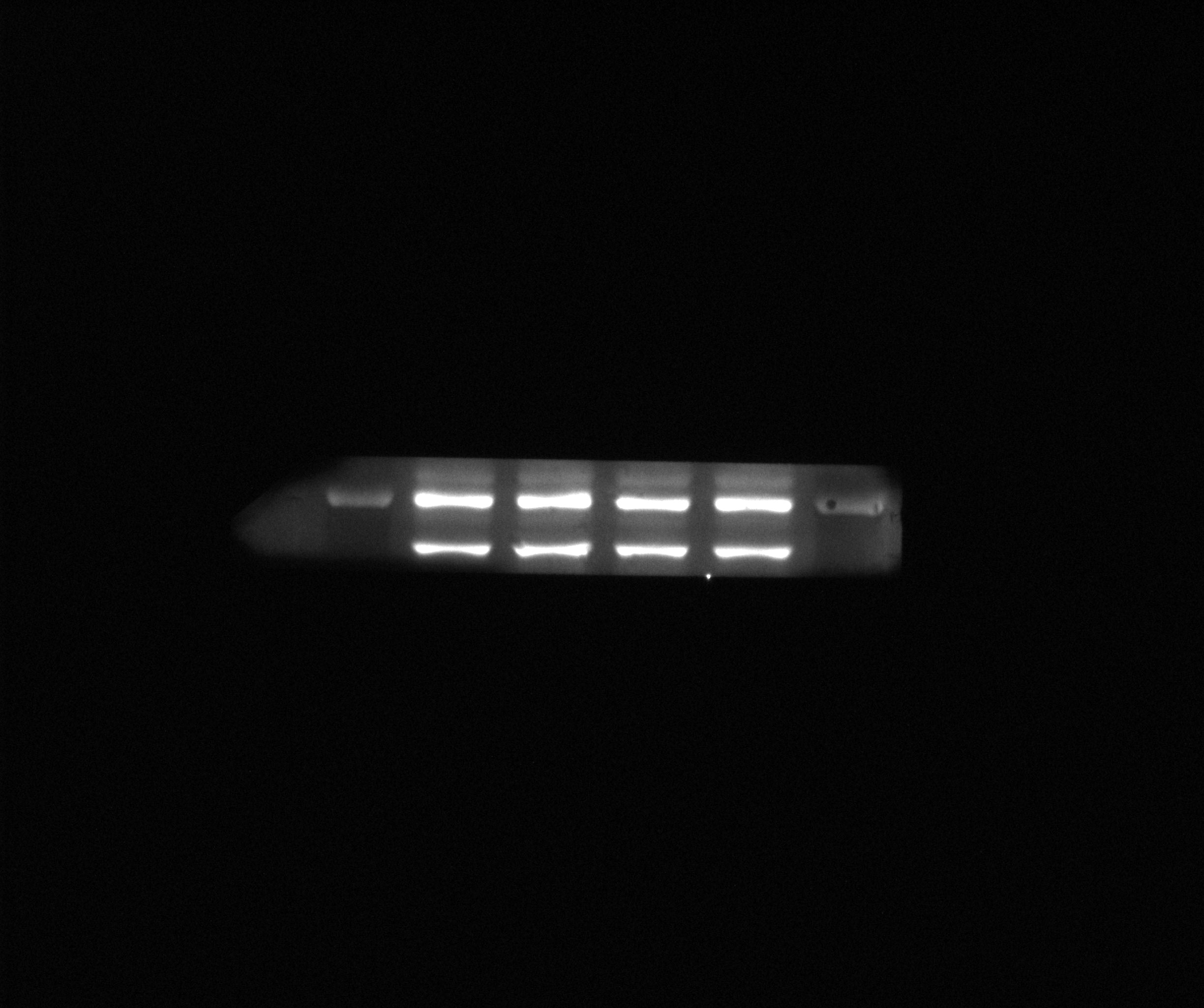

Supplement: Supplementary file 7 [file DataSheet2.ZIP › JNK/JNK-B.jpg]

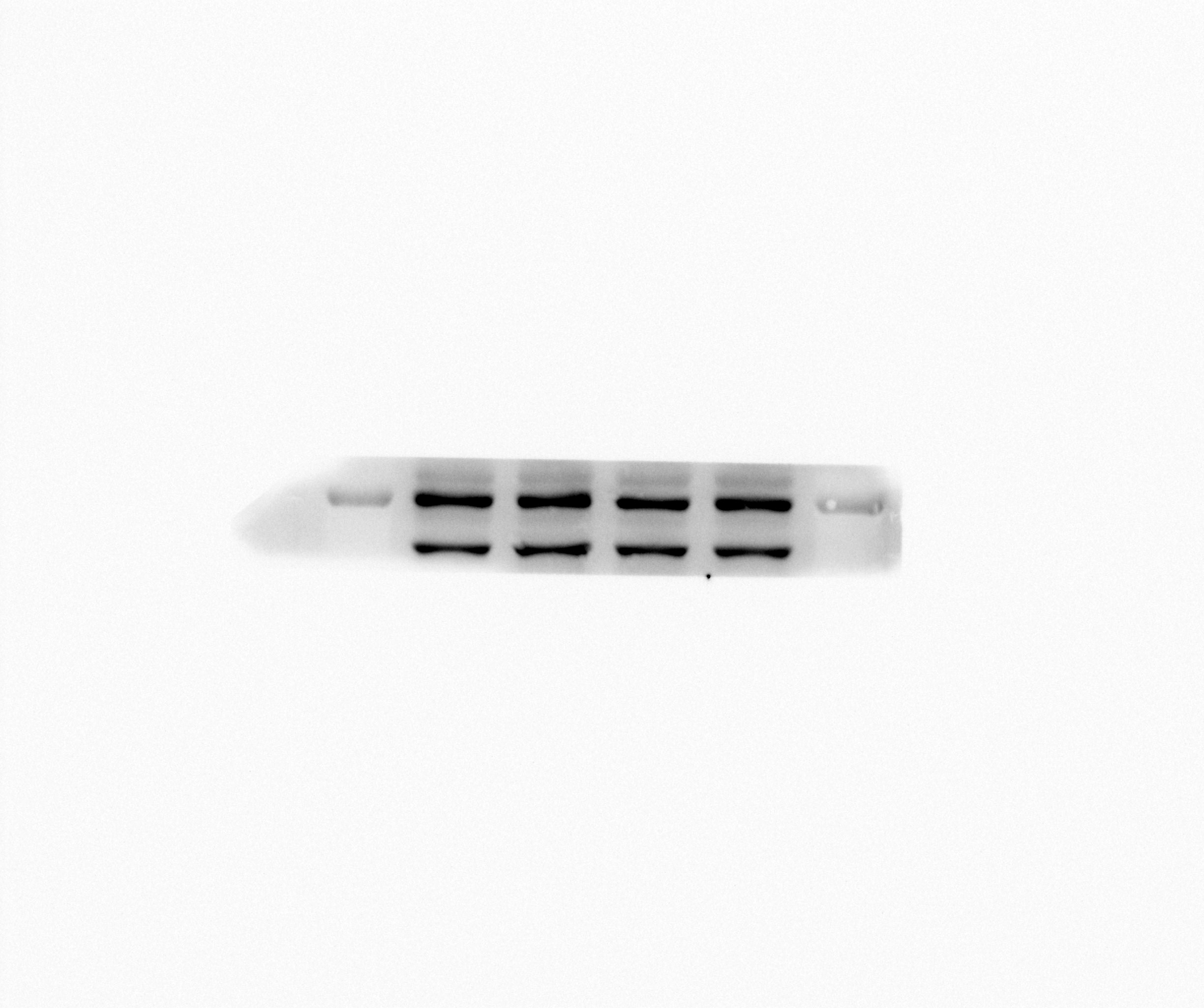

Supplement: Supplementary file 7 [file DataSheet2.ZIP › JNK/JNK-F-2.jpg]

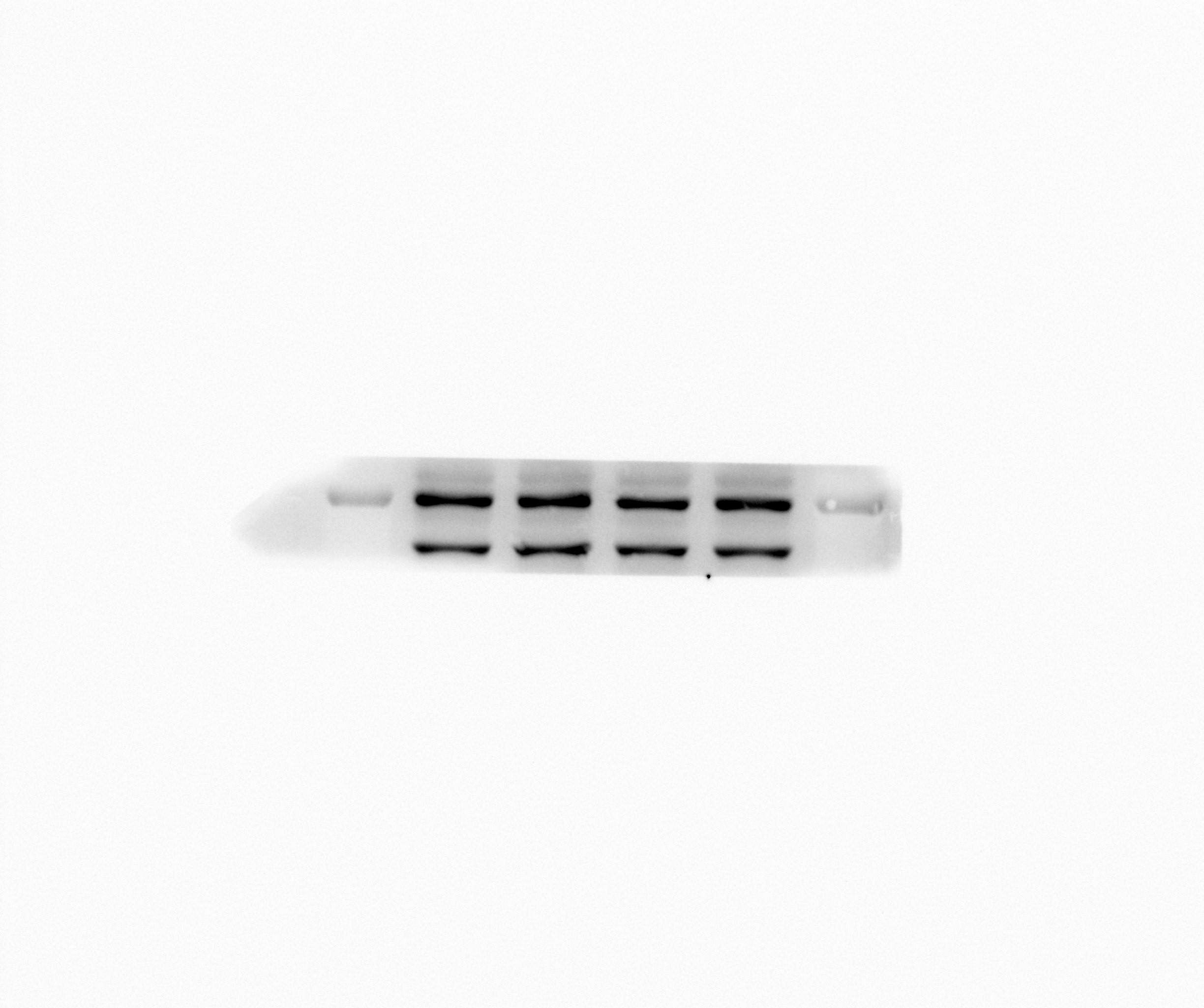

Supplement: Supplementary file 7 [file DataSheet2.ZIP › JNK/JNK-F.jpg]

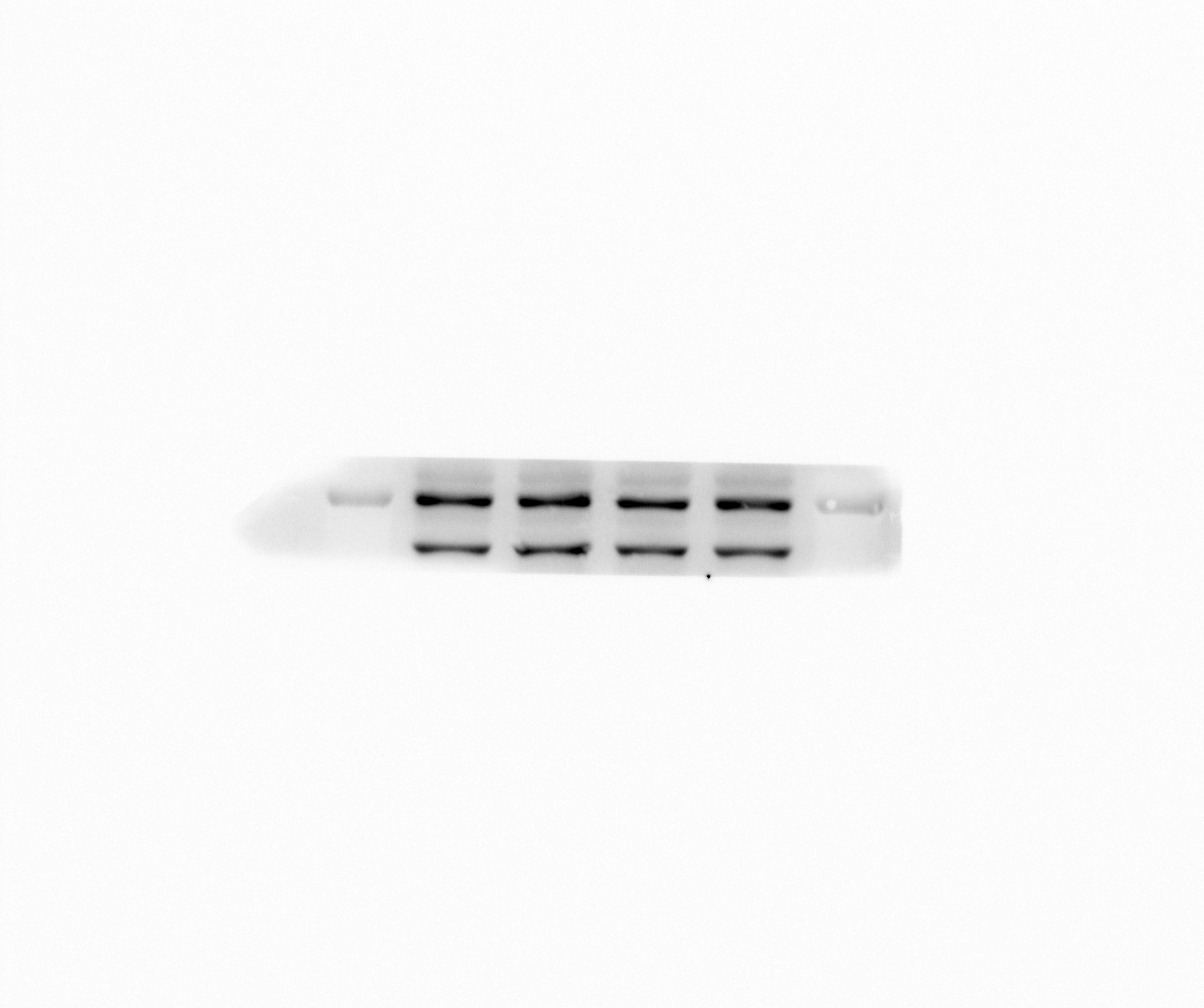

Supplement: Supplementary file 7 [file DataSheet2.ZIP › JNK/JNK.jpg]

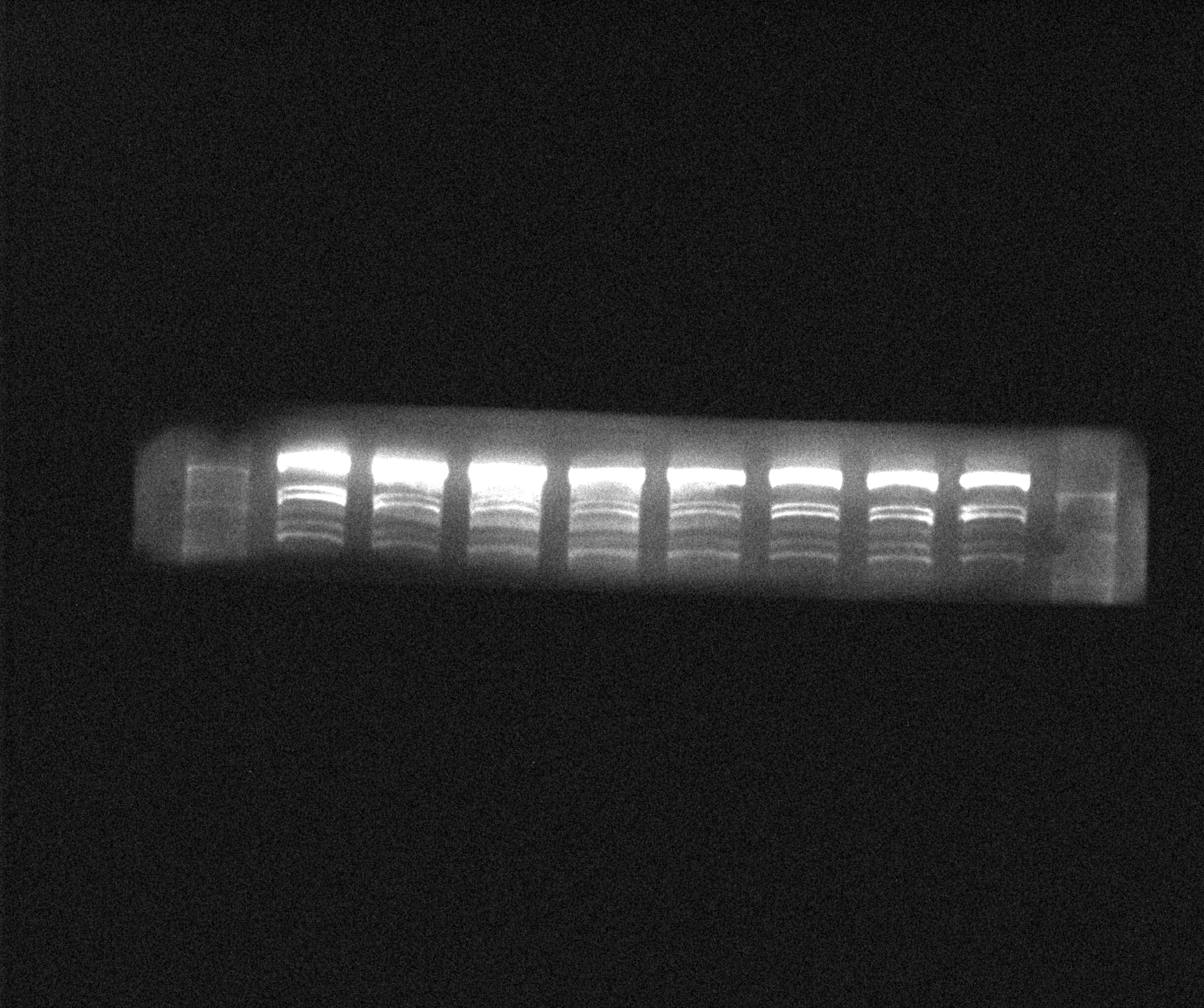

Supplement: Supplementary file 7 [file DataSheet2.ZIP › mTOR/mTOR-B-2.jpg]

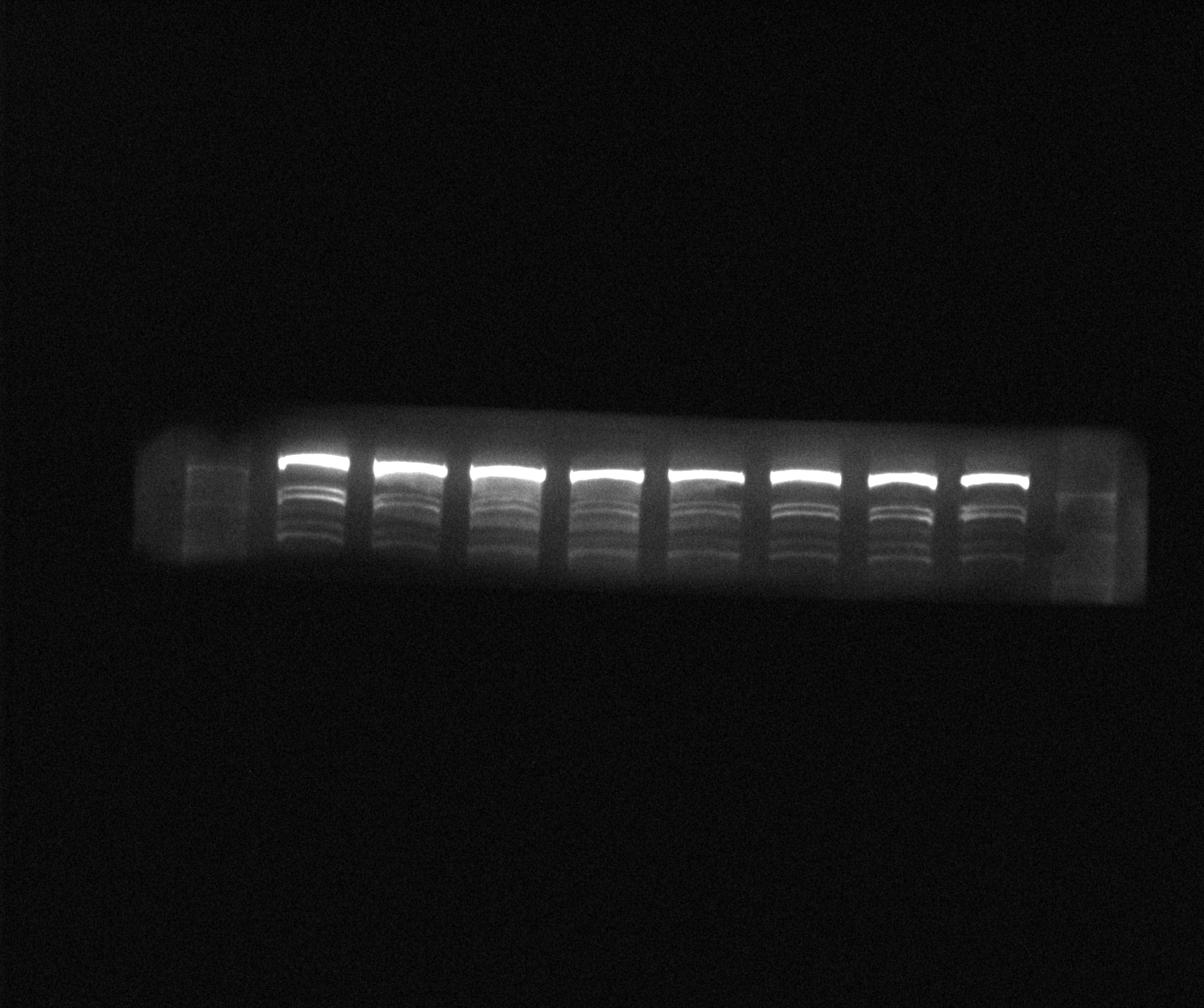

Supplement: Supplementary file 7 [file DataSheet2.ZIP › mTOR/mTOR-B.jpg]

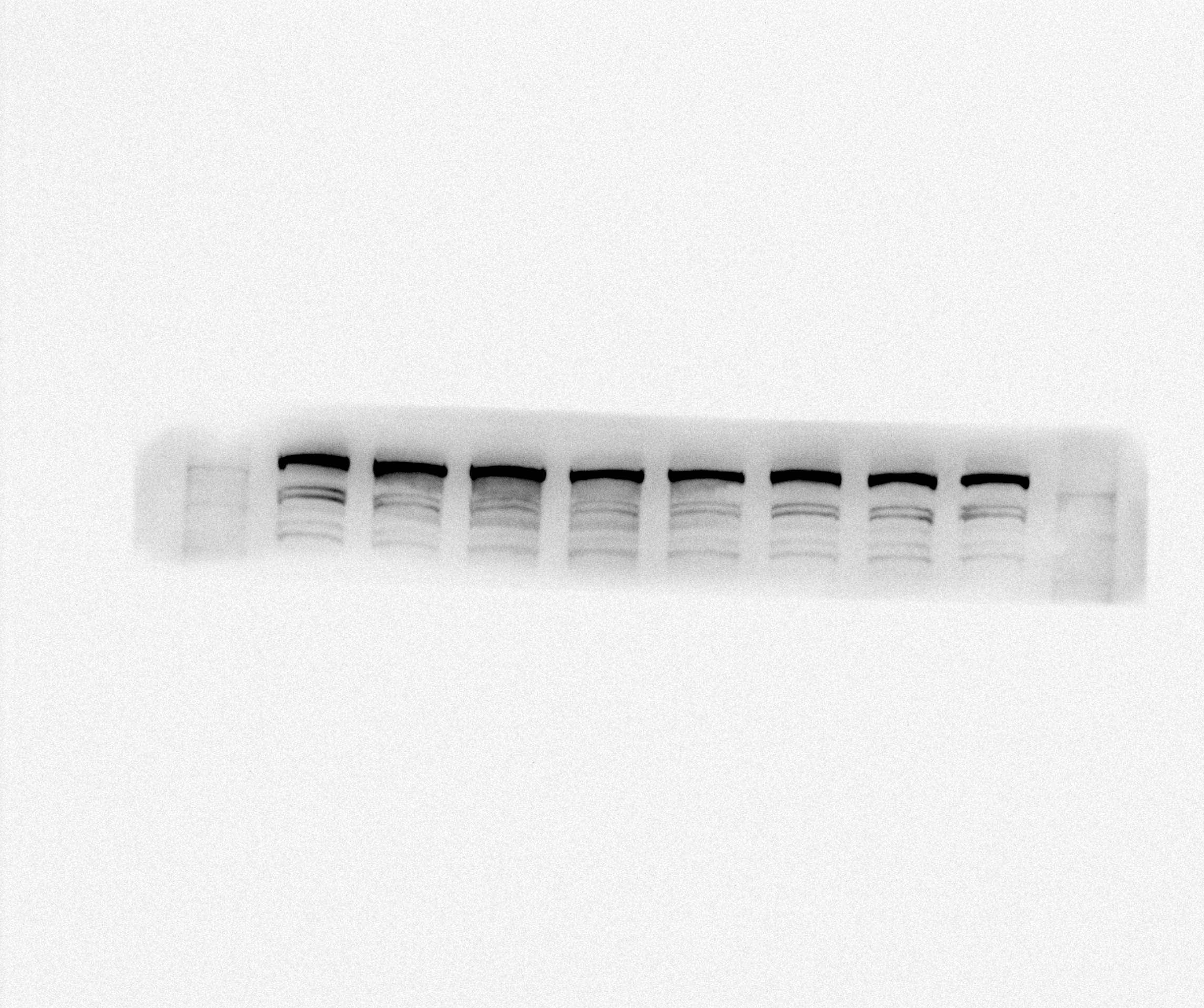

Supplement: Supplementary file 7 [file DataSheet2.ZIP › mTOR/mTOR-F-2.jpg]

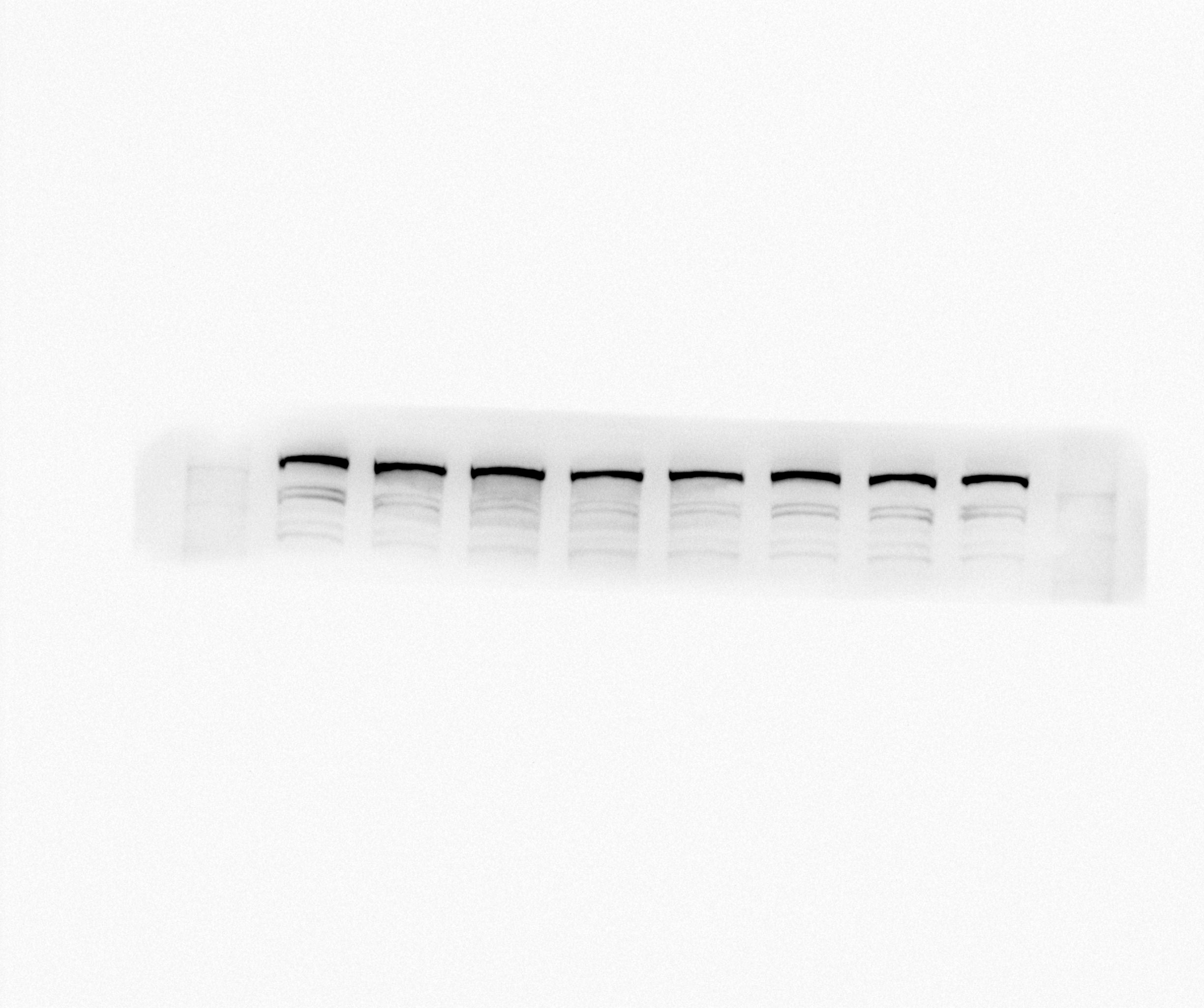

Supplement: Supplementary file 7 [file DataSheet2.ZIP › mTOR/mTOR-F.jpg]

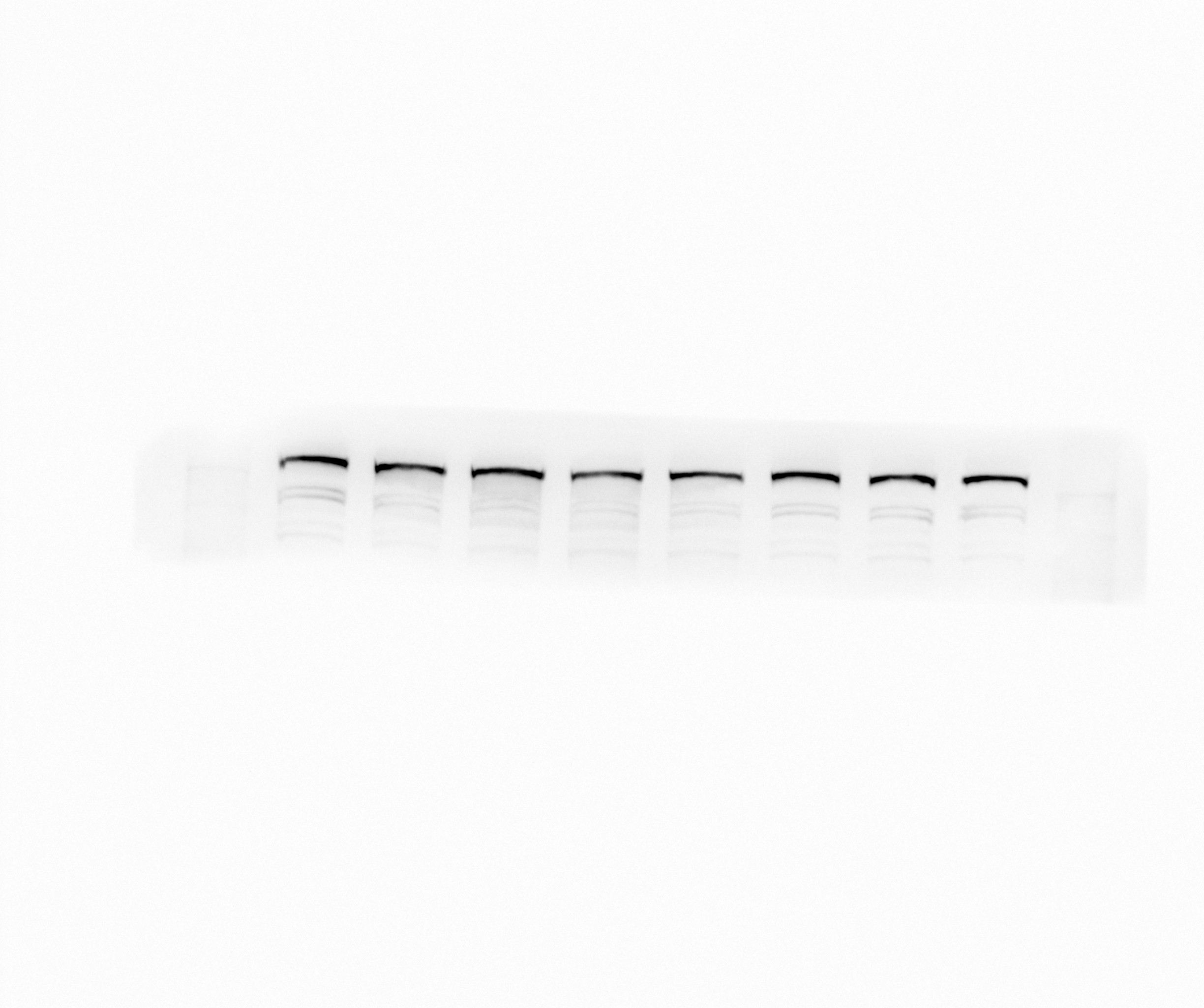

Supplement: Supplementary file 7 [file DataSheet2.ZIP › mTOR/mTOR.jpg]

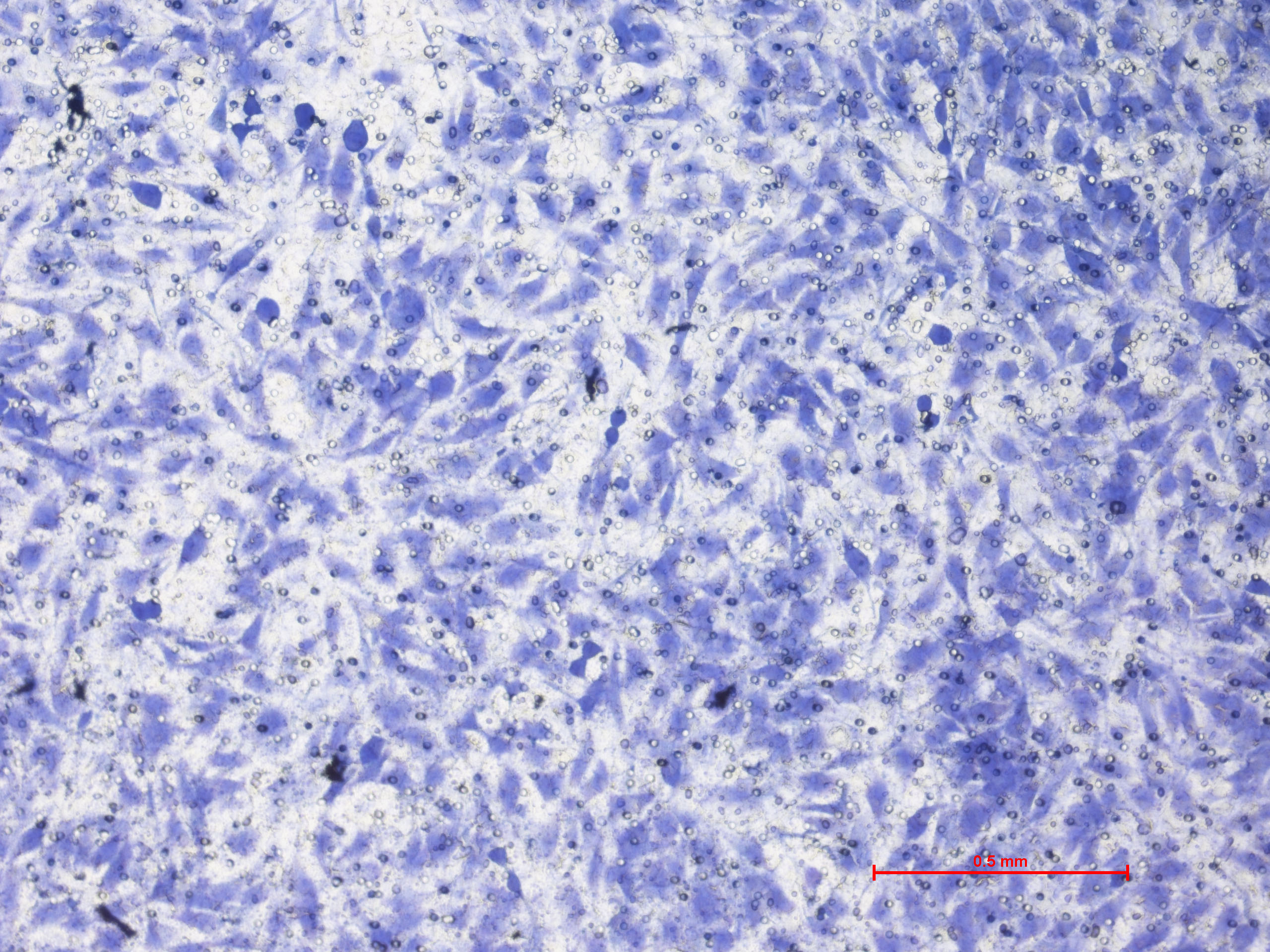

Supplement: Supplementary file 8 [file DataSheet5.ZIP › Fig 1E/Ctrlú¿1ú⌐.jpg]

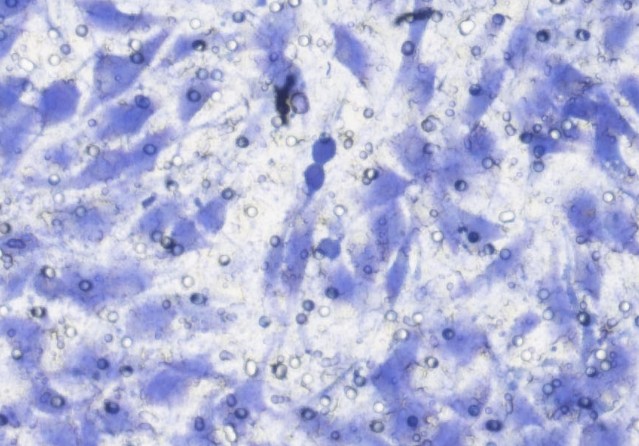

Supplement: Supplementary file 8 [file DataSheet5.ZIP › Fig 1E/Ctrlú¿2ú⌐.jpg]

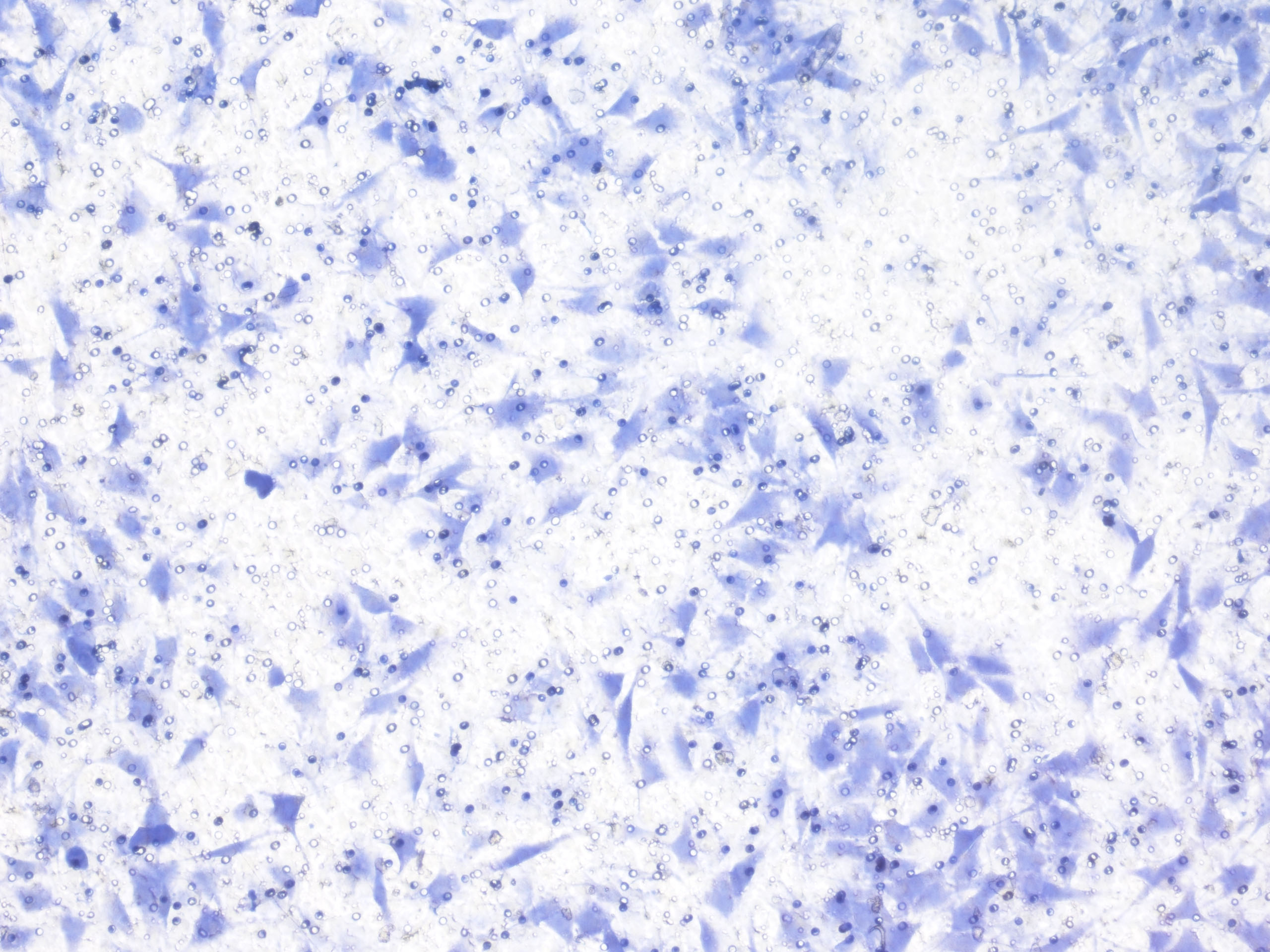

Supplement: Supplementary file 8 [file DataSheet5.ZIP › Fig 1E/PTX 10nMú¿1ú⌐.jpg]

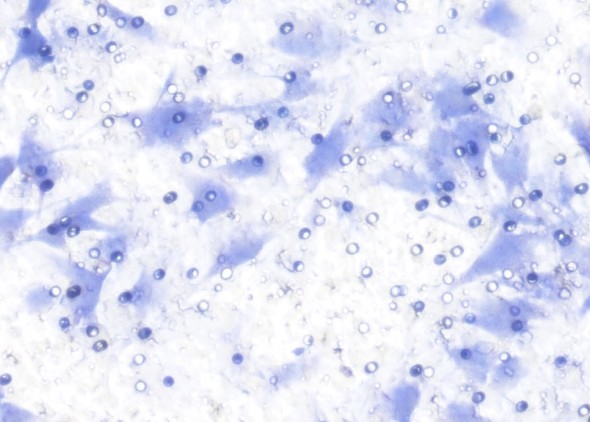

Supplement: Supplementary file 8 [file DataSheet5.ZIP › Fig 1E/PTX 10nMú¿2ú⌐.jpg]

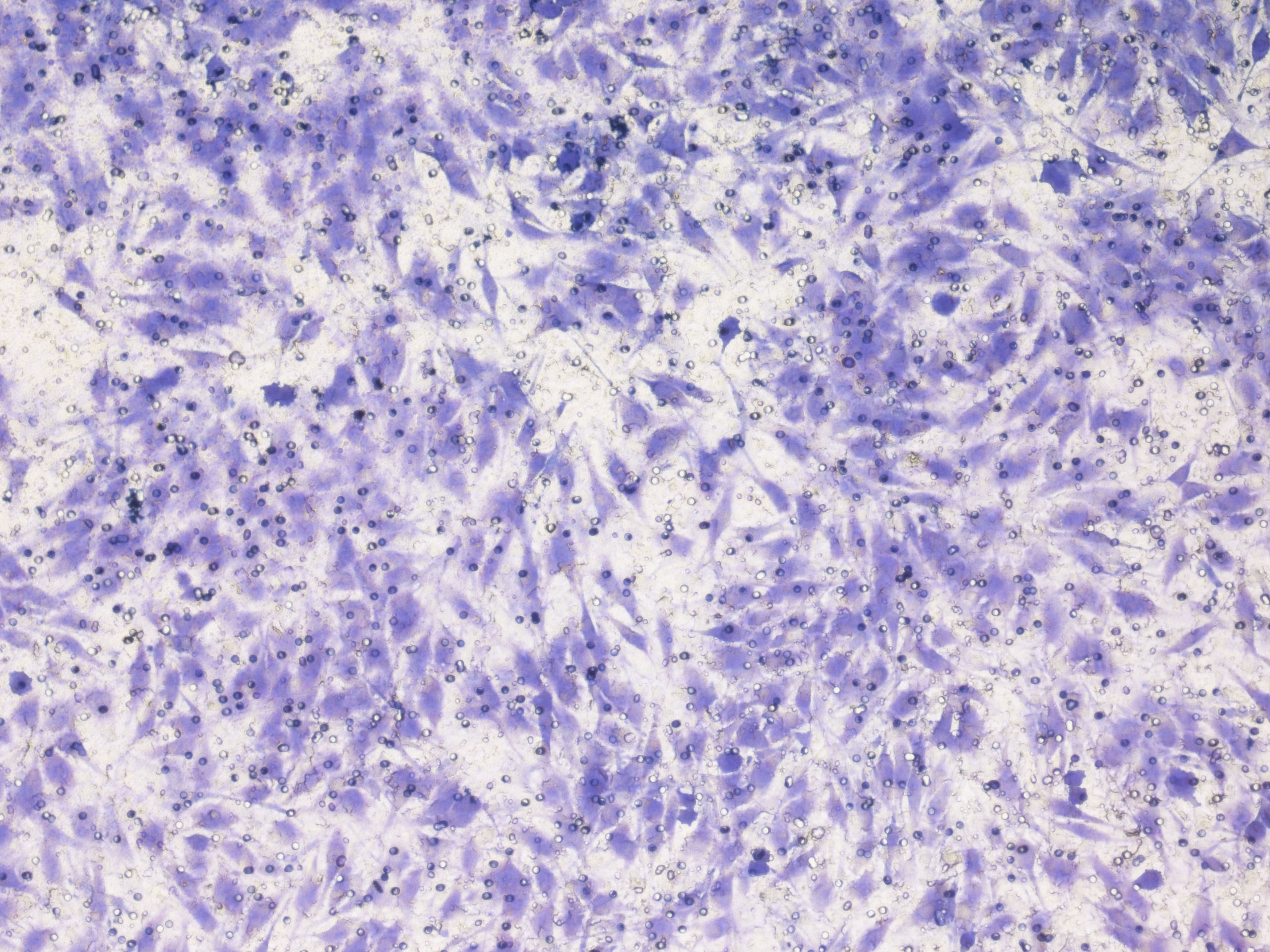

Supplement: Supplementary file 8 [file DataSheet5.ZIP › Fig 1E/PTX 2.5nMú¿1ú⌐.jpg]

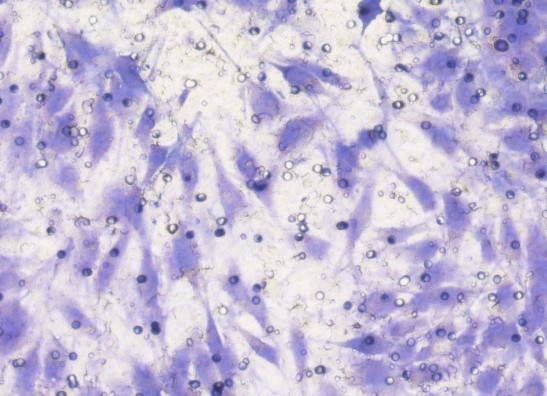

Supplement: Supplementary file 8 [file DataSheet5.ZIP › Fig 1E/PTX 2.5nMú¿2ú⌐.jpg]

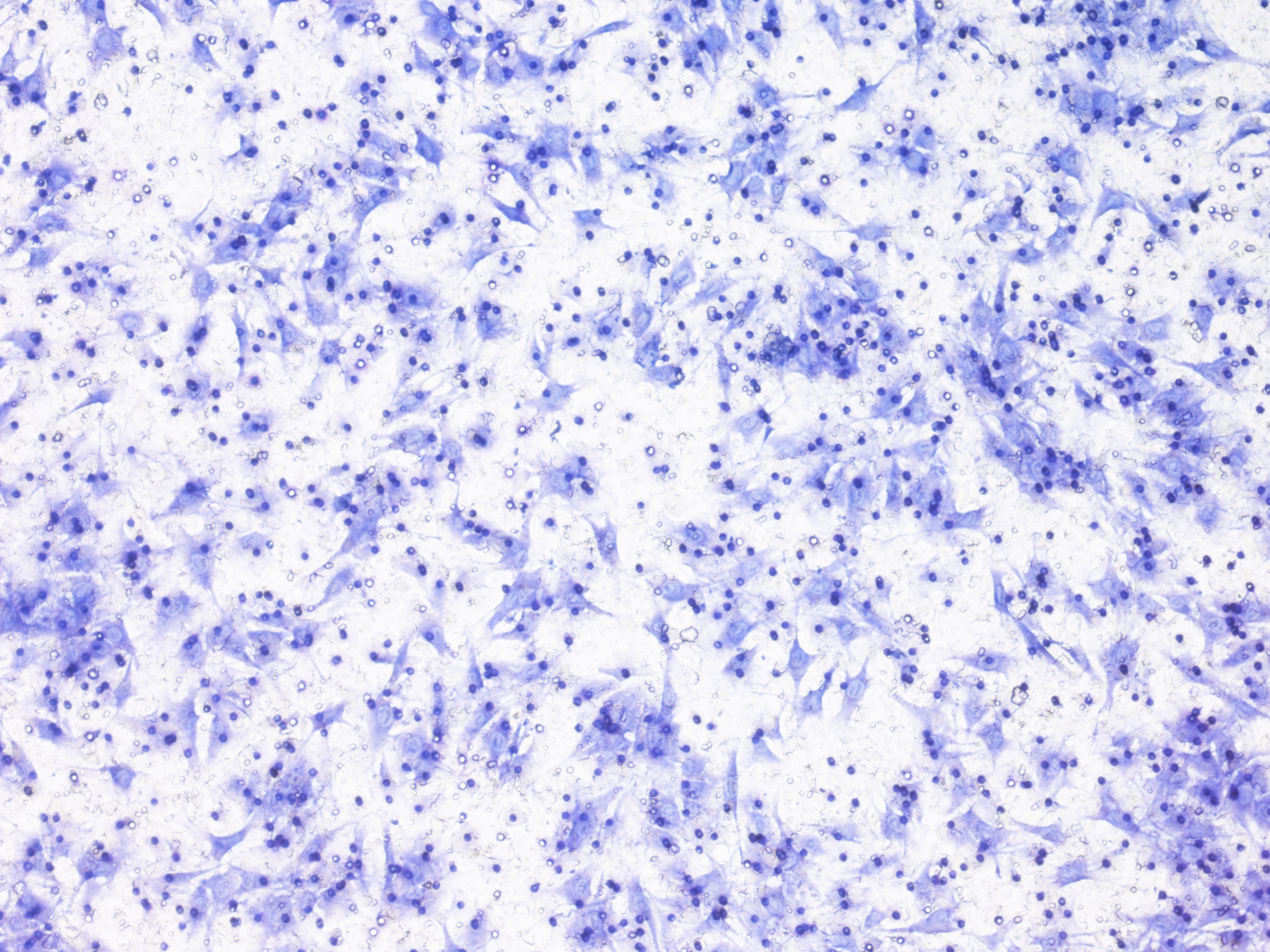

Supplement: Supplementary file 8 [file DataSheet5.ZIP › Fig 1E/PTX 5nMú¿1ú⌐.jpg]

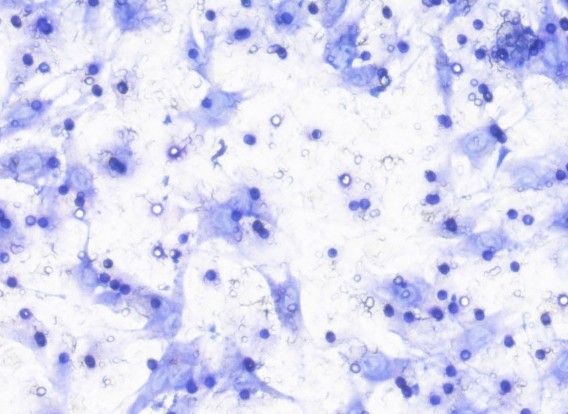

Supplement: Supplementary file 8 [file DataSheet5.ZIP › Fig 1E/PTX 5nMú¿2ú⌐.jpg]
